# A Large Maize (Zea mays L.) SNP Genotyping Array: Development and Germplasm Genotyping, and Genetic Mapping to Compare with the B73 Reference Genome

**DOI:** 10.1371/journal.pone.0028334

**Published:** 2011-12-08

**Authors:** Martin W. Ganal, Gregor Durstewitz, Andreas Polley, Aurélie Bérard, Edward S. Buckler, Alain Charcosset, Joseph D. Clarke, Eva-Maria Graner, Mark Hansen, Johann Joets, Marie-Christine Le Paslier, Michael D. McMullen, Pierre Montalent, Mark Rose, Chris-Carolin Schön, Qi Sun, Hildrun Walter, Olivier C. Martin, Matthieu Falque

**Affiliations:** 1 TraitGenetics GmbH, Gatersleben, Germany; 2 Etude du Polymorphisme des Génomes Végétaux, INRA – CEA – Institut de Génomique – Centre National de Génotypage, Evry, France; 3 Cornell University, Ithaca, New York, United States of America; 4 UMR de Génétique Végétale, INRA – Université Paris-Sud – CNRS – AgroParisTech, Gif-sur-Yvette, France; 5 Syngenta Biotechnology Inc., Research Triangle Park, North Carolina, United States of America; 6 Illumina Inc., San Diego, California, United States of America; 7 Plant Genetics Research Unit, USDA-Agricultural Research Service, Columbia, Missouri, United States of America; 8 Department of Plant Breeding, Technische Universität München, Freising, Germany; University of Guelph, Canada

## Abstract

SNP genotyping arrays have been useful for many applications that require a large number of molecular markers such as high-density genetic mapping, genome-wide association studies (GWAS), and genomic selection. We report the establishment of a large maize SNP array and its use for diversity analysis and high density linkage mapping. The markers, taken from more than 800,000 SNPs, were selected to be preferentially located in genes and evenly distributed across the genome. The array was tested with a set of maize germplasm including North American and European inbred lines, parent/F1 combinations, and distantly related teosinte material. A total of 49,585 markers, including 33,417 within 17,520 different genes and 16,168 outside genes, were of good quality for genotyping, with an average failure rate of 4% and rates up to 8% in specific germplasm. To demonstrate this array's use in genetic mapping and for the independent validation of the B73 sequence assembly, two intermated maize recombinant inbred line populations – IBM (B73×Mo17) and LHRF (F2×F252) – were genotyped to establish two high density linkage maps with 20,913 and 14,524 markers respectively. 172 mapped markers were absent in the current B73 assembly and their placement can be used for future improvements of the B73 reference sequence. Colinearity of the genetic and physical maps was mostly conserved with some exceptions that suggest errors in the B73 assembly. Five major regions containing non-colinearities were identified on chromosomes 2, 3, 6, 7 and 9, and are supported by both independent genetic maps. Four additional non-colinear regions were found on the LHRF map only; they may be due to a lower density of IBM markers in those regions or to true structural rearrangements between lines. Given the array's high quality, it will be a valuable resource for maize genetics and many aspects of maize breeding.

## Introduction

Maize (*Zea mays* ssp. *mays*), along with wheat and rice, is one of the most important crop plants. Being widely grown around the world in tropical and temperate climatic zones, it is important both as a food and feed plant but it has also recently gained additional interest as a renewable energy plant due to its high biomass potential. The cultivated maize was domesticated from the grass teosinte (*Zea mays ssp. parviglumis*). Current maize morphology differs from teosinte mainly because of a few major genes for which specific alleles were selected during domestication [1]. Through selfing, homozygous inbred lines have been developed and in current hybrid varieties a high level of heterosis is achieved through the combination of lines from different heterotic groups.

The genome of maize is approximately 2.3 Gbp which makes it comparable in terms of size to the human genome. The maize inbred B73 has been used as a reference line for sequencing [2]. The current version of the B73 genome assembly covers a “golden path” of 2066 Mb. This assembly is based on the most recent physical mapping data (BAC contig assembly through FPC fingerprinting, optical mapping), integration of molecular markers from genetic maps, and within-BAC sequence assembly [3]–[5]. Like many other plant genomes, the maize genome has undergone several duplication events of which the most recent is a whole genome duplication resulting in a haploid chromosome number of n = 10 approximately 12 million years ago [6], [7].

Maize in traditional populations is a highly heterozygous plant that displays an extremely high level of sequence polymorphism. Through whole genome sequencing of maize inbreds [8], sequencing of genomic fractions with reduced complexity (*i.e.* through the elimination of highly repeated DNA sequences) or transcriptome sequencing, large numbers of SNP markers have been identified. SNP polymorphisms appear, on average, every 44–75 bp [9]. This level of polymorphism is 10 to 20 times higher than in most animal species. Furthermore, it has been found that individual maize lines have extensive structural differences such as copy number variations and presence/absence polymorphisms [10].

To date, over 180 genetic mapping studies have been performed in maize (http://www.maizegdb.org/), based on different mapping populations such as F2 [11], recombinant inbred lines (RILs) [12], and high-resolution Intermated Recombinant Inbred Lines (IRILs) which include several generations of random intermating starting from F2 plants to increase the number of effective meioses before repeated selfing to obtain inbred lines, thus increasing the resolution of the map [13]–[15]. The current biparental reference genetic map for maize is the IBM map based on IRILs obtained from the cross B73×Mo17. An additional population named LHRF that is more relevant to studying European maize material was produced from the cross F2×F252 [16] using exactly the same scheme. More recently, a star-shaped multi-parental mapping experiment called Nested Association Mapping (NAM) [17] was developed using the B73 inbred as the pivotal line.

The analysis of very large numbers of SNP markers in precisely located single copy sequences is a prerequisite towards the elucidation of the detailed genome structure and precision breeding. Arrays with many thousands of SNPs genotyped in a highly parallel fashion [18], [19] as well as new genotyping by sequencing methods [20], are approaches towards this goal. As originally demonstrated for humans, large genotyping arrays with several million SNPs are useful for the analysis of many individuals at a very high genetic resolution. They permit the analysis of traits that are inherited as single locus (qualitative) traits as well as traits that are influenced by multiple loci (QTLs or quantitative traits) to a resolution that leads directly to the identification of candidate genes, using linkage mapping in very large sets of recombinant individuals or genome wide association studies (GWAS). In contrast to the genetic analysis in segregating populations, GWAS studies are based on the precise phenotypic analysis of a given trait in a large set of individuals that are widely unrelated (*i.e.* have no or little family structure) but are derived from a common gene pool. GWAS with large SNP genotyping arrays containing several million SNP markers are now routinely used to identify loci that are associated with many complex traits in human and other organisms [21]. In important domesticated animal species [22], [23], the analysis of large numbers of SNP markers has opened the door to new breeding schemes such as genomic selection (GS). For GS, the effect of all markers present on the array is estimated in precisely phenotyped reference populations through a variety of statistical approaches [24], [25]. Breeding values are subsequently calculated for newly generated, not yet phenotyped progenies based on their genotyping and marker effects estimated in the reference population(s). In cattle, this approach has been so successful that genomic breeding values are now used as reliable predictors for progeny individuals. Recent data suggest that GS is also promising in maize and other plant species [26].

The objectives of the work presented here are (1) to use SNPs previously identified in maize to develop a first reliable and standardized large scale SNP genotyping array; (2) to genotype a set of several hundred maize lines in order to define the functionality over a wide set of maize germplasm and (3) to produce two high-density linkage maps based on biparental IRIL populations for an independent comparison with the B73 genome sequence to identify assignment and ordering discrepancies between the genetic and physical maps.

## Results

### Establishment of an accurate genotype calling procedure through cluster definition

**Figure 1:**
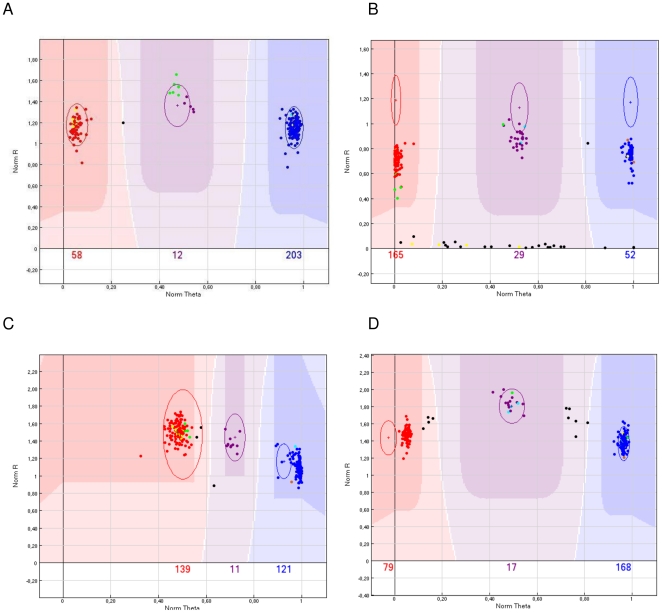
Representative samples from the observed cluster types with maize SNPs based on the GenomeStudio software. The three highlighted clusters display the area where the three different genotypes with homozygous allele A (red), heterozygous AB (purple) and homozygous allele B (blue) are called. Allele calls that fall in the lighter colored areas in between or below these areas are set to ‘failed’. Ellipses are used to adjust the position of the allele calling areas. A) Cluster Type 1: Accurate genotype calling of all three genotypes in essentially all 274 maize lines with clearly defined clusters; B) Cluster Type 2: Three clusters with a number of failed samples at the bottom of the analysis plane that are not called; C) Cluster Type 3: Three clusters with a shift towards one side which is indicative for a duplicated locus. Clusters have to be shifted to the left for accurate allele calling; D) Cluster Type 4: Five clusters which are indicative of two simultaneously scored polymorphic loci in a duplicated sequence. Such markers cannot be scored accurately, so they were deleted from the data set.

**Figure 2:**
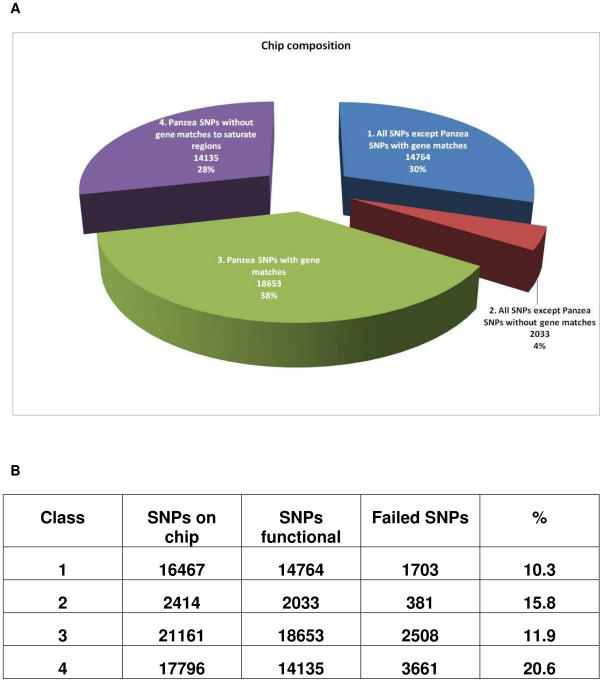
Distribution and success rates of SNP markers on the Infinium array. A) Numbers and percentage of SNP markers from the different marker groups on the array, showing that most of the SNPs coming from sources other than Panzea were located in genes; B) Success rates for the individual marker classes. Numbers are based on all 57,838 SNP markers manufactured.

From the 57,838 synthesized SNP markers, 56,110 markers passed bead representation and decoding quality metrics. When the cluster distribution of the genotypes from the 274 maize lines was analyzed, it was found that the distribution of the genotype calls in the two-dimensional analysis was producing mainly four distinct patterns (Figure 1). Pattern Type 1 was represented by three clearly defined clusters representing the three possible genotypes (AA, AB and BB). Such markers did not require any significant adjustment for the genotype calling as the genotyping software algorithm identified 3 clusters. Usually this pattern was observed with markers that could be scored in all or nearly all 274 maize lines. Pattern Type 2 was obtained with the majority of the markers on the array and was similar to the Pattern Type 1. However, frequently the cluster corresponding to the heterozygous situation was not as compact as in Type 1 and often a significant number of lines were not automatically scored due to lower than expected signal intensities. With the appropriate adjustment of the area for the respective genotype, such a marker could be scored accurately with the genotypes of the samples producing very weak signal intensities set to failed. The Pattern Type 3 also produced three defined clusters but two of those clusters were shifted to normalized theta space along the X axis of the SNP graph (normalized theta ranges from 0–1 corresponding to an angle relative to the “A” allele intensity of 0–90 degrees, respectively). Such a marker could not be easily interpreted with the GenomeStudio software [27]. In Pattern Type 3, the genotype clusters required manual adjustment in a significant manner so that the three genotype classes could be called accurately. In cases when the three clusters were very close and shifted strongly to one side of the theta space, the GenomeStudio software did not permit the definition of the three different genotypes and the marker had to be scored as failed. Such markers often occurred in groups when the markers were positioned along the maize genome sequence. The final Pattern Type 4 resulted in five instead of three clusters. These markers could not be scored with the GenomeStudio v2009 software and were thus set to failed. After evaluating all 56,110 markers, 49,585 passed the analytical phase. Figure 2 shows the final attribution of the 49,585 functional markers to different groups used for the SNP selection procedure, as well as the individual functionality rates for each group. Among the 49,585 scorable SNPs, 34,182 came from Panzea, 13,037 from Syngenta, 1,816 from INRA, 400 from TraitGenetics, and 150 from other sources. These markers covered a total of 17,520 different genes, with sometimes numerous markers in the same gene, which may increase resolution for gene haplotyping.

### Quality control of the markers for pedigree consistency, reproducibility and call rate in the different maize samples

In addition to the definition of the genotype clusters based on the distribution on the two-dimensional plane of the genotyping software, further quality control steps were performed such as the analysis of the markers in a number of parent/F1 triplets for pedigree consistency and in DNA duplicates for technical reproducibility. 35 triplet combinations including 25 parent/F1 combinations for the NAM populations, 9 parent/F1 combinations from European maize material and the parent/F1 combination of B73/Mo17 (IBM population) were used for determining the pedigree consistency of the genotype calls based upon Mendelian expectations. The consistency of the genotype calls was also determined in technical (same DNA analyzed twice) duplicates (three samples each from B73, Mo17 and the F1) and sample duplicates from different seed sources. In summary, the allele calls were highly reproducible (Table S1) with no or negligible inconsistencies observed between samples with respect to genotype calls in technical replicates. A slightly higher level of inconsistency (up to 1%) was observed with duplicated samples from different seed samples and sources. Similarly, a pedigree inconsistency of less than 1% was observed in the analyzed triplets further confirming the quality of the genotype calling with the array. Also, for the three DNA replicates of B73, Mo17 and the hybrid, highly similar but not identical numbers of markers (B73: 49,546–49,560 out of 49,585; Mo17: 48,719–48,737 out of 49,585; B73×Mo17 F1: 48,795–48,889 out of 49,585) could be scored.

**Figure 3:**
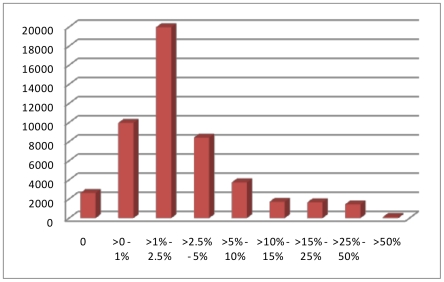
Number of SNPs with their corresponding failure rate. Failure rates are presented in % for the 49,585 markers analyzed in the 274 maize samples based on the MaizeSNP50_B.egt cluster file.

**Figure 4:**
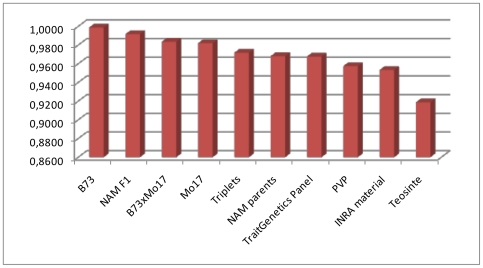
Success rate for the 49,585 markers in the different sample groups.

In the next step, the cluster file was used to score all 274 maize samples and the quality of the genotyping data was assessed with respect to the failure rate on a marker by marker basis (Figure 3 and Table S2). The 49,585 markers that could be scored with the established cluster had an average failure rate of 4.0% corresponding to an average of 11 samples per marker. However, 8,628 (17.4%) of the markers had a failure rate of more than 5% although these markers produced the correct genotypes in the analyzed triplets as far as they could be fully analyzed there. To further elucidate the reasons for the relatively high failure rate for the maize SNP markers, a more detailed analysis was performed for individual groups of maize lines (Figure 4). These data revealed that the highest call rate (0.9987) was obtained for B73 and the lowest (0.9187) for the teosinte samples with additional major groups such as the INRA material with European inbreds and the expired PVP inbred material from the US [28] in the middle, suggesting a correlation with increased sequence divergence relative to B73 and other materials used to discover intital polymorphisms. Detailed information for each SNP, including source and genotypes across all 274 lines is given as Table S3.

### Level of polymorphism between lines and potential ascertainment bias

The SNP markers were selected from different sources: many via the B73-Mo17 pair (especially the Syngenta SNPs, see also Material and Methods), many as well from the NAM material which represented a more comprehensive sample of the maize germplasm, and still others from polymorphisms between key sets of lines (Table S4). A very high number of polymorphisms was observed for the combination of B73×Mo17 with approximately 25,325 SNPs (numbers differ slightly between individual samples due to variation between the samples, see above). A dendrogram generated using all markers illustrated that Mo17 was one of the most distant lines compared to the other analyzed samples and that the unadapted teosinte material was slightly less distant in the dendrogram than Mo17. This was mainly due to the Syngenta SNPs which were specifically selected for their high value in detecting polymorphism between B73 and Mo17. When only the less biased Panzea markers (derived from the diverse set of NAM parents) were considered, the position of Mo17 in the dendrogram changed significantly and to a more expected position (Figure S1).

### Analysis of the functional markers in comparison to the maize genome sequence

To include markers in as many maize genes as possible, one of the criteria during the original marker selection was their presence in maize genes. Originally, for 19,350 maize genes at least one SNP was put on the array. After the elimination of the markers that could not be scored, the 49,585 SNPs consisted of 33,417 SNPs located in 17,520 genes and 16,168 SNPs located in intergenic regions. The minimum number of SNPs in a gene was 1 and the maximum number was 15 SNPs (Table S5). Altogether 17,520 genes could be analyzed with the established cluster file for the 49,585 markers, whereby the vast majority (15,404 or 88%) of these genes contained from one to three SNPs. The details about the number of SNPs per gene and their correspondence to the respective filtered gene or other maize genes are displayed in the Table S6.

Because the remaining 16,168 SNPs were not in known maize genes, the full set of the 49,585 SNPs were located onto the maize B73 reference sequence (AGPv2). In general, markers were well-distributed over the chromosomes with lower numbers of markers per megabase in many centromeric regions and a slightly higher number of markers per megabase near the telomeres.

While typically there was an uncovered region of approximately 1 Mbp on most of the chromosomes, on maize chromosome 6, there was a region of more than 2 Mbp that did not contain a single SNP. The distribution of distances between adjacent markers shows that most distances are a few kilobases, but nevertheless for a significant number of regions the distance between adjacent markers was more than 100 kb.

### The IBM and LHRF framework maps

All polymorphic markers were first analyzed for segregation bias in the allele frequencies within the two mapping populations. Some regions were skewed (Figure S2), with close markers generally distorted towards the same parent. On chromosome 3, the IBM and LHRF populations showed similar distortion patterns, suggesting the presence of common sources of segregation bias in both populations. Some markers are clear outliers, falling outside of the patterns, confirming the need for filtering out markers with high fractions of missing data or extreme distortion.

After removing low-quality markers, 24,816 SNPs were used for mapping on the IBM population and 17,047 on the LHRF population. 8,883 SNPs were common to both populations. The genomic distribution of mapped markers for each population is given in Figure S3. The IBM and LHRF linkage maps were constructed independently using precisely the same procedures and parameters. To generate maps that are independent of the B73 genome sequence, the map construction was based only on the SNP genotyping data. The theoretical resolution of the IBM and LHRF maps, based on populations composed of 239 and 226 IRIL individuals respectively, should be better than 0.1 cM if there are enough markers. Neglecting heterogeneity in the spacings of our 24,816 or 17,047 marker, such a resolution seems accessible. However, for such high numbers of markers, especially if they are heterogeneously spaced, many will be indistinguishable, preventing one from building maps with robust marker orders. Thus, as a first step, a scaffold map was constructed with a limited number of markers, but whose order is very reliable. The IBM (respectively LHRF) scaffold obtained contained 311 markers separated by 4.7 to 11.4 cM (respectively 345 markers separated by 4.5 to 10.0 cM). The scaffolds were then augmented by adding as many markers as possible while keeping the robustness of marker order above a threshold. This produced an IBM framework map with 1,934 markers separated by 0.2 to 12.3 cM and a LHRF framework map with 1,785 markers separated by 0.2 to 14.5 cM. The total map length was 1,689 cM for IBM and 2,168 cM for LHRF (Figure S4 and Table S7).

### Mapping of all additional polymorphic SNPs

The two framework maps were limited in their marker number by the constraint of maintaining high statistical robustness of the ordering. Given a framework map, additional markers can be placed by determining their positions without including them explicitly into the map. This approach is mandatory for mapping high numbers of markers using relatively small populations. For the IBM population, this placement led to a high density map with 20,913 markers of total length 1,725 cM with a largest gap size of 11.6 cM. For the LHRF population, we obtained a high density map with 14,524 markers, a total length of 2,208 cM, and a largest gap size of 12.1 cM. Furthermore, a total of 7,368 SNPs monomorphic on IBM could be mapped on LHRF, so the use of LHRF increased the overall number of markers that could be genetically mapped by 35%. The results are presented in Figure S5 and detailed in Table S8 for all mapped markers.

### Genome-wide and chromosome-specific comparison between the genetic maps and the B73 genome sequence

Among the 49,585 markers used, over 400 markers, or approximately 1% could not be unambiguously located on the B73 genome sequence using a BLAST search. The majority of these unassigned markers were not found at all in the B73 reference sequence indicating that the corresponding genomic sequence was missing in the current assembly. Among such markers, 172 could be mapped on one or both of the genetic maps, indicating a potential location of the associated genomic sequence on a maize chromosome (Table S9).

**Figure 5:**
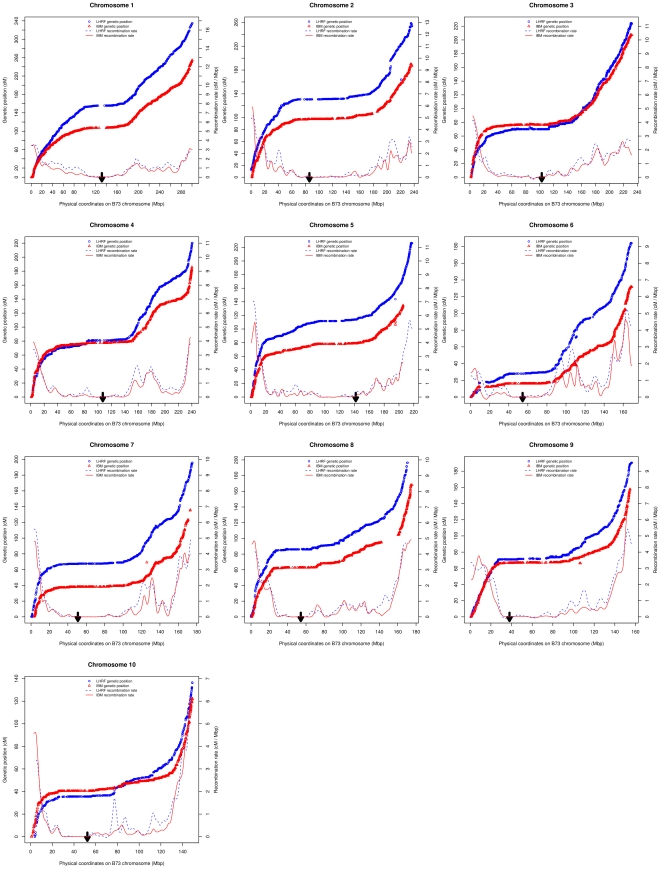
Relationship between physical and genetic positions, and corresponding recombination rates. X-axis: physical position (in Mbp) of the SNPs on the B73 physical map. Left Y-axis: genetic positions of SNP markers on IBM (red triangles) and LHRF (blue circles) linkage maps. Right Y-axis: recombination rate in centiMorgan per Mega base pair for the IBM (solid red line) and LHRF (dashed blue line) maps. Recombination rates were obtained as the first derivative of the smoothed curve representing genetic versus physical positions. The thick arrow indicates approximate centromere position according to MaizeGDB.

Figure 5 shows the relationship between genetic and physical positions on all chromosomes, along with the first derivative of this relationship providing an estimate of the meiotic recombination rate. In all chromosomes (less for chromosome 6), recombination occurred predominantly near both telomeres whereas very large pericentromeric regions were almost devoid of recombination. Variations in recombination rate along the chromosomes were similar in both genetic maps except for some highly recombining regions that were specific for one cross such as in the middle of chromosome 10 for the LHRF map.

**Figure 6:**
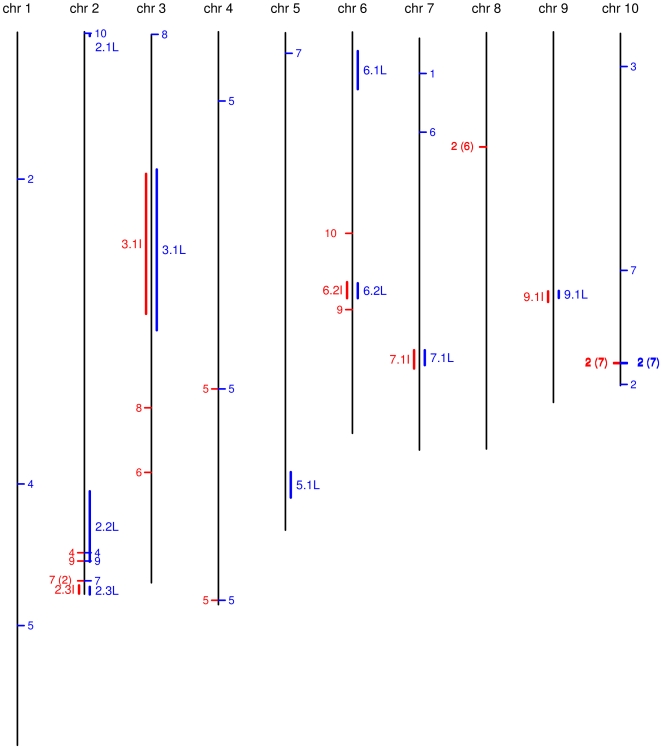
Non-conserved chromosomal assignments or non-colinearity between the genetic maps and the B73 sequence. Positions on the black vertical lines are according to the physical coordinates on the B73 genome. Red (blue) ticks on the left (right) side of the lines indicate the positions of the markers that are mapped on a different chromosome in the IBM (LHRF) genetic map as compared to the B73 genome. Numbers beside the ticks indicate the chromosome onto which the respective markers were genetically mapped. Numbers in parentheses indicate the number of differently mapped markers. Thick red (blue) lanes on the left (right) side of each chromosome line indicate the regions with markers mapped on the same chromosome, but with significant order disagreements between IBM (LHRF) genetic maps and the B73 genome. The name of each such non-colinear regions, indicated besides the thick lane, starts with the chromosome number followed by a dot, and ends with “I” for IBM or “L” for LHRF. The same names are used in Table S11.

Among 20,788 (respectively 14,432) markers genetically mapped on the IBM (respectively LHRF) map and which were physically placed on the B73 sequence, 23 (respectively 24) did not show conserved chromosomal assignments (Figure 6). Some of these non-syntenic markers were singletons, but in other cases they formed clusters. There were cases of singletons and clusters where both the IBM and LHRF genetic maps provided the same chromosomal assignments, yet were non-syntenic with the B73 genome. For instance, on chromosome 10 of the B73 sequence, there was a cluster of seven markers that were mapped to chromosome 2 in both genetic maps strongly suggesting that these markers were erroneously positioned on the B73 genome sequence. In another case, there were six markers forming a cluster in the B73 sequence on chromosome 8 that the IBM map assigned to chromosome 2 whereas the LHRF map showed no assignment discrepancy with the B73 genome on chromosome 8. Table S10 lists all markers that were non-syntenic between genetic and physical maps, as well as their inferred physical position on the B73 genome based on their genetically determined chromosome assignment and positions.

Colinearity within chromosomes was determined through the genome-wide comparative analysis of physical and genetic positions of shared markers (Figure 5), based on the complete genetic maps. Some physical segments were devoid of markers on the two genetic maps (*e.g.* the centromeric region of chromosome 1) or specifically in one of the two maps. For instance, a large segment of approximately 15 Mb was completely lacking any markers on chromosome 8 of the IBM map. Otherwise, the coverage was quite dense and the maps mainly colinear. A first class of exceptions within chromosomes consisted of individual markers that lied far away from the common pattern of the other markers. These were quite rare, but interestingly they were often outliers for both the IBM and the LHRF maps (*e.g.* on chromosome 5). A second class of exceptions consisted of groups of markers generally corresponding to small inversions between the genetic and physical map (*e.g.* on chromosome 3 at position 85 Mb in both IBM and LHRF, see Figure 5). Since small inversions would require larger populations to reach a high confidence level, these were not considered further.

Based on the framework maps, we then defined regions containing *major* non-colinearities with the B73 sequence (Figure 6 and Table S11). These regions involved several markers of one of the genetic maps, or involved one marker but overlapped with a non-colinear region of the other genetic map. This led to five regions for the IBM genetic map (2.3I, 3.1I, 6.2I, 7.1I, and 9.1I) and nine (2.1L, 2.2L, 2.3L, 3.1L, 5.1L, 6.1L, 6.2L, 7.1L, and 9.1L) for the LHRF genetic map. Interestingly, all five IBM regions closely overlapped with LHRF regions (see Figure 6 and Table S11): this concordance strongly points to probable errors in the B73 sequence assembly. On the other hand, the remaining regions (2.1L, 2.2L, 5.1L, and 6.1L) could be suggestive of structural rearrangements between parental lines of the two populations.

**Figure 7:**
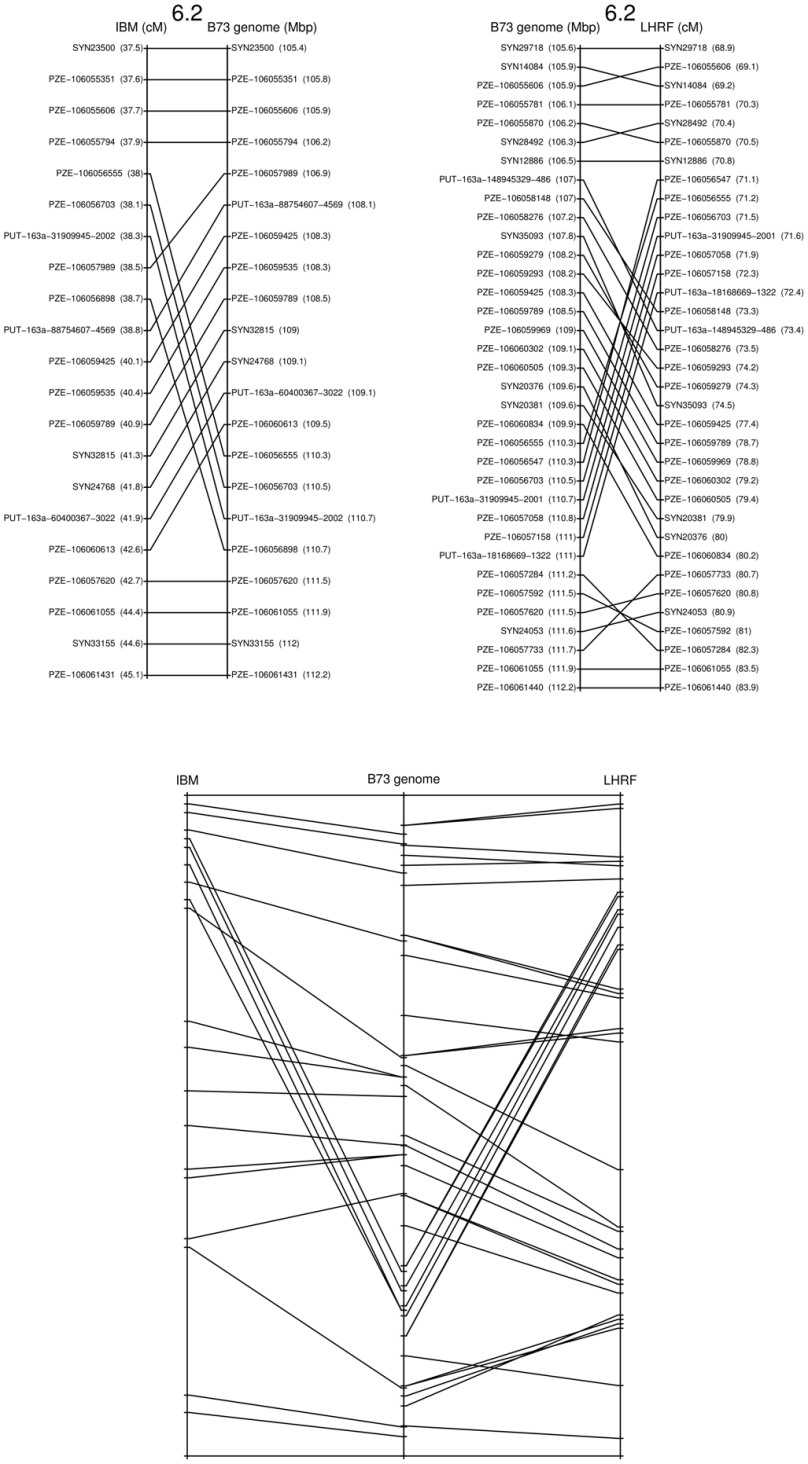
Region 6.2 showing major marker order differences between the genetic maps and the B73 sequence. The complete genetic maps (with framework as well as placed markers) are shown for the IBM and LHRF map in comparison with the B73 genome for the region 6.2 defined in Figure 6 and Table S11. In the ladder diagrams of the upper panel, positions of the markers indicate only their index. Numbers in parentheses indicate the map coordinate in cM for IBM or LHRF genetic maps, or in Mbp for the B73 genome sequence. In the lower panel, positions are proportional to the map coordinates in cM or Mb. Scales were adjusted to fit the two maps to the same height.

Using framework maps alone led to order robustness. However, their coverage is low and potentially misses small regions of non-colinearities or lacks power in revealing the fine structure of non-colinearities. Thus, the complete maps were used to further analyze these regions (e.g. in Figure 7; all regions are shown in Figure S6); this analysis reinforced the evidence for non-colinearities and refined their structure. The extra resolution also revealed a non-colinearity between the IBM genetic map and the B73 sequence in the region 5.1L compatible with that observed on LHRF, suggesting in this case an error in the B73 assembly rather than a structural rearrangement between maize lines.

## Discussion

### Array development and array characterization

Starting from over 800,000 SNPs that were identified in a number of SNP discovery projects, a set of 57,838 SNPs was selected for synthesis and manufacture. The marker selection was based on the fact that maize is a highly polymorphic plant species with a low level of linkage disequilibrium (LD). Especially in unadapted or wild maize material, LD extends only from several hundred base pairs to several kilobases [29]. Because polymorphism within genes or their close vicinity are expected to be the main basis of phenotypic variation, in the SNP selection process a first priority was given to SNPs located in genes. SNPs in genes are potentially more informative in GWAS studies and thus it was attempted to cover as many genes as possible based on the filtered gene or high confidence gene set [2]. With 17,520 genes containing at least one SNP, more than 50% of these high confidence genes could be covered. Another 16,168 SNPs were used to populate other regions of the maize genome with markers and to obtain a relatively even marker distribution. In theory, an even distribution of 50,000 markers would result in an average distance between markers of 40–50 kilobases. In reality and with the focus on one or more SNPs in maize genes, the distance between many adjacent SNP markers was much lower (in the range of a few kilobases). In other regions, the distance between markers went up to hundreds of kilobases because the maize genome contains large stretches of highly repeated sequences that cannot be used for SNP analysis on arrays.

Compared to animal species for which large genotyping arrays have been developed [22], [23], the MaizeSNP50 array contains a relatively large number of markers that were difficult to score or had to be dropped from the analysis altogether. Furthermore, compared to mammalian species where significantly more than 99% of all marker/individual combinations could be scored, in maize on average, only approximately 96% of all marker/individual combinations could be scored. Also, in parent/offspring triplets, more than 300 markers did not produce the correct genotype in the F1 compared to the two inbred parents. This relatively low marker functionality has probably two causes: The first is that maize, compared to animals, has a much higher level of genetic variation. While in animals and humans [30], [31], on average one SNP is observed about every kilobase, in maize there is a 10–20 times higher genetic variation. Depending on the analyzed germplasm, on average, one SNP appears every 44–75 base pairs. With such a high level of SNP polymorphism, it is very likely that in different maize lines there will be adjacent SNPs within the approximately 20 base pairs that are necessary for the Infinium assay primer. If this is the case, then it is very likely that the respective SNP will fail in the respective line(s). The percentage of generated SNP data points for a given line is roughly correlated with its genetic distance to B73. Indeed the reference sequence represented by B73 [2] has the highest success rate over all assays, reaching 99.87% while the genetically highly diverse teosinte had only a success rate of, on average, 91.87%. A second source of low marker functionality is the fact that maize is an ancient polyploid species with large genomic regions that have been duplicated in its evolutionary past. Due to this genome duplication, many maize genes have a second copy at another position in the maize genome that differs by a varying extent. A considerable number of these duplicated genes have a sequence diversity of significantly less than 10%. Thus, it is very likely that a number of SNP assays detect not one locus but multiple highly similar paralog(s). This is confirmed by the observation that in maize, a considerable number of SNP markers show a pattern (Pattern Types 3 and 4 as described in the Results) that are indicative of detecting more than one locus (shift of the clusters to one side or five clusters) as it is found in true tetraploid species [27]. Markers with such a pattern occurred frequently in groups at specific positions that correspond to the duplicated regions identified in the maize genome sequence. As a result, the number of robust markers on the array is 49,585 from a starting point of 56,110.

Another aspect that has to be considered regarding this array is that the employed SNPs are biased towards SNPs identified between B73 and Mo17; particularly the Syngenta marker set was specifically selected for detecting polymorphism between these two lines, and this marker set was extremely valuable for mapping a large number of markers in the IBM population. For diversity studies within cultivated maize, it is thus recommended to use only the Panzea set of markers as this set was unbiased for polymorphism between any specific lines. For teosinte which has not been included in the majority of the original SNP identification, one has to be aware that the ascertainment bias might underestimate polymorphism there too.

### Use of the array for the generation of highly saturated genetic maps and independent validation of sequence assemblies

To produce high-resolution linkage maps that may help improve the B73 genome assembly, the developed array was used for the analysis of two mapping populations. 20,913 polymorphic markers in the IBM mapping population (B73×Mo17) and 14,524 polymorphic markers in the LHRF population (F2×F252) could be mapped, demonstrating that with this array extremely large numbers of polymorphic markers can be analyzed rapidly and high density genetic maps containing many thousands of markers can be generated. The array data from the two populations increase the number of mapped markers significantly compared to previously published data.

The main interests of the mapping data from these two populations and the associated novel findings are twofold: (1) with such high density genetic maps that are generated without the use of the B73 reference sequence, it is possible to independently check and validate the current B73 reference sequence for inconsistencies, and (2) the data permit a precise comparison of physical distances with genetic distances, revealing the variation of meiotic recombination rates in either cross at a much higher resolution than before.

The genetic mapping of markers that are not present in the current sequence assembly of the B73 genome will permit an improvement of the genome sequence assembly. Most regions of the IBM and LHRF maps agree in their marker order with the B73 reference sequence, indicating a good reference assembly (e.g., BAC clone assemblies). However, a limited number of regions and markers have been identified that are non-colinear with the B73 genome. While discrepancies with individual markers could be caused by various reasons such as detection of a polymorphism in a paralogous sequence or double crossovers, discrepancies that occur between both maps and the B73 genome sequence provide solid evidence of problems in the B73 genome assembly. These include a region containing seven markers assigned on the B73 sequence to chromosome 10 that map in both genetic maps to maize chromosome 2, and five larger regions on chromosomes 2, 3 6, 7 and 9 for which the B73 genome sequence appears to be non-colinear with both genetic maps. In principle, the non-colinearities could also be caused by structural rearrangements between B73 and Mo17; however, if one considers for instance inversions, one would expect a severe local suppression of recombination. In the cases observed here, the markers mapped without such suppression in both populations, so the non-colinearities may not be explained in this way, suggesting other phenomena. There are also four regions where the LHRF framework map shows a clear order discrepancy with the B73 genome assembly and possibly with the IBM genetic map, whereas the IBM map is colinear with the B73 genome. Some of these regions may contain actual large scale structural differences between B73/Mo17 compared to the F2/F252 inbreds; indeed, different maize lines are known to possess significant structural differences amongst each other. In that respect, the two genetic maps generated with this array proved to be particularly useful to compare marker orders with the B73 physical genome sequence. Since in a typical population many thousands of markers will be polymorphic, genetic maps generated in the future with the array will permit genetic map comparisons from different crosses at a resolution that was not possible previously.

It is now possible to also precisely locate chromosomal regions where the level of polymorphism differs significantly between our mapping populations. For example on chromosome 8, a large segment of the physical map is devoid of polymorphic markers on the IBM genetic map, whereas many of the respective markers are polymorphic in the LHRF genetic map. This provides a strong indication that the two IBM parents are identical by descent in this region, a possibility that was previously suggested by Springer et al. [10] based on the local absence of CNV polymorphism.

Considering the genetic/physical distance comparisons, such high density genetic maps can also be used to precisely pinpoint chromosomal regions with significant variations of the meiotic recombination rate in the cross considered. The recombination landscape in maize shows strong suppression of recombination in the centromeric region [32]. With the high density genetic maps generated here with the array, it is now possible to delimit regions with low or high recombination to precise physical regions and, for example, to correlate to the distribution of crossovers obtained from cytological approaches [33].

In addition to its relevance for genetic mapping and genetic diversity analysis, the developed array will also be useful in other applications. Previously, the analysis of marker/trait associations in maize panels was primarily limited to the analysis of candidate genes (*e.g.* from specific biosynthetic pathways). With the array and its high marker density, it will be possible to perform such association studies at a genome wide level (GWAS). Currently, the genetic materials for which the array will be most useful in GWAS experiments remains to be determined. In highly diverse maize material, the number of markers and their selection will not be sufficient to find associations due to a very low level of linkage disequilibrium. In this case, other methods are needed, that analyze larger numbers of markers through larger arrays, genotyping by sequencing, or whole genome sequencing. On the other hand, it is known that specific groups of commercial maize breeding material display large segments of high linkage disequilibrium that frequently extends over thousands or millions of base pairs [34]–[36]. Then, it is very likely that the array can be applied for association studies in at least some groups of commercial maize breeding material showing high LD [37], [38]. In such material, the array will likely be most useful for the genetic improvement of maize lines through genomic selection, just as it has been demonstrated with a large genotyping array for cattle and in first experiments also for maize [26]. Thus it seems quite inevitable that the array will also open the door to a number of novel applications in maize breeding.

## Materials and Methods

Details on the Material and Methods for the marker selection and genetic mapping procedures are provided in Text S1.

### Maize material

For the initial characterization of the MaizeSNP50 array, a total of 274 maize lines were genotyped. These lines included sequenced reference lines (*e.g.* B73 and Mo17), duplicated DNA samples and duplicated samples from different origins, parent/F1 combinations, 25 NAM parents [32], important inbred lines from North America and Europe, teosinte inbred lines and other samples. This material represented samples from most of the crossing range of maize [34] (Table S12). For the genetic mapping, 239 lines from the IBM population (B73×Mo17) and 226 individuals from the LHRF population (F2×F252) were genotyped.

### Selection of SNP markers for the array

A total of 839,350 SNP markers were used as starting material for the design of the array. This library of markers was derived from five classes. (1) The majority of these markers (78,9051) were a subset of the 2,000,000 SNPs from the first generation haplotype map [9]. The selected SNPs (PZ) showed allele frequencies greater than 0.2 in the 25 NAM parents and this set was termed the Panzea set. (2) 40,000 markers provided by Syngenta (Research Triangle, North Carolina) were high confidence SNPs (SYN or SYNGENTA) arising between B73 and Mo17 with known minor allele frequencies ranging from 0.1 to 0.50 (mean = 0.28). The allele frequencies were derived from transcriptome sequencing of maize inbreds at the National Center of Genome Resources (NCGR) representing significant genetic variation through the incorporation of: 1) elite inbred lines (defined as commercially relevant) from the US composed primarily from stiff stalk and non-stiff stalk heterotic groups, as well as other heterotic groups, 2) historical founder lines and non-elite inbred lines from the US and 3) diverse inbred lines from non-US sources. (3) Another 4,907 SNPs were provided by INRA (PUT) and they resulted from comparative sequencing of B73 and F2 ESTs. (4) 3,996 SNPs were derived from comparative Sanger sequencing of a diverse maize panel of 14 lines at TraitGenetics (ZM) that contained some key inbreds for European and North American maize breeding. (5) The remaining 1,396 SNPs (all other designations) were collected from various other published marker sets [39].

The SNP selection process on these 814,863 SNPs started with the elimination of duplicated SNPs. To satisfy Illumina Infinium assay design quality requirements, assay design scores were generated for the remaining SNPs and further SNPs were eliminated because they contained nearby known SNPs in both flanking sequences. These two selection steps together reduced the size of the SNP pool to a total of 216,723 candidate SNPs having high design scores.

Technically, the final SNP selection procedure for the array was performed in four steps. Step 1 was the selection of SNPs provided by all other sources with the exception of the Panzea set and that had matches to the high-confidence filtered genes described by Schnable et al. [2]. In Step 2, SNPs from all other sources except for the Panzea set were selected that had no matches to filtered genes. This was done to increase coverage for diverse lines as all these SNPs were derived from transcribed sequences and the B73 line does not represent the entire *Zea mays* gene set [2], [9]. Step 3 included the selection of Panzea SNPs that had matches to the filtered genes that had not been covered in Steps 1 and 2 in order to represent as many genes as possible. The last selection step (Step 4) was based on the available genomic maize sequence with the goal to optimize coverage and even spacing throughout the genome notably regions insufficiently covered in the gene-based selection steps described above.

In case multiple SNPs met the selection criteria for genes/regions, Infinium II assays (one bead type per assay) were preferred over Infinium I assays (two bead types per assay), and higher assay design scores were preferred over lower ones. Altogether this resulted in 57,838 SNP markers that were synthesized. All these SNPs have been deposited in dbSNP. Their NCBI assay IDs (ss#) are given in Table S13.

### SNP marker analysis and development of the cluster file

The maize array was used to genotype all maize lines and the mapping populations described in the Maize Materials section. Illumina manufacturing processes led us to eliminate 1,728 markers that failed to meet bead representation and decoding quality metrics. The remaining set of 56,110 markers was analyzed with respect to the clustering of the genotypes using GenomeStudio Genotyping software (v2009, Illumina, Inc). In this step, the quality of each marker was assessed by visual inspection of the cluster distribution and by subsequent adjustment of the cluster calling for each marker so that three clearly identifiable and scorable clusters were generated. The cluster definition was performed with the genotype data from all 274 maize lines and was mainly based on the correct calling of the markers in the parent/F1 combinations in the panel (B73/Mo17 and its F1, B73×NAM parents and its F1, Triplets with their two parents and F1). This clustering analysis resulted in the MaizeSNP50_B.egt cluster file (http://www.illumina.com/support/downloads.ilmn) with 56,110 markers of which 49,585 markers were considered as robust and could be scored in the 274 lines.

### Building linkage maps for the IBM and LHRF populations

The algorithms and parameters used to compute the IBM and LHRF genetic maps were strictly identical and produced *de novo* maps without using the B73 genome sequence information. The software CarthaGene [40] was used for the different steps of map construction using R scripts. For the map constructions, only SNPs homozygous and polymorphic in the pair of founding parents of the IRILs were considered. When a genotype was heterozygous, it was replaced by a missing data point. The data were filtered on their quality by the GenCall score (GC score, produced by the GenomeStudio Genotyping software). A GC score threshold of 0.8 was used for the framework maps and 0.6 for the placement of additional markers. Genotype data points below this GC score threshold were turned into missing data points. SNPs were considered for mapping if they had less than 35% of missing data for the framework maps and 50% for the complete map. Furthermore, only markers with a minor allele frequency greater than 0.10 were considered.

In the first step of map construction, a seed marker was used to aggregate further markers into a highly accurate scaffold map of a chromosome in which markers were separated by at least 10 cM. A marker was added to the scaffold if its placement score was higher than a threshold and the order of the map was then recalculated. The process was iterated until no more markers could be added. Several replicates of the scaffolds were produced using different seed markers. The second step consisted in increasing the density of markers of these scaffold maps to build framework maps. All markers were assigned to a linkage group based on the scaffold and then each candidate marker was tentatively inserted into the map while robustness of the whole map order after insertion was controlled. The addition of markers to the scaffold was finished when all the markers had been examined, resulting in a framework map, in which gaps had been reduced and marker order was still statistically highly robust.

Using the framework maps, complete maps were obtained by individual placements of markers, referred to as bin-mapping [16]. The associated placed markers then had positions that were statistically less strongly supported than those of the framework map. The estimation of the genetic distances in IRILs was performed using a specific method to compute real centiMorgan genetic distances by correcting for the higher amount of recombination occurring during the intermating generations [15], [41].

## Supporting Information

Figure S1 **Dendrogram of the investigated maize lines.** Dendrogram for the 274 maize lines based on the marker data from the array for only the PZ (Panzea) markers. Method of analysis: NTSYS Similarity of qualitative data (DICE coefficient).
(PDF)

Figure S2 **Allele frequency distribution for all polymorphic markers in the two mapping populations.** Allele frequencies of the parent B73 in the IBM population (lower part, red dots), and of the parent F2 in the LHRF population (upper part, blue dots) for all SNPs mapped and all chromosomes. Lines represent 1% confidence intervals of the expected 0.5 value under Mendelian segregation.
(PDF)

Figure S3 **Distribution of SNP markers polymorphic on the IBM and LHRF mapping populations.** Top: IBM mapping population; Bottom: LHRF mapping population. Bin size is 5 Mbp along the physical coordinates of the B73 sequence.
(PDF)

Figure S4 **Whole-chromosome comparison between the** ***framework*** **genetic maps IBM and LHRF and the B73 genome coordinates for entire chromosomes.** In the ladder diagrams of the two left panels, the position of a marker corresponds to its index in the ordered map and not to its genetic position. Numbers in parentheses indicate the map coordinate in cM for IBM or LHRF genetic maps and in Mbp for the B73 genome sequence. In the right panel, positions of the markers are proportional to the cM or Mb map coordinate. The ladders have their scales adjusted to fit the two maps to the same height. In the right panel, genetic maps are scaled to the physical map length. Blue rectangles indicate marker intervals containing the centromere, according to MaizeGDB.
(PDF)

Figure S5 **Whole-chromosome comparison of the** ***complete*** **genetic maps for the IBM and LHRF populations in relation to the B73 genome for entire chromosomes.** The *complete* genetic maps contain both framework and placed markers. In the ladder diagrams of the two left panels, positions of the markers correspond to their index in the ordered maps and not to their genetic position. Numbers in parentheses indicate the map coordinate in cM for IBM or LHRF genetic maps and in Mbp for the B73 genome sequence. In the right panel, positions of the markers are proportional to the cM or Mb map coordinate. The ladders have their scales adjusted to fit the two maps to the same height. In the right panel, genetic maps are scaled to the physical map length. Blue rectangles indicate marker intervals containing the centromere, according to MaizeGDB.
(PDF)

Figure S6 **Comparison between the** ***complete*** **genetic maps IBM and LHRF, and the B73 genome for nine regions for which either genetic map contains markers non-colinear with the B73 genome.** The *complete* genetic maps containing both framework and placed markers are displayed for the nine non-colinear regions defined in Figure 6 and Table S11. In the ladder diagrams of the upper panels, positions of the markers correspond to their index in the ordered maps and not to their genetic position. Numbers in parentheses indicate the map coordinate in cM for IBM or LHRF genetic maps and in Mbp for the B73 genome sequence. In the lower panel, positions are proportional to the physical map coordinates. Scales were adjusted to fit the two maps to the same height.
(PDF)

Table S1 **Duplicate reproducibility and parent/hybrid heritability.** Top part shows duplicate reproducibility for DNA and sample duplicates. Bottom part shows correctness of analyzed triplets (2 parents and F1).
(XLS)

Table S2 **Quality data for the 49,585 scorable markers for the 274 maize lines based on MaizeSNP50_B.egt cluster file.**
(XLS)

Table S3 **Genotype data of the 274 maize lines and hybrids used for the establishment of the cluster file.** This ZIP compressed comma-separated text file contains the genotyping data for all 49,585 SNP markers on the 274 maize lines. In addition to the marker name, the source of the marker and the dbSNP number are displayed. For each maize line, its name, classification, and the actual genotyping data (in base calls according to IUPAC) are shown in a column. Failed = no genotype data.
(CSV)

Table S4 **Polymorphism matrix for main groups of the investigated maize lines.** Groups can be found on different sheets. Lines are indicated as described in Table S12.
(XLS)

Table S5 **Number of SNPs in individual maize genes.** Genes are listed according to their accession number.
(XLS)

Table S6 **Markers and gene assignment.** For each marker, its assignment to filtered genes is displayed. NULL = no assignment.
(XLS)

Table S7 **List of all markers of the IBM and LHRF** ***framework*** **maps (with statistically supported order).** Genetic coordinate are cM for genetic positions obtained by taking into account the intermating during population development. *Genetic coordinate (pseudo cM) are (overestimated) cM for genetic positions obtained by computing the distances as if the plants were RILs instead of Intermated RILs.
(XLS)

Table S8 **List of all markers of the IBM and LHRF** ***complete*** **maps including the framework maps and all markers placed on the frameworks.** “IBM status” and “LHRF status” indicate if the SNP was included in the framework map (“frame”), or placed onto the framework (“placed”), or not mapped (“−”). “IBM chrom” and “LHRF chrom” indicate the chromosome assignment obtained by genetic mapping and “B73 genome chrom” the coordinates on the B73 physical map. “IBM coordinate (cM)” and “LHRF coordinate (cM)” indicate centiMorgan coordinates of the SNPs on the genetic maps for genetic positions obtained by taking into account the intermating during population development. *IBM and LHRF coordinate (pseudo cM) are (overestimated) cM for genetic positions obtained by computing the distances as if the plants were RILs instead of Intermated RILs.
(XLS)

Table S9 **Position of mapped SNP markers not located on B73 genome sequence.** The genetic mapping results are displayed for the 172 markers that were not found on the B73 genome sequence. Table columns are as in Table S8.
(XLS)

Table S10 **Position of markers for which the chromosomal assignments on the genetic map and on the B73 sequence do not agree.** B73 genome chrom: chromosomal assignment based on the B73 genome sequence; Genetic chrom: chromosomal assignment based on the genetic mapping in the mapping population. Predicted coordinate on B73 genome: homothetically estimated physical positions based on the genetic mapping results.
(XLS)

Table S11 **List of major non-colinear regions between genetic and physical maps.** The names of the regions are the same as in Figure 6.
(XLS)

Table S12 **List of 274 investigated maize lines with assignment of the respective class.** Sheet 1: List of lines; Sheet 2: Lines sorted according to groups defined in Figure 4.
(XLS)

Table S13 **List of the 57,838 SNP markers that were put on the array, with their NCBI assay ID (ss#) in dbSNP.**
(XLS)

Text S1 **Detailed methods.** More detailed description of procedures and parameters used for the development of the SNP array and the construction of the IBM and LHRF genetic maps.
(PDF)

## References

[1] Doebley JF, Gaut BS, Smith BD (2006). The Molecular Genetics of Crop Domestication.. Cell.

[2] Schnable PS, Ware D, Fulton RS, Stein JC, Wei F (2009). The B73 Maize Genome: Complexity, Diversity, and Dynamics.. Science.

[3] Zhou S, Wei F, Nguyen J, Bechner M, Potamousis K (2009). A Single Molecule Scaffold for the Maize Genome.. PLoS Genet.

[4] Wei F, Stein JC, Liang C, Zhang J, Fulton RS (2009). Detailed Analysis of a Contiguous 22-Mb Region of the Maize Genome.. PLoS Genet.

[5] Wei F, Zhang J, Zhou S, He R, Schaeffer M (2009). The Physical and Genetic Framework of the Maize B73 Genome.. PLoS Genet.

[6] Messing J, Dooner HK (2006). Organization and variability of the maize genome.. Current Opinion in Plant Biology.

[7] Bennetzen JL (2007). Patterns in grass genome evolution.. Current Opinion in Plant Biology.

[8] Lai J, Li R, Xu X, Jin W, Xu M (2010). Genome-wide patterns of genetic variation among elite maize inbred lines.. Nat Genet.

[9] Gore MA, Chia J-M, Elshire RJ, Sun Q, Ersoz ES (2009). A First-Generation Haplotype Map of Maize.. Science.

[10] Springer NM, Ying K, Fu Y, Ji T, Yeh C-T (2009). Maize Inbreds Exhibit High Levels of Copy Number Variation (CNV) and Presence/Absence Variation (PAV) in Genome Content.. PLoS Genet.

[11] Beavis WD, Grant D (1991). A linkage map based on information from four F2 populations of maize (Zea mays L.). Theoret. Appl.. Genetics.

[12] Burr B, Burr FA, Thompson KH, Albertson MC, Stuber CW (1988). Gene Mapping with Recombinant Inbreds in Maize.. Genetics.

[13] Beavis W, Lee M, Grant D, Hallauer A, Owens T (1992). The influence of random mating on recombination among RFLP loci.. Maize Newsl.

[14] Sharopova N, McMullen MD, Schultz L, Schroeder S, Sanchez-Villeda H (2002). Development and mapping of SSR markers for maize.. Plant Molecular Biology.

[15] Winkler CR, Jensen NM, Cooper M, Podlich DW, Smith OS (2003). On the Determination of Recombination Rates in Intermated Recombinant Inbred Populations.. Genetics.

[16] Falque M, Decousset L, Dervins D, Jacob A-M, Joets J (2005). Linkage Mapping of 1454 New Maize Candidate Gene Loci.. Genetics.

[17] Yu J, Holland JB, McMullen MD, Buckler ES (2008). Genetic Design and Statistical Power of Nested Association Mapping in Maize.. Genetics.

[18] Steemers FJ, Chang W, Lee G, Barker DL, Shen R (2006). Whole-genome genotyping with the single-base extension assay.. Nat Meth.

[19] Gunderson KL, Steemers FJ, Ren H, Ng P, Zhou L (2006). Whole-Genome Genotyping.. DNA Microarrays, Part A: Array Platforms and Wet-Bench Protocols.

[20] Elshire RJ, Glaubitz JC, Sun Q, Poland JA, Kawamoto K (2011). A Robust, Simple Genotyping-by-Sequencing (GBS) Approach for High Diversity Species.. PLoS ONE.

[21] McCarthy MI, Abecasis GR, Cardon LR, Goldstein DB, Little J (2008). Genome-wide association studies for complex traits: consensus, uncertainty and challenges.. Nat Rev Genet.

[22] Matukumalli LK, Lawley CT, Schnabel RD, Taylor JF, Allan MF (2009). Development and Characterization of a High Density SNP Genotyping Assay for Cattle.. PLoS ONE.

[23] Ramos AM, Crooijmans RPMA, Affara NA, Amaral AJ, Archibald AL (2009). Design of a High Density SNP Genotyping Assay in the Pig Using SNPs Identified and Characterized by Next Generation Sequencing Technology.. PLoS ONE.

[24] Meuwissen THE, Hayes BJ, Goddard ME (2001). Prediction of Total Genetic Value Using Genome-Wide Dense Marker Maps.. Genetics.

[25] Heffner EL, Sorrells ME, Jannink J-L (2009). Genomic Selection for Crop Improvement.. Crop Science.

[26] Albrecht T, Wimmer V, Auinger H-J, Erbe M, Knaak C (2011). Genome-based prediction of testcross values in maize.. Theor Appl Genet.

[27] Durstewitz G, Polley A, Plieske J, Luerssen H, Graner EM (2010). SNP discovery by amplicon sequencing and multiplex SNP genotyping in the allopolyploid species Brassica napus.. Genome.

[28] Nelson PT, Coles ND, Holland JB, Bubeck DM, Smith S (2008). Molecular Characterization of Maize Inbreds with Expired U.S. Plant Variety Protection.. Crop Science.

[29] Remington DL, Thornsberry JM, Matsuoka Y, Wilson LM, Whitt SR (2001). Structure of linkage disequilibrium and phenotypic associations in the maize genome.. Proceedings of the National Academy of Sciences.

[30] Li WH, Sadler LA (1991). Low Nucleotide Diversity in Man.. Genetics.

[31] Zhao Z, Yu N, Fu Y-X, Li W-H (2006). Nucleotide Variation and Haplotype Diversity in a 10-kb Noncoding Region in Three Continental Human Populations.. Genetics.

[32] McMullen MD, Kresovich S, Villeda HS, Bradbury P, Li H (2009). Genetic Properties of the Maize Nested Association Mapping Population.. Science.

[33] Anderson LK, Doyle GG, Brigham B, Carter J, Hooker KD (2003). High-Resolution Crossover Maps for Each Bivalent of Zea mays Using Recombination Nodules.. Genetics.

[34] Yan J, Shah T, Warburton ML, Buckler ES, McMullen MD (2009). Genetic Characterization and Linkage Disequilibrium Estimation of a Global Maize Collection Using SNP Markers.. PLoS ONE.

[35] Ching A, Caldwell K, Jung M, Dolan M, Smith O (2002). SNP frequency, haplotype structure and linkage disequilibrium in elite maize inbred lines.. BMC Genetics.

[36] Jung M, Ching A, Bhattramakki D, Dolan M, Tingey S (2004). Linkage disequilibrium and sequence diversity in a 500-kbp region around the adh1 locus in elite maize germplasm.. Theor Appl Genet.

[37] Beló A, Zheng P, Luck S, Shen B, Meyer DJ (2007). Whole genome scan detects an allelic variant of fad2 associated with increased oleic acid levels in maize.. Mol Genet Genomics.

[38] Inghelandt D, Reif JC, Dhillon BS, Flament P, Melchinger AE (2011). Extent and genome-wide distribution of linkage disequilibrium in commercial maize germplasm.. Theor Appl Genet.

[39] Jones E, Chu W-C, Ayele M, Ho J, Bruggeman E (2009). Development of single nucleotide polymorphism (SNP) markers for use in commercial maize (Zea mays L.) germplasm.. Mol Breeding.

[40] de Givry S, Bouchez M, Chabrier P, Milan D, Schiex T (2004). CarthaGene: multipopulation integrated genetic and radiation hybrid mapping.. Bioinformatics.

[41] Falque M (2005). IRILmap: linkage map distance correction for intermated recombinant inbred lines/advanced recombinant inbred strains.. Bioinformatics.

