# Supplementary figures and images for: A Large Maize (Zea mays L.) SNP Genotyping Array: Development and Germplasm Genotyping, and Genetic Mapping to Compare with the B73 Reference Genome
Source: PLoS One. 2011 Dec 8;6(12):e28334. doi: 10.1371/journal.pone.0028334 (PMC3234264; doi:10.1371/journal.pone.0028334)

# PZE markers

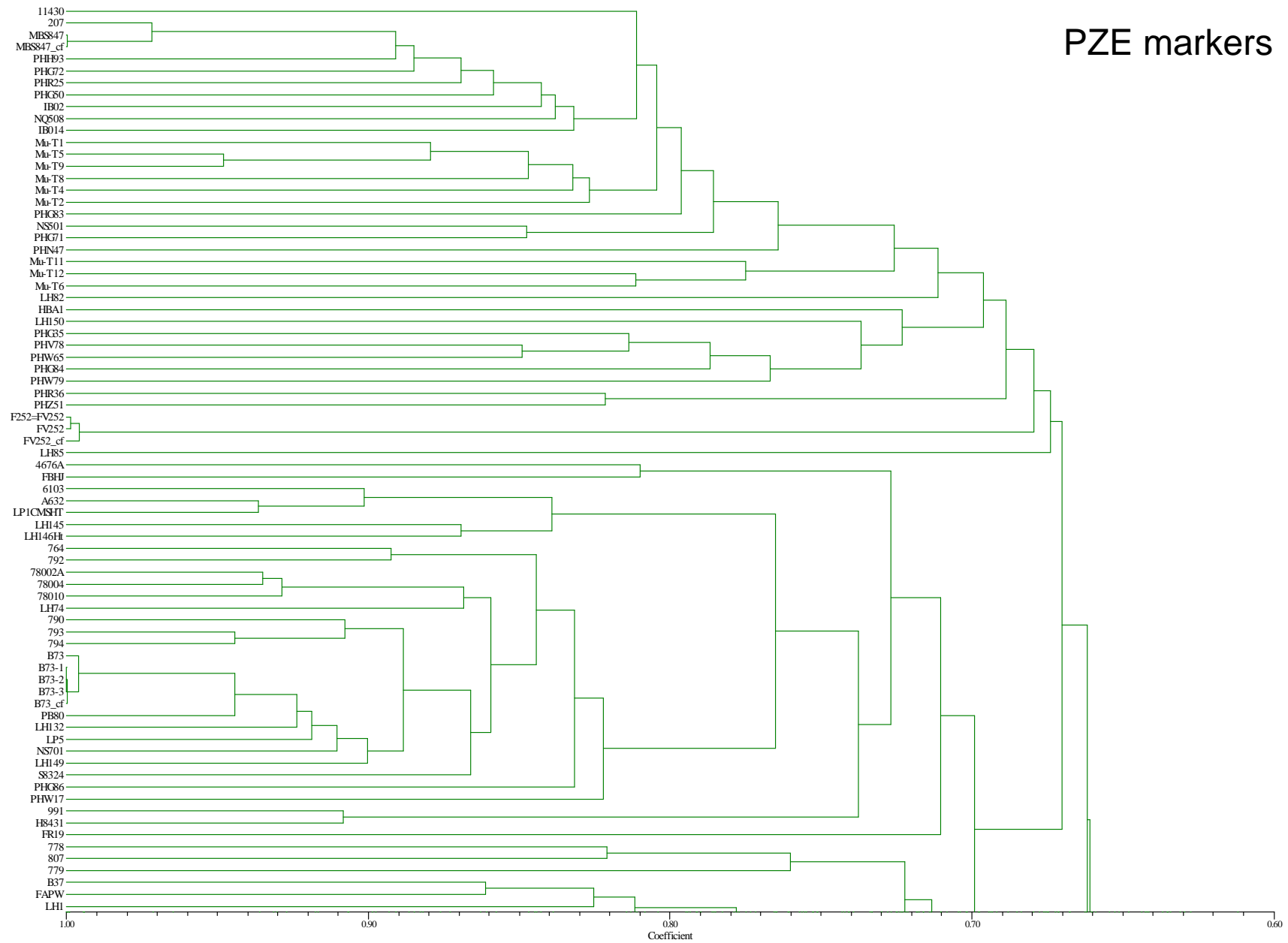

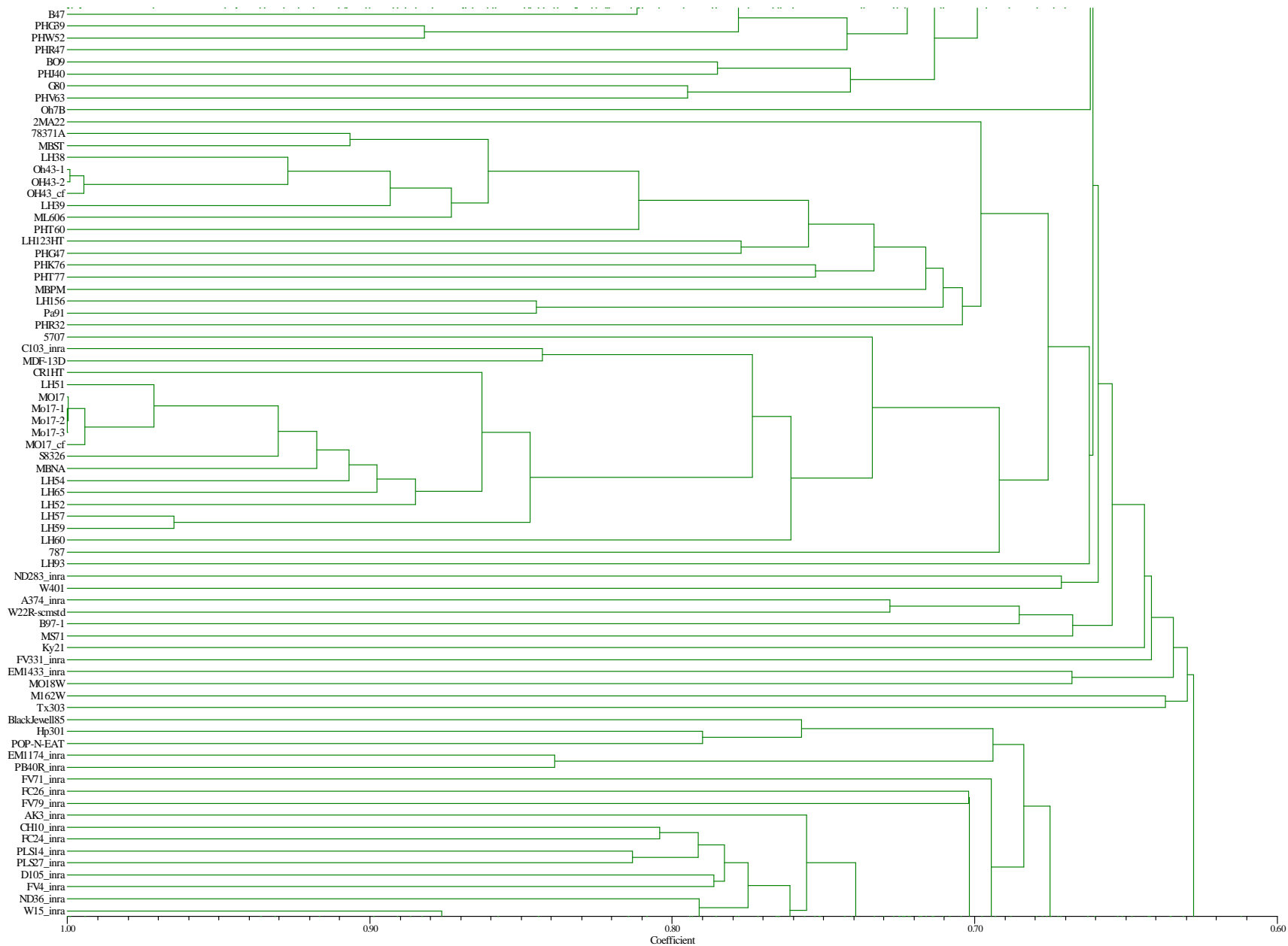

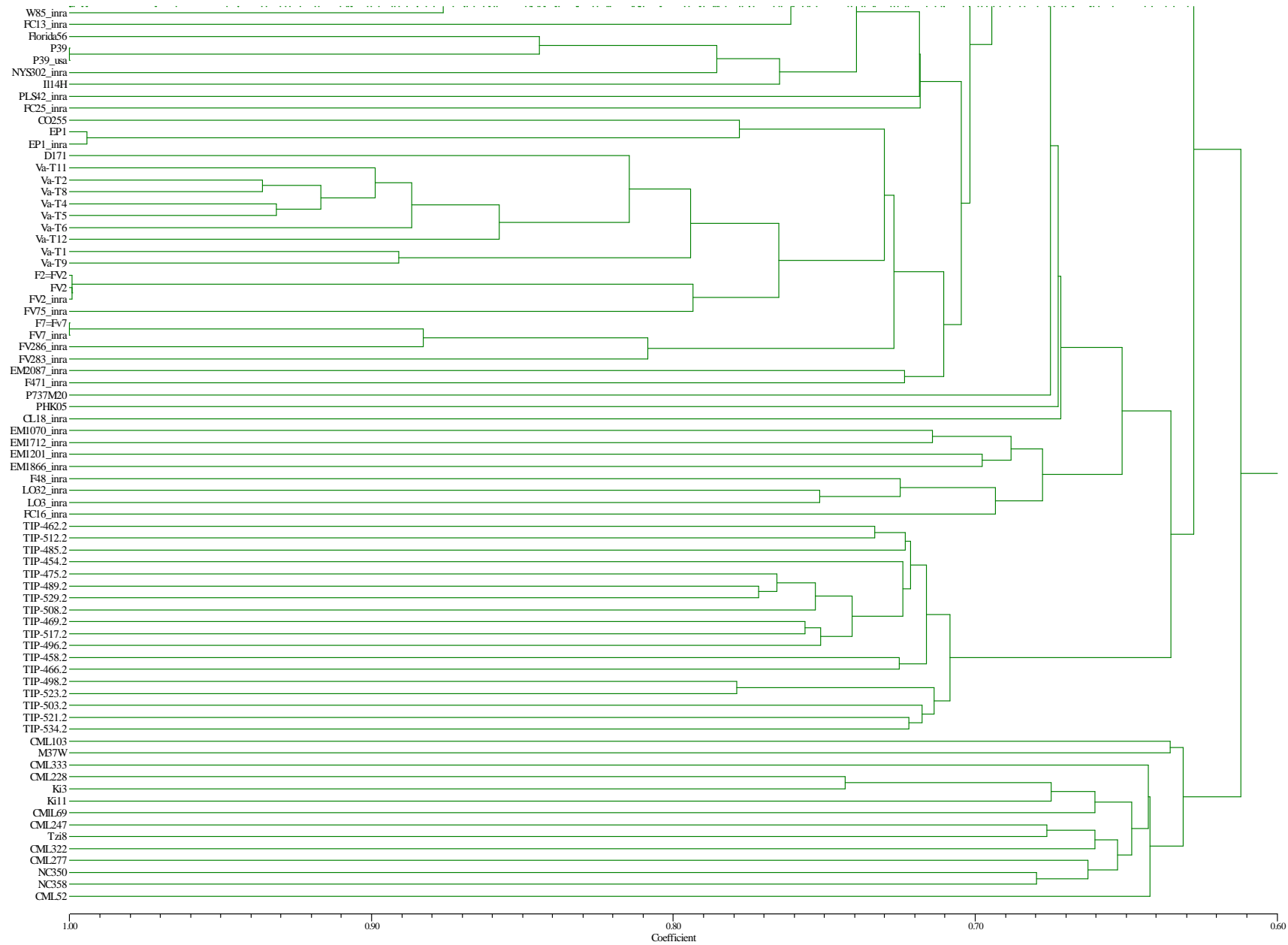

Supplement: Figure S1 — Dendrogram of the investigated maize lines. Dendrogram for the 274 maize lines based on the marker data from the array for only the PZ (Panzea) markers. Method of analysis: NTSYS Similarity of qualitative data (DICE coefficient). (PDF) [file pone.0028334.s001.pdf]

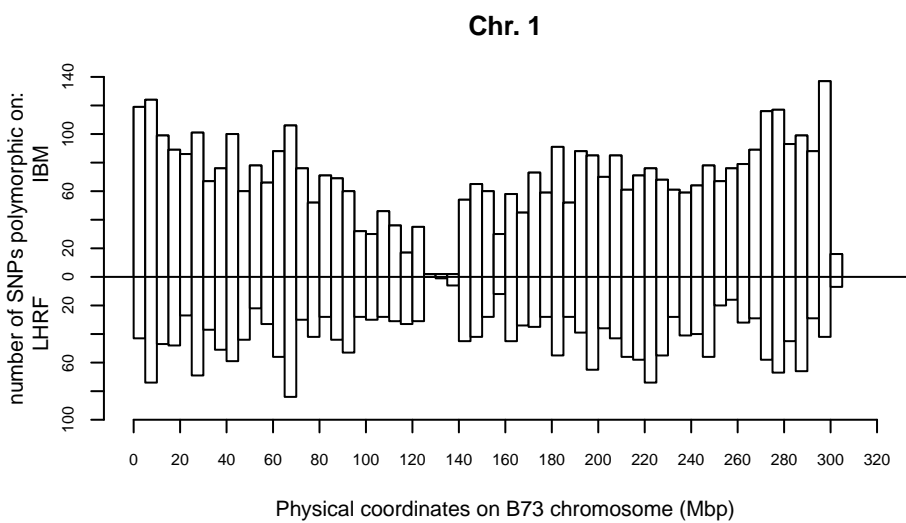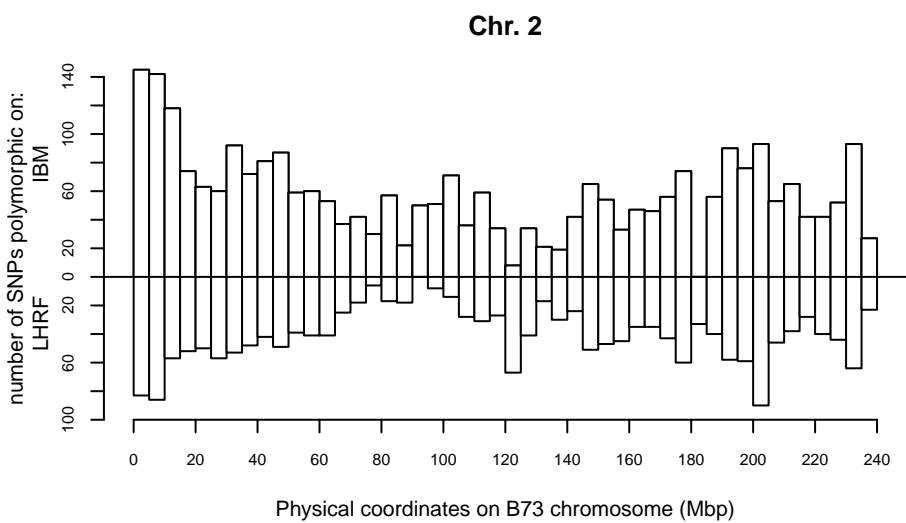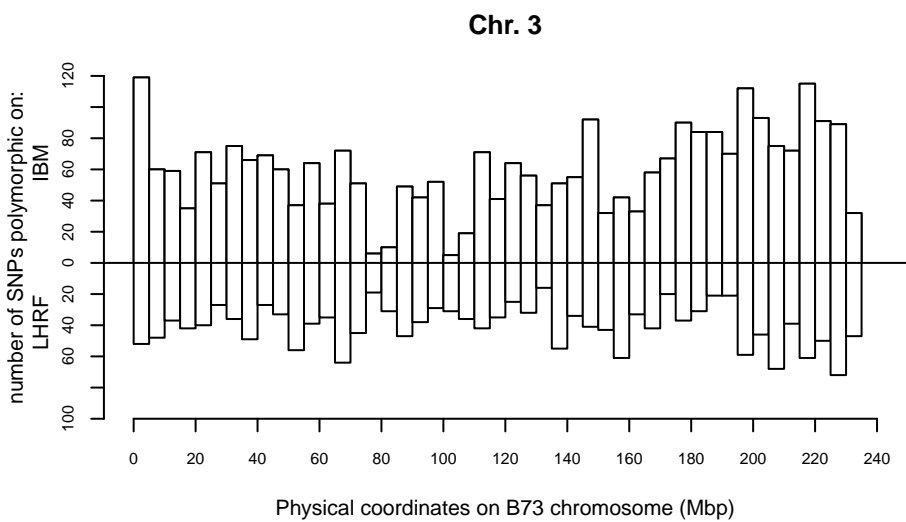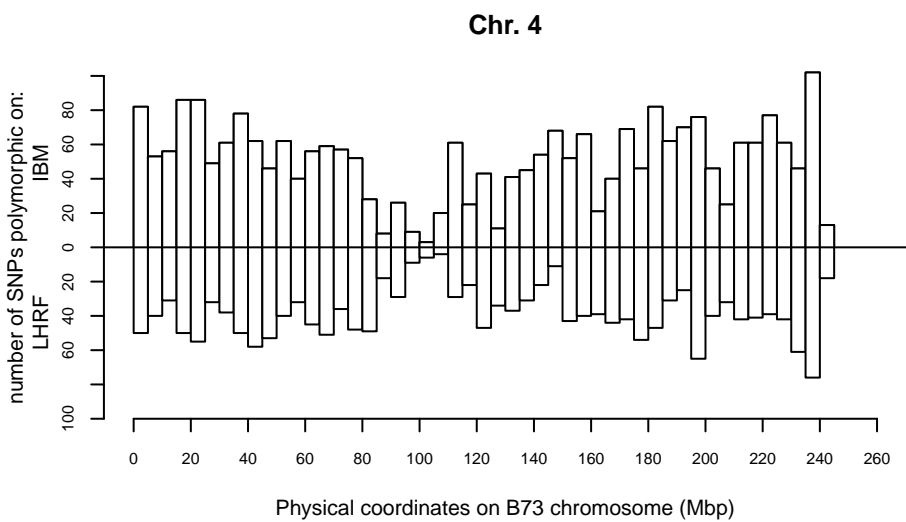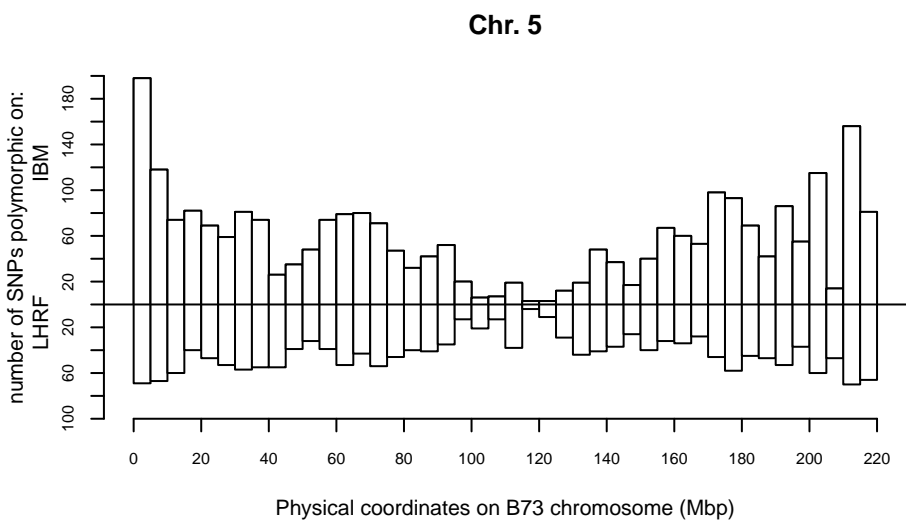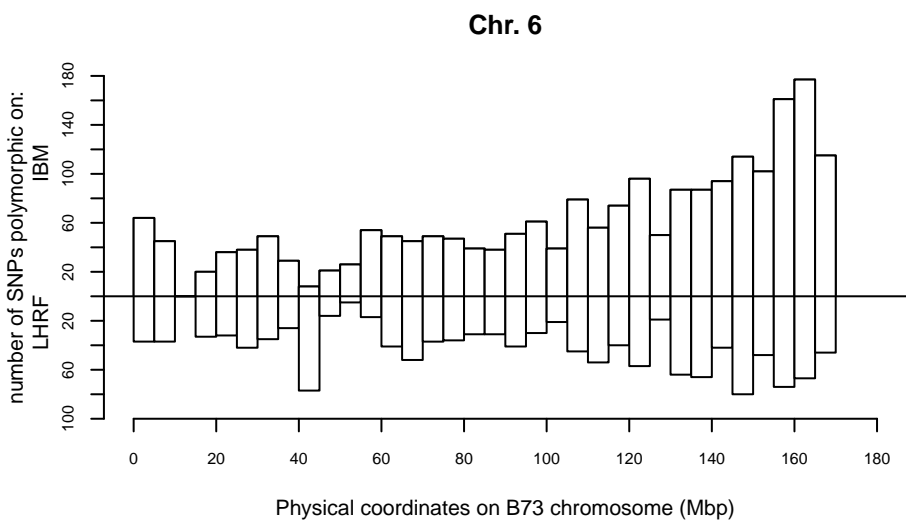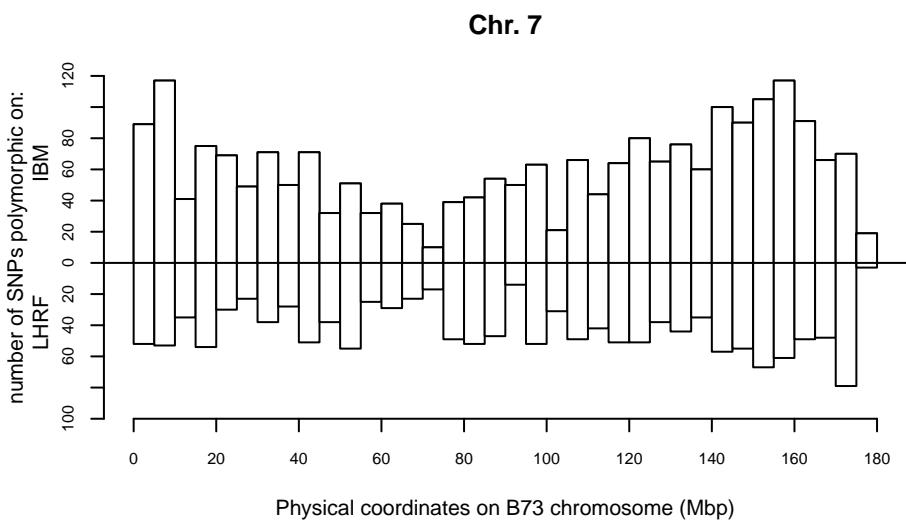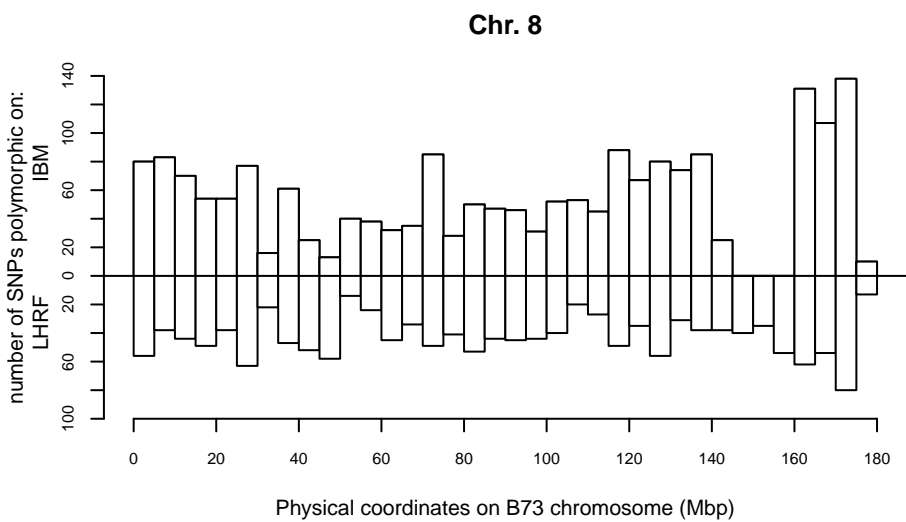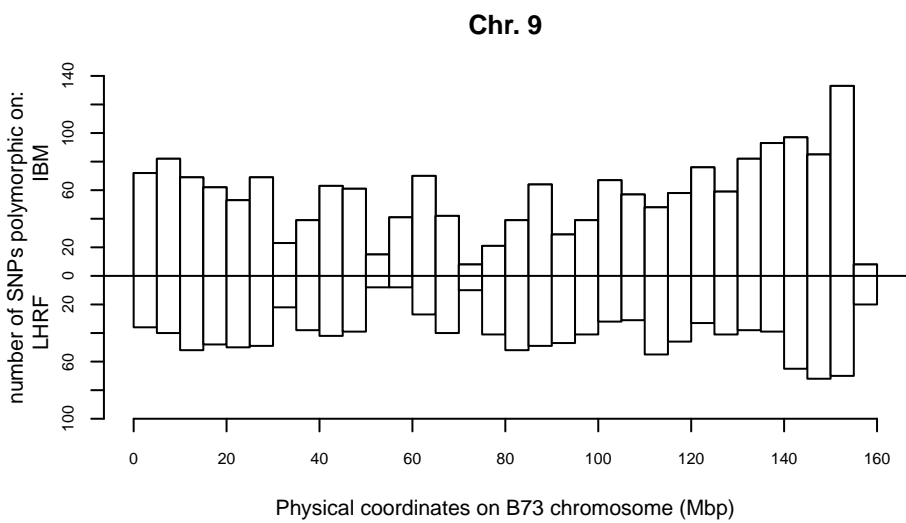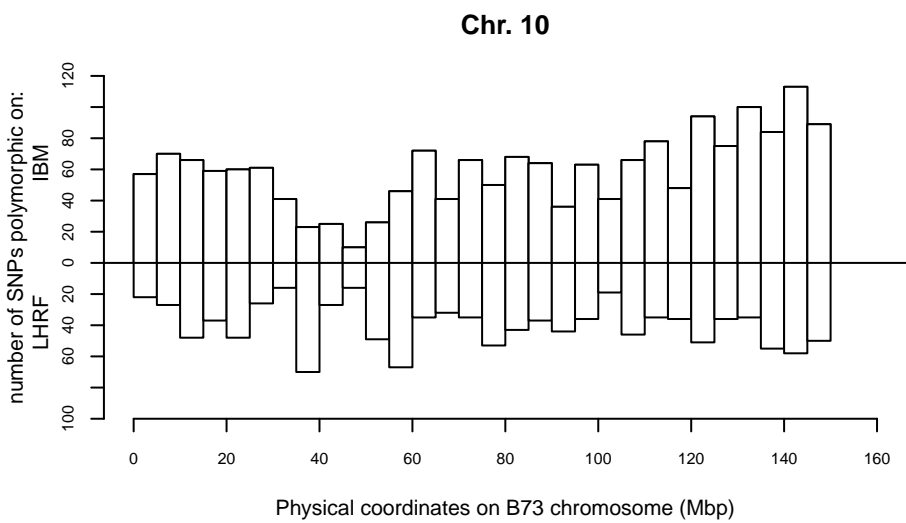

Supplement: Figure S3 — Distribution of SNP markers polymorphic on the IBM and LHRF mapping populations. Top: IBM mapping population; Bottom: LHRF mapping population. Bin size is 5 Mbp along the physical coordinates of the B73 sequence. (PDF) [file pone.0028334.s003.pdf]

[illegible]

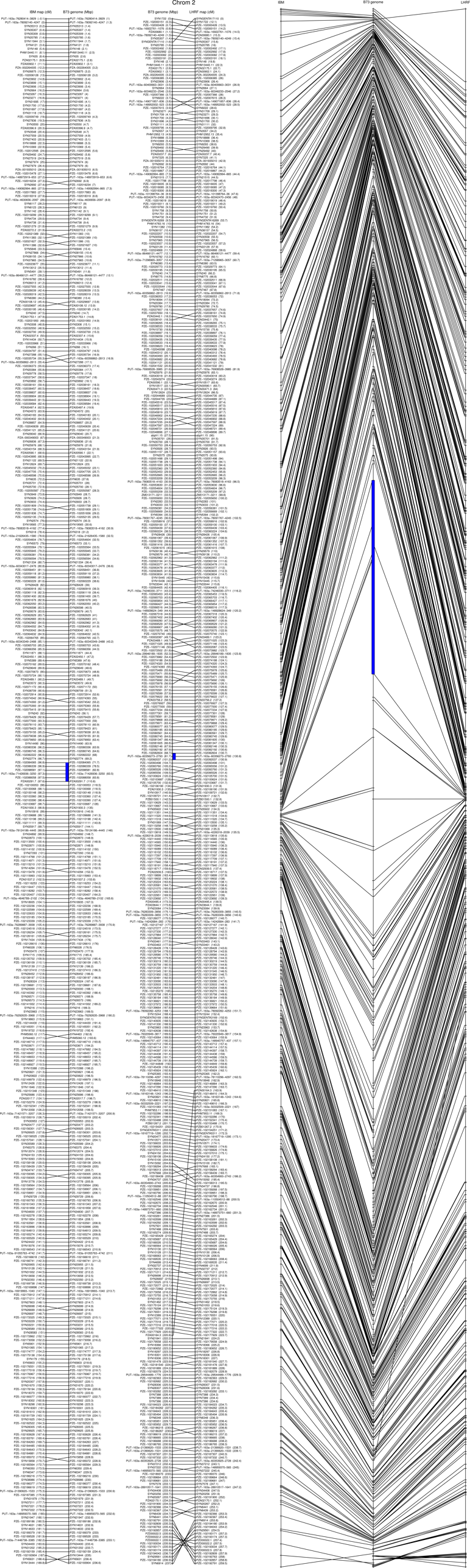

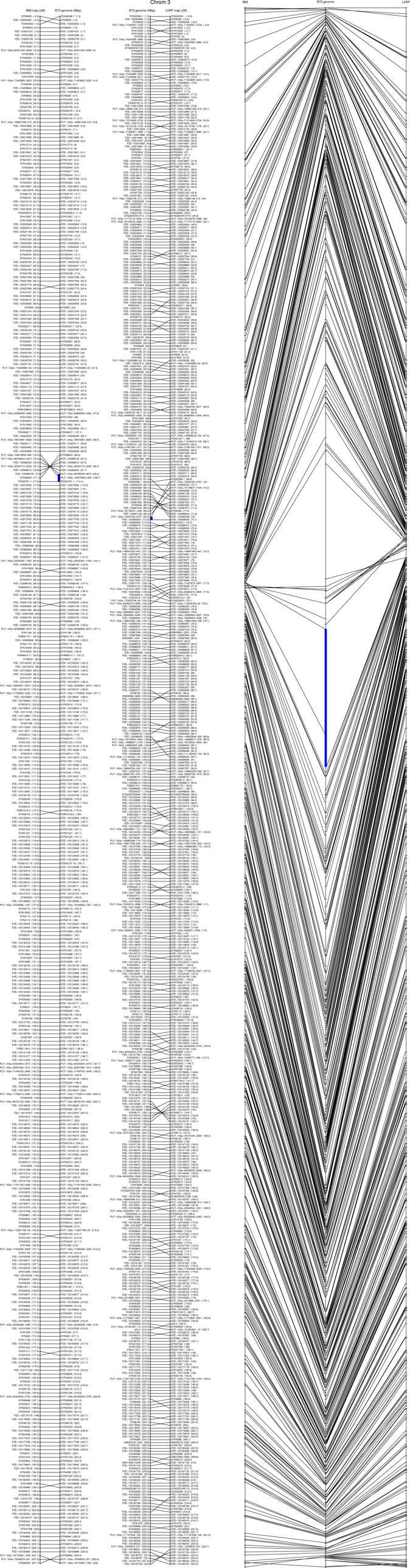

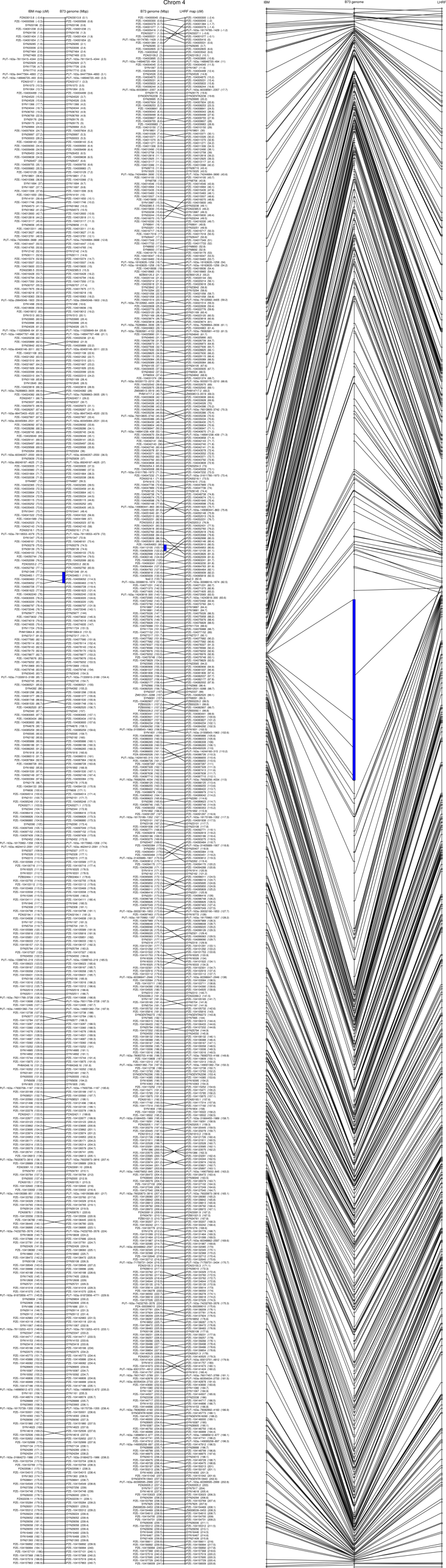

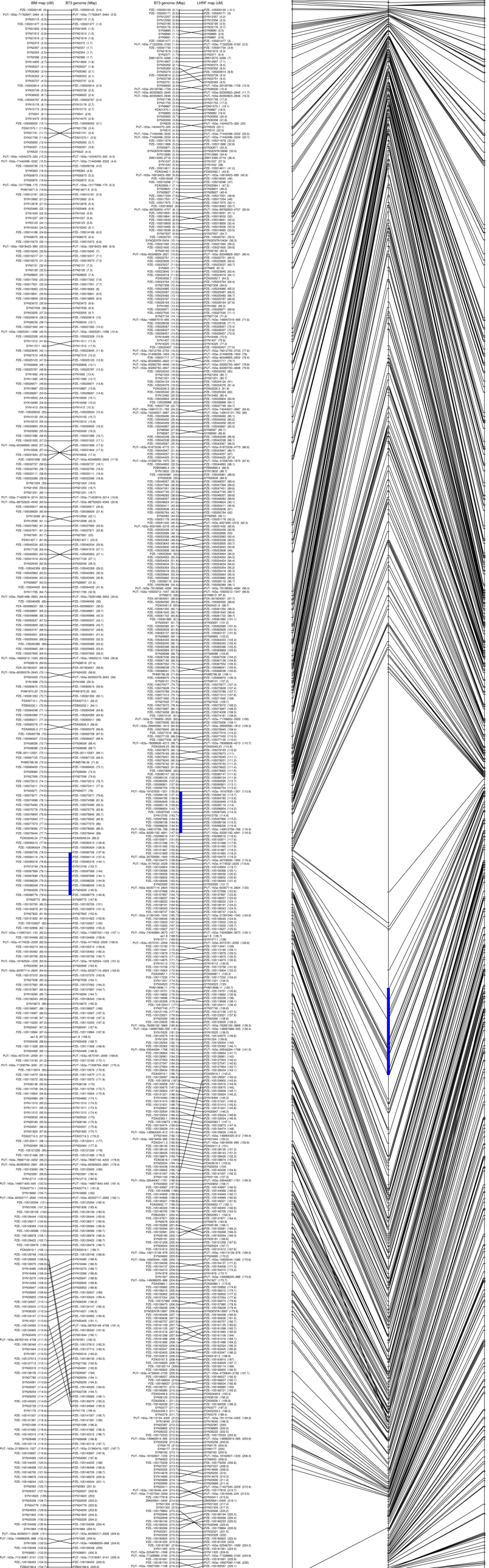

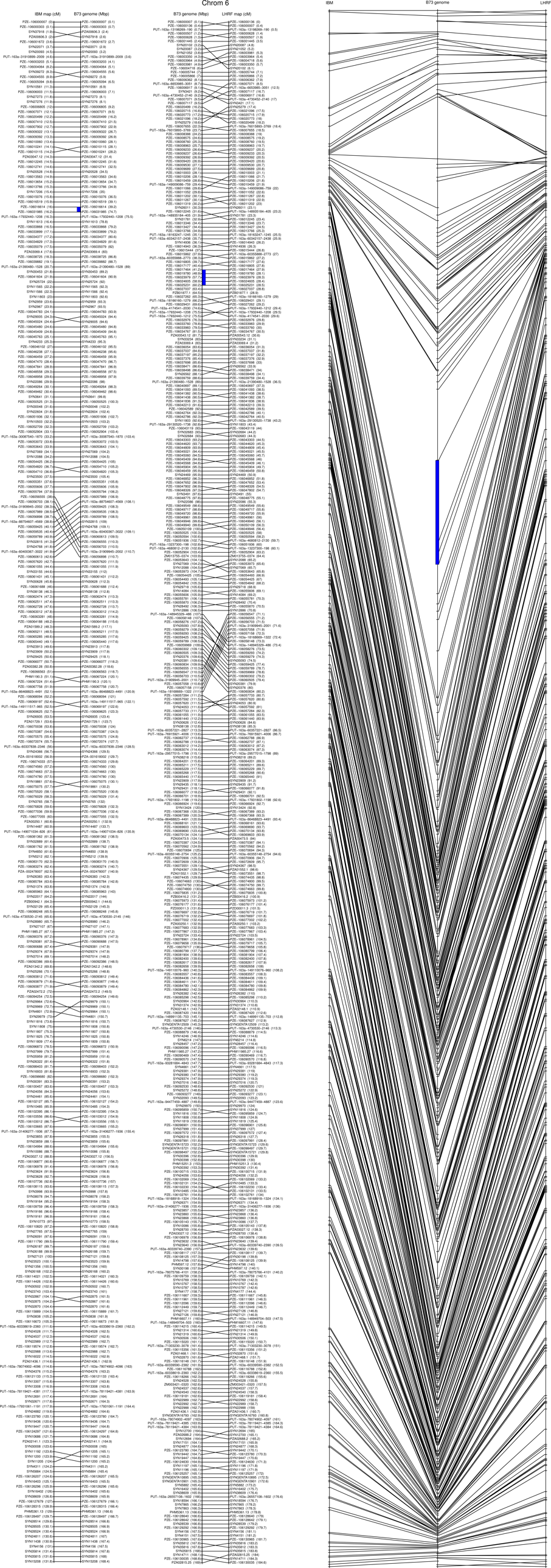

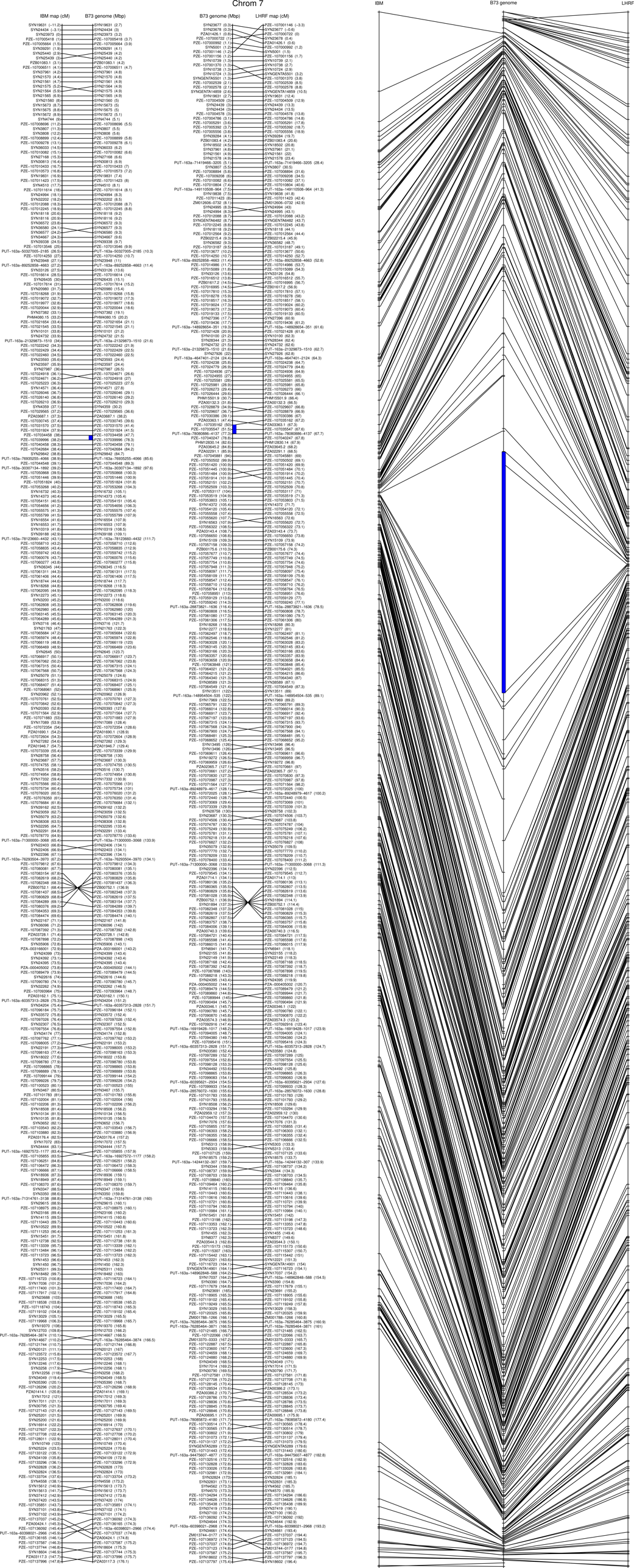

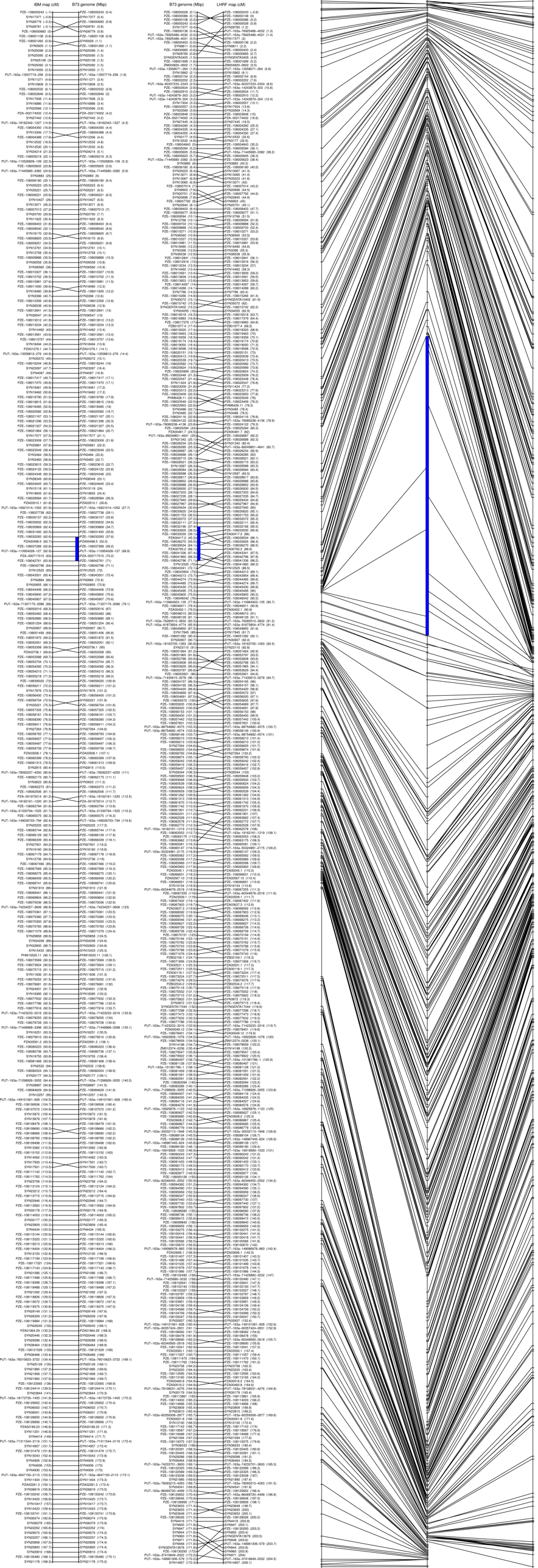

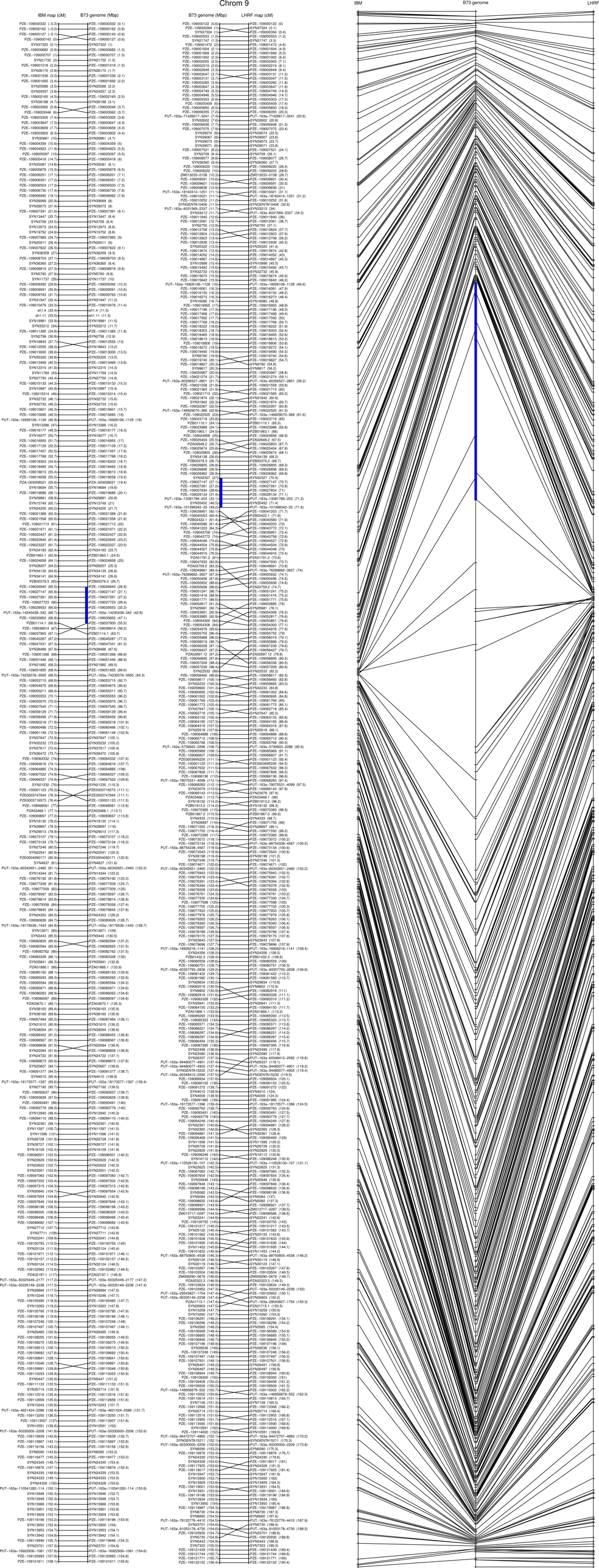

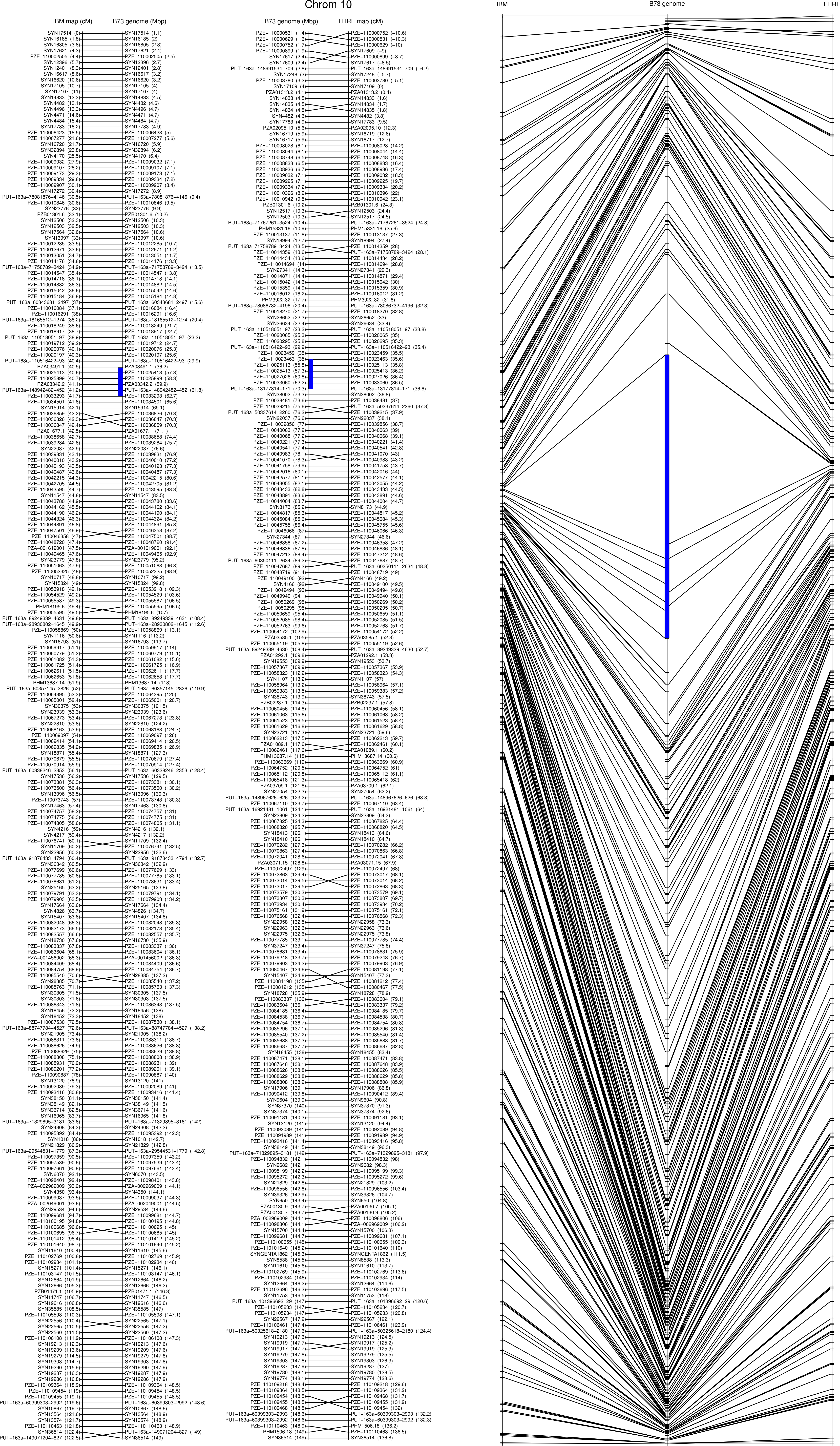

Supplement: Figure S5 — Whole-chromosome comparison of the complete genetic maps for the IBM and LHRF populations in relation to the B73 genome for entire chromosomes. The complete genetic maps contain both framework and placed markers. In the ladder diagrams of the two left panels, positions of the markers correspond to their index in the ordered maps and not to their genetic position. Numbers in parentheses indicate the map coordinate in cM for IBM or LHRF genetic maps and in Mbp for the B73 genome sequence. In the right panel, positions of the markers are proportional to the cM or Mb map coordinate. The ladders have their scales adjusted to fit the two maps to the same height. In the right panel, genetic maps are scaled to the physical map length. Blue rectangles indicate marker intervals containing the centromere, according to MaizeGDB. (PDF) [file pone.0028334.s005.pdf]
