# Supplementary material for: A Large Maize (Zea mays L.) SNP Genotyping Array: Development and Germplasm Genotyping, and Genetic Mapping to Compare with the B73 Reference Genome
Source: PLoS One. 2011 Dec 8;6(12):e28334. doi: 10.1371/journal.pone.0028334 (PMC3234264; doi:10.1371/journal.pone.0028334)
Supplement: Figure S2 — Allele frequency distribution for all polymorphic markers in the two mapping populations. Allele frequencies of the parent B73 in the IBM population (lower part, red dots), and of the parent F2 in the LHRF population (upper part, blue dots) for all SNPs mapped and all chromosomes. Lines represent 1% confidence intervals of the expected 0.5 value under Mendelian segregation. (PDF) [file pone.0028334.s002.pdf]

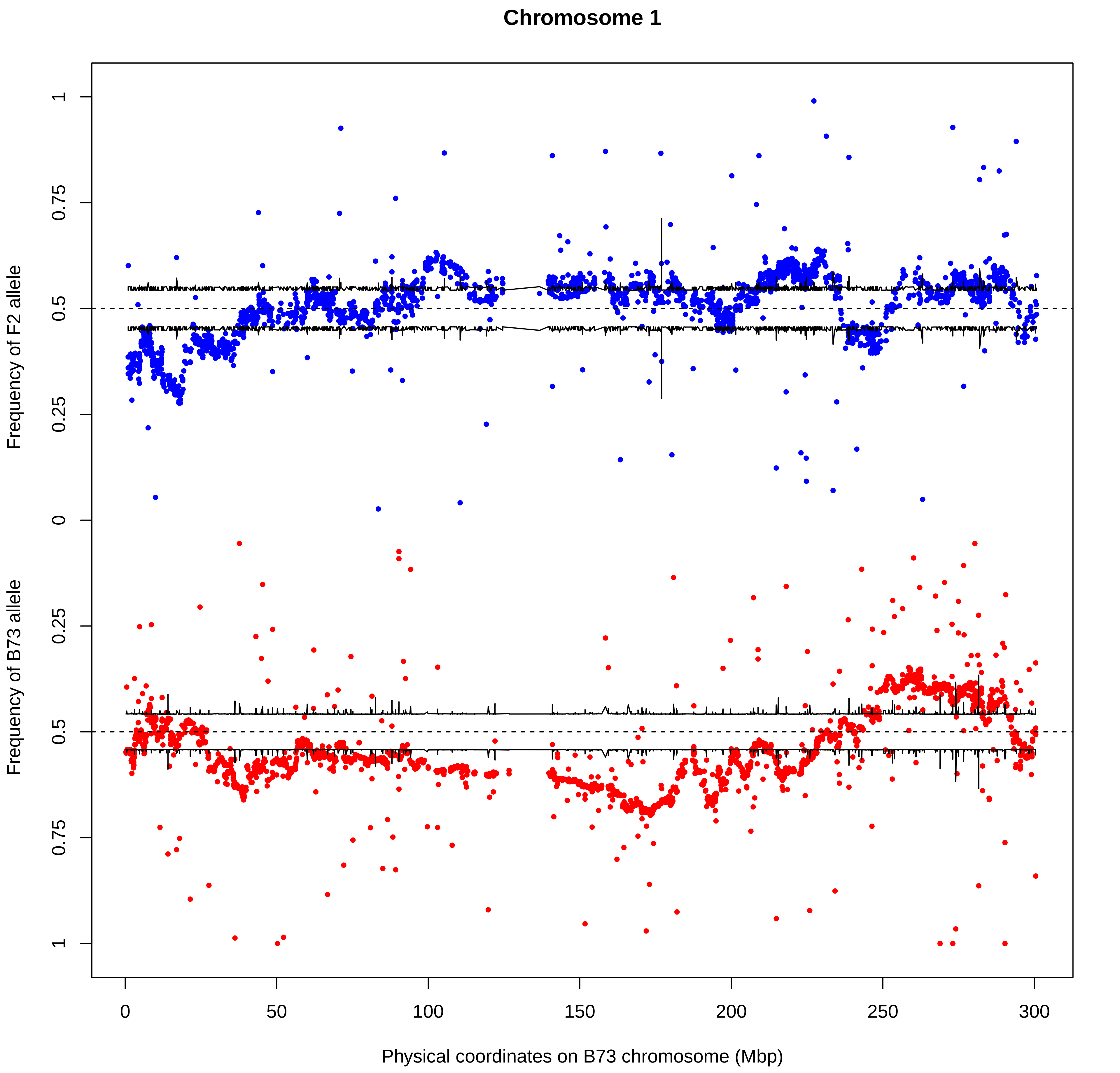

Chromosome 1
Frequency of F2 allele
Frequency of B73 allele
1
0.75
0.5
0.25
0
0.25
0.5
0.75
1
0
50
100
150
200
250
300
Physical coordinates on B73 chromosome (Mbp)

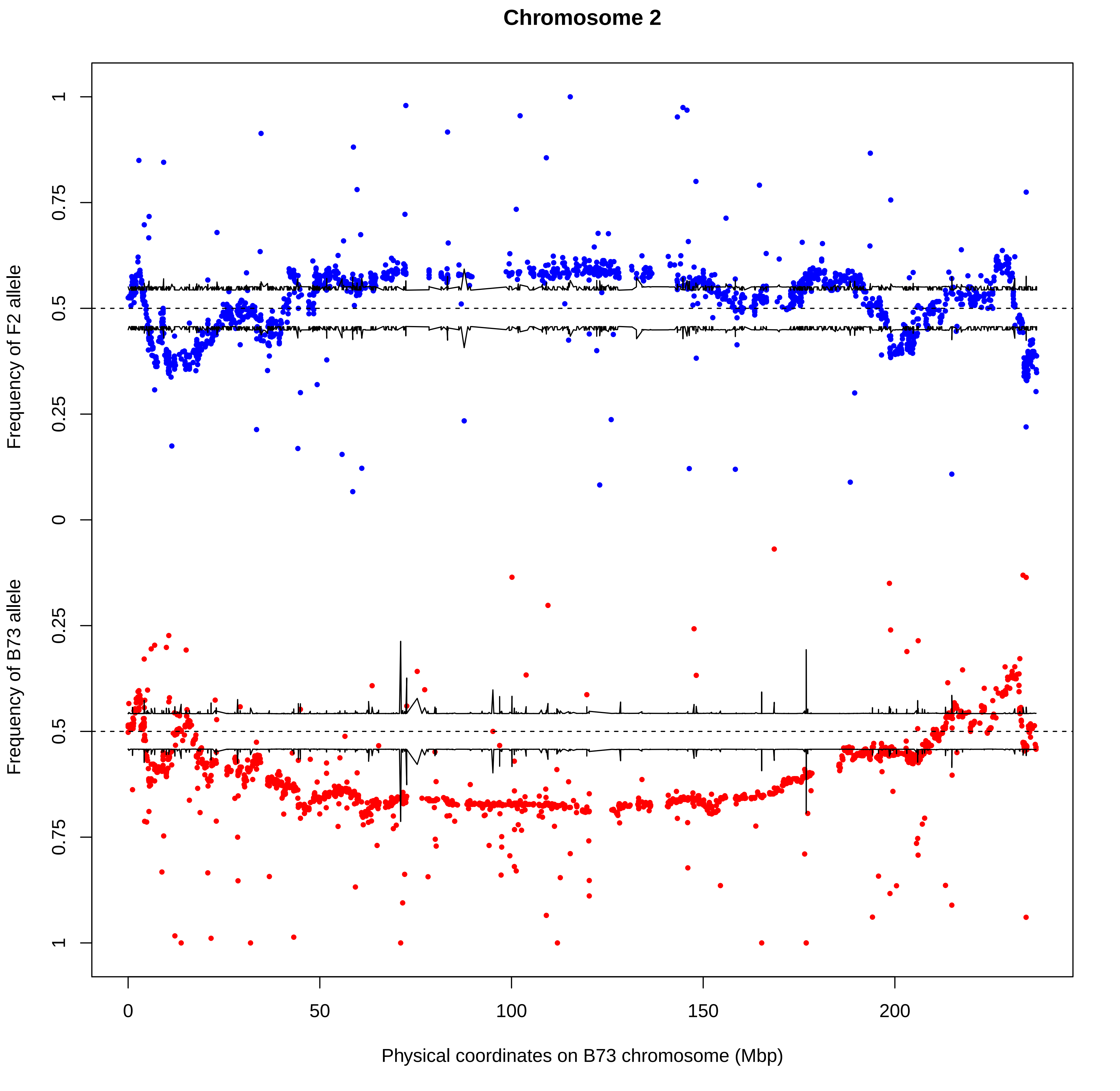
Chromosome 2
Frequency of F2 allele
Frequency of B73 allele
1
0.75
0.5
0.25
0
0.25
0.5
0.75
1
0
50
100
150
200
Physical coordinates on B73 chromosome (Mbp)

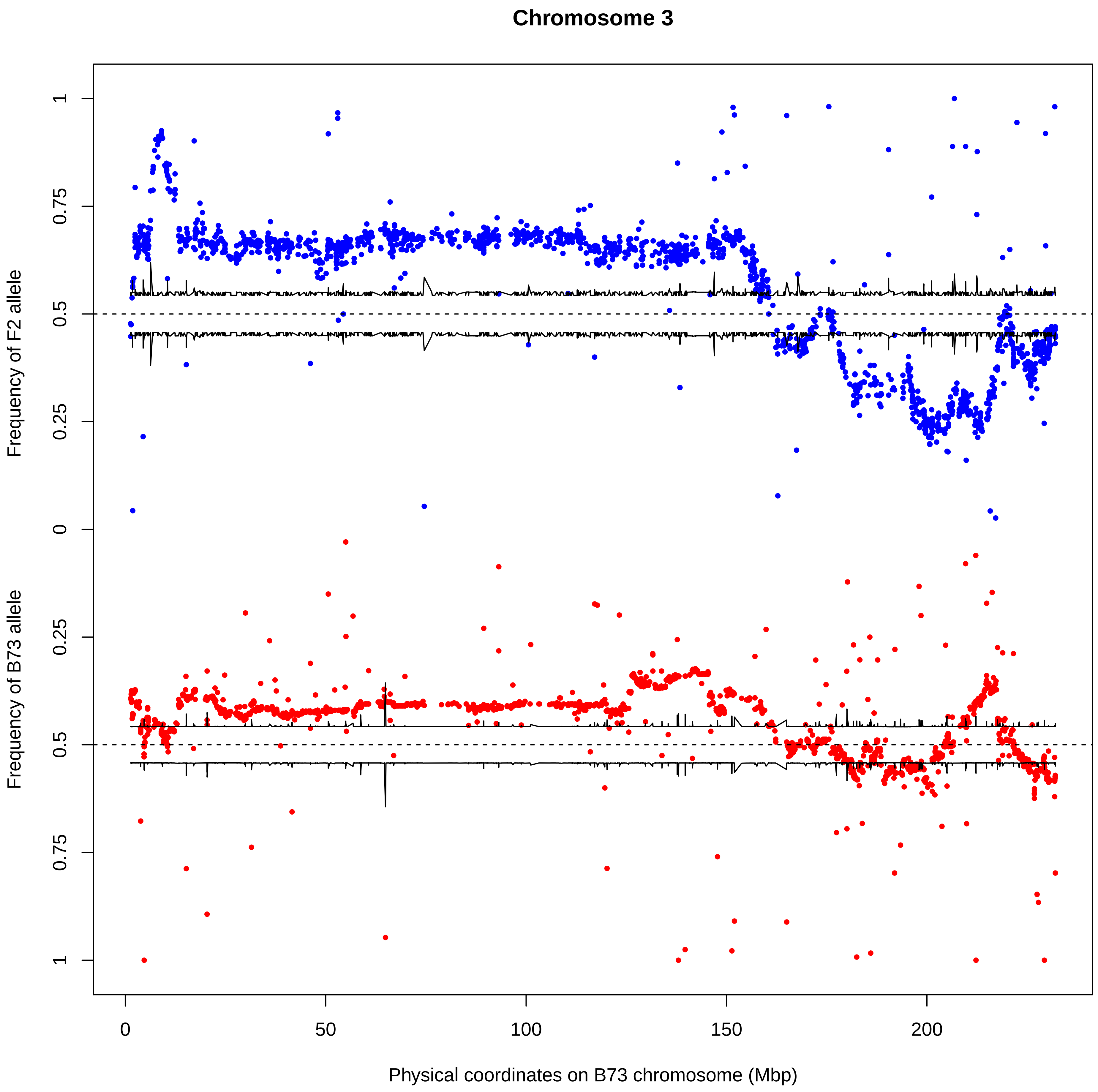

Chromosome 3
Frequency of F2 allele
Frequency of B73 allele
1
0.75
0.5
0.25
0
0.25
0.5
0.75
1
0
50
100
150
200
Physical coordinates on B73 chromosome (Mbp)

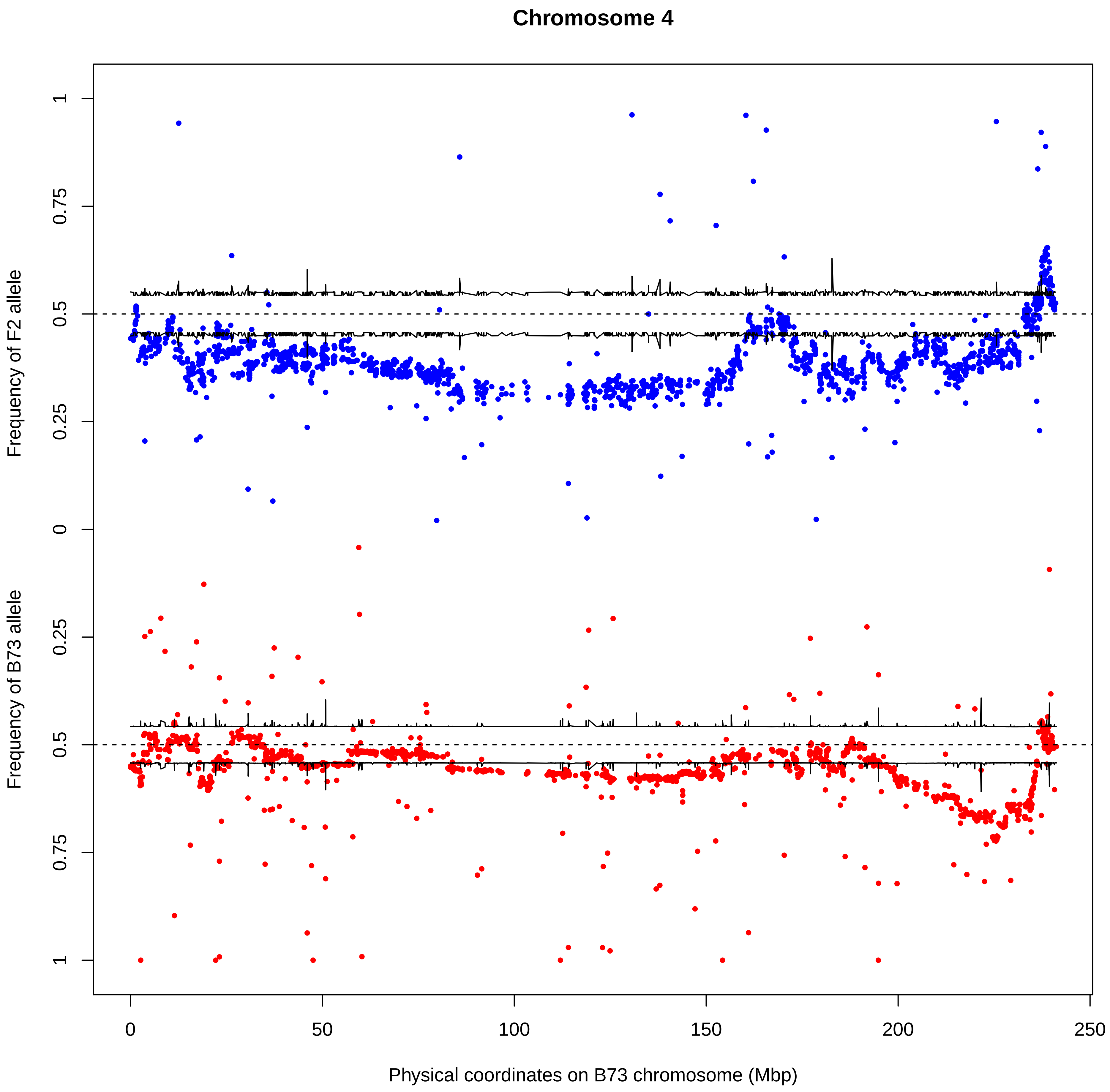

Chromosome 4
Frequency of F2 allele
Frequency of B73 allele
1
0.75
0.5
0.25
0
0.25
0.5
0.75
1
0
50
100
150
200
250
Physical coordinates on B73 chromosome (Mbp)

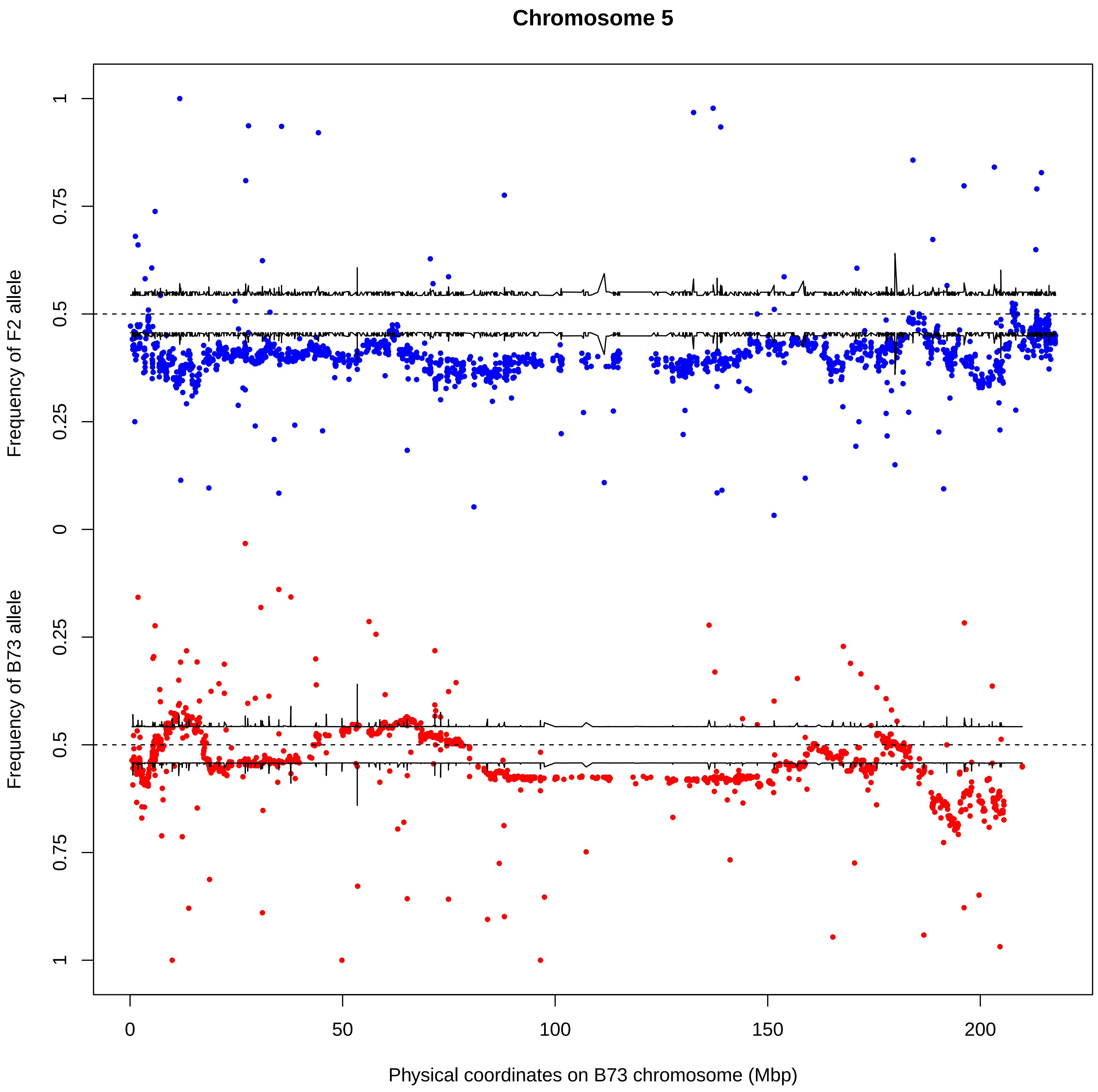
Chromosome 5
Frequency of F2 allele
Frequency of B73 allele
1
0.75
0.5
0.25
0
0.25
0.5
0.75
1
0
50
100
150
200
Physical coordinates on B73 chromosome (Mbp)

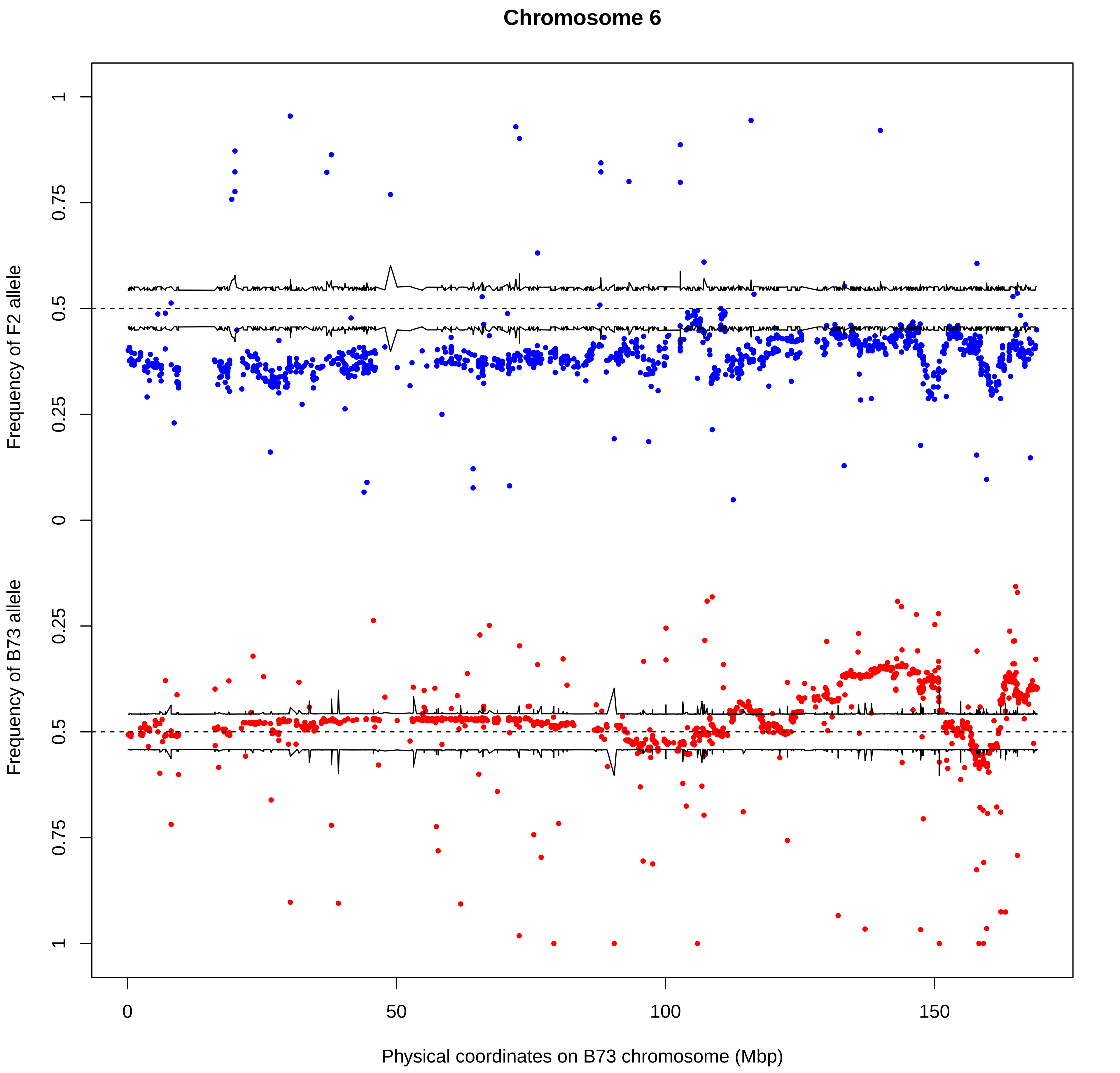

Chromosome 6
Frequency of F2 allele
Frequency of B73 allele
1
0.75
0.5
0.25
0
0.25
0.5
0.75
1
0
50
100
150
Physical coordinates on B73 chromosome (Mbp)

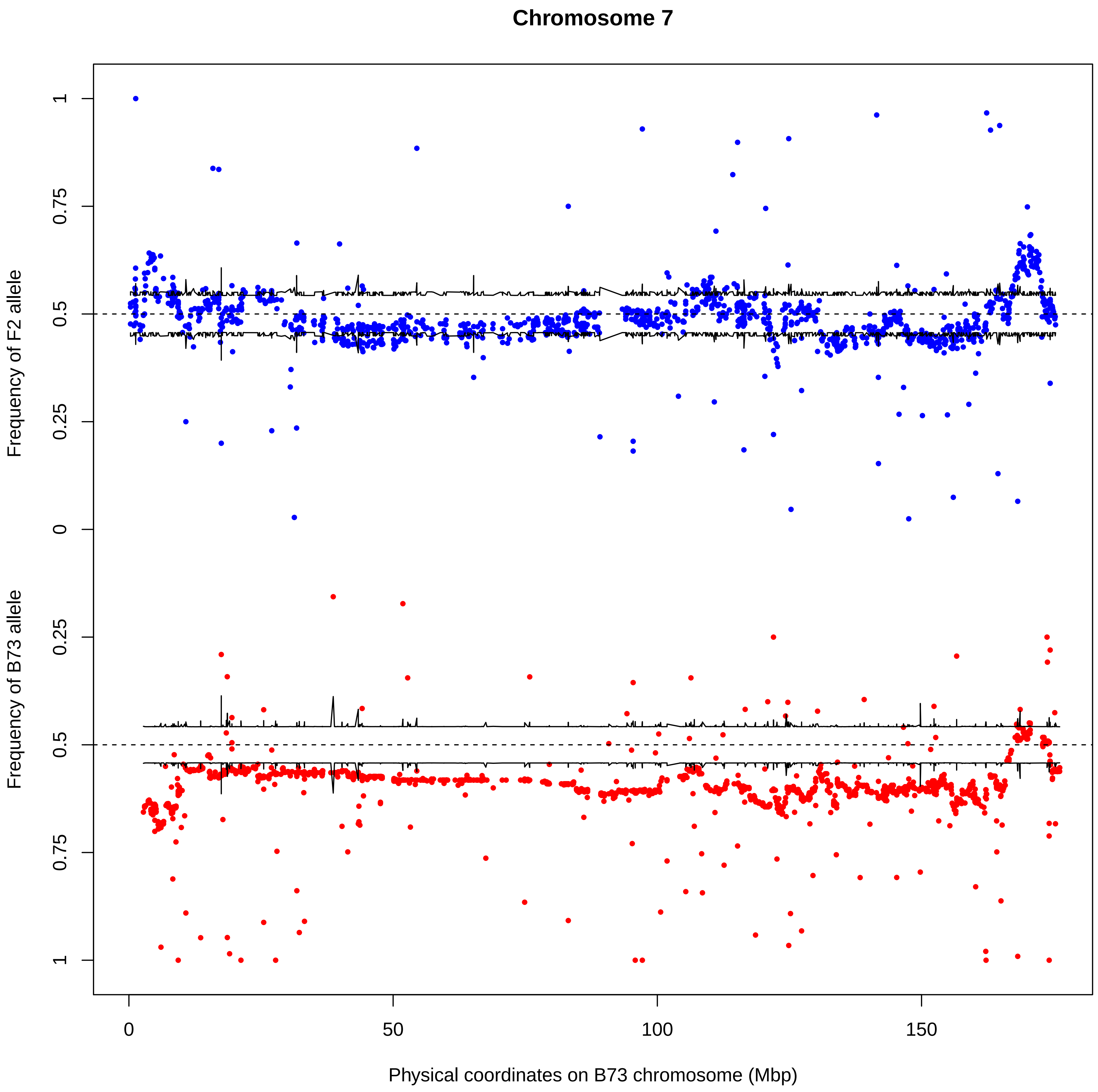

Chromosome 7
Frequency of F2 allele
Frequency of B73 allele
1
0.75
0.5
0.25
0
0.25
0.5
0.75
1
0
50
100
150
Physical coordinates on B73 chromosome (Mbp)

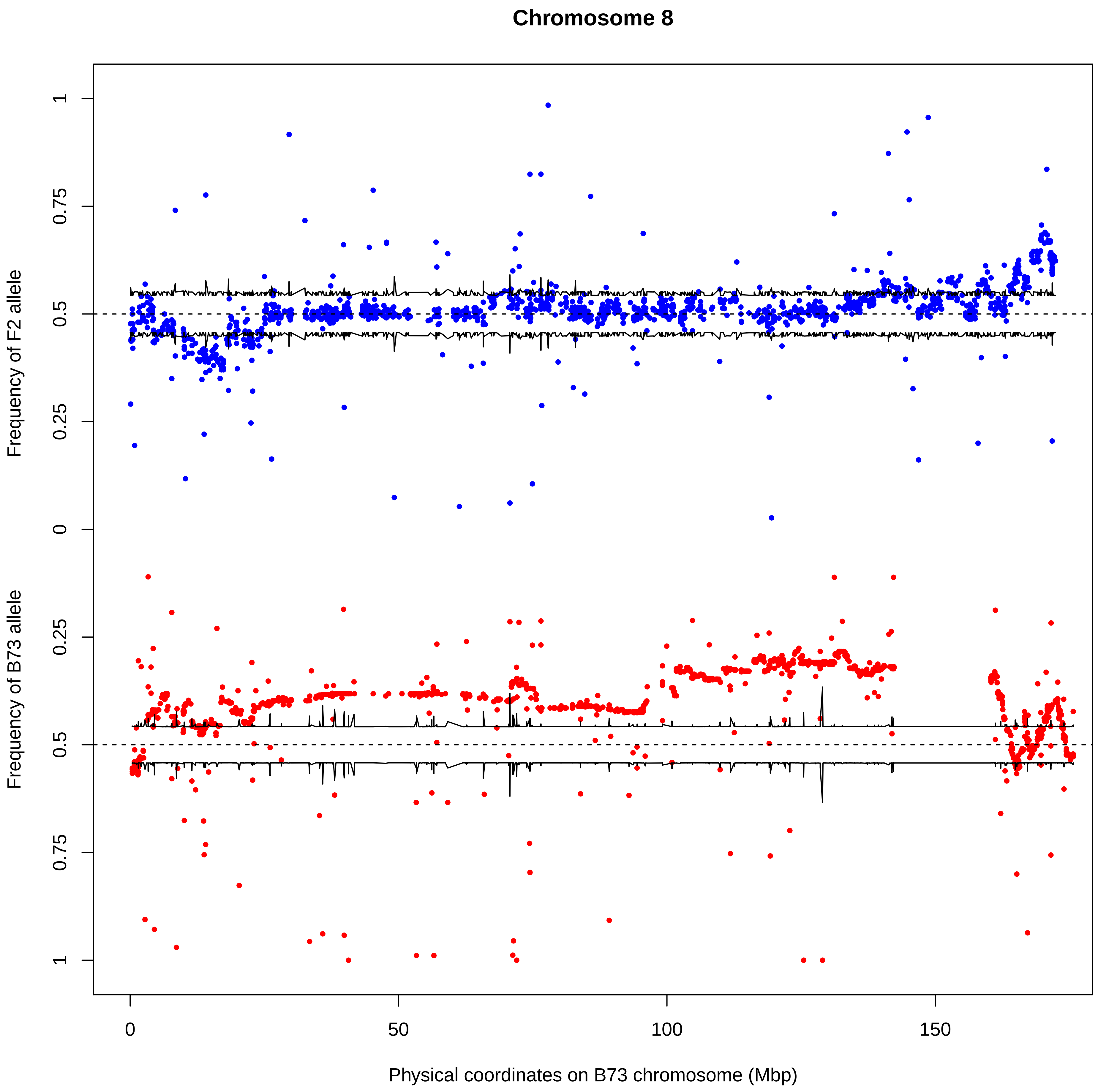
Chromosome 8
Frequency of F2 allele
Frequency of B73 allele
Physical coordinates on B73 chromosome (Mbp)
1
0.75
0.5
0.25
0
0.25
0.5
0.75
1
0
50
100
150

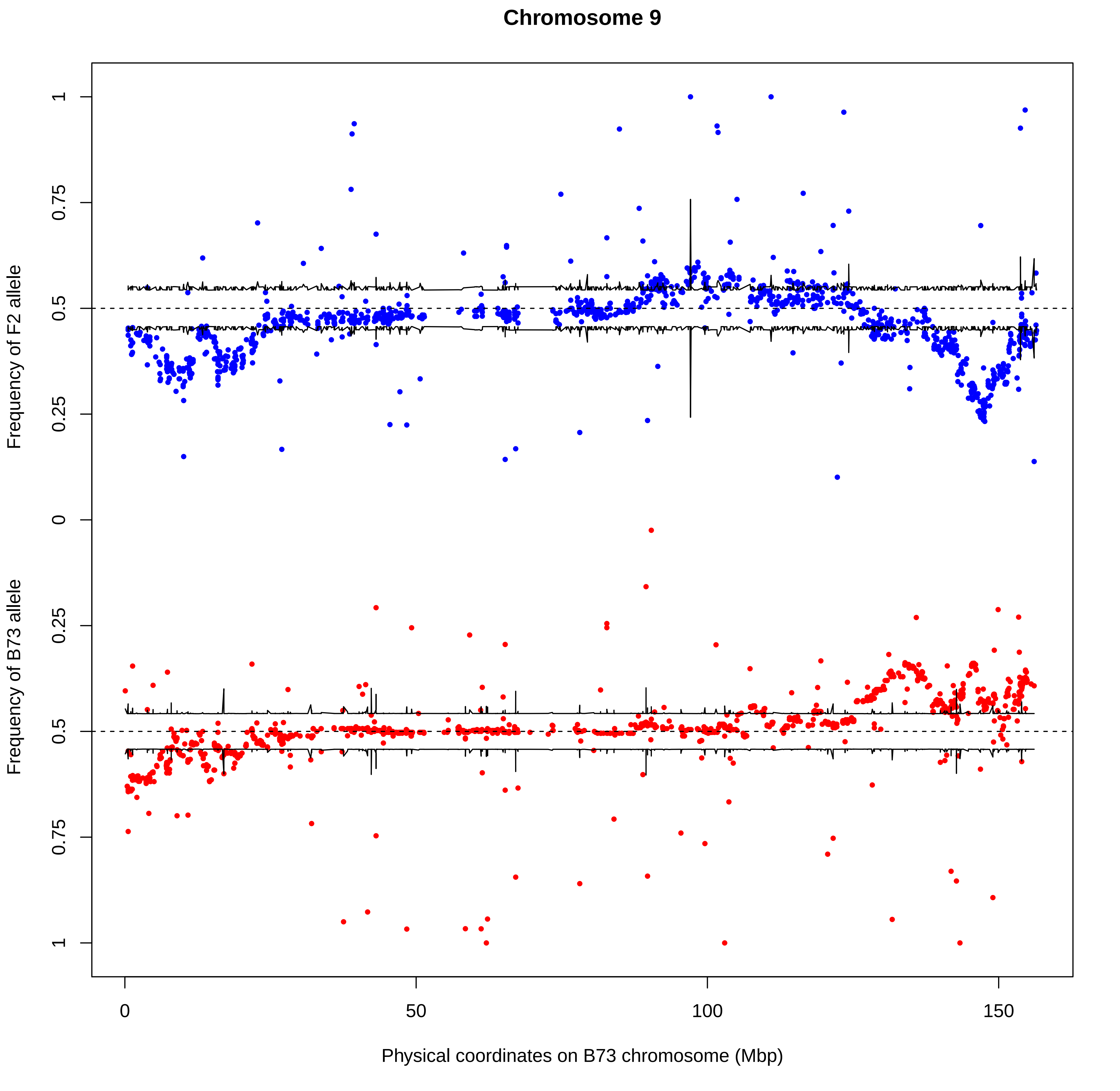

Chromosome 9
Frequency of F2 allele
Frequency of B73 allele
1
0.75
0.5
0.25
0
0.25
0.5
0.75
1
0
50
100
150
Physical coordinates on B73 chromosome (Mbp)

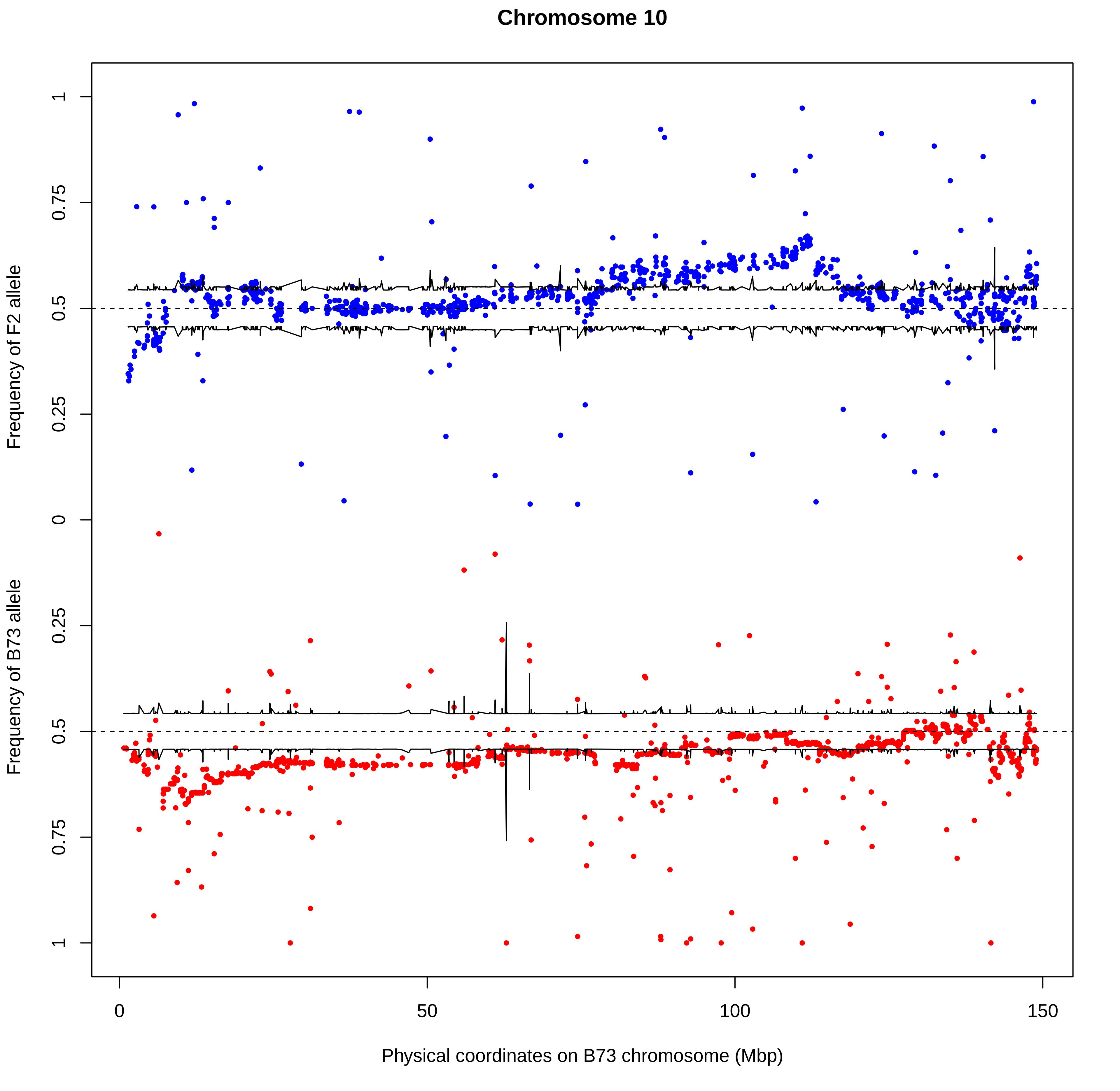
Chromosome 10
Frequency of F2 allele
Frequency of B73 allele
1
0.75
0.5
0.25
0
0.25
0.5
0.75
1
0
50
100
150
Physical coordinates on B73 chromosome (Mbp)
