# Supplementary material for: A Large Maize (Zea mays L.) SNP Genotyping Array: Development and Germplasm Genotyping, and Genetic Mapping to Compare with the B73 Reference Genome
Source: PLoS One. 2011 Dec 8;6(12):e28334. doi: 10.1371/journal.pone.0028334 (PMC3234264; doi:10.1371/journal.pone.0028334)

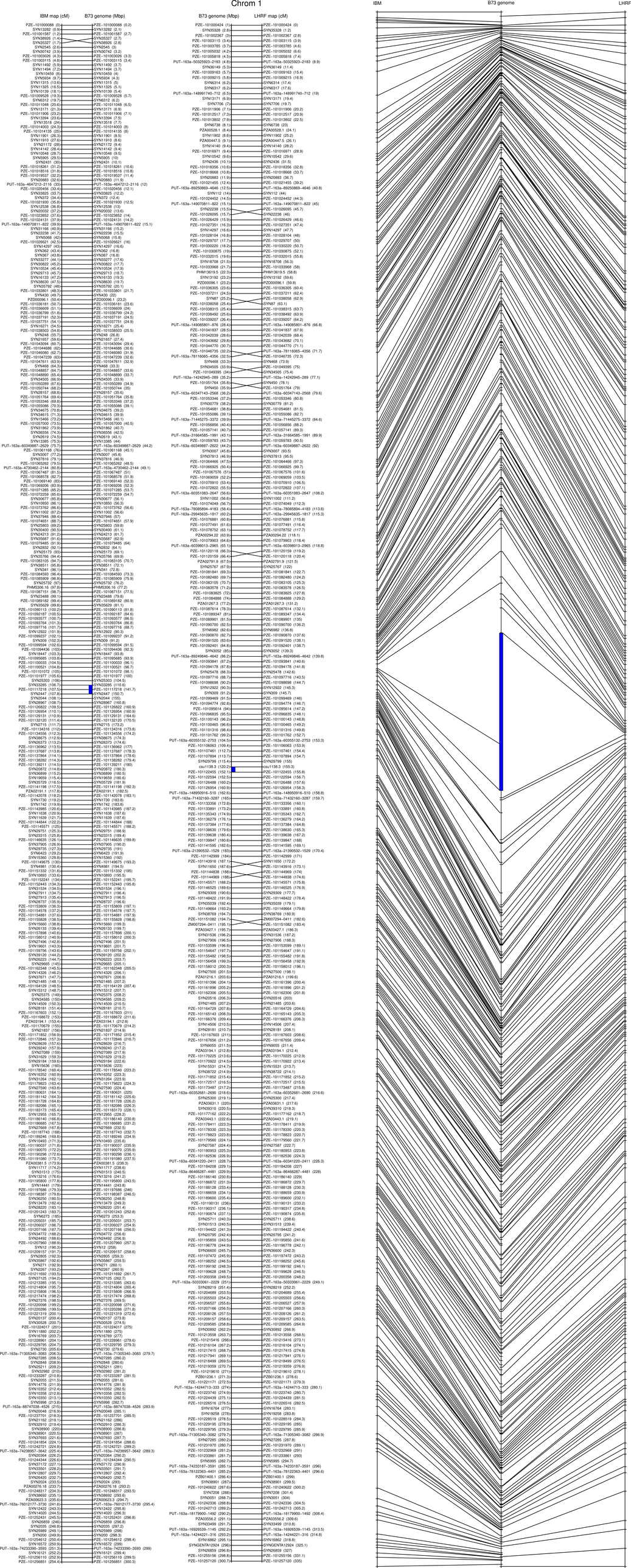
Chrom 1
IBM map (cM)
B73 genome (Mbp)
B73 genome (Mbp)
LHRF map (cM)
IBM
B73 genome
LHRF

Chrom 2

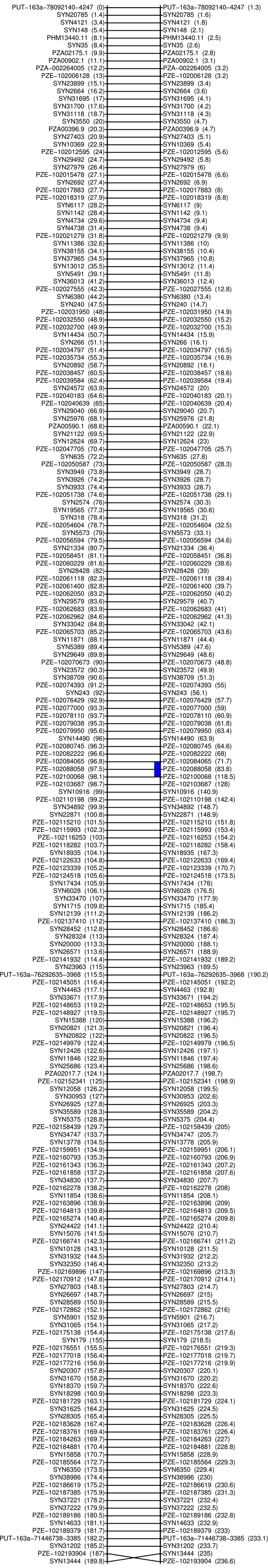
IBM map (cM)
B73 genome (Mbp)
PUT−163a−78092140−4247 (0)
PUT−163a−78092140−4247 (1.3)
SYN20785 (1.4)
SYN20785 (1.6)
SYN4121 (3.4)
SYN4121 (1.8)
SYN148 (5.4)
SYN148 (2.1)
PHM13440.11 (8.1)
PHM13440.11 (2.5)
SYN35 (8.4)
SYN35 (2.6)
PZA02175.1 (9.9)
PZA02175.1 (2.8)
PZA00902.1 (11.1)
PZA00902.1 (3.1)
PZA−002264005 (12.2)
PZA−002264005 (3.2)
PZE−102006128 (13)
PZE−102006128 (3.2)
SYN23899 (15.1)
SYN23899 (3.4)
SYN2664 (16.2)
SYN2664 (3.6)
SYN31695 (17)
SYN31695 (4.1)
SYN31700 (17.6)
SYN31700 (4.2)
SYN31118 (18.7)
SYN31118 (4.3)
SYN3550 (20)
SYN3550 (4.7)
PZA00396.9 (20.3)
PZA00396.9 (4.7)
SYN27403 (20.9)
SYN27403 (5.1)
SYN10369 (22.9)
SYN10369 (5.4)
PZE−102012595 (24)
PZE−102012595 (5.6)
SYN29492 (24.7)
SYN29492 (5.8)
SYN27979 (26.4)
SYN27979 (6)
PZE−102015478 (27.1)
PZE−102015478 (6.6)
SYN2692 (27.4)
SYN2692 (6.9)
PZE−102017883 (27.7)
PZE−102017883 (8)
PZE−102018319 (27.9)
PZE−102018319 (8.8)
SYN6117 (28.2)
SYN6117 (9)
SYN1142 (28.4)
SYN1142 (9.1)
SYN4734 (29.6)
SYN4734 (9.4)
SYN4738 (31.4)
SYN4738 (9.4)
PZE−102021279 (31.8)
PZE−102021279 (9.9)
SYN11386 (32.6)
SYN11386 (10)
SYN38155 (34.1)
SYN38155 (10.4)
SYN37965 (34.5)
SYN37965 (10.8)
SYN13012 (35.5)
SYN13012 (11.4)
SYN5491 (39.1)
SYN5491 (11.8)
SYN36013 (41.2)
SYN36013 (12.4)
PZE−102027555 (42.3)
PZE−102027555 (12.8)
SYN6380 (44.2)
SYN6380 (13.4)
SYN240 (47.5)
SYN240 (14.7)
PZE−102031950 (48)
PZE−102031950 (14.9)
PZE−102032550 (48.9)
PZE−102032550 (15.2)
PZE−102032700 (49.9)
PZE−102032700 (15.3)
SYN14434 (50.7)
SYN14434 (15.9)
SYN266 (51.1)
SYN266 (16.1)
PZE−102034797 (51.4)
PZE−102034797 (16.5)
PZE−102035734 (55.3)
PZE−102035734 (16.9)
SYN20892 (58.7)
SYN20892 (18.1)
PZE−102038457 (60.5)
PZE−102038457 (18.6)
PZE−102039584 (62.4)
PZE−102039584 (19.4)
SYN24572 (63.9)
SYN24572 (20)
PZE−102040183 (64.6)
PZE−102040183 (20.1)
PZE−102040639 (65)
PZE−102040639 (20.4)
SYN29040 (66.9)
SYN29040 (20.7)
SYN25976 (68.1)
SYN25976 (21.8)
PZA00590.1 (68.6)
PZA00590.1 (22.1)
SYN21122 (69.5)
SYN21122 (22.9)
SYN12624 (69.7)
SYN12624 (23)
PZE−102047705 (70.4)
PZE−102047705 (25.7)
SYN635 (72.2)
SYN635 (27.8)
PZE−102050587 (73)
PZE−102050587 (28.3)
SYN3949 (73.8)
SYN3949 (28.7)
SYN3926 (74.2)
SYN3926 (28.7)
SYN3933 (74.4)
SYN3933 (28.7)
PZE−102051738 (74.6)
PZE−102051738 (29.1)
SYN2574 (76)
SYN2574 (30.3)
SYN19565 (77.3)
SYN19565 (30.6)
SYN318 (78.4)
SYN318 (31.2)
PZE−102054604 (78.7)
PZE−102054604 (32.5)
SYN5573 (79)
SYN5573 (33.1)
PZE−102056594 (79.5)
PZE−102056594 (34.6)
SYN21334 (80.7)
SYN21334 (36.4)
PZE−102058451 (81.1)
PZE−102058451 (36.8)
PZE−102060229 (81.6)
PZE−102060229 (38.6)
SYN28428 (82)
SYN28428 (39)
PZE−102061118 (82.3)
PZE−102061118 (39.4)
PZE−102061400 (82.8)
PZE−102061400 (39.7)
PZE−102062050 (83.2)
PZE−102062050 (40.2)
SYN29579 (83.6)
SYN29579 (40.7)
PZE−102062683 (83.9)
PZE−102062683 (41)
PZE−102062962 (84.6)
PZE−102062962 (41.3)
SYN33042 (84.8)
SYN33042 (42.1)
PZE−102065703 (85.2)
PZE−102065703 (43.6)
SYN11871 (88.1)
SYN11871 (44.4)
SYN5389 (89.4)
SYN5389 (47.6)
SYN29649 (89.8)
SYN29649 (48.6)
PZE−102070673 (90)
PZE−102070673 (48.8)
SYN23572 (90.3)
SYN23572 (49.9)
SYN38709 (90.6)
SYN38709 (51.3)
PZE−102074393 (91.2)
PZE−102074393 (55)
SYN243 (92)
SYN243 (56.1)
PZE−102076429 (92.9)
PZE−102076429 (57.7)
PZE−102077000 (93.3)
PZE−102077000 (59)
PZE−102078110 (93.7)
PZE−102078110 (60.9)
PZE−102079038 (95.3)
PZE−102079038 (61.8)
PZE−102079950 (95.6)
PZE−102079950 (63.4)
SYN14490 (96)
SYN14490 (63.9)
PZE−102080745 (96.3)
PZE−102080745 (64.6)
PZE−102082222 (96.6)
PZE−102082222 (68)
PZE−102084065 (96.8)
PZE−102084065 (71.7)
PZE−102088058 (97.5)
PZE−102088058 (83.8)
PZE−102100068 (98.1)
PZE−102100068 (118.5)
PZE−102103687 (98.7)
PZE−102103687 (128)
SYN10916 (99)
SYN10916 (140.9)
PZE−102110198 (99.2)
PZE−102110198 (142.4)
SYN34892 (99.9)
SYN34892 (148.7)
SYN22871 (100.8)
SYN22871 (148.9)
PZE−102115210 (101.5)
PZE−102115210 (151.8)
PZE−102115993 (102.3)
PZE−102115993 (153.4)
PZE−102116253 (103)
PZE−102116253 (154.2)
PZE−102118282 (103.7)
PZE−102118282 (158.4)
SYN18935 (104.1)
SYN18935 (167.3)
PZE−102122633 (104.8)
PZE−102122633 (169.4)
PZE−102123339 (105.2)
PZE−102123339 (170.7)
PZE−102124518 (105.6)
PZE−102124518 (173.5)
SYN17434 (105.9)
SYN17434 (176)
SYN6028 (106.1)
SYN6028 (176.5)
SYN33470 (107)
SYN33470 (177.9)
SYN1715 (109.8)
SYN1715 (185.4)
SYN12139 (111.2)
SYN12139 (186.2)
PZE−102137410 (112)
PZE−102137410 (186.3)
SYN28452 (112.8)
SYN28452 (186.6)
SYN28324 (113)
SYN28324 (187.4)
SYN20000 (113.3)
SYN20000 (188.1)
SYN26571 (113.6)
SYN26571 (188.9)
PZE−102141932 (114.4)
PZE−102141932 (189.2)
SYN23963 (115)
SYN23963 (189.5)
PUT−163a−76292635−3968 (115.5)
PUT−163a−76292635−3968 (190.2)
PZE−102145051 (116.4)
PZE−102145051 (192.2)
SYN4463 (117.1)
SYN4463 (192.8)
SYN33671 (117.9)
SYN33671 (194.2)
PZE−102148653 (119.2)
PZE−102148653 (195.5)
PZE−102148927 (119.5)
PZE−102148927 (195.7)
SYN15388 (120)
SYN15388 (196.2)
SYN20821 (121.3)
SYN20821 (196.4)
SYN20822 (122)
SYN20822 (196.5)
PZE−102149979 (122.4)
PZE−102149979 (196.5)
SYN12426 (122.6)
SYN12426 (197.1)
SYN11846 (122.9)
SYN11846 (197.4)
SYN25686 (123.4)
SYN25686 (198.6)
PZA02017.7 (124.1)
PZA02017.7 (198.7)
PZE−102152341 (125)
PZE−102152341 (198.9)
SYN12058 (126.2)
SYN12058 (199.5)
SYN30953 (127)
SYN30953 (202.6)
SYN26925 (127.8)
SYN26925 (203.3)
SYN35589 (128.3)
SYN35589 (204.2)
SYN5375 (128.8)
SYN5375 (204.4)
PZE−102158439 (129.7)
PZE−102158439 (205)
SYN34747 (133.7)
SYN34747 (205.7)
SYN13778 (134.5)
SYN13778 (205.9)
PZE−102159951 (134.9)
PZE−102159951 (206.1)
PZE−102160793 (135.3)
PZE−102160793 (206.9)
PZE−102161343 (136.3)
PZE−102161343 (207.2)
PZE−102161858 (137.2)
PZE−102161858 (207.6)
SYN34830 (137.7)
SYN34830 (207.7)
PZE−102162278 (138.2)
PZE−102162278 (208)
SYN11854 (138.6)
SYN11854 (208.1)
PZE−102163896 (138.9)
PZE−102163896 (209)
PZE−102164813 (139.8)
PZE−102164813 (209.5)
PZE−102165274 (140.4)
PZE−102165274 (209.8)
SYN24422 (141.1)
SYN24422 (210.4)
SYN15076 (141.5)
SYN15076 (210.7)
PZE−102166741 (142.3)
PZE−102166741 (211.2)
SYN10128 (143.1)
SYN10128 (211.5)
SYN31932 (144.5)
SYN31932 (212.2)
SYN32350 (146.4)
SYN32350 (213.2)
PZE−102169896 (147)
PZE−102169896 (213.3)
PZE−102170912 (147.8)
PZE−102170912 (214.1)
SYN27803 (148.1)
SYN27803 (214.7)
SYN26697 (148.7)
SYN26697 (215)
SYN28589 (150.9)
SYN28589 (215.5)
PZE−102172862 (152.1)
PZE−102172862 (216)
SYN5901 (152.9)
SYN5901 (216.7)
SYN31065 (154.1)
SYN31065 (217.2)
PZE−102175138 (154.4)
PZE−102175138 (217.6)
SYN179 (155)
SYN179 (218.5)
PZE−102176551 (155.5)
PZE−102176551 (219.3)
PZE−102177018 (156.4)
PZE−102177018 (219.7)
PZE−102177216 (156.9)
PZE−102177216 (219.9)
SYN20307 (157.8)
SYN20307 (220.1)
SYN31670 (158.2)
SYN31670 (220.2)
SYN18370 (159.7)
SYN18370 (222.6)
SYN18298 (160.9)
SYN18298 (223.3)
PZE−102181729 (163.1)
PZE−102181729 (224.1)
SYN31625 (164.2)
SYN31625 (224.5)
SYN28305 (165.4)
SYN28305 (225.5)
PZE−102183628 (167.4)
PZE−102183628 (226.4)
PZE−102183761 (169.4)
PZE−102183761 (226.4)
PZE−102184263 (169.7)
PZE−102184263 (227)
PZE−102184881 (170.4)
PZE−102184881 (228.8)
SYN15858 (170.7)
SYN15858 (228.9)
PZE−102185564 (172.7)
PZE−102185564 (229.3)
SYN6350 (173.5)
SYN6350 (229.4)
SYN38986 (174.4)
SYN38986 (230)
PZE−102186619 (175.2)
PZE−102186619 (230.6)
PZE−102187385 (175.9)
PZE−102187385 (231.3)
SYN37221 (178.2)
SYN37221 (232.4)
SYN37222 (179.9)
SYN37222 (232.5)
PZE−102189186 (180.5)
PZE−102189186 (232.8)
SYN14633 (181.1)
SYN14633 (232.9)
PZE−102189379 (181.7)
PZE−102189379 (233)
PUT−163a−71446738−3385 (182.2)
PUT−163a−71446738−3385 (233.1)
SYN31202 (185.2)
SYN31202 (233.7)
PZE−102193904 (187)
SYN13444 (235)
SYN13444 (189.8)
PZE−102193904 (236.6)

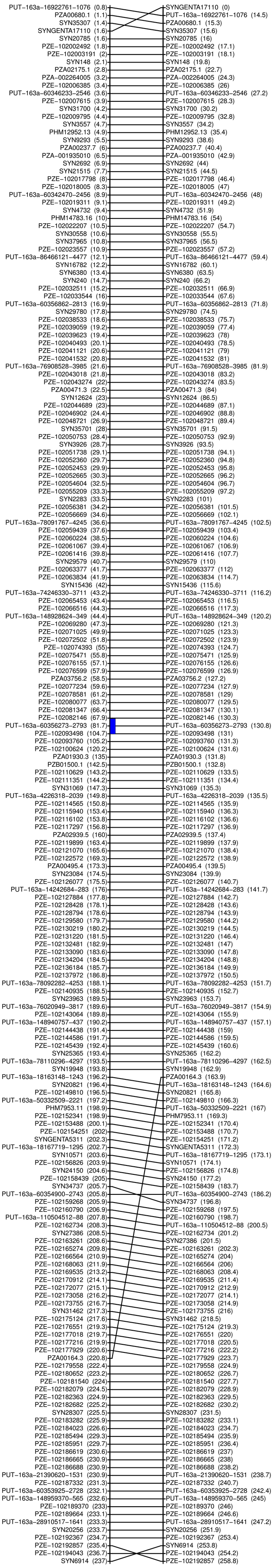
B73 genome (Mbp)
LHRF map (cM)
PUT−163a−16922761−1076 (0.8)
SYNGENTA17110 (0)
PZA00680.1 (1.1)
PUT−163a−16922761−1076 (14.5)
SYN35307 (1.4)
PZA00680.1 (15.3)
SYNGENTA17110 (1.6)
SYN35307 (15.6)
SYN20785 (1.6)
SYN20785 (16)
PZE−102002492 (1.8)
PZE−102002492 (17.1)
PZE−102003191 (2)
PZE−102003191 (18.1)
SYN148 (2.1)
SYN148 (19.8)
PZA02175.1 (2.8)
PZA02175.1 (22.7)
PZA−002264005 (3.2)
PZA−002264005 (24.3)
PZE−102006385 (3.4)
PZE−102006385 (26)
PUT−163a−60346233−2546 (3.6)
PUT−163a−60346233−2546 (27.2)
PZE−102007615 (3.9)
PZE−102007615 (28.3)
SYN31700 (4.2)
SYN31700 (30.2)
PZE−102009795 (4.4)
PZE−102009795 (32.8)
SYN3557 (4.7)
SYN3557 (34.2)
PHM12952.13 (4.9)
PHM12952.13 (35.4)
SYN9293 (5.5)
SYN9293 (38.6)
PZA00237.7 (6)
PZA00237.7 (40.4)
PZA−001935010 (6.5)
PZA−001935010 (42.9)
SYN2692 (6.9)
SYN2692 (44)
SYN21515 (7.7)
SYN21515 (44.5)
PZE−102017798 (8)
PZE−102017798 (46.4)
PZE−102018005 (8.3)
PZE−102018005 (47)
PUT−163a−60342470−2456 (8.9)
PUT−163a−60342470−2456 (48)
PZE−102019311 (9.1)
PZE−102019311 (49.2)
SYN4732 (9.4)
SYN4732 (51.9)
PHM14783.16 (10)
PHM14783.16 (54)
PZE−102022207 (10.5)
PZE−102022207 (54.7)
SYN30558 (10.6)
SYN30558 (55.5)
SYN37965 (10.8)
SYN37965 (56.5)
PZE−102023557 (10.9)
PZE−102023557 (57.2)
PUT−163a−86466121−4477 (12.1)
PUT−163a−86466121−4477 (59.4)
SYN16782 (12.2)
SYN16782 (60.1)
SYN6380 (13.4)
SYN6380 (63.5)
SYN240 (14.7)
SYN240 (66.2)
PZE−102032511 (15.2)
PZE−102032511 (66.9)
PZE−102033544 (16)
PZE−102033544 (67.6)
PUT−163a−60356862−2813 (16.9)
PUT−163a−60356862−2813 (71.8)
SYN29780 (17.8)
SYN29780 (74.5)
PZE−102038533 (18.6)
PZE−102038533 (75.7)
PZE−102039059 (19.2)
PZE−102039059 (77.4)
PZE−102039623 (19.4)
PZE−102039623 (78)
PZE−102040493 (20.1)
PZE−102040493 (78.5)
PZE−102041121 (20.6)
PZE−102041121 (79)
PZE−102041532 (20.8)
PZE−102041532 (81)
PUT−163a−76908528−3985 (21.6)
PUT−163a−76908528−3985 (81.9)
PZE−102043018 (21.8)
PZE−102043018 (83.2)
PZE−102043274 (22)
PZE−102043274 (83.5)
PZA00471.3 (22.5)
PZA00471.3 (84)
SYN12624 (23)
SYN12624 (86.5)
PZE−102044689 (23)
PZE−102044689 (87.1)
PZE−102046902 (24.4)
PZE−102046902 (88.8)
PZE−102048721 (26.9)
PZE−102048721 (89.4)
SYN35701 (28)
SYN35701 (91.5)
PZE−102050753 (28.4)
PZE−102050753 (92.9)
SYN3926 (28.7)
SYN3926 (93.5)
PZE−102051738 (29.1)
PZE−102051738 (94.1)
PZE−102052360 (29.7)
PZE−102052360 (94.8)
PZE−102052453 (29.9)
PZE−102052453 (95.8)
PZE−102052665 (30.3)
PZE−102052665 (96.2)
PZE−102054604 (32.5)
PZE−102054604 (96.7)
PZE−102055209 (33.3)
PZE−102055209 (97.2)
SYN2283 (33.5)
SYN2283 (101)
PZE−102056381 (34.2)
PZE−102056381 (101.5)
PZE−102056669 (34.6)
PZE−102056669 (102.1)
PUT−163a−78091767−4245 (36.6)
PUT−163a−78091767−4245 (102.5)
PZE−102059439 (37.6)
PZE−102059439 (103.4)
PZE−102060224 (38.5)
PZE−102060224 (104.6)
PZE−102061067 (39.4)
PZE−102061067 (106.9)
PZE−102061416 (39.8)
PZE−102061416 (107.7)
SYN29579 (40.7)
SYN29579 (110)
PZE−102063377 (41.7)
PZE−102063377 (112)
PZE−102063834 (41.9)
PZE−102063834 (114.7)
SYN15436 (42)
SYN15436 (115.6)
PUT−163a−74246330−3711 (43.2)
PUT−163a−74246330−3711 (116.2)
PZE−102065453 (43.4)
PZE−102065453 (116.5)
PZE−102066516 (44.3)
PZE−102066516 (117.3)
PUT−163a−148928624−349 (44.4)
PUT−163a−148928624−349 (120.2)
PZE−102069280 (47.3)
PZE−102069280 (121.3)
PZE−102071025 (49.9)
PZE−102071025 (123.3)
PZE−102072502 (51.8)
PZE−102072502 (123.9)
PZE−102074393 (55)
PZE−102074393 (124.7)
PZE−102075471 (55.8)
PZE−102075471 (125.9)
PZE−102076155 (57.1)
PZE−102076155 (126.6)
PZE−102076599 (57.9)
PZE−102076599 (126.9)
PZA03756.2 (58.5)
PZA03756.2 (127.2)
PZE−102077234 (59.6)
PZE−102077234 (127.9)
PZE−102078581 (61.2)
PZE−102078581 (129)
PZE−102080077 (63.7)
PZE−102080077 (129.5)
PZE−102081347 (66.4)
PZE−102081347 (130.1)
PZE−102082146 (67.9)
PZE−102082146 (130.3)
PUT−163a−60356273−2793 (81.7)
PUT−163a−60356273−2793 (130.8)
PZE−102093498 (104.7)
PZE−102093498 (131)
PZE−102093760 (105.2)
PZE−102093760 (131.3)
PZE−102100624 (120.2)
PZE−102100624 (131.6)
PZA01930.3 (135)
PZA01930.3 (131.8)
PZB01500.1 (142.5)
PZB01500.1 (132.8)
PZE−102110629 (143.2)
PZE−102110629 (133.5)
PZE−102111351 (144.2)
PZE−102111351 (134.4)
SYN31069 (147.3)
SYN31069 (135.3)
PUT−163a−4226318−2039 (149.8)
PUT−163a−4226318−2039 (135.5)
PZE−102114565 (150.8)
PZE−102114565 (135.9)
PZE−102115940 (153.4)
PZE−102115940 (136.3)
PZE−102116102 (153.8)
PZE−102116102 (136.6)
PZE−102117297 (156.8)
PZE−102117297 (136.9)
PZA02939.5 (160)
PZA02939.5 (137.4)
PZE−102119899 (163.4)
PZE−102119899 (137.9)
PZE−102121070 (165.6)
PZE−102121070 (138.4)
PZE−102122572 (169.3)
PZE−102122572 (138.9)
PZA00495.4 (173.3)
PZA00495.4 (139.5)
SYN23084 (174.5)
SYN23084 (139.9)
PZE−102126077 (175.5)
PZE−102126077 (140.7)
PUT−163a−14242684−283 (176)
PUT−163a−14242684−283 (141.7)
PZE−102127884 (177.8)
PZE−102127884 (142.7)
PZE−102128428 (178.1)
PZE−102128428 (143.6)
PZE−102128794 (178.6)
PZE−102128794 (143.9)
PZE−102129580 (179.7)
PZE−102129580 (144.2)
PZE−102130219 (180.2)
PZE−102130219 (144.5)
PZE−102131220 (181.5)
PZE−102131220 (146.4)
PZE−102132481 (182.9)
PZE−102132481 (147)
PZE−102133090 (183.6)
PZE−102133090 (147.8)
PZE−102134204 (184.5)
PZE−102134204 (148.8)
PZE−102136184 (185.7)
PZE−102136184 (149.9)
PZE−102137972 (186.8)
PZE−102137972 (150.5)
PUT−163a−78092282−4253 (188.1)
PUT−163a−78092282−4253 (151.7)
PZE−102140935 (188.5)
PZE−102140935 (152.7)
SYN23963 (189.5)
SYN23963 (153.7)
PUT−163a−76020949−3817 (189.6)
PUT−163a−76020949−3817 (154.9)
PZE−102143064 (189.8)
PZE−102143064 (155.9)
PUT−163a−148940757−437 (190.2)
PUT−163a−148940757−437 (157.1)
PZE−102144438 (191.4)
PZE−102144438 (159)
PZE−102144586 (191.7)
PZE−102144586 (159.5)
PZE−102145439 (192.4)
PZE−102145439 (160.6)
SYN25365 (193.4)
SYN25365 (162.2)
PUT−163a−78110296−4297 (193.5)
PUT−163a−78110296−4297 (162.5)
SYN19948 (193.8)
SYN19948 (162.9)
PUT−163a−18163148−1243 (196.2)
PZA00164.3 (163.9)
SYN20821 (196.4)
PUT−163a−18163148−1243 (164.6)
PZE−102149810 (196.5)
SYN20821 (165.8)
PUT−163a−50332509−2221 (197.2)
PZE−102149810 (166.3)
PHM7953.11 (198.9)
PUT−163a−50332509−2221 (167)
PZE−102152341 (198.9)
PHM7953.11 (169.3)
PZE−102153488 (200.1)
PZE−102152341 (170.4)
PZE−102154251 (202)
PZE−102153488 (170.7)
SYNGENTA5311 (202.3)
PZE−102154251 (171.2)
PUT−163a−18167719−1295 (202.7)
SYNGENTA5311 (172.3)
SYN10571 (203.6)
PUT−163a−18167719−1295 (173.1)
PZE−102156826 (203.9)
SYN10571 (174.1)
SYN24150 (204.6)
PZE−102156826 (174.8)
PZE−102158439 (205)
SYN24150 (177.2)
SYN34737 (205.7)
PZE−102158439 (183.7)
PUT−163a−60354900−2743 (205.8)
PUT−163a−60354900−2743 (186.2)
PZE−102159268 (205.9)
SYN34737 (196.8)
PZE−102160790 (206.9)
PZE−102159268 (197.5)
PUT−163a−110504512−88 (207.8)
PZE−102160790 (198.7)
PZE−102162734 (208.3)
PUT−163a−110504512−88 (200.5)
SYN27386 (208.5)
PZE−102162734 (201.2)
PZE−102163261 (208.6)
SYN27386 (201.5)
PZE−102165274 (209.8)
PZE−102163261 (202.3)
PZE−102166564 (210.9)
PZE−102165274 (204)
PZE−102168063 (211.9)
PZE−102166564 (206)
PZE−102169535 (213.2)
PZE−102168063 (208.4)
PZE−102170912 (214.1)
PZE−102169535 (211.4)
PZE−102172077 (215.1)
PZE−102170912 (212.9)
PZE−102173058 (216.2)
PZE−102172077 (214.1)
PZE−102173755 (216.7)
PZE−102173058 (214.9)
SYN31462 (217.3)
PZE−102173755 (216)
PZE−102175124 (217.6)
SYN31462 (218.5)
PZE−102176551 (219.3)
PZE−102175124 (219.3)
PZE−102177018 (219.7)
PZE−102176551 (220)
PZE−102177216 (219.9)
PZE−102177018 (220.5)
PZE−102177929 (220.6)
PZE−102177216 (222.2)
PZA00164.3 (220.8)
PZE−102177929 (223.7)
PZE−102179558 (222.4)
PZE−102179558 (224.9)
PZE−102180652 (223.2)
PZE−102180652 (226.7)
PZE−102181540 (224)
PZE−102181540 (227.7)
PZE−102182079 (224.5)
PZE−102182079 (228.9)
PZE−102182363 (224.9)
PZE−102182363 (229.5)
PZE−102182682 (225.2)
PZE−102182682 (230.2)
SYN28307 (225.5)
SYN28307 (231.5)
PZE−102183282 (225.9)
PZE−102183282 (233.1)
PZE−102184023 (226.6)
PZE−102184023 (234.7)
PZE−102185494 (229.3)
PZE−102185494 (235.9)
PZE−102185951 (229.7)
PZE−102185951 (236.4)
PZE−102186619 (230.6)
PZE−102186619 (237)
PZE−102186665 (230.9)
PZE−102186665 (238)
PZE−102186688 (230.9)
PZE−102186688 (238.2)
PUT−163a−21390620−1531 (230.9)
PUT−163a−21390620−1531 (238.7)
PZE−102187332 (231.3)
PZE−102187332 (240.7)
PUT−163a−60353925−2728 (232.1)
PUT−163a−60353925−2728 (242.4)
PUT−163a−148959370−565 (232.6)
PUT−163a−148959370−565 (245)
PZE−102189370 (233)
PZE−102189370 (246)
PZE−102189664 (233.1)
PZE−102189664 (246.6)
PUT−163a−28910517−1641 (233.3)
PUT−163a−28910517−1641 (247.2)
SYN20256 (233.7)
SYN20256 (251.9)
PZE−102192367 (234.7)
PZE−102192367 (253.4)
PZE−102192857 (235.4)
SYN6914 (253.8)
PZE−102194043 (236.7)
PZE−102194043 (254.2)
SYN6914 (237)
PZE−102192857 (258.8)

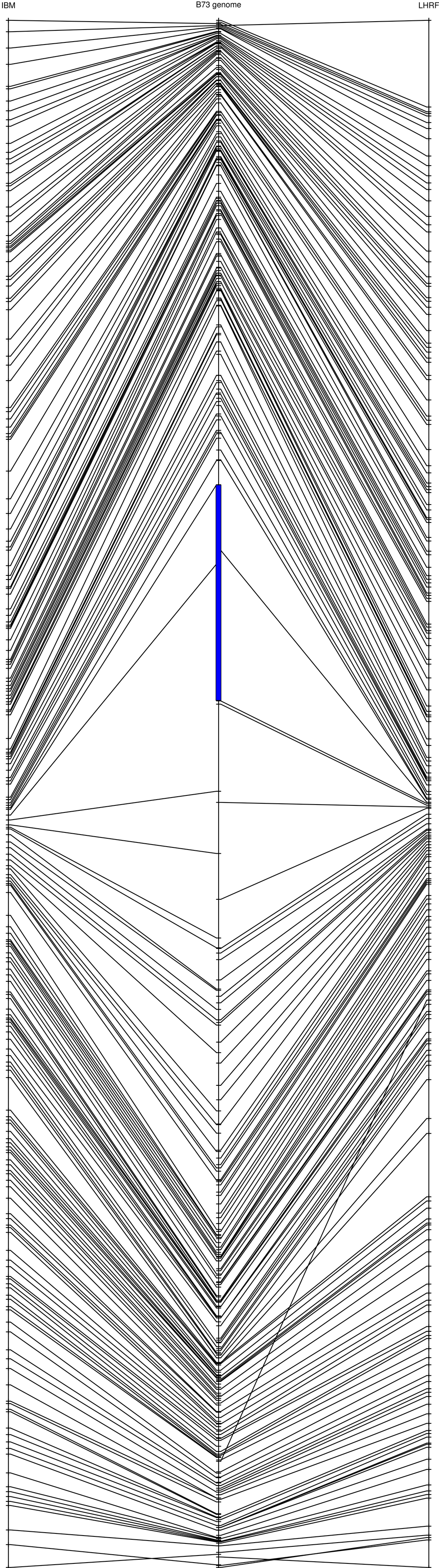
IBM
B73 genome
LHRF

Chrom 3

IBM map (cM)
B73 genome (Mbp)
PZA00309.1 (0) PZA00309.1 (1.3)
PZE−103001531 (1.6) PZE−103001531 (1.7)
PZE−103001968 (2.1) PZE−103001968 (2)
PZE−103002726 (4.4) PZE−103002726 (2.1)
SYN39150 (7.4) SYN39150 (2.4)
SYN10208 (10.7) SYN10208 (2.7)
SYN34566 (11) SYN34566 (3.1)
SYN25632 (11.9) SYN25632 (3.4)
SYN25624 (12.2) SYN25624 (3.5)
SYN15338 (19.1) SYN15338 (3.8)
PUT−163a−71423695−3220 (20.8) PUT−163a−71423695−3220 (4.4)
SYN5646 (23.8) SYN5646 (4.7)
PZE−103008604 (25.1) PZE−103008604 (4.7)
SYN5652 (25.9) SYN5652 (4.7)
SYN28478 (26.4) SYN28478 (4.9)
PZA02678.1 (28.8) PZA02678.1 (5.4)
SYN31006 (29.3) SYN31006 (5.6)
PUT−163a−148951348−515 (30.2) PUT−163a−148951348−515 (5.8)
SYN14580 (35.9) SYN14580 (7.4)
PZE−103014890 (36.5) PZE−103014890 (8)
PZE−103015226 (38.3) PZE−103015226 (8.2)
SYN13714 (42.3) SYN13714 (9)
SYN13710 (42.6) SYN13710 (9)
PZE−103016418 (43.4) PZE−103016418 (9.3)
SYN21251 (44.2) SYN21251 (9.4)
SYN10094 (46.9) SYN10094 (9.6)
SYN36244 (47.5) SYN36244 (10.1)
SYN24350 (48.4) SYN24350 (10.5)
SYN38181 (50.4) SYN38181 (10.7)
PZE−103019119 (51.5) PZE−103019119 (11.2)
SYN12567 (51.9) SYN12567 (12.1)
SYN12568 (52.2) SYN12568 (12.2)
PZE−103020815 (55.1) PZE−103020815 (12.9)
SYN37725 (57.4) SYN37725 (13.3)
SYN37354 (58.3) SYN37354 (13.7)
PZE−103022322 (59.2) PZE−103022322 (14.4)
SYN33443 (61.1) SYN33443 (15.2)
SYN33798 (62.4) SYN33798 (17.4)
PZE−103027358 (66.6) PZE−103027358 (20)
SYN15181 (67.1) SYN15181 (22.3)
PZE−103030210 (67.5) PZE−103030210 (22.6)
PZE−103030646 (68.5) PZE−103030646 (22.8)
PZE−103031418 (69.4) PZE−103031418 (23.4)
PZE−103031634 (69.8) PZE−103031634 (23.8)
PZE−103032163 (70.1) PZE−103032163 (24.9)
PZE−103034362 (70.4) PZE−103034362 (27.6)
SYN32857 (70.7) SYN32857 (28.6)
SYN32858 (71.4) SYN32858 (28.6)
PZE−103035766 (71.6) PZE−103035766 (29)
PZE−103036706 (71.9) PZE−103036706 (30.2)
PUT−163a−110204885−63 (72.6) PUT−163a−110204885−63 (31.9)
PZE−103037845 (73) PZE−103037845 (32.2)
SYN12770 (73.3) SYN12770 (35.4)
PZE−103041113 (73.6) PZE−103041113 (37.2)
PZE−103041880 (73.9) PZE−103041880 (38.6)
SYN18257 (74.2) SYN18257 (43.9)
PUT−163a−84969550−4460 (74.5) PUT−163a−84969550−4460 (47.8)
PZE−103046325 (74.8) PZE−103046325 (48.4)
SYN12902 (75.2) SYN12902 (49.4)
PZE−103049695 (75.4) PZE−103049695 (54.1)
PZE−103052045 (75.7) PZE−103052045 (58.1)
PZE−103053111 (75.9) PZE−103053111 (59.8)
PZE−103055698 (76.2) PZE−103055698 (66.9)
PZE−103066354 (76.5) SYN38653 (86.9)
PZE−103068012 (76.8) PZE−103068012 (87.5)
SYN38653 (77.1) PZE−103066354 (91.7)
PZE−103070590 (77.5) PZE−103070590 (114.9)
PZE−103072120 (77.8) PZE−103072120 (118.8)
PZE−103072900 (78.2) PZE−103072900 (120.3)
PZE−103074774 (78.6) PZE−103074774 (123.7)
PZE−103075826 (79.6) PZE−103075826 (125.7)
PZE−103076118 (80.5) PZE−103076118 (126.4)
PZE−103077634 (81.1) PZE−103077634 (128.6)
PZE−103077912 (81.3) PZE−103077912 (128.9)
PZE−103080303 (81.5) PZE−103080303 (133.2)
PZE−103081648 (81.9) PZE−103081648 (135.4)
PZE−103082609 (82.2) PZE−103082609 (136.9)
PZE−103085447 (82.6) PZE−103085447 (141.4)
PZE−103086783 (83.3) PZE−103086783 (143.8)
SYN10940 (85.8) SYN10940 (145.7)
SYN32915 (86.2) SYN32915 (146.6)
PZE−103089180 (86.5) PZE−103089180 (147.4)
SYN34785 (87.2) SYN34785 (149.2)
PZE−103090925 (88.7) PZE−103090925 (149.9)
PZE−103092754 (89.9) PZE−103092754 (153.8)
SYN20899 (90.2) SYN20899 (155)
PUT−163a−60398389−2973 (91.1) PUT−163a−60398389−2973 (157.1)
PZE−103099066 (93) PZE−103099066 (159.5)
SYN16048 (94.4) SYN16048 (160.5)
PZE−103102032 (97.8) PZE−103102032 (162.3)
PZE−103104368 (98.3) PZE−103104368 (165.9)
SYN14727 (98.9) SYN14727 (166)
PZE−103105074 (99.8) PZE−103105074 (166.3)
PUT−163a−76293601−3974 (100.3) PUT−163a−76293601−3974 (166.4)
PUT−163a−71759935−3438 (101.2) PUT−163a−71759935−3438 (167.7)
PZE−103109369 (103.1) PZE−103109369 (170.1)
SYN23410 (103.8) SYN23410 (170.4)
PZE−103110860 (104.4) PZE−103110860 (171)
SYN37788 (105) SYN37788 (171.9)
PZE−103112971 (105.8) PZE−103112971 (172.7)
SYN35210 (106.1) SYN35210 (173)
PZA03475.1 (106.4) PZA03475.1 (173.4)
SYN31233 (107.3) SYN31233 (173.6)
PZE−103116145 (108.8) PZE−103116145 (175.9)
PZE−103116979 (109.2) PZE−103116979 (176.3)
PZE−103118006 (110.8) PZE−103118006 (176.6)
SYN1546 (111.2) SYN1546 (176.8)
PZE−103118331 (111.5) PZE−103118331 (177)
SYN18160 (111.8) SYN18160 (177.5)
PZE−103119085 (112.6) PZE−103119085 (178)
PZE−103119687 (112.8) PZE−103119687 (178.3)
SYN28230 (113.2) SYN28230 (178.9)
PZE−103120924 (113.7) PZE−103120924 (179.2)
SYN30212 (115.4) SYN30212 (179.4)
SYN39105 (115.8) SYN39105 (179.5)
PZE−103122604 (116.3) PZE−103122604 (180.5)
SYN31222 (117.7) SYN31222 (181.1)
SYN31225 (118.7) SYN31225 (181.1)
PZE−103123698 (119.2) PZE−103123698 (181.5)
PZE−103123992 (119.6) PZE−103123992 (181.6)
PZE−103124901 (119.9) PZE−103124901 (182.1)
PZE−103125579 (121.9) PZE−103125579 (183.1)
PZE−103125803 (122.4) PZE−103125803 (183.4)
PZE−103126215 (123.8) PZE−103126215 (183.9)
SYN23233 (127) SYN23233 (184.6)
SYN10650 (127.8) SYN10650 (185.7)
PZE−103129454 (128.7) PZE−103129454 (186.2)
SYN36300 (129.5) SYN36300 (187)
PZE−103130978 (130.1) PZE−103130978 (187.4)
SYN13421 (130.5) SYN13421 (187.5)
PZE−103132150 (131.2) PZE−103132150 (188.2)
PZE−103132614 (132.3) PZE−103132614 (188.5)
PZE−103133568 (134.1) PZE−103133568 (189.5)
PZE−103134006 (134.7) PZE−103134006 (189.8)
SYN30268 (136.1) SYN30268 (190.8)
SYN6041 (136.8) SYN6041 (191.7)
SYN23092 (137.1) SYN23092 (192)
SYN20190 (137.4) SYN20190 (193.8)
SYN5792 (138) SYN5792 (194)
SYN24182 (138.6) SYN24182 (195.1)
SYN37387 (141) SYN37387 (195.9)
PZE−103141146 (141.5) PZE−103141146 (196.6)
PZE−103141953 (142.2) PZE−103141953 (197.4)
SYN34418 (143.8) SYN34418 (199.3)
PZE−103144468 (144.2) PZE−103144468 (199.6)
PUT−163a−88754120−4563 (145.1) PUT−163a−88754120−4563 (200.7)
PZE−103146632 (145.7) PZE−103146632 (201.2)
SYN32692 (147.9) SYN32692 (201.3)
SYN15015 (148.2) SYN15015 (202)
SYN15017 (148.5) SYN15017 (202)
PZE−103149272 (150.5) PZE−103149272 (203)
PZE−103150041 (151.8) PZE−103150041 (204.3)
PZE−103150482 (152.6) PZE−103150482 (204.6)
SYN20313 (152.9) SYN20313 (204.7)
PZE−103151399 (154.6) PZE−103151399 (205.3)
PUT−163a−71761183−3448 (155.6) PUT−163a−71761183−3448 (205.4)
SYN19676 (155.9) SYN19676 (206.6)
PZE−103155594 (158.3) PZE−103155594 (208.3)
PZE−103156977 (161.2) PZE−103156977 (209)
PZE−103157276 (162.1) PZE−103157276 (209.1)
SYN34635 (162.7) SYN34635 (209.7)
SYN36312 (163.1) SYN36312 (209.9)
SYN36308 (163.6) SYN36308 (210)
SYN31429 (165.3) SYN31429 (210.7)
SYN32032 (165.7) SYN32032 (211.7)
SYN1958 (165.9) SYN1958 (212)
PUT−163a−71440306−3328 (167.1) PUT−163a−71440306−3328 (212.2)
SYN31152 (167.3) SYN31152 (212.4)
PZE−103162577 (167.6) PZE−103162577 (212.8)
PZE−103163035 (168.7) PZE−103163035 (213.5)
PZE−103163529 (169) PZE−103163529 (213.7)
SYN36395 (169.3) SYN36395 (213.8)
SYN35424 (169.8) SYN35424 (214.3)
PZE−103164677 (170.1) PZE−103164677 (214.5)
SYN35556 (171.3) SYN35556 (214.8)
SYN36908 (171.7) SYN36908 (214.9)
SYN33391 (172.4) SYN33391 (215.5)
SYN31407 (172.8) SYN31407 (215.5)
PZE−103166902 (173.1) PZE−103166902 (215.9)
PUT−163a−22545298−1589 (173.4) PUT−163a−22545298−1589 (216)
SYN1293 (173.8) SYN1293 (217)
SYN484 (174.3) SYN484 (217.1)
SYN31522 (177.2) SYN31522 (217.6)
PZE−103169904 (177.9) PZE−103169904 (217.7)
SYN32258 (179.5) SYN32258 (218)
PZE−103171163 (180) PZE−103171163 (218.2)
SYN28000 (180.5) SYN28000 (218.9)
SYN4045 (181.6) SYN4045 (219.5)
PZE−103172732 (183.3) PZE−103172732 (219.6)
SYN31046 (185.9) SYN31046 (219.8)
PUT−163a−60355202−2755 (186.8) PUT−163a−60355202−2755 (220.9)
SYN38998 (187.1) SYN38998 (221.2)
SYN33527 (188.3) SYN33527 (221.5)
SYN33530 (188.9) SYN33530 (221.6)
SYN38130 (189.4) SYN38130 (222)
SYN34350 (189.7) SYN34350 (222.8)
SYN24268 (190.3) SYN24268 (223.1)
PZE−103177780 (190.6) PZE−103177780 (223.7)
PZE−103178060 (191.4) PZE−103178060 (223.8)
SYN31243 (193.4) SYN31243 (224.2)
PZE−103179078 (194) PZE−103179078 (224.5)
SYN27420 (195.1) SYN27420 (225.2)
PZE−103180492 (195.9) PZE−103180492 (225.4)
SYN30477 (196.4) SYN30477 (225.8)
SYN29679 (198.8) SYN29679 (226.8)
SYN29677 (199.6) SYN29677 (227)
PZE−103182291 (200.1) PZE−103182291 (227.1)
PZE−103182712 (200.8) PZE−103182712 (227.3)
SYN30342 (203.2) SYN30342 (228.4)
SYN37058 (204) SYN37058 (229.1)
SYN4860 (204.8) SYN4860 (229.3)
SYN32818 (205.7) SYN32818 (229.6)
PZE−103185889 (206.9) PZE−103185889 (230.3)
SYN38592 (207.7) SYN38592 (230.4)

B73 genome (Mbp)
LHRF map (cM)
SYN765 (2.1) SYN765 (0)
PUT−163a−74244282−3684 (2.4) PUT−163a−74244282−3684 (4.3)
SYNGENTA1720 (2.8) SYNGENTA1720 (6.3)
SYN34559 (3.1) SYN34559 (7.4)
SYN34622 (3.3) SYN34622 (8.4)
SYN25628 (3.5) SYN25628 (10.3)
PZE−103006731 (3.8) PZE−103006731 (13)
PUT−163a−71423695−3217 (4.4) PUT−163a−71423695−3217 (14.4)
SYN5652 (4.7) SYN5652 (16.2)
SYN28478 (4.9) SYN28478 (17.2)
PUT−163a−148951348−515 (5.8) PUT−163a−148951348−515 (20.1)
PZE−103011799 (6.3) PZE−103011799 (22.6)
PUT−163a−18172180−1378 (6.9) PUT−163a−18172180−1378 (29.7)
PZE−103014981 (8.1) PZE−103014981 (32.8)
SYN23644 (9.9) SYN23644 (37)
SYN7561 (10.3) SYN7561 (37.3)
PZE−103018378 (10.6) PZE−103018378 (38.1)
PZE−103018970 (11.2) PZE−103018970 (39.8)
PZE−103019841 (12.2) PZE−103019841 (40.4)
SYN37729 (13.5) SYN37729 (43.3)
PZE−103022026 (14) PZE−103022026 (44.3)
PZE−103022830 (15.1) PZE−103022830 (45)
SYN26605 (15.4) PZE−103023198 (45.4)
PZE−103023198 (15.5) SYN26605 (45.6)
PUT−163a−78120078−4386 (17.4) PUT−163a−78120078−4386 (46)
PZE−103024934 (17.6) PZE−103024934 (47.1)
PZE−103026250 (19.3) PZE−103026250 (48.6)
PZE−103027704 (20.4) PZE−103027704 (51)
PZE−103028869 (21.6) PZE−103028869 (52.8)
PZE−103029945 (22.4) PZE−103029945 (53.9)
PZE−103030646 (22.8) PZE−103030646 (56.3)
PZE−103031418 (23.4) PZE−103031418 (57.2)
PZE−103031593 (23.7) PZE−103031593 (57.8)
PZA02427.1 (24.4) PZA02427.1 (58.6)
PZE−103033486 (26.1) PZE−103033486 (59.6)
PZE−103035720 (29) PZE−103035720 (60.6)
SYN9228 (30) SYN9228 (61)
SYN11138 (31.4) SYN11138 (61.4)
PUT−163a−110204885−63 (31.9) PUT−163a−110204885−63 (62.5)
PZE−103039915 (35.6) PZE−103039915 (63.5)
PZE−103040656 (36.3) PZE−103040656 (63.8)
PZE−103041696 (38.2) PZE−103041696 (64.1)
SYN18838 (40.3) SYN18838 (64.3)
PZE−103042957 (41.2) PZE−103042957 (64.8)
PZE−103044836 (45.2) PUT−163a−74236155−3617 (65.5)
PUT−163a−74236155−3617 (47.4) PZE−103044836 (65.8)
PUT−163a−84969550−4459 (47.8) PUT−163a−84969550−4459 (66.6)
PZE−103047477 (50.1) PZE−103047477 (67.1)
PUT−163a−149008324−754 (50.6) PUT−163a−149008324−754 (67.9)
PUT−163a−21481350−1571 (54.7) PUT−163a−21481350−1571 (68.3)
PZE−103051068 (56.3) PZE−103051068 (68.5)
PZE−103051969 (58) PZE−103051969 (68.7)
PZE−103053111 (59.8) PZE−103053111 (69.5)
PZE−103056467 (68.8) PUT−163a−74245700−3707 (70)
PZE−103069166 (85.8) PZE−103056467 (70.7)
PZE−103068243 (86.9) SYN5048 (71.4)
SYN5048 (88.7) PZE−103068243 (71.8)
PUT−163a−74245700−3707 (92.5) PZE−103069166 (73)
PUT−163a−148941943−447 (121.1) PUT−163a−148941943−447 (74.3)
PZE−103075826 (125.7) PZE−103075826 (75.3)
PZE−103076834 (127.5) PZE−103076834 (76.1)
PUT−163a−71427473−3257 (129.1) PUT−163a−71427473−3257 (77.3)
PZA00295.6 (130) PZA00295.6 (78.1)
PZE−103082568 (136.8) PZE−103082568 (78.6)
PUT−163a−148941995−448 (140.7) PUT−163a−148941995−448 (79.1)
PZE−103087649 (145.5) PZE−103087649 (80)
SYN32914 (146.7) SYN32914 (80.3)
PZE−103088576 (146.7) PZE−103088576 (80.5)
PZB02044.2 (148.3) PZB02044.2 (82.1)
PZE−103091040 (150.1) PZE−103091040 (84)
PZE−103091351 (150.8) PZE−103091351 (84.5)
PZE−103091742 (151.6) PZE−103091742 (84.9)
ZM008266−0663 (153.3) PZE−103092646 (85.7)
PZE−103092646 (153.3) ZM008266−0663 (86.1)
PZE−103092933 (153.9) PZE−103092933 (86.8)
PZE−103093392 (154.6) PZE−103093392 (87.6)
PZE−103093699 (154.7) PZE−103094153 (87.9)
PZE−103094153 (155) PZE−103093699 (88.4)
PZE−103096408 (156.3) PZE−103096408 (90.2)
PZE−103096694 (156.5) PZE−103096694 (91)
PZE−103097269 (157) PZE−103097269 (91.5)
PUT−163a−148963330−592 (157.1) PUT−163a−148963330−592 (92.7)
PZD00037.1 (159) PZD00037.1 (93.7)
PZE−103100245 (160.5) PZE−103100245 (95.9)
PZE−103100890 (161.6) PZE−103100890 (96.5)
PZE−103102419 (162.7) PZE−103102712 (100.7)
PZE−103102712 (164.2) PZE−103102419 (101.4)
PUT−163a−16925568−1101 (165.7) PUT−163a−16925568−1101 (102.6)
PUT−163a−148995396−718 (167.5) PUT−163a−148995396−718 (104.6)
PZE−103108302 (169.1) PZE−103108302 (105.2)
PZE−103109054 (169.9) PZE−103109054 (105.8)
PZE−103109587 (170.2) PZE−103109587 (106.4)
PZD00027.2 (171.4) PZD00027.2 (108)
PZA03475.1 (173.4) PZA03475.1 (110)
PZE−103115334 (175.4) PZE−103115334 (111.1)
PZE−103116979 (176.3) PZE−103116979 (113.1)
PZE−103117610 (176.6) PZE−103117610 (114)
PUT−163a−4628221−2094 (176.7) PUT−163a−4628221−2094 (115)
SYN7263 (178) SYN7263 (116)
PZE−103119152 (178.1) PZE−103119152 (118.1)
PZE−103119655 (178.3) PZE−103119655 (118.8)
PZE−103120110 (178.6) PZE−103120110 (120.1)
PZE−103120779 (179.1) PZE−103120779 (120.8)
SYN7807 (179.8) SYN7807 (121.8)
PZE−103123019 (180.7) PZE−103123019 (123.5)
PZE−103124449 (181.8) PZE−103124449 (127.6)
PZE−103124738 (182) PZE−103124738 (128)
PZA03735.1 (182.3) PZA03735.1 (128.6)
PZE−103125173 (182.5) PZE−103125173 (129.5)
PZE−103125662 (183.3) PZE−103125662 (130.3)
PZE−103125892 (183.6) PZE−103125892 (131.5)
SYN23237 (184.6) SYN23237 (135.1)
PZE−103128864 (185.8) PZE−103128864 (135.9)
SYN4113 (186) SYN4113 (136.9)
PZA03648.2 (187) PZA03648.2 (137.9)
PZE−103132648 (188.5) PZE−103132648 (139.2)
PZE−103132991 (188.7) PZE−103132991 (139.9)
PZE−103136011 (191.4) PZE−103136011 (141.9)
SYN5788 (194) SYN5788 (143.8)
SYN25916 (195.7) SYN25916 (148.1)
PZE−103140361 (196.1) PZE−103140361 (150.5)
PZB01109.2 (196.4) PZB01109.2 (151.7)
PZE−103141032 (196.5) PZE−103141032 (152.6)
SYN38171 (199.1) PZE−103143575 (154)
PZE−103143575 (199.2) SYN38171 (154.7)
SYN33375 (199.6) SYN33375 (156.4)
PZE−103145494 (200.4) PZE−103145494 (156.9)
PUT−163a−78121045−4390 (200.7) PUT−163a−78121045−4390 (158.3)
SYN8109 (201.2) SYN8109 (158.7)
SYN32692 (201.3) SYN32692 (159)
PZE−103148470 (202.4) PZE−103148470 (160.1)
PZE−103148618 (202.6) PZE−103148618 (161.4)
PZE−103149074 (202.8) PZE−103149074 (162.3)
PZE−103150212 (204.5) PZE−103150212 (162.9)
SYN20313 (204.7) SYN20313 (163.6)
PUT−163a−148964486−613 (205.4) PUT−163a−148964486−613 (166.7)
PZE−103154386 (207.2) PZE−103154386 (167.8)
PUT−163a−24934942−1601 (208.3) PUT−163a−24934942−1601 (168.5)
SYN14789 (208.6) SYN14789 (169.6)
PZE−103156306 (208.8) PZE−103156306 (170.5)
PZE−103156977 (209) PZE−103156977 (172)
SYN28662 (209.6) SYN28662 (172.8)
SYN1557 (210.1) SYN1557 (173.1)
PZE−103159374 (210.8) PZE−103159374 (173.6)
PUT−163a−71440306−3328 (212.2) PUT−163a−71440306−3328 (176.5)
PZE−103162577 (212.8) PZE−103162577 (177.9)
PZE−103162726 (213.3) PZE−103162726 (178.5)
PZE−103163074 (213.5) PZE−103163074 (178.9)
SYN36395 (213.8) SYN36395 (179.2)
SYN36908 (214.9) SYN36908 (182)
PZE−103165697 (215.1) PZE−103165697 (182.6)
PUT−163a−71772697−3551 (215.7) PUT−163a−71772697−3551 (183.6)
PUT−163a−101387821−12 (216.3) PUT−163a−101387821−12 (185.7)
SYN484 (217.1) SYN484 (186.9)
SYN31522 (217.6) SYN31522 (188)
SYN31528 (217.7) SYN31528 (190)
PZE−103170973 (218.1) PZE−103170973 (192)
PZE−103171561 (218.8) PZE−103171561 (193)
PZE−103172406 (219.4) PZE−103172406 (194)
SYN30135 (219.7) SYN30135 (194.6)
PUT−163a−71331763−3193 (219.8) PUT−163a−71331763−3193 (195.3)
PZE−103175040 (221.3) PZE−103175040 (196.3)
PZE−103175677 (221.7) PZE−103175677 (198.3)
PZE−103176462 (222.4) PZE−103176462 (199.1)
PZE−103177256 (223.1) PZE−103177256 (200.3)
PZE−103178060 (223.8) PZE−103178060 (201.6)
PZE−103179078 (224.5) PZE−103179078 (204.6)
SYN27421 (225.2) SYN27421 (206.5)
PZE−103180492 (225.4) PZE−103180492 (207.8)
PZE−103180686 (225.6) PZE−103180686 (208.8)
SYN30480 (225.8) SYN30480 (209.3)
PZE−103181164 (226.3) PZE−103181164 (210)
PZE−103181733 (226.8) PZE−103181733 (211.8)
PZE−103181990 (227) PZE−103181990 (212.4)
PZE−103182399 (227.1) PZE−103182399 (213)
PZE−103182717 (227.3) PZE−103182717 (214.7)
SYN24589 (227.5) SYN24589 (215.3)
PZE−103183225 (227.6) PZE−103183225 (215.6)
SYN30342 (228.4) PZE−103183711 (216.3)
PZE−103183711 (228.4) SYN30342 (216.6)
PZE−103184603 (229.4) PZE−103184603 (217.5)
PZE−103185158 (229.7) PZE−103185158 (218.8)
SYN1576 (230) SYN1576 (220.4)
SYN28995 (230.3) SYN28995 (221)
PUT−163a−111191936−146 (230.5) PUT−163a−111191936−146 (221.9)
PZE−103186202 (230.5) PZE−103186202 (223.6)
PZE−103186797 (231) PZE−103186797 (224.5)

IBM
B73 genome
LHRF

Chrom 4

IBM map (cM)	B73 genome (Mbp)
SYN33196 (0)	SYN33196 (0.8)
SYN24156 (1.4)	SYN24156 (1.3)
PZE−104001654 (1.7)	PZE−104001654 (2)
SYN29285 (1.9)	SYN29285 (2.1)
SYN33006 (2.5)	SYN33006 (2.4)
SYN27469 (2.8)	SYN27469 (2.5)
PUT−163a−78115415−4344 (3.5)	PUT−163a−78115415−4344 (2.5)
SYN22929 (4.6)	SYN22929 (2.7)
PUT−163a−94477564−4893 (7.2)	PUT−163a−94477564−4893 (3.1)
SYN1073 (10.3)	SYN1073 (3.5)
SYN1064 (13.7)	SYN1064 (3.5)
SYN24520 (15.5)	SYN24520 (3.7)
SYN11366 (17.7)	SYN11366 (4.2)
SYN24044 (18.7)	SYN24044 (4.6)
SYN36763 (19.2)	SYN36763 (4.9)
SYN29175 (24.5)	SYN29175 (5)
SYN29997 (27.2)	SYN29997 (5.3)
SYN30003 (28.2)	SYN30003 (5.3)
PZE−104009755 (35.1)	PZE−104009755 (6.6)
PZE−104010129 (36)	PZE−104010129 (7.2)
SYN19801 (36.6)	SYN19801 (7.3)
SYN11694 (37)	SYN11694 (9.7)
SYN11697 (37.7)	SYN11697 (9.8)
SYN14191 (39.4)	SYN14191 (10)
SYN31962 (41.2)	SYN31962 (10.2)
PZE−104012818 (44.2)	PZE−104012818 (11.1)
PZE−104013117 (45.8)	PZE−104013117 (11.3)
PZE−104013627 (48.8)	PZE−104013627 (11.9)
PZE−104014765 (50.8)	PZE−104014765 (14)
SYN12142 (52.4)	SYN12142 (14.5)
PZE−104015507 (53.2)	PZE−104015507 (14.9)
PZE−104016428 (53.7)	PZE−104016428 (16.2)
PZE−104016794 (54.2)	PZE−104016794 (16.6)
SYN30707 (55.3)	SYN30707 (17.4)
PZE−104017875 (56.4)	PZE−104017875 (17.7)
PZE−104018218 (57.5)	PZE−104018218 (18)
PZE−104018363 (58.9)	PZE−104018363 (18.2)
PUT−163a−29945048−1803 (59.4)	PUT−163a−29945048−1803 (18.2)
SYN1498 (59.8)	SYN1498 (18.6)
PZE−104019012 (60)	PZE−104019012 (19.2)
SYN1513 (60.3)	SYN1513 (20.1)
SYN33996 (60.6)	SYN33996 (20.4)
SYN24026 (61.2)	SYN24026 (20.7)
SYN23842 (62.6)	SYN23842 (21.9)
PZE−104021262 (63.5)	PZE−104021262 (22.7)
PZE−104021510 (64.1)	PZE−104021510 (23.1)
PZE−104022099 (64.6)	PZE−104022099 (23.9)
SYN31169 (65.8)	SYN31169 (26.4)
PZE−104023818 (66.3)	PZE−104023818 (26.9)
SYN23037 (66.8)	SYN23037 (30.7)
PUT−163a−60345084−2531 (67.7)	PUT−163a−60345084−2531 (33.4)
PZE−104028286 (68.3)	PZE−104028286 (34.1)
PZE−104029141 (69.3)	PZE−104029141 (35.2)
SYN33264 (69.8)	SYN33264 (36)
SYN25839 (70.6)	SYN25839 (37)
PUT−163a−89249187−4625 (71.2)	PUT−163a−89249187−4625 (37)
PZE−104030980 (71.4)	PZE−104030980 (37.3)
PZE−104031664 (71.7)	PZE−104031664 (38.3)
SYN4887 (72)	SYN4887 (39.3)
PZE−104032365 (72.2)	PZE−104032365 (39.5)
PZE−104033664 (72.7)	PZE−104033664 (42.1)
PZE−104035115 (73.4)	PZE−104035115 (44.6)
SYN10441 (73.7)	SYN10441 (49.4)
PZE−104042023 (74.1)	PZE−104042023 (57.9)
PZE−104042143 (74.9)	PZE−104042143 (58)
SYN1347 (75.4)	SYN1347 (72.9)
SYN39139 (75.7)	SYN39139 (74.6)
PZE−104049103 (75.9)	PZE−104049103 (75.5)
PZE−104052099 (76.2)	PZE−104052099 (82.6)
SYN21348 (77.2)	SYN21348 (91.4)
PZE−104061623 (77.9)	PZE−104061623 (121.4)
PZE−104066797 (78.4)	PZE−104066797 (131.9)
SYN25677 (78.9)	SYN25677 (144)
PZE−104074935 (79.4)	PZE−104074935 (147)
SYN11724 (79.9)	SYN11724 (151.5)
PHM15864.8 (80.3)	PHM15864.8 (151.5)
SYN27217 (81.6)	SYN27217 (151.7)
PZE−104078143 (82.5)	PZE−104078143 (152.4)
PZE−104079202 (83.1)	PZE−104079202 (153.5)
PUT−163a−71330910−3189 (85.1)	PUT−163a−71330910−3189 (154.4)
PZE−104080521 (85.6)	PZE−104080521 (155)
SYN4392 (86)	SYN4392 (155.2)
PZE−104081530 (86.7)	PZE−104081530 (155.9)
SYN2340 (87.1)	SYN2340 (157)
PZE−104083693 (88.1)	PZE−104083693 (157.6)
SYN4676 (90.1)	SYN4676 (158.1)
SYN5595 (90.6)	SYN5595 (159.7)
SYN2192 (90.8)	SYN2192 (160)
PZE−104086186 (91.1)	PZE−104086186 (160.3)
SYN1918 (91.6)	SYN1918 (160.6)
PZE−104087964 (92.5)	PZE−104087964 (162.9)
PZE−104088728 (93)	PZE−104088728 (163.8)
SYN1892 (95.8)	SYN1892 (166.9)
PZE−104091530 (97.6)	PZE−104091530 (167.1)
SYN2178 (98.7)	SYN2178 (170.8)
SYN606 (100.4)	SYN606 (171.1)
SYN2151 (100.7)	SYN2151 (171.7)
PZE−104095248 (101.6)	PZE−104095248 (171.8)
PZA00271.1 (103.9)	PZA00271.1 (172.5)
PZE−104096416 (104.7)	PZE−104096416 (172.8)
SYN2090 (105)	SYN2090 (173.4)
SYN2452 (106.4)	SYN2452 (173.9)
PUT−163a−4624412−2091 (107.2)	PUT−163a−4624412−2091 (174.9)
SYN2327 (109.6)	SYN2327 (177.1)
SYN2328 (110.3)	SYN2328 (177.1)
PZE−104100589 (110.7)	PZE−104100589 (177.3)
PZE−104100716 (111)	PZE−104100716 (177.4)
SYN18331 (112.1)	SYN18331 (178.5)
PZE−104102755 (114.9)	PZE−104102755 (178.8)
PZE−104103558 (115.3)	PZE−104103558 (179.8)
SYN15395 (115.5)	SYN15395 (180.2)
SYN1846 (117.1)	SYN1846 (180.5)
SYN2308 (117.8)	SYN2308 (181.1)
PZE−104104798 (118.2)	PZE−104104798 (181.1)
PZA02194.1 (118.7)	PZA02194.1 (181.2)
SYN1197 (119.2)	SYN1197 (181.3)
SYN5704 (119.7)	SYN5704 (181.7)
PZE−104105589 (120.1)	PZE−104105589 (181.9)
PZE−104105816 (120.4)	PZE−104105816 (181.9)
PZE−104105851 (121.1)	PZE−104105851 (182)
PZE−104106033 (121.4)	PZE−104106033 (182.1)
SYN25794 (121.9)	SYN25794 (183.2)
SYN34550 (122.3)	SYN34550 (184.8)
PUT−163a−13388743−219 (123.4)	PUT−163a−13388743−219 (185.5)
PZE−104109358 (124.8)	PZE−104109358 (185.7)
SYN3156 (125.2)	SYN3156 (185.9)
PZE−104110312 (125.7)	PZE−104110312 (186.6)
SYN32511 (125.9)	SYN32511 (186.7)
PZE−104110698 (126.2)	PZE−104110698 (186.8)
PZE−104112738 (127.3)	PZE−104112738 (188)
PZE−104112784 (127.9)	PZE−104112784 (188.1)
PZE−104113360 (128.4)	PZE−104113360 (188.5)
PZE−104114276 (128.8)	PZE−104114276 (189.8)
PZE−104114687 (129.1)	PZE−104114687 (190.3)
SYN14892 (129.9)	SYN14892 (191.1)
PHM4348.16 (131.2)	PHM4348.16 (191.8)
PZE−104116352 (131.5)	PZE−104116352 (192.2)
SYN35555 (131.8)	SYN35555 (193.2)
SYN5656 (132)	SYN5656 (194.2)
SYN1805 (132.2)	SYN1805 (195)
SYN38521 (132.7)	SYN38521 (198.1)
PZE−104122100 (133.3)	PZE−104122100 (199.1)
PZE−104122677 (133.7)	PZE−104122677 (199.9)
PZE−104123695 (134.3)	PZE−104123695 (200.8)
PZE−104123974 (134.9)	PZE−104123974 (201.2)
PZE−104126171 (135.2)	PZE−104126171 (204.2)
PZE−104126314 (135.5)	PZE−104126314 (204.3)
PZE−104126813 (136)	PZE−104126813 (205.1)
PZE−104128889 (136.8)	PZE−104128889 (209.3)
SYN34761 (137)	SYN34761 (210.1)
SYN2000 (137.4)	SYN2000 (212.9)
PZE−104132640 (138.6)	PZE−104132640 (216)
PZE−104133002 (139)	PZE−104133002 (216.4)
PUT−163a−149109388−951 (139.3)	PUT−163a−149109388−951 (217)
SYN29124 (139.7)	SYN29124 (219.5)
SYN19608 (140.7)	SYN19608 (224.2)
PZE−104137686 (141.6)	PZE−104137686 (224.6)
SYN22426 (141.9)	SYN22426 (224.9)
SYN18860 (142.1)	SYN18860 (225.7)
PZE−104139549 (143.4)	PZE−104139549 (227.2)
PZE−104139996 (143.9)	PZE−104139996 (228)
PZE−104140188 (144.8)	PZE−104140188 (228.6)
SYN17405 (145.1)	SYN17405 (228.6)
SYN12608 (145.4)	SYN12608 (229)
PZE−104141070 (145.7)	PZE−104141070 (229.4)
SYN29804 (146)	SYN29804 (230.2)
SYN22806 (146.3)	SYN22806 (230.4)
SYN1686 (146.5)	SYN1686 (231.1)
SYN29112 (146.8)	SYN29112 (231.4)
PZE−104142983 (147.2)	PZE−104142983 (231.5)
PZE−104143118 (148.3)	PZE−104143118 (231.6)
SYN23347 (149.1)	SYN23347 (233.2)
SYN23418 (149.6)	SYN23418 (233.8)
SYN22075 (150.4)	SYN22075 (234.2)
PZE−104145773 (151.7)	PZE−104145773 (234.4)
PZE−104146082 (153.8)	PZE−104146082 (234.4)
SYN28905 (154.5)	SYN28905 (234.6)
SYN19392 (155.2)	SYN19392 (234.7)
PZE−104146999 (155.5)	PZE−104146999 (234.9)
SYN1161 (156.1)	SYN1161 (235.3)
SYN28888 (157.7)	SYN28888 (235.7)
SYN23993 (158.3)	SYN23993 (236.1)
PZE−104150001 (162.7)	PZE−104150001 (236.6)
SYN14093 (163.7)	SYN14093 (236.9)
SYN39090 (167.1)	SYN39090 (237.3)
SYN14623 (169)	SYN14623 (237.6)
PZE−104152652 (169.9)	PZE−104152652 (237.7)
SYN37134 (170.5)	SYN37134 (238.1)
PUT−163a−31664272−1986 (171.5)	PUT−163a−31664272−1986 (238.2)
PZE−104153704 (172.9)	PZE−104153704 (238.3)
PZE−104154015 (173.2)	PZE−104154015 (238.4)
SYN1363 (174.1)	SYN1363 (238.5)
SYN38841 (175.5)	SYN38841 (238.7)
PZE−104154549 (175.8)	PZE−104154549 (238.8)
SYN37256 (176.6)	SYN37256 (239)
SYN26000 (178.1)	SYN26000 (239.1)
PZA02239.11 (178.9)	PZA02239.11 (239.1)
PZE−104155284 (179.3)	PZE−104155284 (239.2)
SYN39224 (180.4)	SYN39224 (239.4)
SYN29056 (181.1)	SYN29056 (239.4)
SYN29058 (181.4)	SYN29058 (239.4)
SYN16460 (181.7)	SYN16460 (239.5)
SYN16468 (182.1)	SYN16468 (239.7)
PZE−104157101 (186)	PZE−104157101 (240.1)

B73 genome (Mbp)	LHRF map (cM)
PZE−104005416 (1.2)	PZE−104005416 (0)
SYN29285 (2.1)	SYN29285 (3.7)
PZE−104002805 (2.7)	PZE−104002805 (6.6)
PZE−104003542 (3)	PZE−104003542 (9.6)
PUT−163a−148946720−494 (3.3)	PUT−163a−148946720−494 (11)
PZE−104004045 (3.4)	PZE−104004045 (11.5)
PZE−104004499 (3.6)	PZE−104004499 (12.4)
PZE−104004875 (3.9)	PZE−104004875 (15.2)
PUT−163a−60338561−2357 (4.8)	PUT−163a−60338561−2357 (17.7)
SYN29175 (5)	SYN29175 (19.6)
PZE−104007604 (5.2)	PZE−104007604 (20.6)
PZE−104008253 (5.5)	PZE−104008253 (23.3)
PZE−104009755 (6.6)	PZE−104009755 (27.9)
PZE−104009983 (7)	PZE−104009983 (28.9)
PZE−104010326 (7.4)	PZE−104010326 (30.2)
PZE−104011306 (9.9)	PZE−104011306 (34.3)
PZE−104012490 (10.8)	PZE−104012490 (38.1)
PZE−104012758 (10.9)	PZE−104012758 (39)
PZE−104013117 (11.3)	PZE−104013117 (41.4)
PZE−104014100 (12.9)	PZE−104014100 (43.7)
PZE−104015435 (14.8)	PZE−104015435 (47.9)
PZE−104015507 (14.9)	PZE−104015507 (48.6)
SYN13987 (15.3)	SYN13987 (49.3)
PZE−104016717 (16.5)	PZE−104016717 (50.3)
PZE−104017733 (17.5)	PZE−104017733 (52)
PZE−104018179 (18)	PZE−104018179 (53)
PZE−104018347 (18.2)	PZE−104018347 (53.5)
PUT−163a−18163635−1256 (18.7)	PUT−163a−18163635−1256 (54.2)
MZB00125.2 (21.1)	MZB00125.2 (55.2)
PZE−104020214 (21.6)	PZE−104020214 (56.1)
PUT−163a−78122682−4405 (24.9)	PUT−163a−78122682−4405 (59.2)
SYN31169 (26.4)	SYN31169 (60.4)
PZE−104023674 (26.7)	PZE−104023674 (61)
SYN34840 (31.1)	SYN34840 (61.7)
PZE−104026739 (31.6)	PZE−104026739 (64.7)
PZE−104027630 (32.8)	PZE−104027630 (66.2)
PZE−104030935 (37.3)	PZE−104030935 (67.7)
PZE−104031374 (38)	PZE−104031374 (68.7)
PZE−104032675 (39.8)	PZE−104032675 (69)
PHM14717.2 (40.7)	PHM14717.2 (69.2)
PZE−104033608 (42.1)	PZE−104033608 (69.6)
PZE−104033928 (42.4)	PZE−104033928 (69.9)
PZE−104035236 (44.8)	PUT−163a−76013805−3742 (70.3)
PUT−163a−76013805−3742 (45.3)	PZE−104035236 (70.5)
PZE−104040670 (54.9)	PZE−104040670 (71.2)
PZE−104042143 (58)	PZE−104042143 (71.7)
PZE−104043078 (61.4)	PZE−104043078 (72.4)
PZE−104043371 (62.3)	PZE−104043371 (72.7)
SYN1615 (72.1)	SYN1615 (73.6)
PZE−104047736 (72.6)	PZE−104047736 (74)
PZE−104048738 (74.7)	PZE−104048738 (74.8)
PZE−104049598 (76.7)	PZE−104049598 (75.6)
PZE−104052431 (83.3)	PZE−104052431 (77.5)
PZE−104052618 (83.6)	PZE−104052618 (79.6)
PZE−104112105 (103.1)	PZE−104112105 (81.1)
PZE−104062009 (122.2)	PZE−104062009 (81.6)
PZE−104064763 (127.9)	PZE−104064763 (81.9)
fea2.3 (133.7)	fea2.3 (82.4)
PZE−104071373 (142.3)	PZE−104071373 (82.8)
PZE−104072065 (143.2)	PZE−104072065 (83.5)
SYN19887 (145.6)	SYN19887 (84.4)
PZE−104075026 (147.1)	PZE−104075026 (86.5)
PZE−104075958 (149.7)	PZE−104075958 (88.2)
PZE−104077152 (151.7)	PZE−104077152 (89)
PZE−104077682 (152.1)	PZE−104077682 (90.2)
PZE−104078566 (152.8)	PZE−104078566 (92.2)
PZE−104079202 (153.5)	PZE−104079202 (92.6)
PZE−104079829 (154.2)	PZE−104079829 (93.5)
SYN23565 (154.9)	SYN23565 (94.2)
PZE−104080906 (155.3)	PZE−104080906 (94.9)
PZE−104081806 (156.2)	PZE−104081806 (97.4)
PZE−104082002 (156.4)	PZE−104082002 (97.9)
PZB00092.1 (157.2)	PZB00092.1 (99.8)
PZE−104083501 (157.5)	PZE−104083501 (100.6)
PUT−163a−31558543−1963 (158.5)	PUT−163a−31558543−1963 (102.6)
PZE−104086003 (160.3)	PZE−104086003 (105.9)
PZE−104086726 (161.1)	PZE−104086726 (110.3)
PZE−104087328 (162.1)	PZE−104087328 (111.3)
PUT−163a−76926256−4034 (162.9)	PUT−163a−76926256−4034 (113)
PZE−104088422 (163.4)	PZE−104088422 (113.7)
SYN2280 (165.6)	SYN2280 (114.6)
PZE−104090353 (166)	PZE−104090353 (115.2)
SYN1892 (166.9)	SYN1892 (115.9)
PUT−163a−18170189−1352 (167.1)	PUT−163a−18170189−1352 (117.3)
SYN23101 (167.2)	SYN23101 (117.7)
PZE−104092771 (168.8)	PZE−104092771 (118.1)
PZE−104093384 (170)	PZE−104093384 (119)
PZE−104094170 (171)	PZE−104094170 (120.2)
PZE−104094288 (171.1)	PZE−104094288 (120.8)
PZE−104095809 (172.1)	PZE−104095809 (125.2)
SYN2251 (173.2)	SYN2251 (125.9)
PZE−104096826 (173.3)	PZE−104096826 (126.5)
SYN18772 (173.6)	PUT−163a−30032190−1853 (127.7)
PUT−163a−30032190−1853 (173.8)	SYN18772 (128)
PZE−104098648 (175.1)	PZE−104098648 (128.7)
SYN2321 (177.1)	SYN2321 (129.4)
SYN2318 (177.2)	SYN2318 (129.9)
PZE−104101753 (178.4)	PZE−104101753 (133.8)
PUT−163a−60396647−2946 (179.6)	PUT−163a−60396647−2946 (138)
PZE−104103711 (180)	PZE−104103711 (138.8)
PZE−104104390 (180.6)	PZE−104104390 (139.6)
SYN2310 (181.1)	SYN2310 (140.7)
SYN5704 (181.7)	SYN5704 (141.8)
PZE−104105816 (181.9)	PZE−104105816 (143)
SYNGENTA6272 (182)	SYNGENTA6272 (143.8)
PZE−104106315 (182.6)	PZE−104106315 (144.4)
SYN25794 (183.2)	SYN25794 (144.9)
SYN34550 (184.8)	SYN34550 (145.9)
PZE−104108388 (185.1)	PZE−104108388 (146.4)
PZE−104109167 (185.7)	PZE−104109167 (147.1)
PZE−104109358 (185.7)	PZE−104109358 (148.5)
PZE−104110006 (186.2)	PZE−104110006 (149.3)
PUT−163a−78083703−4166 (186.5)	PUT−163a−78083703−4166 (149.8)
PZE−104112313 (187.3)	PZE−104112313 (150.2)
PZE−104112738 (188)	PZE−104112738 (150.7)
PZE−104113793 (189.1)	PZE−104113793 (152.2)
SYNGENTA2296 (189.3)	SYNGENTA2296 (153.4)
SYN14363 (190.9)	SYN14363 (154.3)
PZE−104115252 (191)	PZE−104115252 (154.6)
PZE−104115471 (191.2)	PZE−104115471 (155.2)
PZA00193.1 (192.2)	PZA00193.1 (157.6)
PZE−104118322 (195)	PZE−104118322 (158.2)
PZA01810.2 (198.1)	PZA01810.2 (159.9)
PZE−104121726 (198.8)	PZE−104121726 (160.4)
PZE−104123291 (200.3)	PZE−104123291 (162.4)
PUT−163a−149075652−845 (204.3)	PUT−163a−149075652−845 (163.2)
SYN36940 (204.6)	SYN36940 (163.5)
PZE−104126579 (204.7)	PZE−104126579 (163.7)
PZE−104127102 (206)	PZE−104127102 (164.3)
PZE−104127249 (206.1)	PZE−104127249 (164.6)
PUT−163a−76020873−3816 (207.4)	PUT−163a−76020873−3816 (165.1)
PZE−104128813 (209.2)	PZE−104128813 (165.9)
PZE−104128792 (209.2)	PZE−104128792 (166.4)
PZA03081.9 (209.8)	PZA03081.9 (167.2)
SYN34761 (210.1)	SYN34761 (167.8)
PZE−104130207 (211.1)	PZE−104130207 (168.3)
SYN1276 (212.2)	SYN1276 (168.6)
PUT−163a−60348862−2597 (214.9)	PUT−163a−60348862−2597 (169.6)
PZE−104132368 (215.4)	PZE−104132368 (170.2)
PZA03155.3 (216.6)	PZA03155.3 (171.1)
PZE−104134329 (218.4)	PZE−104134329 (172.4)
PZE−104135135 (219.7)	PZE−104135135 (172.9)
PZE−104136278 (222)	PZE−104136278 (174.1)
PUT−163a−74232765−3576 (224)	PUT−163a−74232765−3576 (175.3)
PZE−104137781 (224.7)	PZE−104137781 (176.4)
PZE−104139266 (226.7)	PZE−104139266 (178.1)
PZA00521.3 (229.2)	PZA00521.3 (179)
PZE−104141424 (229.8)	PZE−104141424 (180.2)
PUT−163a−60354034−2733 (231.5)	PUT−163a−60354034−2733 (181.5)
SYN11080 (232.9)	SYN11080 (184.7)
SYN23338 (233.3)	SYN23338 (186)
PUT−163a−78082892−4160 (233.8)	PUT−163a−78082892−4160 (186.9)
PZE−104145773 (234.4)	PZE−104145773 (188.1)
PZE−104146750 (234.8)	PZE−104146750 (190.4)
PZE−104146999 (234.9)	PZE−104146999 (194.4)
PUT−163a−149095258−897 (235.4)	PUT−163a−149095258−897 (196.3)
PZE−104148975 (236.1)	PZE−104148975 (197.6)
SYN24015 (236.2)	SYN24015 (198.1)
PZE−104149728 (236.4)	PZE−104149728 (198.7)
SYN14093 (236.9)	SYN14093 (201.3)
PZA00005.2 (237.4)	PZA00005.2 (203)
SYN14618 (237.6)	SYN14618 (205.4)
SYN7825 (238.3)	SYN7825 (207.6)
PZE−104154700 (239)	PZE−104154700 (209)
PZE−104155312 (239.2)	PZE−104155312 (211.5)
SYN29056 (239.4)	SYN29056 (213.1)
SYN16446 (239.5)	SYN16446 (214.5)
PZE−104156611 (239.8)	PZE−104156611 (216)
PZE−104157368 (240.2)	PZE−104157368 (218.6)
PZE−104157695 (240.5)	PZE−104157695 (220.3)

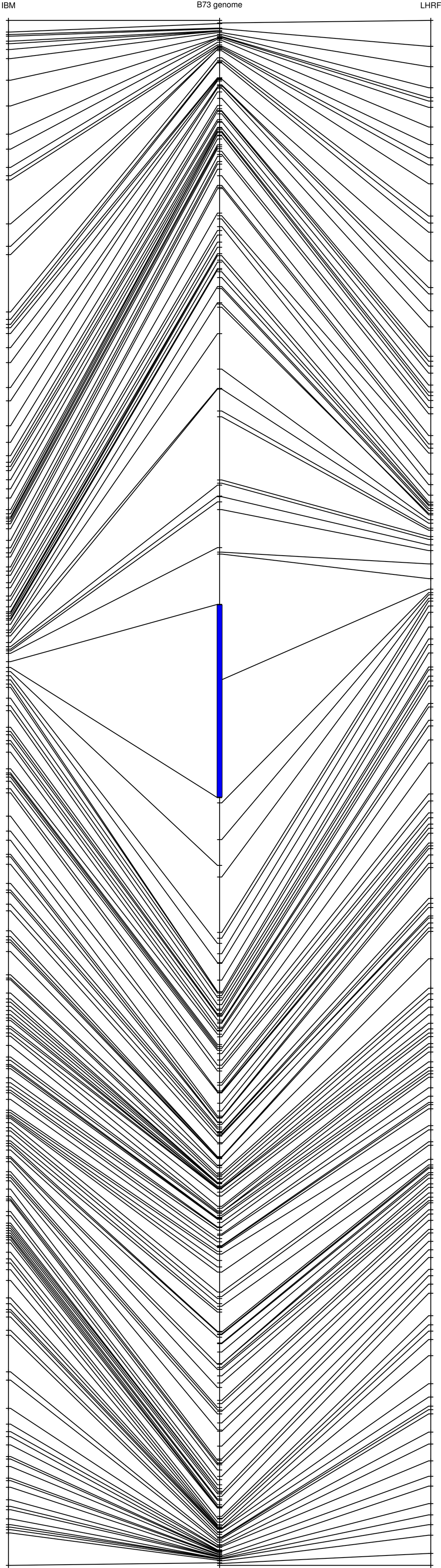

Chrom 5

IBM map (cM)	B73 genome (Mbp)
PZE−105000145 (0)	PZE−105000145 (0.4)
SYN21612 (0.9)	SYN21612 (1.5)
SYN21619 (2.1)	SYN21619 (1.6)
SYN2372 (2.3)	SYN2372 (1.7)
SYN14900 (2.7)	SYN14900 (1.8)
SYN35027 (3.1)	SYN35027 (1.9)
SYN35353 (4.1)	SYN35353 (2.1)
PZE−105003814 (4.5)	PZE−105003814 (2.3)
SYN33739 (5.3)	SYN33739 (2.3)
SYN36933 (6.7)	SYN36933 (2.4)
SYN15176 (8.1)	SYN15176 (2.7)
SYN15172 (8.5)	SYN15172 (2.7)
SYN541 (9.1)	SYN541 (2.8)
SYN14475 (9.9)	SYN14475 (2.9)
SYN31758 (11.8)	SYN31758 (3.4)
SYN523 (13.4)	SYN523 (4.3)
PUT−163a−14244370−320 (15.3)	PUT−163a−14244370−320 (4.3)
PZE−105009736 (15.7)	PZE−105009736 (4.5)
SYN5363 (18.3)	SYN5363 (4.8)
SYN30873 (19.5)	SYN30873 (5.2)
PZE−105012181 (20.3)	PZE−105012181 (5.3)
SYN12682 (21.2)	SYN12682 (5.4)
SYN12678 (21.5)	SYN12678 (5.4)
SYN25466 (22.1)	SYN25466 (5.6)
SYN1037 (22.7)	SYN1037 (5.9)
SYN3123 (24.1)	SYN3123 (5.9)
PZE−105015473 (30.1)	PZE−105015473 (6.6)
PZE−105016045 (30.8)	PZE−105016045 (7)
PZE−105016573 (31.3)	PZE−105016573 (7.3)
SYN2126 (32.5)	SYN2126 (7.3)
SYN28825 (33.1)	SYN28825 (7.4)
PZE−105017551 (33.3)	PZE−105017551 (7.7)
PZE−105018063 (33.9)	PZE−105018063 (8)
PZE−105018841 (35.6)	PZE−105018841 (8.5)
SYN23472 (35.9)	SYN23472 (8.6)
SYN33205 (37.3)	SYN33205 (9.7)
SYN38234 (38.7)	SYN38234 (10.1)
PZE−105021560 (40.1)	PZE−105021560 (10.2)
PZE−105022328 (40.6)	PZE−105022328 (10.9)
PZE−105023645 (42.1)	PZE−105023645 (11.9)
SYN27310 (46.8)	SYN27310 (12.2)
SYN35808 (48.1)	SYN35808 (13.1)
PZE−105025787 (48.8)	PZE−105025787 (13.2)
SYN1692 (49.4)	SYN1692 (13.4)
PZE−105026971 (49.8)	PZE−105026971 (13.8)
SYN19687 (50.5)	SYN19687 (13.8)
SYN16500 (54.2)	SYN16500 (15.1)
SYN16499 (54.6)	SYN16499 (15.2)
SYN1412 (54.9)	SYN1412 (15.3)
SYN15152 (55.3)	SYN15152 (15.7)
PZE−105030600 (55.8)	PZE−105030600 (16.2)
PZE−105031086 (56.6)	PZE−105031086 (16.7)
PZE−105031503 (57.2)	PZE−105031503 (17.1)
PZE−105031844 (57.8)	PZE−105031696 (17.2)
PZE−105031696 (58)	PZE−105031844 (17.3)
PZE−105033111 (58.8)	PZE−105033111 (18.4)
SYN21200 (59.6)	SYN21200 (18.7)
SYN21201 (60.1)	SYN21201 (18.7)
PUT−163a−88752620−4549 (60.6)	PUT−163a−88752620−4549 (20.8)
PZE−105036629 (60.9)	PZE−105036629 (21.4)
PZE−105037660 (61.2)	PZE−105037660 (22.6)
SYN27691 (61.7)	SYN27691 (23)
PZE−105040234 (62.2)	PZE−105040234 (25.6)
SYN17126 (62.4)	PZE−105041019 (27.1)
PZE−105041019 (62.6)	SYN17126 (27.1)
SYN32049 (62.9)	SYN32049 (28.3)
PZE−105042963 (63.2)	PZE−105042963 (30.3)
SYN26667 (63.5)	SYN26667 (31.6)
PZE−105044425 (64)	PZE−105044425 (31.9)
SYN11795 (64.7)	SYN11795 (32.9)
PZE−105048851 (65.2)	PZE−105048851 (39.7)
PZE−105049996 (66.3)	PZE−105049996 (42.3)
PZE−105050537 (67.5)	PZE−105050537 (43.1)
PZE−105050809 (68.3)	PZE−105050809 (43.7)
PZE−105053747 (68.5)	PZE−105053747 (49.8)
PZE−105055665 (69.1)	PZE−105055665 (53.4)
PZE−105057600 (69.5)	PZE−105057600 (56.2)
SYN38516 (69.8)	SYN38516 (57.4)
SYN24320 (70.2)	SYN24320 (58.8)
PZE−105061350 (70.7)	PZE−105061350 (60.1)
PZE−105065011 (71.3)	PZE−105065011 (66)
PZE−105065758 (72)	PZE−105065758 (67.5)
PZE−105066407 (72.6)	PZE−105066407 (68.4)
SYN28088 (72.9)	SYN28088 (68.7)
PZE−105067125 (73.2)	PZE−105067125 (69.3)
PZE−105069405 (73.4)	PZE−105069405 (73.1)
SYN27556 (73.9)	SYN27556 (74.6)
PZE−105072012 (74.1)	PZE−105072012 (76.7)
SYN34071 (74.5)	SYN34071 (78)
PZE−105074598 (76.1)	PZE−105074598 (81.9)
PZE−105075776 (76.8)	PZE−105075776 (83.8)
PZE−105078090 (77.5)	PZE−105078090 (88.2)
PZA03049.24 (77.8)	PZA03049.24 (89.3)
PZE−105090424 (78)	PZE−105090424 (126.8)
PZE−105090726 (78.5)	PZE−105090726 (127.6)
PZE−105095318 (78.8)	PZE−105095318 (140.1)
PZE−105097569 (79.1)	PZE−105097569 (144)
PZE−105098226 (79.3)	PZE−105098226 (144.9)
PZE−105098779 (79.6)	PZE−105098779 (145.8)
SYN28772 (80)	SYN28772 (147.6)
PZE−105100878 (81.5)	PZE−105100878 (151.5)
SYN27822 (81.8)	SYN27822 (152.4)
PZE−105102856 (82.1)	PZE−105102856 (155.2)
PUT−163a−4174532−2029 (82.9)	PUT−163a−4174532−2029 (158.9)
PZE−105105213 (83.2)	PZE−105105213 (159.8)
PZE−105105462 (83.4)	PZE−105105462 (160.2)
PZE−105105756 (83.6)	PZE−105105756 (160.7)
PUT−163a−18162524−1229 (83.9)	PUT−163a−18162524−1229 (161.5)
SYN33595 (84.2)	SYN33595 (163.4)
PZE−105107370 (84.8)	PZE−105107370 (163.9)
SYN27038 (85.3)	SYN27038 (164.1)
SYN18294 (85.7)	SYN18294 (164.7)
PZE−105108927 (86.1)	PZE−105108927 (166)
PZE−105110067 (86.5)	PZE−105110067 (167.3)
PZE−105110200 (87.2)	PZE−105110200 (167.3)
SYN30458 (88.6)	SYN30458 (168.7)
PZE−105111928 (89.4)	PZE−105111928 (168.9)
SYN34468 (90.1)	SYN34468 (168.9)
PUT−163a−4573181−2059 (91.1)	PUT−163a−4573181−2059 (169.8)
PZE−105113974 (92)	PZE−105113974 (170.8)
PZE−105114470 (92.6)	PZE−105114470 (171.2)
SYN36136 (93.6)	SYN36136 (172)
PZE−105115758 (94.2)	PZE−105115758 (172.7)
PZE−105115904 (94.8)	PZE−105115904 (172.9)
SYN34982 (95.1)	SYN34982 (174.1)
SYN11012 (95.5)	SYN11012 (174.4)
SYN34521 (96.2)	SYN34521 (175.6)
SYN31820 (97.8)	SYN31820 (175.7)
SYN32464 (98.2)	SYN32464 (177.2)
PZE−105121226 (99)	PZE−105121226 (178)
PZE−105123093 (99.7)	PZE−105123093 (180)
SYN12712 (100.4)	SYN12712 (180.9)
SYN16682 (100.7)	SYN16682 (182)
PZE−105125294 (101)	PZE−105125294 (182.4)
SYN31836 (101.3)	SYN31836 (183.4)
PZE−105126109 (102)	PZE−105126109 (183.5)
PZE−105126444 (102.6)	PZE−105126444 (183.6)
PZE−105128217 (104.6)	PZE−105128217 (185.6)
PZE−105128384 (104.9)	PZE−105128384 (185.6)
PZE−105129422 (105.7)	PZE−105129422 (186.7)
SYN16485 (106.8)	SYN16485 (188.4)
SYN16484 (108.3)	SYN16484 (188.5)
SYN10270 (109.2)	SYN10270 (188.7)
SYN35847 (109.5)	SYN35847 (188.8)
SYN35853 (109.9)	SYN35853 (188.8)
PZE−105133024 (110.2)	PZE−105133024 (189.4)
SYN14521 (110.6)	SYN14521 (190.5)
PZE−105134993 (110.9)	PZE−105134993 (190.9)
SYN30405 (111.2)	SYN30405 (191.1)
PZE−105136340 (111.4)	PZE−105136340 (191.6)
SYN31644 (112.2)	SYN31644 (192.1)
SYN1851 (112.5)	SYN1851 (192.3)
PZE−105137712 (113.1)	PZE−105137712 (192.4)
SYN27782 (113.5)	SYN27782 (193.5)
SYN26067 (114.4)	SYN26067 (194)
SYN29255 (114.7)	SYN29255 (194.2)
SYN32728 (115.4)	SYN32728 (194.7)
SYN1176 (116.6)	SYN1176 (195.4)
PZE−105141007 (116.9)	PZE−105141007 (195.7)
PZE−105141391 (118.3)	PZE−105141391 (196)
PZE−105142315 (118.7)	PZE−105142315 (196.7)
SYN28488 (119.2)	SYN28488 (196.8)
SYN34083 (120.2)	SYN34083 (197.9)
PZE−105144035 (120.5)	PZE−105144035 (198)
PZE−105146498 (121.6)	PZE−105146498 (199.6)
PZE−105146705 (121.9)	PZE−105146705 (199.7)
PZE−105149244 (123.1)	PZE−105149244 (201.1)
SYN2083 (125.7)	SYN2083 (201.6)
SYN29327 (127.6)	SYN29327 (202.8)
SYN14923 (128)	SYN14923 (203)
SYN22059 (128.7)	SYN22059 (203.2)
SYN24779 (129)	SYN24779 (203.6)
SYN24659 (129.8)	SYN24659 (203.8)
SYN21802 (130.1)	SYN21802 (204.2)
SYN1884 (130.8)	SYN1884 (204.5)
SYN1926 (132.5)	SYN1926 (204.6)
SYN36851 (133.5)	SYN36851 (205.3)
PZE−105156453 (134.3)	PZE−105156453 (205.5)
PZB02189.6 (134.7)	PZB02189.6 (205.6)

B73 genome (Mbp)	LHRF map (cM)
PZE−105000171 (0.5)	PZE−105000171 (0)
SYN9890 (1.1)	SYN9890 (2.5)
SYN21619 (1.6)	SYN21619 (5.3)
SYN2371 (1.7)	SYN2371 (6.4)
SYN14907 (1.8)	SYN14907 (7.1)
PZE−105003814 (2.3)	PZE−105003814 (8.8)
SYN31748 (3.4)	SYN31748 (17.2)
SYN9983 (3.5)	SYN9983 (19.4)
SYN35950 (3.7)	SYN35950 (20.4)
SYN529 (4.3)	SYN529 (23.1)
SYNGENTA16696 (5.3)	SYNGENTA16696 (33.4)
SYN12680 (5.4)	SYN12680 (34.4)
ZM013390−0716 (5.5)	ZM013390−0716 (36.4)
SYN1042 (5.9)	SYN1042 (38)
PZE−105014611 (6.4)	PZE−105014611 (41.2)
PZA02462.1 (6.9)	PZA02462.1 (45.6)
PZE−105016296 (7.1)	PZE−105016296 (47)
PZE−105017254 (7.6)	PZE−105017254 (49)
PZE−105018063 (8)	PZE−105018063 (50.7)
PZE−105018841 (8.5)	PZE−105018841 (52.2)
PZE−105019465 (9.1)	PZE−105019465 (53.5)
SYN37837 (9.9)	SYN37837 (54.7)
SYNGENTA10434 (10.1)	SYNGENTA10434 (56.3)
PZE−105021560 (10.2)	PZE−105021560 (59.2)
PUT−163a−60349829−2621 (10.6)	PUT−163a−60349829−2621 (60.4)
PZA03092.7 (12)	PZA03092.7 (64.3)
PZE−105025087 (12.6)	PZE−105025087 (66.5)
PZE−105026164 (13.3)	PZE−105026164 (67.6)
SYN1692 (13.4)	SYN1692 (69.3)
PZE−105027530 (14.1)	PZE−105027530 (71.1)
PUT−163a−148947019−495 (14.3)	PUT−163a−148947019−495 (71.6)
PZE−105028067 (14.6)	PZE−105028067 (72.4)
SYN1407 (15.3)	SYN1407 (75.9)
PUT−163a−60348955−2602 (17.9)	PUT−163a−60348955−2602 (79.4)
SYN21204 (18.6)	SYN21204 (80.1)
SYN21201 (18.7)	SYN21201 (80.7)
PZE−105034124 (19.4)	PZE−105034124 (81)
PZE−105034376 (19.5)	PZE−105034376 (81.4)
PZE−105035340 (20.3)	PZE−105035340 (82)
PZE−105035804 (20.8)	PZE−105035804 (83.9)
PUT−163a−149015131−783 (24.2)	PUT−163a−149015131−783 (85)
PZE−105040897 (26.9)	PZE−105040897 (85.5)
PZE−105042248 (28.4)	PZE−105042248 (85.9)
PZE−105042338 (28.6)	PZE−105042338 (86.1)
PUT−163a−91873336−4773 (31.1)	PUT−163a−91873336−4773 (86.6)
PZE−105044057 (31.5)	PZE−105044057 (87)
PZE−105044425 (31.9)	PZE−105044425 (87.4)
PZE−105044893 (32.5)	PZE−105044893 (88.1)
PZB00869.4 (33.1)	PZB00869.4 (88.5)
PZE−105045981 (34)	PZE−105045981 (88.9)
SYN30639 (35)	SYN30639 (89.3)
PZE−105047745 (37.3)	PZE−105047745 (89.7)
PZE−105049624 (41.6)	PZE−105049624 (90.1)
PZE−105050411 (42.9)	PZE−105050411 (90.6)
PZE−105050638 (43.4)	PZE−105050638 (91)
SYN2065 (44.5)	SYN2065 (92.1)
PZE−105053643 (49.4)	PZE−105053643 (93.8)
PZE−105054053 (50.3)	PZE−105054053 (94.3)
PZE−105054634 (51.4)	PZE−105054634 (94.6)
PZE−105055664 (53.4)	PZE−105055664 (95.4)
PZE−105056389 (54.4)	PZE−105056389 (96.1)
PUT−163a−16920212−1047 (56.8)	PUT−163a−16920212−1047 (96.6)
PZA−001804001 (58.6)	PZA−001804001 (97.7)
PZA03451.6 (60)	PZA03451.6 (98.7)
PZE−105061645 (60.7)	PZE−105061645 (99.4)
PZE−105061722 (60.8)	PZE−105061722 (99.7)
PZE−105061880 (61)	PZE−105061880 (101.1)
PZE−105062629 (61.8)	PZE−105062629 (101.5)
PZE−105063137 (62.6)	PZE−105063137 (101.8)
SYN26865 (64.1)	SYN26865 (103.2)
PZE−105065396 (66.8)	PZE−105065396 (103.5)
SYN28088 (68.7)	SYN28088 (103.8)
PZE−105067036 (69.3)	PZE−105067036 (104.2)
PZE−105067126 (69.3)	PZE−105067126 (104.8)
PZE−105067552 (70.1)	PZE−105067552 (105.5)
PZE−105070577 (74.7)	PZE−105070577 (107.3)
PZE−105071013 (75.1)	PZE−105071013 (107.8)
PZE−105071692 (76.4)	PZE−105071692 (108)
PZE−105073687 (80.1)	PZE−105073687 (108.3)
PZE−105074008 (80.8)	PZE−105074008 (108.5)
PZE−105074181 (81)	PZE−105074181 (108.9)
PZE−105075006 (82.6)	PZE−105075006 (109.1)
PUT−163a−29945580−1813 (84.8)	PUT−163a−29945580−1813 (109.3)
PZE−105077018 (86)	PZE−105077018 (110.2)
PUT−163a−78088608−4213 (88.7)	PUT−163a−78088608−4213 (110.7)
PZE−105079073 (90.1)	PZE−105079073 (111)
PZE−105079742 (91.3)	PZE−105079742 (111.3)
PZE−105090595 (127.3)	PZE−105090595 (112.7)
PZE−105090801 (127.8)	PZE−105090801 (113.1)
PUT−163a−18167835−1301 (135.8)	PUT−163a−18167835−1301 (113.6)
PZE−105095118 (139.8)	PZE−105095118 (114)
PZE−105097045 (143)	PZE−105097045 (114.3)
PZE−105098158 (144.8)	PZE−105098158 (115.5)
PZE−105098226 (144.9)	PZE−105098226 (115.9)
PUT−163a−149012758−768 (146.8)	PUT−163a−149012758−768 (116.4)
PZE−105099516 (147.7)	PZE−105099516 (116.6)
PZE−105100971 (151.6)	PZE−105100971 (117.6)
PZE−105101490 (152.4)	PZE−105101490 (117.9)
PZE−105101867 (152.8)	PZE−105101867 (118.2)
PUT−163a−30705690−1900 (155.8)	PUT−163a−30705690−1900 (119.4)
PZE−105105066 (159.5)	PZE−105105066 (120.1)
PZE−105106832 (163.3)	PZE−105106832 (120.6)
PZE−105106949 (163.4)	PZE−105106949 (121.4)
SYN33595 (163.4)	SYN33595 (121.7)
PUT−163a−60357114−2824 (163.6)	PUT−163a−60357114−2824 (122)
PZE−105107668 (164.4)	PZE−105107668 (123.6)
PZE−105108343 (164.9)	PZE−105108343 (124.3)
PZE−105109045 (166.3)	PZE−105109045 (124.9)
PZE−105110003 (167.2)	PZE−105110003 (125.4)
PZE−105110200 (167.3)	PZE−105110200 (125.7)
ae1.8 (168.5)	ae1.8 (126.7)
SYN15711 (169.5)	SYN15711 (128)
PZE−105113978 (170.8)	PZE−105113978 (129.3)
PZE−105114670 (171.3)	PZE−105114670 (130.6)
PZE−105115758 (172.7)	PZE−105115758 (131.6)
PZA00987.1 (172.9)	PZA00987.1 (133.3)
SYN11021 (174.3)	SYN11021 (134.3)
SYN34523 (175.6)	SYN34523 (135)
PHM13696.11 (176.1)	PHM13696.11 (135.7)
PZE−105120411 (177)	PZE−105120411 (136.4)
SYN37740 (177.7)	SYN37740 (136.8)
SYN23392 (180.4)	SYN23392 (138.6)
SYN15525 (181.2)	SYN15525 (139.5)
SYN1324 (181.6)	SYN1324 (139.8)
PZE−105125004 (182.1)	PZE−105125004 (140)
PUT−163a−29544284−1768 (183.3)	PUT−163a−29544284−1768 (141.5)
PZE−105127053 (184.2)	PZE−105127053 (142.4)
PZE−105127954 (185.4)	PZE−105127954 (142.7)
PZA02919.1 (186.7)	PZA02919.1 (143.2)
PZE−105130516 (187)	PZE−105129902 (143.9)
PZA02383.1 (189.8)	PZE−105130516 (144.9)
PZE−105134474 (190.6)	PZA02383.1 (147.1)
PUT−163a−148964320−612 (191.8)	PZE−105134474 (148)
SYN31644 (192.1)	PUT−163a−148964320−612 (149.4)
PUT−163a−16918459−995 (192.2)	SYN31644 (150.2)
PZA02411.3 (192.8)	PUT−163a−16918459−995 (150.6)
PZE−105138143 (193.3)	PZA02411.3 (151)
PZE−105138874 (193.7)	PZE−105138143 (151.3)
PZA03618.1 (194)	PZE−105138874 (152.4)
SYN29254 (194.1)	PZA03618.1 (153.6)
PZE−105140438 (194.8)	SYN29254 (154)
PZE−105129902 (195.1)	PZE−105140438 (154.8)
PZE−105141007 (195.7)	PZE−105141007 (156.3)
SYN31100 (196.2)	SYN31100 (157.3)
PUT−163a−29544067−1761 (196.6)	PUT−163a−29544067−1761 (158.3)
SYN33650 (197.5)	SYN33650 (158.7)
PZE−105144068 (198)	PZE−105144068 (160.6)
PZE−105145021 (198.8)	PZE−105145021 (161.6)
PZA00652.17 (199.2)	PZA00652.17 (162.1)
PZE−105146300 (199.4)	PZE−105146300 (162.6)
PZB02283.1 (200.4)	PZB02283.1 (164.3)
PZE−105150268 (201.8)	PZE−105150268 (166.3)
PZE−105151612 (202.8)	PZE−105151612 (167.8)
SYN24660 (203.8)	SYN24660 (170.2)
PZE−105154210 (204.5)	PZE−105154210 (172.2)
PUT−163a−149088205−888 (204.8)	PUT−163a−149088205−888 (173.6)
PZE−105155892 (205.3)	PZE−105155892 (174.6)
PZE−105156210 (205.4)	PZE−105156210 (175.1)
PZE−105156902 (205.6)	PZE−105156902 (177.3)
PZE−105158473 (206.3)	PZE−105158473 (178.8)
PZE−105159228 (206.7)	PZE−105159228 (179.4)
SYNGENTA13997 (206.9)	SYNGENTA13997 (179.8)
PZE−105160408 (207.1)	PZE−105160408 (180.6)
PZE−105160636 (207.5)	PZE−105160636 (181.5)
PZE−105160757 (207.5)	PZE−105160757 (182.7)
PZE−105161125 (207.7)	PZE−105161125 (183.6)
PZE−105161488 (208.1)	PZE−105161488 (184.2)
PZE−105163425 (208.3)	PZE−105163425 (185.9)
PZE−105164629 (208.7)	PZE−105164629 (187.7)
PZE−105165114 (209)	PZE−105165114 (189)
PZE−105165602 (209.3)	PZE−105165602 (190.5)
PZE−105166527 (209.9)	PZE−105166527 (192.5)
PZE−105166721 (210.1)	PZE−105166721 (193.2)
PZA00836.1 (211.2)	PZA00836.1 (195.8)
SYN3377 (211.4)	SYN3377 (197.2)
PZA03339.3 (211.7)	PZA03339.3 (198.2)
PUT−163a−78112154−4305 (212)	PUT−163a−78112154−4305 (199.2)
SYN22387 (212.2)	SYN22387 (200)
PZE−105172533 (212.6)	PZE−105172533 (202.6)
PUT−163a−148962814−585 (212.8)	PUT−163a−148962814−585 (203.6)
SYN9177 (213)	SYN9177 (205)
PUT−163a−18162607−1230 (213.3)	PUT−163a−18162607−1230 (206.4)
SYN37237 (213.5)	SYN37237 (208.3)
SYN14678 (213.9)	SYN14690 (209.3)
SYN14690 (213.9)	SYN14678 (210.3)
PZE−105177818 (214.4)	PZE−105177818 (212.7)
ZM005841−0400 (214.6)	ZM005841−0400 (216.1)
SYN31322 (215)	SYN31322 (217.4)
PZE−105179864 (215.3)	PZE−105179864 (220.9)
PZE−105181381 (216)	PZE−105181381 (224.5)
PZE−105181974 (216.6)	PZE−105181974 (226.5)

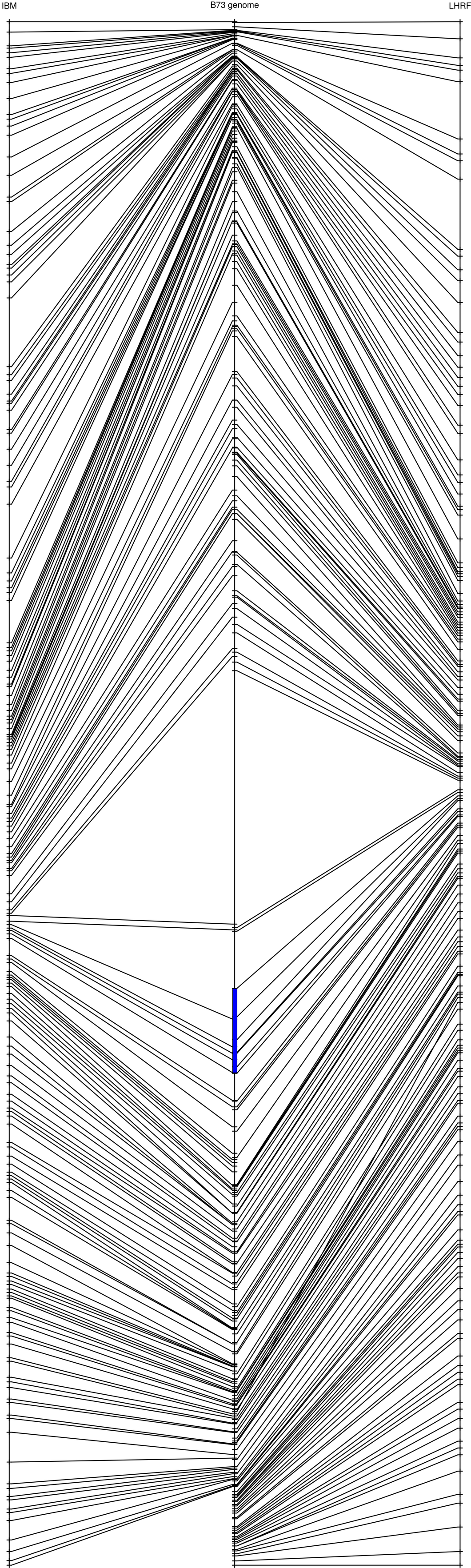

Chrom 6

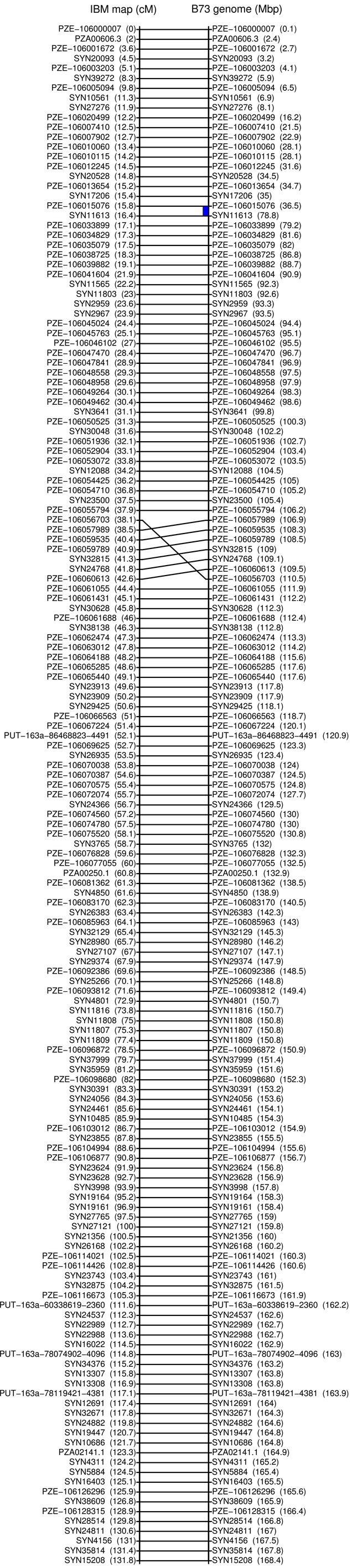
IBM map (cM)
B73 genome (Mbp)
PZE–106000007 (0)
PZA00606.3 (2)
PZE–106001672 (3.6)
SYN20093 (4.5)
PZE–106003203 (5.1)
SYN39272 (8.3)
PZE–106005094 (9.8)
SYN10561 (11.3)
SYN27276 (11.9)
PZE–106020499 (12.2)
PZE–106007410 (12.5)
PZE–106007902 (12.7)
PZE–106010060 (13.4)
PZE–106010115 (14.2)
PZE–106012245 (14.5)
SYN20528 (14.8)
PZE–106013654 (15.2)
SYN17206 (15.4)
PZE–106015076 (15.8)
SYN11613 (16.4)
PZE–106033899 (17.1)
PZE–106034829 (17.3)
PZE–106035079 (17.5)
PZE–106038725 (18.3)
PZE–106039882 (19.1)
PZE–106041604 (21.9)
SYN11565 (22.2)
SYN11803 (23)
SYN2959 (23.6)
SYN2967 (23.9)
PZE–106045024 (24.4)
PZE–106045763 (25.1)
PZE–106046102 (27)
PZE–106047470 (28.4)
PZE–106047841 (28.9)
PZE–106048558 (29.3)
PZE–106048958 (29.6)
PZE–106049264 (30.1)
PZE–106049462 (30.4)
SYN3641 (31.1)
PZE–106050525 (31.3)
SYN30048 (31.6)
PZE–106051936 (32.1)
PZE–106052904 (33.1)
PZE–106053072 (33.8)
SYN12088 (34.2)
PZE–106054425 (36.2)
PZE–106054710 (36.8)
SYN23500 (37.5)
PZE–106055794 (37.9)
PZE–106056703 (38.1)
PZE–106057989 (38.5)
PZE–106059535 (40.4)
PZE–106059789 (40.9)
SYN32815 (41.3)
SYN24768 (41.8)
PZE–106060613 (42.6)
PZE–106061055 (44.4)
PZE–106061431 (45.1)
SYN30628 (45.8)
PZE–106061688 (46)
SYN38138 (46.3)
PZE–106062474 (47.3)
PZE–106063012 (47.8)
PZE–106064188 (48.2)
PZE–106065285 (48.6)
PZE–106065440 (49.1)
SYN23913 (49.6)
SYN23909 (50.2)
SYN29425 (50.6)
PZE–106066563 (51)
PZE–106067224 (51.4)
PUT–163a–86468823–4491 (52.1)
PZE–106069625 (52.7)
SYN26935 (53.5)
PZE–106070038 (53.8)
PZE–106070387 (54.6)
PZE–106070575 (55.4)
PZE–106072074 (55.7)
SYN24366 (56.7)
PZE–106074560 (57.2)
PZE–106074780 (57.5)
PZE–106075520 (58.1)
SYN3765 (58.7)
PZE–106076828 (59.6)
PZE–106077055 (60)
PZA00250.1 (60.8)
PZE–106081362 (61.3)
SYN4850 (61.6)
PZE–106083170 (62.3)
SYN26383 (63.4)
PZE–106085963 (64.1)
SYN32129 (65.4)
SYN28980 (65.7)
SYN27107 (67)
SYN29374 (67.9)
PZE–106092386 (69.6)
SYN25266 (70.1)
PZE–106093812 (71.6)
SYN4801 (72.9)
SYN11816 (73.8)
SYN11808 (75)
SYN11807 (75.3)
SYN11809 (77.4)
PZE–106096872 (78.5)
SYN37999 (79.7)
SYN35959 (81.2)
PZE–106098680 (82)
SYN30391 (83.3)
SYN24056 (84.3)
SYN24461 (85.6)
SYN10485 (85.9)
PZE–106103012 (86.7)
SYN23855 (87.8)
PZE–106104994 (88.6)
PZE–106106877 (90.8)
SYN23624 (91.9)
SYN23628 (92.7)
SYN3998 (93.9)
SYN19164 (95.2)
SYN19161 (96.9)
SYN27765 (97.5)
SYN27121 (100)
SYN21356 (100.5)
SYN26168 (102.2)
PZE–106114021 (102.5)
PZE–106114426 (102.8)
SYN23743 (103.4)
SYN32875 (104.2)
PZE–106116673 (105.3)
PUT–163a–60338619–2360 (111.6)
SYN24537 (112.3)
SYN22989 (112.7)
SYN22988 (113.6)
SYN16022 (114.5)
PUT–163a–78074902–4096 (114.8)
SYN34376 (115.2)
SYN13307 (115.8)
SYN13308 (116.9)
PUT–163a–78119421–4381 (117.1)
SYN12691 (117.4)
SYN32671 (117.8)
SYN24882 (119.8)
SYN19447 (120.7)
SYN10686 (121.7)
PZA02141.1 (123.3)
SYN4311 (124.2)
SYN5884 (124.5)
SYN16403 (125.1)
PZE–106126296 (125.9)
SYN38609 (126.8)
PZE–106128315 (128.9)
SYN28514 (129.8)
SYN24811 (130.6)
SYN4156 (131)
SYN35814 (131.4)
SYN15208 (131.8)
PZE–106000007 (0.1)
PZA00606.3 (2.4)
PZE–106001672 (2.7)
SYN20093 (3.2)
PZE–106003203 (4.1)
SYN39272 (5.9)
PZE–106005094 (6.5)
SYN10561 (6.9)
SYN27276 (8.1)
PZE–106020499 (16.2)
PZE–106007410 (21.5)
PZE–106007902 (22.9)
PZE–106010060 (28.1)
PZE–106010115 (28.1)
PZE–106012245 (31.6)
SYN20528 (34.5)
PZE–106013654 (34.7)
SYN17206 (35)
PZE–106015076 (36.5)
SYN11613 (78.8)
PZE–106033899 (79.2)
PZE–106034829 (81.6)
PZE–106035079 (82)
PZE–106038725 (86.8)
PZE–106039882 (88.7)
PZE–106041604 (90.9)
SYN11565 (92.3)
SYN11803 (92.6)
SYN2959 (93.3)
SYN2967 (93.5)
PZE–106045024 (94.4)
PZE–106045763 (95.1)
PZE–106046102 (95.5)
PZE–106047470 (96.7)
PZE–106047841 (96.9)
PZE–106048558 (97.5)
PZE–106048958 (97.9)
PZE–106049264 (98.3)
PZE–106049462 (98.6)
SYN3641 (99.8)
PZE–106050525 (100.3)
SYN30048 (102.2)
PZE–106051936 (102.7)
PZE–106052904 (103.4)
PZE–106053072 (103.5)
SYN12088 (104.5)
PZE–106054425 (105)
PZE–106054710 (105.2)
SYN23500 (105.4)
PZE–106055794 (106.2)
PZE–106057989 (106.9)
PZE–106059535 (108.3)
PZE–106059789 (108.5)
SYN32815 (109)
SYN24768 (109.1)
PZE–106060613 (109.5)
PZE–106056703 (110.5)
PZE–106061055 (111.9)
PZE–106061431 (112.2)
SYN30628 (112.3)
PZE–106061688 (112.4)
SYN38138 (112.8)
PZE–106062474 (113.3)
PZE–106063012 (114.2)
PZE–106064188 (115.6)
PZE–106065285 (117.6)
PZE–106065440 (117.6)
SYN23913 (117.8)
SYN23909 (117.9)
SYN29425 (118.1)
PZE–106066563 (118.7)
PZE–106067224 (120.1)
PUT–163a–86468823–4491 (120.9)
PZE–106069625 (123.3)
SYN26935 (123.4)
PZE–106070038 (124)
PZE–106070387 (124.5)
PZE–106070575 (124.8)
PZE–106072074 (127.7)
SYN24366 (129.5)
PZE–106074560 (130)
PZE–106074780 (130)
PZE–106075520 (130.8)
SYN3765 (132)
PZE–106076828 (132.3)
PZE–106077055 (132.5)
PZA00250.1 (132.9)
PZE–106081362 (138.5)
SYN4850 (138.9)
PZE–106083170 (140.5)
SYN26383 (142.3)
PZE–106085963 (143)
SYN32129 (145.3)
SYN28980 (146.2)
SYN27107 (147.1)
SYN29374 (147.9)
PZE–106092386 (148.5)
SYN25266 (148.8)
PZE–106093812 (149.4)
SYN4801 (150.7)
SYN11816 (150.7)
SYN11808 (150.8)
SYN11807 (150.8)
SYN11809 (150.8)
PZE–106096872 (150.9)
SYN37999 (151.4)
SYN35959 (151.6)
PZE–106098680 (152.3)
SYN30391 (153.2)
SYN24056 (153.6)
SYN24461 (154.1)
SYN10485 (154.3)
PZE–106103012 (154.9)
SYN23855 (155.5)
PZE–106104994 (155.6)
PZE–106106877 (156.7)
SYN23624 (156.8)
SYN23628 (156.9)
SYN3998 (157.8)
SYN19164 (158.3)
SYN19161 (158.4)
SYN27765 (159)
SYN27121 (159.8)
SYN21356 (160)
SYN26168 (160.2)
PZE–106114021 (160.3)
PZE–106114426 (160.6)
SYN23743 (161)
SYN32875 (161.5)
PZE–106116673 (161.9)
PUT–163a–60338619–2360 (162.2)
SYN24537 (162.6)
SYN22989 (162.7)
SYN22988 (162.7)
SYN16022 (162.9)
PUT–163a–78074902–4096 (163)
SYN34376 (163.2)
SYN13307 (163.8)
SYN13308 (163.8)
PUT–163a–78119421–4381 (163.9)
SYN12691 (164)
SYN32671 (164.3)
SYN24882 (164.6)
SYN19447 (164.8)
SYN10686 (164.8)
PZA02141.1 (164.9)
SYN4311 (165.2)
SYN5884 (165.4)
SYN16403 (165.5)
PZE–106126296 (165.6)
SYN38609 (165.9)
PZE–106128315 (166.4)
SYN28514 (166.8)
SYN24811 (167)
SYN4156 (167.5)
SYN35814 (167.8)
SYN15208 (168.4)

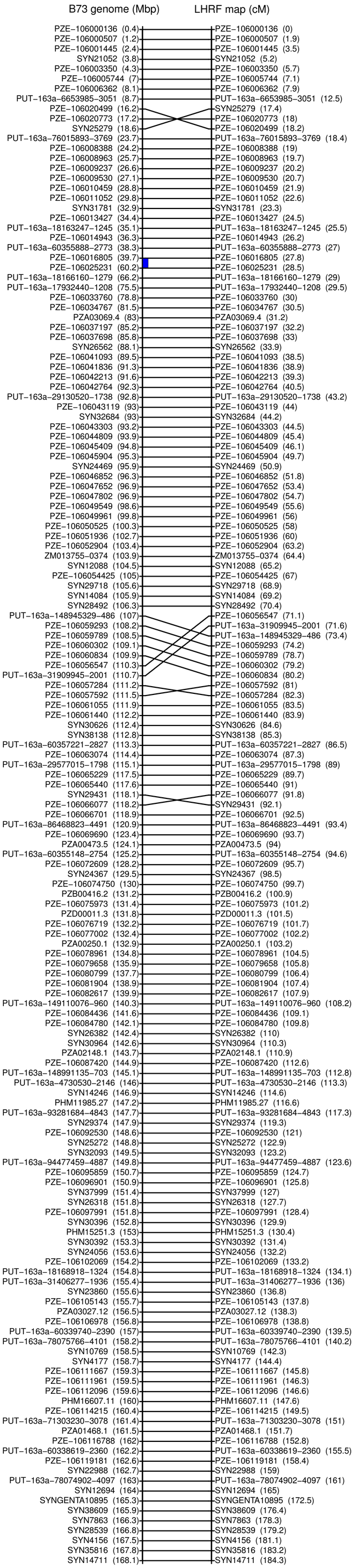
B73 genome (Mbp)
LHRF map (cM)
PZE–106000136 (0.4)
PZE–106000507 (1.2)
PZE–106001445 (2.4)
SYN21052 (3.8)
PZE–106003350 (4.3)
PZE–106005744 (7)
PZE–106006362 (8.1)
PUT–163a–6653985–3051 (8.7)
PZE–106020499 (16.2)
PZE–106020773 (17.2)
SYN25279 (18.6)
PUT–163a–76015893–3769 (23.7)
PZE–106008388 (24.2)
PZE–106008963 (25.7)
PZE–106009237 (26.6)
PZE–106009530 (27.1)
PZE–106010459 (28.8)
PZE–106011052 (29.8)
SYN31781 (32.9)
PZE–106013427 (34.4)
PUT–163a–18163247–1245 (35.1)
PZE–106014943 (36.3)
PUT–163a–60355888–2773 (38.3)
PZE–106016805 (39.7)
PZE–106025231 (60.2)
PUT–163a–18166160–1279 (66.2)
PUT–163a–17932440–1208 (75.5)
PZE–106033760 (78.8)
PZE–106034767 (81.5)
PZA03069.4 (83)
PZE–106037197 (85.2)
PZE–106037698 (85.8)
SYN26562 (88.1)
PZE–106041093 (89.5)
PZE–106041836 (91.3)
PZE–106042213 (91.6)
PZE–106042764 (92.3)
PUT–163a–29130520–1738 (92.8)
PZE–106043119 (93)
SYN32684 (93)
PZE–106043303 (93.2)
PZE–106044809 (93.9)
PZE–106045409 (94.8)
PZE–106045904 (95.3)
SYN24469 (95.9)
PZE–106046852 (96.3)
PZE–106047652 (96.9)
PZE–106047802 (96.9)
PZE–106049549 (98.6)
PZE–106049961 (99.8)
PZE–106050525 (100.3)
PZE–106051936 (102.7)
PZE–106052904 (103.4)
ZM013755–0374 (103.9)
SYN12088 (104.5)
PZE–106054425 (105)
SYN29718 (105.6)
SYN14084 (105.9)
SYN28492 (106.3)
PUT–163a–148945329–486 (107)
PZE–106059293 (108.2)
PZE–106059789 (108.5)
PZE–106060302 (109.1)
PZE–106060834 (109.9)
PZE–106056547 (110.3)
PUT–163a–31909945–2001 (110.7)
PZE–106057284 (111.2)
PZE–106057592 (111.5)
PZE–106061055 (111.9)
PZE–106061440 (112.2)
SYN30626 (112.4)
SYN38138 (112.8)
PUT–163a–60357221–2827 (113.3)
PZE–106063074 (114.4)
PUT–163a–29577015–1798 (115.1)
PZE–106065229 (117.5)
PZE–106065440 (117.6)
SYN29431 (118.1)
PZE–106066077 (118.2)
PZE–106066701 (118.9)
PUT–163a–86468823–4491 (120.9)
PZE–106069690 (123.4)
PZA00473.5 (124.1)
PUT–163a–60355148–2754 (125.2)
PZE–106072609 (128.2)
SYN24367 (129.5)
PZE–106074750 (130)
PZB00416.2 (131.2)
PZE–106075973 (131.4)
PZD00011.3 (131.8)
PZE–106076719 (132.2)
PZE–106077002 (132.4)
PZA00250.1 (132.9)
PZE–106078961 (134.8)
PZE–106079658 (135.9)
PZE–106080799 (137.7)
PZE–106081904 (138.9)
PZE–106082617 (139.9)
PUT–163a–149110076–960 (140.3)
PZE–106084436 (141.6)
PZE–106084780 (142.1)
SYN26382 (142.4)
SYN30964 (142.6)
PZA02148.1 (143.7)
PZE–106087420 (144.9)
PUT–163a–148991135–703 (145.1)
PUT–163a–4730530–2146 (146)
SYN14246 (146.9)
PHM11985.27 (147.2)
PUT–163a–93281684–4843 (147.7)
SYN29374 (147.9)
PZE–106092530 (148.6)
SYN25272 (148.8)
SYN32093 (149.5)
PUT–163a–94477459–4887 (149.8)
PZE–106095859 (150.7)
PZE–106096901 (150.9)
SYN37999 (151.4)
SYN26318 (151.8)
PZE–106097991 (151.8)
SYN30396 (152.8)
PHM15251.3 (153)
SYN30392 (153.3)
SYN24056 (153.6)
PZE–106102069 (154.2)
PUT–163a–18168918–1324 (154.8)
PUT–163a–31406277–1936 (155.4)
SYN23860 (155.6)
PZE–106105143 (155.7)
PZA03027.12 (156.5)
PZE–106106978 (156.8)
PUT–163a–60339740–2390 (157)
PUT–163a–78075766–4101 (158.2)
SYN10769 (158.5)
SYN4177 (158.7)
PZE–106111667 (159.3)
PZE–106111961 (159.5)
PZE–106112096 (159.6)
PHM16607.11 (160)
PZE–106114215 (160.4)
PUT–163a–71303230–3078 (161.4)
PZA01468.1 (161.5)
PZE–106116788 (162)
PUT–163a–60338619–2360 (162.2)
PZE–106119181 (162.6)
SYN22988 (162.7)
PUT–163a–78074902–4097 (163)
SYN12694 (164)
SYNGENTA10895 (165.3)
SYN38609 (165.9)
SYN7863 (166.3)
SYN28539 (166.8)
SYN4156 (167.5)
SYN35816 (167.8)
SYN14711 (168.1)
PZE–106000136 (0)
PZE–106000507 (1.9)
PZE–106001445 (3.5)
SYN21052 (5.2)
PZE–106003350 (5.7)
PZE–106005744 (7.1)
PZE–106006362 (7.9)
PUT–163a–6653985–3051 (12.5)
SYN25279 (17.4)
PZE–106020773 (18)
PZE–106020499 (18.2)
PUT–163a–76015893–3769 (18.4)
PZE–106008388 (19)
PZE–106008963 (19.7)
PZE–106009237 (20.2)
PZE–106009530 (20.7)
PZE–106010459 (21.9)
PZE–106011052 (22.6)
SYN31781 (23.3)
PZE–106013427 (24.5)
PUT–163a–18163247–1245 (25.5)
PZE–106014943 (26.2)
PUT–163a–60355888–2773 (27)
PZE–106016805 (27.8)
PZE–106025231 (28.5)
PUT–163a–18166160–1279 (29)
PUT–163a–17932440–1208 (29.5)
PZE–106033760 (30)
PZE–106034767 (30.5)
PZA03069.4 (31.2)
PZE–106037197 (32.2)
PZE–106037698 (33)
SYN26562 (33.9)
PZE–106041093 (38.5)
PZE–106041836 (38.9)
PZE–106042213 (39.3)
PZE–106042764 (40.5)
PUT–163a–29130520–1738 (43.2)
PZE–106043119 (44)
SYN32684 (44.2)
PZE–106043303 (44.5)
PZE–106044809 (45.4)
PZE–106045409 (46.1)
PZE–106045904 (49.7)
SYN24469 (50.9)
PZE–106046852 (51.8)
PZE–106047652 (53.4)
PZE–106047802 (54.7)
PZE–106049549 (55.6)
PZE–106049961 (56)
PZE–106050525 (58)
PZE–106051936 (60)
PZE–106052904 (63.2)
ZM013755–0374 (64.4)
SYN12088 (65.2)
PZE–106054425 (67)
SYN29718 (68.9)
SYN14084 (69.2)
SYN28492 (70.4)
PZE–106056547 (71.1)
PUT–163a–31909945–2001 (71.6)
PUT–163a–148945329–486 (73.4)
PZE–106059293 (74.2)
PZE–106059789 (78.7)
PZE–106060302 (79.2)
PZE–106060834 (80.2)
PZE–106057592 (81)
PZE–106057284 (82.3)
PZE–106061055 (83.5)
PZE–106061440 (83.9)
SYN30626 (84.6)
SYN38138 (85.3)
PUT–163a–60357221–2827 (86.5)
PZE–106063074 (87.3)
PUT–163a–29577015–1798 (89)
PZE–106065229 (89.7)
PZE–106065440 (91)
PZE–106066077 (91.8)
SYN29431 (92.1)
PZE–106066701 (92.5)
PUT–163a–86468823–4491 (93.4)
PZE–106069690 (93.7)
PZA00473.5 (94)
PUT–163a–60355148–2754 (94.6)
PZE–106072609 (95.7)
SYN24367 (98.5)
PZE–106074750 (99.7)
PZB00416.2 (100.9)
PZE–106075973 (101.2)
PZD00011.3 (101.5)
PZE–106076719 (101.7)
PZE–106077002 (102.2)
PZA00250.1 (103.2)
PZE–106078961 (104.5)
PZE–106079658 (105.8)
PZE–106080799 (106.4)
PZE–106081904 (107.4)
PZE–106082617 (107.9)
PUT–163a–149110076–960 (108.2)
PZE–106084436 (109.1)
PZE–106084780 (109.8)
SYN26382 (110)
SYN30964 (110.3)
PZA02148.1 (110.9)
PZE–106087420 (112.6)
PUT–163a–148991135–703 (112.8)
PUT–163a–4730530–2146 (113.3)
SYN14246 (114.6)
PHM11985.27 (116.6)
PUT–163a–93281684–4843 (117.3)
SYN29374 (119.3)
PZE–106092530 (121)
SYN25272 (122.9)
SYN32093 (123.2)
PUT–163a–94477459–4887 (123.6)
PZE–106095859 (124.7)
PZE–106096901 (125.8)
SYN37999 (127)
SYN26318 (127.7)
PZE–106097991 (128.4)
SYN30396 (129.9)
PHM15251.3 (130.4)
SYN30392 (131.4)
SYN24056 (132.2)
PZE–106102069 (133.2)
PUT–163a–18168918–1324 (134.1)
PUT–163a–31406277–1936 (136)
SYN23860 (136.8)
PZE–106105143 (137.8)
PZA03027.12 (138.3)
PZE–106106978 (138.8)
PUT–163a–60339740–2390 (139.5)
PUT–163a–78075766–4101 (140.2)
SYN10769 (142.3)
SYN4177 (144.4)
PZE–106111667 (145.8)
PZE–106111961 (146.3)
PZE–106112096 (146.6)
PHM16607.11 (147.6)
PZE–106114215 (149.5)
PUT–163a–71303230–3078 (151)
PZA01468.1 (151.7)
PZE–106116788 (152.8)
PUT–163a–60338619–2360 (155.5)
PZE–106119181 (158.4)
SYN22988 (159)
PUT–163a–78074902–4097 (161)
SYN12694 (165)
SYNGENTA10895 (172.5)
SYN38609 (176.4)
SYN7863 (178.3)
SYN28539 (179.2)
SYN4156 (181.1)
SYN35816 (183.2)
SYN14711 (184.3)

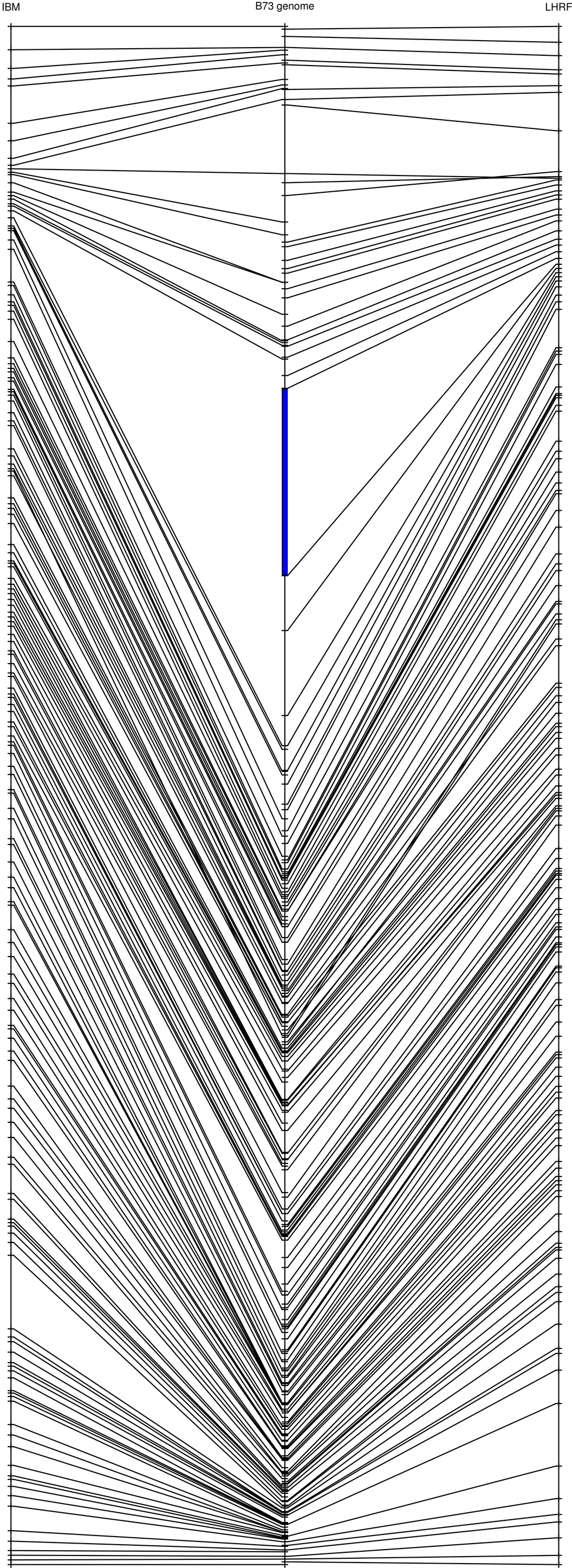
IBM
B73 genome
LHRF

Chrom 7

IBM map (cM)	B73 genome (Mbp)
SYN23973 (0)	SYN23973 (3.2)
PZE−107005418 (1)	PZE−107005418 (3.7)
PZE−107005664 (1.5)	PZE−107005664 (3.9)
SYN39291 (1.9)	SYN39291 (4.1)
SYN25440 (2.9)	SYN25440 (4.2)
PZE−107006511 (4.1)	PZE−107006511 (4.7)
SYN21561 (4.7)	SYN21561 (4.9)
SYN21564 (5.9)	SYN21564 (4.9)
SYN21560 (8)	SYN21560 (5)
SYN15673 (8.7)	SYN15673 (5)
SYN4744 (9)	SYN4744 (5.1)
SYN3807 (11.3)	SYN3807 (5.5)
SYN3808 (12.2)	SYN3808 (5.6)
PZE−107009278 (13.1)	PZE−107009278 (6.1)
SYN36033 (14.5)	SYN36033 (6.2)
PZE−107010082 (15.1)	PZE−107010082 (6.6)
PZE−107010433 (16.7)	PZE−107010433 (7)
PZE−107011423 (17.5)	PZE−107011423 (8)
SYN32202 (18.2)	SYN32202 (8.5)
PZE−107012245 (18.9)	PZE−107012245 (8.8)
SYN18116 (20.9)	SYN18116 (9.2)
SYN36577 (24.2)	SYN36577 (9.3)
PUT−163a−50327005−2185 (26.5)	PUT−163a−50327005−2185 (10.3)
PZE−107014250 (27)	PZE−107014250 (10.7)
SYN33126 (27.5)	SYN33126 (13.6)
PZE−107016614 (28.5)	PZE−107016614 (14)
SYN26435 (30)	SYN26435 (15.1)
PZE−107017614 (31)	PZE−107017614 (15.2)
SYN20980 (31.7)	SYN20980 (15.4)
PZE−107019072 (32.7)	PZE−107019072 (17.3)
SYN37382 (33.1)	SYN37382 (19.1)
PZE−107021654 (33.4)	PZE−107021654 (21.1)
SYN10101 (33.8)	SYN10101 (21.2)
PUT−163a−21329873−1510 (34)	PUT−163a−21329873−1510 (21.6)
PZE−107022242 (34.3)	PZE−107022242 (21.9)
SYN23593 (35.6)	SYN23593 (24.4)
SYN23597 (35.9)	SYN23597 (24.4)
SYN14571 (36.4)	SYN14571 (27.8)
PZE−107026046 (36.7)	PZE−107026046 (29.1)
SYN4359 (37.1)	SYN4359 (30.2)
PZE−107030745 (37.4)	PZE−107030745 (39.6)
PZE−107031924 (37.9)	PZE−107031924 (41.5)
PZE−107040458 (38.3)	PZE−107040458 (79.1)
SYN29842 (38.8)	SYN29842 (84.7)
PZE−107044548 (39.1)	PZE−107044548 (89.3)
PZE−107050868 (39.5)	PZE−107050868 (100.3)
PZE−107051446 (39.9)	PZE−107051446 (100.9)
PZE−107053268 (40.2)	PZE−107053268 (104.3)
SYN14373 (40.4)	SYN14373 (105.4)
PZE−107054656 (41.2)	PZE−107054656 (106.3)
SYN16553 (41.7)	SYN16553 (107.9)
SYN39188 (42.9)	SYN39188 (109.1)
PZE−107058710 (43.2)	PZE−107058710 (112.6)
PZE−107059742 (43.6)	PZE−107059742 (115.2)
SYN36345 (44)	SYN36345 (116.5)
PZE−107061311 (44.3)	PZE−107061311 (117.5)
SYN18744 (44.6)	SYN18744 (117.7)
PZE−107062095 (44.9)	PZE−107062095 (118.3)
SYN3200 (45.2)	SYN3200 (118.6)
PZE−107062980 (45.4)	PZE−107062980 (120)
PZE−107064289 (45.6)	PZE−107064289 (121.3)
SYN3716 (46.4)	SYN3716 (121.7)
SYN21763 (47)	SYN21763 (122.3)
PZE−107065974 (48.4)	PZE−107065974 (122.8)
PZE−107066119 (48.8)	PZE−107066119 (123)
PZE−107067062 (50.2)	PZE−107067062 (123.8)
PZE−107067568 (50.7)	PZE−107067568 (124.3)
SYN25079 (51.1)	SYN25079 (124.6)
PZE−107068407 (51.4)	PZE−107068407 (125.1)
PZE−107068961 (52)	PZE−107068961 (125.9)
PZE−107070761 (52.5)	PZE−107070761 (127.3)
SYN20393 (52.8)	SYN20393 (127.6)
SYN17089 (53.9)	SYN17089 (128.4)
PZE−107072604 (54.3)	PZE−107072604 (128.9)
SYN27282 (54.6)	SYN27282 (129.3)
PZE−107073339 (55.4)	PZE−107073339 (129.9)
SYN28758 (56.4)	SYN28758 (130)
PZE−107074755 (58.1)	PZE−107074755 (130.5)
PZE−107074954 (58.8)	PZE−107074954 (130.8)
SYN17332 (59.4)	SYN17332 (130.9)
PZE−107075566 (60.2)	PZE−107075566 (131)
PZE−107076020 (60.5)	PZE−107076020 (131.2)
PZE−107076350 (61)	PZE−107076350 (131.4)
PZE−107076684 (61.8)	PZE−107076684 (132.1)
SYN23059 (62.7)	SYN23059 (132.5)
SYN35079 (63.2)	SYN35079 (132.6)
SYN38308 (63.5)	SYN38308 (132.8)
SYN32295 (64.7)	SYN32295 (133.4)
PUT−163a−71300000−3068 (65.4)	PUT−163a−71300000−3068 (133.9)
SYN22406 (66.9)	SYN22406 (134.1)
PZE−107079812 (67.6)	PZE−107079812 (134.3)
PZE−107083154 (67.8)	PZE−107080376 (135.5)
PZE−107082619 (68.2)	PZE−107081437 (136.3)
PZE−107081437 (68.5)	PZE−107082619 (137.5)
PZE−107080376 (69.2)	PZE−107083154 (137.7)
PZE−107084474 (69.6)	PZE−107084474 (140.1)
SYN22167 (71.1)	SYN22167 (141.8)
PZE−107087898 (72.2)	PZE−107087898 (143)
SYN35906 (72.8)	SYN35906 (143.1)
SYN24399 (73)	SYN24399 (143.4)
SYN24395 (73.5)	SYN24395 (143.4)
SYN22616 (74)	SYN22616 (144.6)
SYN32262 (74.9)	SYN32262 (146.5)
SYN34204 (75.4)	SYN34204 (151.2)
PZE−107096184 (75.7)	PZE−107096184 (152.1)
PZE−107097026 (76.4)	PZE−107097026 (152.4)
PZE−107097554 (76.8)	PZE−107097554 (152.8)
SYN34174 (77)	SYN34174 (152.8)
PZE−107098163 (77.4)	PZE−107098163 (153.3)
SYN18022 (77.6)	SYN18022 (153.8)
PZE−107098780 (77.9)	PZE−107098780 (153.8)
PZE−107098889 (78.1)	PZE−107098889 (153.8)
PZE−107099144 (79)	PZE−107099144 (154.2)
PZE−107099226 (79.7)	PZE−107099226 (154.2)
PZE−107100523 (80.1)	PZE−107100523 (155)
SYN3467 (80.5)	SYN3467 (155.7)
PZE−107101783 (81)	PZE−107101783 (155.8)
PZE−107102206 (81.3)	PZE−107102206 (156.2)
SYN10134 (81.5)	SYN10134 (156.5)
SYN10135 (81.8)	SYN10135 (156.5)
SYN3652 (82.1)	SYN3652 (156.7)
PZE−107103880 (82.3)	PZE−107103880 (156.9)
PZA03176.4 (82.5)	PZA03176.4 (157.2)
SYN17072 (83)	SYN17072 (157.5)
PZE−107105855 (83.5)	PZE−107105855 (157.9)
PZE−107106251 (84.8)	PZE−107106251 (158.2)
PZE−107106472 (86.3)	PZE−107106472 (158.3)
PZE−107108370 (87.6)	PZE−107108370 (159.7)
SYN3350 (88.6)	SYN3350 (159.8)
SYN29615 (88.9)	SYN29615 (160.1)
SYN23166 (89.4)	SYN23166 (160.2)
SYN10522 (89.9)	SYN10522 (160.8)
PZE−107111253 (90.4)	PZE−107111253 (161.3)
SYN15451 (91.7)	SYN15451 (161.8)
PZE−107112738 (92.5)	PZE−107112738 (161.9)
PZE−107113339 (95.7)	PZE−107113339 (162.1)
PZE−107113484 (96.1)	PZE−107113484 (162.2)
PZE−107113723 (96.5)	PZE−107113723 (162.3)
SYN25311 (99.3)	SYN25311 (163)
SYN18482 (99.7)	SYN18482 (163)
PZE−107116723 (100.8)	PZE−107116723 (164.1)
SYN17036 (101.2)	SYN17036 (164.2)
PZE−107117917 (102.1)	PZE−107117917 (164.8)
SYN23688 (103)	SYN23688 (165)
PZE−107118743 (104)	PZE−107118743 (165.3)
PZE−107119102 (104.8)	PZE−107119102 (165.4)
SYN13029 (105.1)	SYN13029 (165.5)
PZE−107119968 (106.3)	PZE−107119968 (165.7)
SYN12703 (109.8)	SYN12703 (166.2)
SYN20121 (111.1)	SYN20121 (167)
PZE−107123572 (115.8)	PZE−107123572 (167.7)
SYN12253 (117.5)	SYN12253 (168)
PZE−107126296 (120.2)	PZE−107126296 (168.9)
SYN30795 (121.3)	SYN30795 (169.4)
SYN16914 (122.2)	SYN16914 (170)
SYN10749 (123)	SYN10749 (170.4)
SYN25224 (123.5)	SYN25224 (170.6)
SYN34109 (135.8)	SYN34109 (172.9)

B73 genome (Mbp)	LHRF map (cM)
PZE−107000722 (1)	PZE−107000722 (0)
SYNGENTA5501 (1.3)	SYNGENTA5501 (3.2)
PZE−107002539 (2.1)	PZE−107002539 (8.5)
SYNGENTA14859 (2.6)	SYNGENTA14859 (10.5)
PZE−107004578 (3)	PZE−107004578 (13.8)
PZE−107004786 (3.1)	PZE−107004786 (14.8)
PZE−107005392 (3.7)	PZE−107005392 (18.7)
SYN39284 (4.1)	SYN39284 (19.7)
SYN18502 (4.5)	SYN18502 (20.8)
SYN21578 (4.9)	SYN21578 (23.4)
PUT−163a−71419466−3205 (5.1)	PUT−163a−71419466−3205 (28.4)
SYN3807 (5.5)	SYN3807 (30.5)
PZE−107008894 (5.8)	PZE−107008894 (31.6)
PZE−107009208 (6)	PZE−107009208 (34.5)
PZE−107010082 (6.6)	PZE−107010082 (37.1)
PZE−107010804 (7.4)	PZE−107010804 (40.6)
PUT−163a−149110508−964 (7.5)	PUT−163a−149110508−964 (41.3)
SYN19838 (7.5)	SYN19838 (41.8)
PZE−107011423 (8)	PZE−107011423 (42.4)
ZM012606−0732 (8.1)	ZM012606−0732 (42.9)
PZE−107012245 (8.8)	PZE−107012245 (43.8)
PZE−107012564 (9.2)	PZE−107012564 (44.4)
SYN36582 (9.3)	SYN36582 (48.7)
PZE−107013677 (10.2)	PZE−107013677 (50.6)
PUT−163a−89252858−4663 (11.4)	PUT−163a−89252858−4663 (52.8)
PZE−107014986 (11.7)	PZE−107014986 (53.7)
PZE−107015089 (11.8)	PZE−107015089 (54.3)
SYN33126 (13.6)	SYN33126 (54.8)
PZE−107016512 (13.8)	PZE−107016512 (55.7)
PZE−107018278 (15.9)	PZE−107018278 (58)
PZE−107019024 (17.3)	PZE−107019024 (60.2)
SYN27396 (17.7)	SYN27396 (60.9)
PZE−107019436 (17.7)	PZE−107019436 (61.2)
PUT−163a−148928654−351 (19.3)	PUT−163a−148928654−351 (61.6)
PZE−107021428 (20.9)	PZE−107021428 (61.8)
SYN10100 (21.2)	SYN10100 (62.3)
PUT−163a−21329873−1510 (21.6)	PUT−163a−21329873−1510 (62.7)
PUT−163a−4647401−2124 (24.4)	PUT−163a−4647401−2124 (64.3)
PZE−107024779 (26.9)	PZE−107024779 (64.8)
PZE−107025581 (28)	PZE−107025581 (65.5)
PZE−107026273 (29.4)	PZE−107026273 (66)
PHM15501.9 (30.7)	PHM15501.9 (66.4)
PZE−107028879 (34.9)	PZE−107028879 (66.9)
PZA03363.1 (47.4)	PZA03363.1 (67.3)
PZE−107035547 (51.5)	PZE−107035547 (67.6)
PHM12830.14 (82.6)	PHM12830.14 (67.9)
PZA03645.2 (84.8)	PZA03645.2 (68.2)
PZA02291.1 (85.9)	PZA02291.1 (68.5)
PZE−107045881 (94)	PZE−107045881 (69)
PZE−107052509 (103.3)	PZE−107052509 (70.8)
PZE−107053519 (104.9)	PZE−107053519 (71.3)
SYN14372 (105.4)	SYN14372 (71.7)
PZE−107054120 (105.4)	PZE−107054120 (72.1)
PZE−107055620 (107.7)	PZE−107055620 (72.7)
PZE−107056322 (108.5)	PZE−107056322 (73.1)
PZE−107056650 (108.8)	PZE−107056650 (73.8)
PZB00175.6 (110.3)	PZB00175.6 (74.3)
PZE−107057948 (111.3)	PZE−107057948 (75.2)
PZE−107058109 (111.7)	PZE−107058109 (75.9)
PZE−107058951 (113)	PZE−107058951 (76.6)
PUT−163a−28873821−1636 (116.4)	PUT−163a−28873821−1636 (78.5)
PZE−107061080 (117.3)	PZE−107061080 (79.7)
PZE−107061306 (117.5)	PZE−107061306 (80)
SYN18268 (118.3)	SYN18268 (80.3)
SYN12277 (118.6)	SYN12277 (81)
PZE−107063028 (120.1)	PZE−107063028 (83.2)
PZE−107063357 (120.6)	PZE−107063357 (83.9)
PZE−107063658 (120.9)	PZE−107063658 (84.4)
PZE−107063848 (121)	PZE−107063848 (85.4)
PZE−107064215 (121.2)	PZE−107064215 (86.6)
PZE−107064549 (121.4)	PZE−107064549 (87.3)
SYN17969 (122.5)	SYN17969 (89.2)
PZE−107066014 (122.8)	PZE−107066014 (90.3)
PZE−107067197 (123.9)	PZE−107067197 (93.6)
PZE−107068652 (125.4)	PZE−107068652 (95.2)
PZE−107069611 (126.4)	PZE−107069611 (96.6)
PZE−107070830 (127.3)	PZE−107070830 (97.3)
PZE−107070987 (127.3)	PZE−107070987 (97.8)
PZE−107071564 (127.7)	PZE−107071564 (98.2)
PZE−107072025 (128.1)	PZE−107072025 (100)
PZE−107073069 (129.4)	PZE−107073069 (101)
SYN28758 (130)	SYN28758 (102.3)
PZE−107074506 (130.4)	PZE−107074506 (103.7)
PZE−107075781 (131.1)	PZE−107075781 (107.1)
PZE−107076218 (131.4)	PZE−107076218 (107.6)
PZE−107076827 (132.2)	PZE−107076827 (108)
SYN35079 (132.6)	SYN35079 (109.5)
PZE−107078209 (133.1)	PZE−107078209 (110.7)
PUT−163a−71300000−3068 (133.9)	PUT−163a−71300000−3068 (111.3)
PZE−107079545 (134.1)	PZE−107079545 (112.7)
PZE−107080136 (135.2)	PZE−107080136 (113.1)
PZE−107080365 (135.5)	PZE−107082807 (113.5)
PZE−107080829 (135.8)	PZE−107082348 (113.9)
PZB00752.1 (136.9)	PZB00752.1 (114.4)
PZE−107082348 (137.3)	PZE−107080829 (115.3)
PZE−107082807 (137.5)	PZE−107080365 (115.7)
PZA00740.3 (139.6)	PZA00740.3 (116.5)
PZE−107084721 (140.4)	PZE−107084721 (117.5)
PZE−107085598 (141.4)	PZE−107085598 (117.8)
SYN6941 (141.7)	SYN6941 (118.1)
PZE−107087168 (142.4)	PZE−107087168 (118.5)
PZE−107087898 (143)	PZE−107087898 (119.5)
SYN24395 (143.4)	SYN24395 (119.9)
PZA−000405002 (144.1)	PZA−000405002 (120.7)
PZE−107089479 (144.5)	PZE−107089479 (121.2)
PZE−107090494 (145.7)	PZE−107090494 (121.9)
PZA03574.3 (146.9)	PZA03574.3 (123.2)
PUT−163a−16919428−1017 (148.2)	PUT−163a−16919428−1017 (123.9)
PZE−107094380 (149.7)	PZE−107094380 (124.2)
PUT−163a−60357313−2828 (151.7)	PUT−163a−60357313−2828 (124.7)
PZE−107097554 (152.8)	PZE−107097554 (125.5)
PZE−107098865 (153.8)	PZE−107098865 (126.3)
PZE−107099083 (154.1)	PZE−107099083 (126.9)
PUT−163a−60395621−2934 (154.5)	PUT−163a−60395621−2934 (127.6)
PZE−107099933 (154.6)	PZE−107099933 (128.3)
PUT−163a−28576072−1630 (155.6)	PUT−163a−28576072−1630 (128.8)
SYN18508 (156.2)	SYN18508 (129.6)
PZA02959.12 (157.3)	PZA02959.12 (130)
PZE−107104470 (157.5)	PZE−107104470 (130.6)
PZE−107105855 (157.9)	PZE−107105855 (131.4)
PZE−107106666 (158.5)	PZE−107106666 (132.5)
SYN5313 (158.9)	SYN5313 (133.4)
PUT−163a−14244132−307 (159.7)	PUT−163a−14244132−307 (133.9)
PZE−107108840 (160)	PZE−107108840 (135.7)
SYN14115 (160.6)	SYN14115 (136.6)
PZE−107110443 (160.6)	PZE−107110443 (138.1)
PZE−107110616 (160.8)	PZE−107110616 (139.6)
PZE−107110984 (161.1)	PZE−107110984 (140.1)
PZE−107113353 (162.1)	PZE−107113353 (147.8)
PZE−107113723 (162.3)	PZE−107113723 (148.6)
SYN1455 (162.3)	SYN1455 (149.4)
PZA03544.3 (162.8)	PZA03544.3 (150.1)
PZE−107115307 (163)	PZE−107115307 (150.7)
SYN12221 (163.6)	SYN12221 (151.3)
SYNGENTA14901 (164.1)	SYNGENTA14901 (154)
SYN3390 (164.7)	SYN3390 (154.8)
PZE−107119102 (165.4)	PZE−107119102 (155.8)
PZE−107119249 (165.5)	PZE−107119249 (157.8)
SYN13029 (165.5)	SYN13029 (158.3)
PZE−107120325 (165.8)	PZE−107120325 (159.9)
ZM001788−1266 (166.1)	ZM001788−1266 (160.8)
PZE−107122066 (167)	PZE−107122066 (163.7)
ZM013370−0333 (167.2)	ZM013370−0333 (165.7)
PZE−107123600 (167.7)	PZE−107123600 (167.3)
PZE−107124659 (168.1)	PZE−107124659 (169.7)
SYN34049 (168.5)	SYN34049 (171)
SYN17014 (169.2)	SYN17014 (171.5)
SYN30790 (169.5)	SYN30790 (171.7)
PZE−107127708 (170.2)	PZE−107127708 (171.9)
PZE−107128145 (170.4)	PZE−107128145 (173)
PZA00695.1 (171.2)	PZA00695.1 (175.9)
PUT−163a−78085872−4180 (171.7)	PUT−163a−78085872−4180 (177.4)
PZE−107130802 (171.9)	PZE−107130802 (179)
PZE−107131443 (172.4)	PZE−107131443 (180.6)
PUT−163a−94475607−4877 (172.6)	PUT−163a−94475607−4877 (182.8)
PZE−107132828 (172.8)	PZE−107132828 (183.6)
SYN32824 (173)	SYN32824 (185.1)
SYN4570 (173.3)	SYN4570 (185.9)
PZE−107134294 (173.4)	PZE−107134294 (186.6)
SYN37419 (173.8)	SYN37419 (190.1)
PZE−107136092 (174.3)	PZE−107136092 (192)
PUT−163a−60398021−2968 (174.4)	PUT−163a−60398021−2968 (193.2)
PZE−107137037 (174.8)	PZE−107137037 (194.4)
PZE−107137587 (175.2)	PZE−107137587 (195.7)

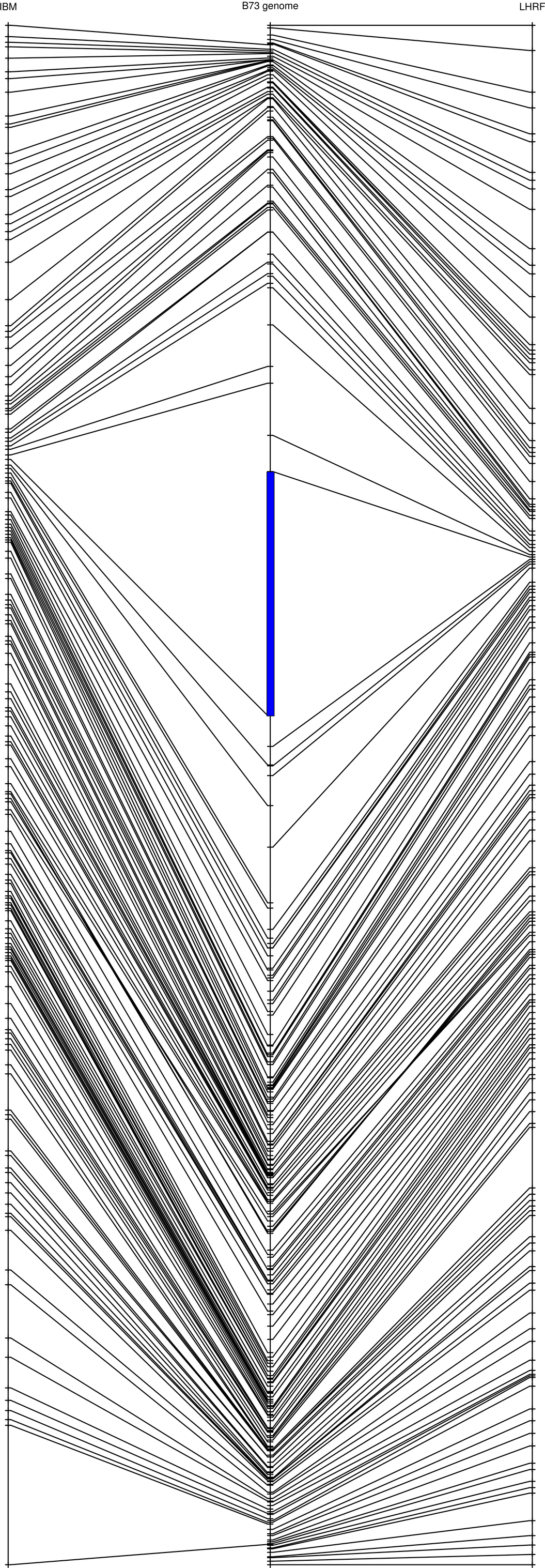

Chrom 8

IBM map (cM)	B73 genome (Mbp)
PZE−108000683 (0)	PZE−108000683 (0.8)
SYN25099 (2.2)	SYN25099 (1.4)
SYN25092 (3.1)	SYN25092 (1.5)
PUT−163a−13557719−256 (3.6)	PUT−163a−13557719−256 (1.8)
SYN11271 (4.6)	SYN11271 (2.4)
SYN10808 (5.1)	SYN10808 (2.5)
PZE−108002846 (9.5)	PZE−108002846 (3)
SYN17938 (11.4)	SYN17938 (3.3)
SYN33566 (12.1)	SYN33566 (3.8)
PZA−002174002 (12.4)	PZA−002174002 (4.2)
PZE−108004350 (16.9)	PZE−108004350 (4.4)
PZE−108004388 (17.9)	PZE−108004388 (4.4)
SYN12532 (18.5)	SYN12532 (4.8)
SYN12530 (20.1)	SYN12530 (4.8)
SYN34214 (21.3)	SYN34214 (5.1)
PZE−108005219 (22.1)	PZE−108005219 (5.3)
PZE−108005605 (23.8)	PZE−108005605 (5.6)
PUT−163a−71445680−3382 (24.6)	PUT−163a−71445680−3382 (5.9)
SYN3883 (25)	SYN3883 (6)
SYN35223 (25.5)	SYN35223 (6.4)
PZE−108006221 (25.8)	PZE−108006221 (6.5)
SYN10427 (26)	SYN10427 (6.6)
SYN13071 (26.2)	SYN13071 (6.9)
PZE−108007013 (27.2)	PZE−108007013 (7)
SYN20700 (29.9)	SYN20700 (7.7)
SYN11622 (30.4)	SYN11622 (8.3)
PZE−108008403 (31.8)	PZE−108008403 (8.4)
PZE−108008540 (32.1)	PZE−108008540 (8.6)
PZE−108008825 (33.5)	PZE−108008825 (8.7)
PZE−108009251 (34.5)	PZE−108009251 (9.9)
SYN12761 (34.9)	SYN12761 (10.1)
SYN12758 (35.4)	SYN12758 (10.1)
SYN36558 (35.8)	SYN36558 (10.8)
SYN36560 (36)	SYN36560 (10.9)
PZE−108010702 (36.5)	PZE−108010702 (11.3)
PZE−108010981 (37.6)	PZE−108010981 (11.5)
PZE−108011830 (39.6)	PZE−108011830 (12.2)
SYN3396 (40.7)	SYN3396 (12.6)
PZE−108012841 (41.5)	PZE−108012841 (13)
PZE−108013234 (42.2)	PZE−108013234 (13.3)
SYN14462 (43)	SYN14462 (13.4)
PZE−108013757 (44)	PZE−108013757 (13.6)
SYN18494 (44.2)	SYN18494 (13.9)
SYN35072 (45)	SYN35072 (15.1)
PZE−108016244 (46.8)	PZE−108016244 (16)
SYN23097 (47.7)	SYN23097 (16.4)
PZE−108017417 (49.7)	PZE−108017417 (17.1)
SYN19462 (50.6)	SYN19462 (17.2)
PZE−108019790 (51.5)	PZE−108019790 (17.9)
PZE−108018485 (52.6)	PZE−108018485 (19)
PZE−108021167 (53.1)	PZE−108021167 (20.1)
PZE−108021327 (54.2)	PZE−108021327 (20.5)
PZE−108021864 (56.1)	PZE−108021864 (20.7)
SYN17577 (57.5)	SYN17577 (21.1)
SYN35861 (57.8)	SYN35861 (22.2)
PZE−108023549 (58.4)	PZE−108023549 (22.5)
PZE−108023615 (59.3)	PZE−108023615 (22.7)
PZE−108024348 (60.3)	PZE−108024348 (23)
SYN38349 (60.6)	SYN38349 (23.1)
SYN15118 (61.1)	SYN15118 (24)
SYN19605 (61.6)	SYN19605 (24.4)
PZA03510.1 (61.8)	PZA03510.1 (26.6)
PZE−108027738 (62)	PZE−108027738 (29.1)
PZE−108030157 (62.2)	PZE−108030157 (33.8)
PZE−108030869 (62.4)	PZE−108030869 (34.7)
PZE−108032283 (62.6)	PZE−108032283 (37.6)
PZA−000717015 (63)	PZA−000717015 (70.2)
PZE−108042761 (63.9)	PZE−108042761 (71)
SYN12525 (65)	SYN12525 (72)
PZE−108043501 (65.4)	PZE−108043501 (72.4)
SYN2884 (66)	SYN2884 (72.9)
PZE−108045907 (67.5)	PZE−108045907 (75.6)
PZE−108050016 (68.3)	PZE−108050016 (87)
SYN30907 (68.9)	SYN30907 (90.7)
PZE−108052051 (69.3)	PZE−108052051 (92.1)
PZE−108053704 (70.1)	PZE−108053704 (95.7)
PZE−108054215 (71.1)	PZE−108054215 (96.3)
PZE−108056020 (72)	PZE−108056020 (100.9)
SYN17876 (73.3)	SYN17876 (101.2)
SYN30221 (75.3)	SYN30221 (101.8)
PZE−108058161 (76.4)	PZE−108058161 (103.7)
SYN27264 (76.9)	SYN27264 (104.6)
PZE−108059057 (77.5)	PZE−108059057 (105.3)
PZE−108059739 (77.9)	PZE−108059739 (106.7)
PZA03508.1 (78.1)	PZA03508.1 (107.1)
PZE−108061313 (78.5)	PZE−108061313 (109.9)
PUT−163a−78092237−4250 (80.6)	PUT−163a−78092237−4250 (111)
PZE−108062506 (81.1)	PZE−108062506 (111.7)
PZE−108063794 (81.6)	PZE−108063794 (113.9)
PZE−108065075 (82.3)	PZE−108065075 (116.3)
PZE−108065744 (82.6)	PZE−108065744 (117.4)
PZE−108066339 (83.4)	PZE−108066339 (118.1)
SYN27931 (84.2)	SYN27931 (118.2)
SYN16160 (84.6)	SYN16160 (118.8)
SYN12736 (84.9)	SYN12736 (119)
PZE−108067569 (85.3)	PZE−108067569 (119.3)
PZE−108069441 (86.1)	PZE−108069441 (121.9)
PUT−163a−74234257−3606 (86.9)	PUT−163a−74234257−3606 (123)
PZE−108070380 (87.7)	PZE−108070380 (123.5)
PZE−108071079 (88.8)	PZE−108071079 (124.4)
SYN32800 (89.7)	SYN32800 (124.8)
SYN15433 (90)	SYN15433 (125.3)
PZE−108073569 (90.3)	PZE−108073569 (128.5)
PZE−108073924 (90.6)	PZE−108073924 (129.1)
SYN11836 (91.4)	SYN11836 (131.3)
SYN24931 (91.7)	SYN24931 (132.9)
SYN18385 (92.1)	SYN18385 (133.2)
PZE−108077786 (92.3)	PZE−108077786 (133.4)
PUT−163a−71423233−3216 (92.6)	PUT−163a−71423233−3216 (133.9)
PZE−108078255 (92.9)	PZE−108078255 (134)
SYN16251 (93.3)	SYN16251 (135.5)
PZE−108080736 (93.7)	PZE−108080736 (137.1)
SYN2532 (94)	SYN2532 (138.6)
SYN20177 (94.3)	SYN20177 (139.1)
SYN38887 (94.8)	SYN38887 (141.5)
PZE−108107072 (104.9)	PZE−108107072 (161.2)
SYN15878 (107.7)	SYN15878 (161.6)
PZE−108108666 (108.4)	PZE−108108666 (162.2)
PZE−108109498 (111.8)	PZE−108109498 (162.6)
SYN13362 (112.5)	SYN13362 (162.8)
SYN14062 (113.3)	SYN14062 (163.3)
PZE−108111762 (114.5)	PZE−108111762 (164)
SYN23758 (114.9)	SYN23758 (164.2)
PZE−108112124 (115.1)	PZE−108112124 (164.2)
SYN23212 (115.7)	SYN23212 (164.4)
PZE−108112715 (115.9)	PZE−108112715 (164.6)
SYN22946 (116.4)	SYN22946 (164.7)
SYN30178 (117.3)	SYN30178 (164.9)
SYN32177 (120.2)	SYN32177 (165.3)
SYN4434 (120.5)	SYN4434 (165.5)
PZE−108115325 (121.1)	PZE−108115325 (165.6)
PZE−108116213 (122.6)	PZE−108116213 (166)
SYN12100 (122.9)	SYN12100 (166.5)
PZE−108117199 (123.9)	PZE−108117199 (166.6)
PZE−108117143 (124.8)	PZE−108117143 (166.7)
SYN31086 (125.1)	SYN31086 (166.7)
PZE−108117488 (125.8)	PZE−108117488 (166.7)
PZE−108118398 (128.7)	PZE−108118398 (167.1)
PZE−108118488 (129.1)	PZE−108118488 (167.2)
SYN21092 (129.4)	SYN21092 (167.2)
PZE−108119072 (129.7)	PZE−108119072 (167.4)
SYN29149 (131.1)	SYN29149 (167.6)
SYN29240 (132)	SYN29240 (168.1)
SYN20446 (132.3)	SYN20446 (168.3)
SYN36464 (132.8)	SYN36464 (168.9)
SYN36468 (133.4)	SYN36468 (169)
PUT−163a−76010623−3722 (134.4)	PUT−163a−76010623−3722 (169.1)
SYN25129 (135)	SYN25129 (169.1)
SYN21960 (137.9)	SYN21960 (169.7)
PZE−108124414 (139.5)	PZE−108124414 (170.1)
SYN23844 (141)	SYN23844 (170.3)
PUT−163a−18173735−1405 (141.9)	PUT−163a−18173735−1405 (170.3)
SYN36002 (142.7)	SYN36002 (170.7)
SYN36001 (143.8)	SYN36001 (170.8)
PZE−108126650 (144.9)	PZE−108126650 (170.9)
PZE−108126856 (145.7)	PZE−108126856 (171)
PZA00189.23 (146.8)	PZA00189.23 (171.3)
SYN4414 (149)	SYN4414 (171.7)
PUT−163a−71311544−3116 (151.1)	PUT−163a−71311544−3116 (172.4)
SYN14957 (151.7)	SYN14957 (172.4)
SYN15043 (152.4)	SYN15043 (172.8)
SYN4926 (152.8)	SYN4926 (172.9)
SYN4936 (153)	SYN4936 (173)
SYN4930 (153.4)	SYN4930 (173)
SYN11404 (154.1)	SYN11404 (173.3)
PZA02281.3 (155.1)	PZA02281.3 (173.4)
SYN36819 (155.8)	SYN36819 (173.5)
PZE−108133242 (156.7)	PZE−108133242 (173.6)
SYN10417 (157)	SYN10417 (173.7)
SYN10423 (159.4)	SYN10423 (173.8)
PZE−108133741 (161.1)	PZE−108133741 (173.8)
SYN36374 (162.6)	SYN36374 (173.9)
SYN29575 (165.9)	SYN29575 (174.2)
SYN20808 (167.5)	SYN20808 (174.4)
SYN20810 (168)	SYN20810 (174.4)

B73 genome (Mbp)	LHRF map (cM)
PZE−108000108 (0.2)	PZE−108000108 (0)
ZM004820−0602 (1.7)	ZM004820−0602 (5.5)
PZE−108002154 (2.2)	PZE−108002154 (6.8)
PUT−163a−60337233−2343 (2.5)	PUT−163a−60337233−2343 (8.6)
PZE−108002634 (2.8)	PZE−108002634 (11.7)
PZE−108003557 (3.5)	PZE−108003557 (13.1)
PZE−108003848 (3.9)	PZE−108003848 (15)
PZA−002174002 (4.2)	PZA−002174002 (16.6)
PZE−108004260 (4.3)	PZE−108004260 (25.3)
PZE−108004350 (4.4)	PZE−108004350 (27.4)
SYN3177 (4.5)	SYN3177 (32.9)
SYN3883 (6)	SYN3883 (40.3)
SYN35223 (6.4)	SYN35223 (41.8)
PZE−108007014 (7)	PZE−108007014 (43.2)
PZE−108007792 (8)	PZE−108007792 (44.6)
PZE−108008403 (8.4)	PZE−108008403 (47.7)
PZE−108009594 (10.1)	PZE−108009594 (51.8)
PZE−108010327 (10.9)	PZE−108010327 (53.8)
SYN3396 (12.6)	SYN3396 (55.3)
PZE−108012916 (13)	PZE−108012916 (56.3)
PZE−108013234 (13.3)	PZE−108013234 (57)
SYN14462 (13.4)	SYN14462 (58.3)
PZE−108013853 (13.8)	PZE−108013853 (59.6)
PZE−108015266 (14.8)	PZE−108015266 (61.4)
PZE−108015742 (15.3)	PZE−108015742 (62.2)
SYN34055 (16)	SYN34055 (62.9)
PZE−108016518 (16.3)	PZE−108016518 (63.7)
PZE−108016983 (16.8)	PZE−108016983 (64.8)
PZB01977.4 (17.4)	PZB01977.4 (68.5)
PZE−108019463 (18.2)	PZE−108019463 (70)
PZE−108019174 (18.4)	PZE−108019174 (70.8)
PZE−108018598 (18.9)	PZE−108018598 (72.5)
PZE−108020151 (19.1)	PZE−108020151 (73)
PZE−108020539 (19.7)	PZE−108020539 (73.4)
PZE−108020824 (19.8)	PZE−108020824 (74.3)
PZE−108022448 (21.3)	PZE−108022448 (76.4)
PZE−108022547 (21.4)	PZE−108022547 (76.8)
SYN11424 (21.8)	SYN11424 (77.2)
PZE−108023549 (22.5)	PZE−108023549 (78)
PUT−163a−78080238−4136 (23.8)	PUT−163a−78080238−4136 (78.9)
PZE−108025294 (24)	PZE−108025294 (80.3)
PZA00481.7 (24.5)	PZA00481.7 (82)
PUT−163a−89249801−4641 (25)	SYN31342 (82.4)
SYN31342 (25.1)	PUT−163a−89249801−4641 (82.7)
PZE−108028588 (26.3)	PZE−108028588 (83.3)
PZE−108028852 (26.6)	PZE−108028852 (83.9)
PZE−108026930 (27.5)	PZE−108026930 (84.5)
PZE−108030620 (34.6)	PZE−108030620 (85.1)
PZE−108032072 (37.2)	PZE−108032072 (85.3)
PZA00417.2 (45.3)	PZA00417.2 (86)
PZA00793.2 (66.1)	PZA00793.2 (86.6)
PZE−108041860 (68.3)	PZE−108042796 (87.8)
PZE−108042796 (71.1)	PZE−108041860 (88.3)
SYN12525 (72)	SYN12525 (88.5)
PZE−108043954 (73)	PZE−108043954 (89.4)
PZE−108045495 (75.3)	PZE−108045495 (90)
PUT−163a−110964923−135 (77.8)	PUT−163a−110964923−135 (90.7)
PZE−108048129 (81.1)	PZE−108048129 (91.1)
SYN17845 (88)	SYN17845 (91.7)
PZE−108051282 (90.4)	PZE−108051282 (92.1)
SYN30907 (90.7)	SYN30907 (92.4)
PZE−108051664 (91.2)	PZE−108051664 (92.9)
PZE−108051965 (91.8)	PZE−108052788 (93.7)
PZE−108052788 (93.9)	PZE−108051965 (94.1)
PZE−108053629 (95.6)	PZE−108053629 (94.4)
PUT−163a−71430615−3278 (96.1)	PUT−163a−71430615−3278 (94.7)
PZE−108054157 (96.3)	PZE−108054157 (96.1)
PZE−108055572 (100)	PZE−108055572 (97)
PZE−108055835 (100.8)	PZE−108055835 (97.6)
PZE−108056400 (101.2)	PZE−108056400 (99.5)
PUT−163a−88754682−4575 (103.3)	PUT−163a−88754682−4575 (100.7)
PZE−108058655 (104.6)	PZE−108058655 (101.7)
PZE−108058793 (104.8)	PZE−108058793 (102.3)
PZE−108059416 (106.2)	PZE−108059416 (102.7)
SYN39344 (106.3)	SYN39344 (103)
PZE−108059500 (106.3)	PZE−108059500 (103.7)
PZE−108061313 (109.9)	PZE−108061313 (104.9)
PZE−108061670 (110.4)	PZE−108061670 (105.9)
PUT−163a−18162161−1219 (112.5)	PUT−163a−18162161−1219 (108.1)
PUT−163a−50324981−2175 (116.9)	PUT−163a−50324981−2175 (109.2)
PZE−108065562 (117.2)	PZE−108065562 (109.5)
SYN16154 (118.8)	SYN16154 (110.8)
PZA03637.3 (119.8)	PZA03637.3 (112.6)
PZE−108069627 (122.2)	PZE−108069627 (114.5)
PZE−108070762 (123.8)	PZE−108070762 (115.7)
PZB02159.1 (124.7)	PZB02159.1 (116.3)
PZE−108071856 (124.8)	PZE−108071856 (116.7)
PZA00118.1 (127.5)	PZA00118.1 (117.3)
PZE−108075118 (130.7)	PZE−108075118 (117.8)
PZE−108077298 (132.9)	PZE−108077298 (118.7)
PZE−108077632 (133.3)	PZE−108077632 (119.3)
PUT−163a−16922828−1078 (134.5)	PUT−163a−16922828−1078 (120)
SYN14198 (134.7)	SYN14198 (120.3)
PZE−108080497 (136.7)	PZE−108080497 (121)
PZE−108081591 (138.5)	PZE−108081591 (121.5)
PZE−108082591 (139.3)	PZE−108082591 (122.2)
PUT−163a−71298926−3055 (140.5)	PUT−163a−71298926−3055 (123.8)
PUT−163a−16925878−1123 (142.3)	PUT−163a−16925878−1123 (125)
PZE−108088139 (145.5)	PZE−108088139 (127)
PUT−163a−16919569−1033 (146.9)	PUT−163a−16919569−1033 (131)
PZE−108091433 (148.5)	PZE−108091433 (133.1)
PUT−163a−60346455−2552 (150.9)	PUT−163a−60346455−2552 (134.2)
PZE−108094362 (151.2)	PZE−108094362 (134.7)
PZE−108094590 (151.3)	PZE−108094590 (135.1)
PZE−108095959 (152.3)	PZE−108095959 (136.5)
PZE−108097440 (153.1)	PZE−108097440 (137.1)
PZE−108098580 (155)	PZE−108098580 (137.8)
PZE−108099415 (155.6)	PZE−108099415 (140.5)
PZE−108100275 (156.4)	PZE−108100275 (141.5)
PZA03695.1 (156.9)	PZA03695.1 (142.5)
PZE−108101499 (157.4)	PZE−108101499 (143.8)
PUT−163a−71425880−3232 (158)	PUT−163a−71425880−3232 (147)
PZE−108103193 (159)	PZE−108103193 (147.7)
PZE−108103787 (159.3)	PZE−108103787 (148.7)
PZE−108104106 (159.4)	PZE−108104106 (149.4)
PZE−108104589 (159.7)	PZE−108104589 (150.3)
PUT−163a−149101061−928 (160.4)	PUT−163a−149101061−928 (152.6)
PZE−108108062 (161.7)	PZE−108108062 (154.6)
PUT−163a−60349565−2618 (162.5)	PUT−163a−60349565−2618 (155.7)
PZA03593.1 (163.1)	PZA03593.1 (157.4)
PZE−108111057 (163.7)	PZE−108111057 (159.4)
PZE−108111470 (164)	PZE−108111470 (160.1)
PZE−108111762 (164)	PZE−108111762 (161.2)
SYN23758 (164.2)	SYN23758 (162.3)
SYN23220 (164.4)	SYN23220 (163.4)
PUT−163a−78108031−4276 (164.9)	PUT−163a−78108031−4276 (164.9)
SYN30179 (164.9)	SYN30179 (165.4)
PZE−108113861 (165)	PZE−108113861 (165.9)
PZE−108114666 (165.3)	PZE−108114666 (168)
SYN23809 (165.4)	SYN23809 (168.9)
PZA00001.8 (166.1)	PZA00001.8 (171.6)
PZE−108117143 (166.7)	PZE−108117143 (174)
PZE−108118488 (167.2)	PZE−108118488 (177.4)
SYN36533 (168)	SYN36533 (180.4)
PZE−108120391 (168.3)	PZE−108120391 (181.1)
SYN36464 (168.9)	SYN36464 (184.5)
PUT−163a−74233701−3600 (169.1)	PUT−163a−74233701−3600 (185.3)
PZE−108123038 (169.5)	PZE−108123038 (187)
SYN34541 (169.8)	SYN34541 (191.9)
PZE−108125652 (170.4)	PZE−108125652 (196.4)

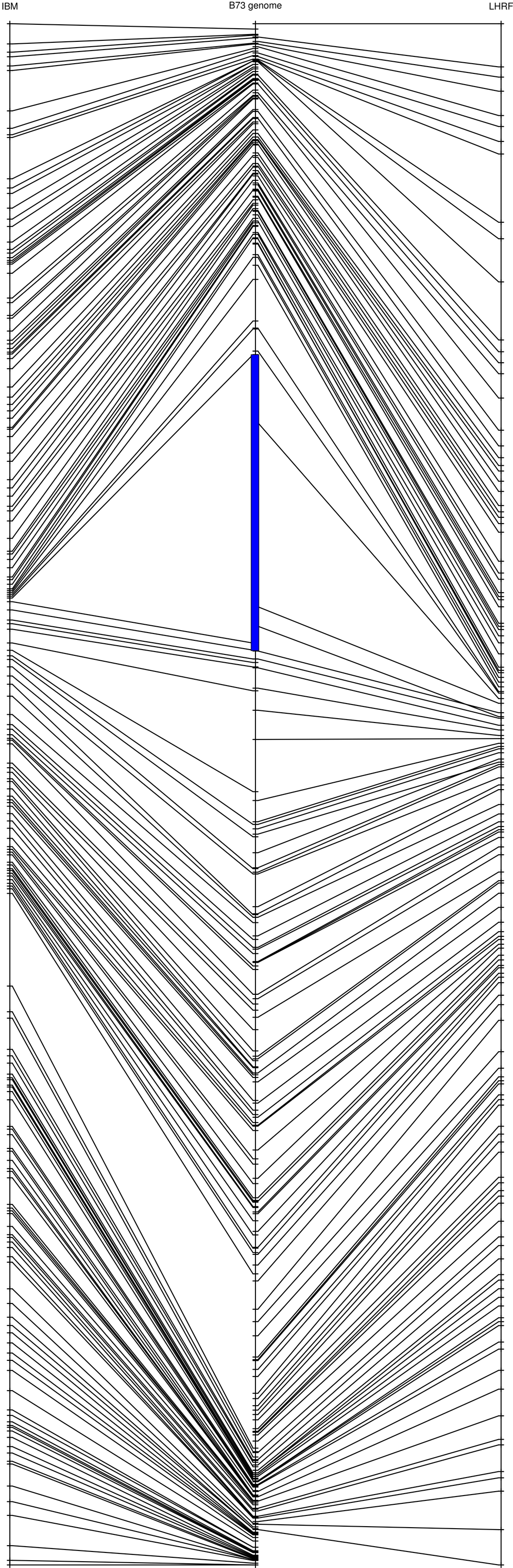

Chrom 9

IBM map (cM) B73 genome (Mbp)

PZE−109000143 (0) PZE−109000143 (0.6)
SYN21732 (2) SYN21732 (1.3)
PZE−109001536 (3.2) PZE−109001536 (2.1)
SYN25586 (3.5) SYN25586 (2.2)
SYN24557 (3.8) SYN24557 (2.3)
SYN36188 (4.7) SYN36188 (3.1)
PZE−109003046 (6) PZE−109003046 (3.7)
PZE−109003305 (7.4) PZE−109003305 (3.8)
PZE−109003659 (7.7) PZE−109003659 (4.3)
PZE−109003902 (8.3) PZE−109003902 (4.4)
SYN30861 (10) SYN30861 (4.7)
SYN35081 (14.8) SYN35081 (6.1)
PZE−109006201 (17.1) PZE−109006201 (7.1)
PZE−109006992 (19.1) PZE−109006992 (7.8)
SYN39069 (20.9) SYN39069 (8)
SYN39073 (21.4) SYN39073 (8)
SYN13447 (22.7) SYN13447 (8.4)
SYN19752 (24.6) SYN19752 (8.8)
SYN20011 (26.4) SYN20011 (9)
SYN36359 (27) SYN36359 (9.3)
SYN11737 (28) SYN11737 (10)
PZE−109009392 (29.8) PZE−109009392 (10.3)
PZE−109009763 (31.7) PZE−109009763 (10.8)
PZE−109010476 (33.3) PZE−109010476 (11.4)
SYN33212 (34) SYN33212 (11.7)
PZE−109011385 (34.8) PZE−109011385 (11.8)
SYN2758 (36.8) SYN2758 (12.9)
SYN18843 (37.7) PZE−109012555 (13)
PZE−109012555 (38.2) SYN18843 (13.2)
PZE−109013000 (38.6) PZE−109013000 (13.5)
SYN35320 (39.8) SYN35320 (13.5)
PZE−109013469 (40.5) PZE−109013469 (13.8)
SYN12315 (41.6) SYN12315 (14.3)
SYN11769 (43) SYN11769 (14.5)
SYN27750 (43.4) SYN27750 (14.8)
PZE−109015133 (44.3) PZE−109015133 (15.3)
PZE−109015314 (46) PZE−109015314 (15.4)
PUT−163a−16926106−1129 (46.9) PUT−163a−16926106−1129 (16)
PZE−109016177 (48.3) PZE−109016177 (16.3)
SYN16077 (50.7) SYN16077 (16.7)
PZE−109016955 (51.7) PZE−109016955 (17)
PZE−109017457 (52.5) PZE−109017457 (17.6)
PZE−109018303 (54.6) PZE−109018303 (18.7)
PZE−109018613 (55.4) PZE−109018613 (18.9)
SYN18694 (55.7) SYN18694 (19.8)
PZE−109019686 (56.1) PZE−109019686 (20.1)
SYN29881 (58.9) SYN29881 (20.8)
SYN15749 (60.1) SYN15749 (21)
PZE−109021713 (61) PZE−109021713 (22)
PZE−109022427 (61.3) PZE−109022427 (22.8)
SYN34183 (62.4) SYN34183 (23.7)
PZB01963.1 (62.9) PZB01963.1 (24.5)
SYN28207 (64.2) SYN28207 (25.3)
SYN34135 (64.6) SYN34135 (26.6)
SYN34141 (64.9) SYN34141 (26.6)
PZE−109026940 (65.5) PZE−109026940 (26.9)
PZE−109027147 (65.8) PZE−109027147 (27.1)
PZE−109027723 (66) PZE−109027723 (28.4)
PZE−109029553 (66.6) PZE−109029553 (32.3)
PZE−109035852 (66.8) PZE−109035852 (47.1)
PZE−109037900 (67.1) PZE−109037900 (55.5)
PZE−109047031 (67.3) PZE−109047031 (81.2)
SYN38486 (67.9) SYN38486 (87.6)
PZE−109051446 (68.1) PZE−109051446 (88.9)
PZE−109051855 (68.4) PZE−109051855 (89.6)
PZE−109054975 (68.8) PZE−109054975 (95.6)
PZE−109055553 (70.1) PZE−109055553 (96.2)
PZE−109057545 (71.1) PZE−109057545 (98.7)
PZE−109058129 (71.7) PZE−109058129 (99.4)
PZE−109060496 (72.2) PZE−109060496 (102.1)
SYN37647 (72.9) SYN37647 (105.1)
SYN37617 (73.4) SYN37617 (105.4)
SYN36472 (73.7) SYN36472 (105.8)
PZE−109064332 (74) PZE−109064332 (107.4)
PZE−109064885 (74.3) PZE−109064885 (108)
PZE−109066337 (74.9) PZE−109066337 (109.3)
SYN21230 (76) SYN21230 (110.3)
PZE−100001123 (76.2) PZE0003716573 (111.1)
PZE0003716573 (76.4) PZE−100001123 (111.3)
PZE−109068561 (77) PZE−109068561 (112.8)
PZE−109069697 (77.2) PZE−109069697 (113.8)
SYN18130 (78.2) SYN18130 (114.1)
SYN29013 (78.9) SYN29013 (117.3)
PZE−109073134 (79.7) PZE−109073134 (118.3)
SYN27246 (80.5) SYN27246 (119.7)
SYN22541 (80.8) SYN22541 (120.3)
SYN4637 (81) SYN4637 (121.6)
SYN14344 (81.7) SYN14344 (123.2)
PZE−109077509 (82) PZE−109077509 (125)
PZE−109078597 (83.5) PZE−109078597 (126.7)
PZE−109079358 (84) PZE−109079358 (127.4)
SYN24353 (84.5) SYN24353 (128.2)
SYN12671 (85) SYN12671 (129)
SYN3443 (85.5) SYN3443 (130.5)
PZE−109082594 (85.9) PZE−109082594 (131.2)
PZA01866.1 (88) PZA01866.1 (133.9)
PZE−109085093 (88.3) PZE−109085093 (133.9)
PZE−109085594 (88.6) PZE−109085594 (134.3)
PZE−109086097 (89) PZE−109086097 (134.6)
SYN38163 (89.4) SYN38163 (135.9)
SYN38160 (89.8) SYN38160 (135.9)
PZE−109087484 (90.2) PZE−109087484 (136.1)
SYN31610 (90.9) SYN31610 (136.2)
PZE−109088452 (91.2) PZE−109088452 (136.8)
SYN24722 (91.9) SYN24722 (137.1)
PZE−109089873 (92.6) PZE−109089873 (137.8)
PZE−109091377 (94.5) PZE−109091377 (138.7)
SYN4010 (95.4) SYN4010 (138.9)
PZE−109093491 (98) PZE−109093491 (140)
SYN12940 (98.4) SYN12940 (140.3)
SYN32361 (99.1) SYN32361 (140.8)
SYN11597 (100.7) SYN11597 (141.5)
SYN11596 (101) SYN11596 (141.5)
SYN26727 (102.1) SYN26727 (141.8)
SYN16109 (102.4) SYN16109 (141.9)
SYN23501 (102.8) SYN23501 (142.5)
PZE−109097202 (103.4) PZE−109097202 (142.8)
SYN30940 (104.7) SYN30940 (142.9)
PZE−109098496 (106.9) PZE−109098496 (143.4)
SYN27712 (107.7) SYN27712 (143.9)
SYN27711 (108) SYN27711 (143.9)
SYN22241 (109.9) SYN22241 (144.8)
PZE−109100755 (110.5) PZE−109100755 (145)
SYN25124 (111.9) SYN25124 (145.4)
PZE−109102157 (112.2) PZE−109102157 (146.2)
SYN30124 (114.5) SYN30124 (146.5)
PZE−109102962 (115.6) PZE−109102962 (146.5)
PZA02197.1 (117) PZA02197.1 (146.9)
SYN26894 (117.5) SYN26894 (147.5)
SYN10263 (119.2) SYN10263 (147.8)
PZE−109106186 (119.9) PZE−109106186 (148.1)
PZE−109107497 (120.7) PZE−109107497 (149.1)
PZE−109108255 (121.6) PZE−109108255 (149.5)
PZE−109109275 (125.8) PZE−109109275 (149.9)
PZE−109109692 (127.8) PZE−109109692 (150.4)
PZE−109109991 (128.8) PZE−109109991 (150.8)
PZE−109110253 (129.9) PZE−109110253 (150.9)
SYN5447 (130.9) SYN5447 (151.2)
PZE−109111133 (133.5) PZE−109111133 (151.3)
SYN35714 (135.3) SYN35714 (151.5)
PUT−163a−4621424−2088 (136.4) PUT−163a−4621424−2088 (151.7)
PZE−109113507 (137) PZE−109113507 (151.8)
SYN10591 (138.8) SYN10591 (152)
PUT−163a−50330000−2208 (141.8) PUT−163a−50330000−2208 (152.6)
PZE−109115897 (143.1) PZE−109115897 (152.8)
SYN6090 (143.6) SYN6090 (153.2)
PZE−109116477 (145.2) PZE−109116477 (153.2)
SYN24345 (146.7) SYN24345 (153.4)
PZE−109116876 (147.1) PZE−109116876 (153.4)
SYN24330 (148.6) SYN24330 (153.5)
SYN24328 (150) SYN24328 (153.6)
SYN15946 (150.3) SYN15946 (153.7)
SYN15948 (152.3) SYN15948 (153.7)
SYN13929 (153.1) SYN13929 (153.8)
SYN13953 (154.7) SYN13953 (154.1)
PZE−109119946 (155.7) PZE−109119946 (154.3)
PUT−163a−16922936−1081 (157.8) PUT−163a−16922936−1081 (154.6)

B73 genome (Mbp) LHRF map (cM)

PZE−109000122 (0.6) PZE−109000122 (0)
SYN21747 (1.3) SYN21747 (3.3)
PZE−109001669 (2.2) PZE−109001669 (5.3)
PZE−109001902 (2.3) PZE−109001902 (6.4)
PZE−109002005 (2.5) PZE−109002005 (7.1)
PZE−109002219 (2.6) PZE−109002219 (8.1)
PZE−109003131 (3.7) PZE−109003131 (11.2)
PZE−109003647 (4.3) PZE−109003647 (11.9)
PZE−109004946 (5.5) PZE−109004946 (14.7)
PZE−109005409 (6) PZE−109005409 (17.4)
PZE−109005850 (6.5) PZE−109005850 (18.3)
PZE−109006355 (7.2) PZE−109006355 (20.3)
SYN32602 (7.6) SYN32602 (20.8)
PZE−109006938 (7.7) PZE−109006938 (21.3)
PZE−109007075 (7.9) PZE−109007075 (23.4)
SYN36360 (9.4) SYN36360 (27.7)
PZE−109009220 (10) PZE−109009220 (28.4)
PZE−109009836 (10.9) PZE−109009836 (31)
PZE−109010252 (11.2) PZE−109010252 (31.6)
SYNGENTA10406 (11.5) SYNGENTA10406 (32.6)
PUT−163a−6031569−2337 (11.7) PUT−163a−6031569−2337 (34.2)
PZE−109011840 (12.6) PZE−109011840 (36)
SYN2755 (12.9) SYN2755 (37.1)
PZE−109012939 (13.4) PZE−109012939 (40.2)
SYN35320 (13.5) SYN35320 (41.4)
PZE−109013674 (13.9) PZE−109013674 (42.8)
PZE−109014867 (14.8) PZE−109014867 (45.3)
SYN32732 (15.6) SYN32732 (45.8)
PUT−163a−16926106−1128 (16) PUT−163a−16926106−1128 (46.6)
PZE−109016150 (16.3) PZE−109016150 (48.2)
PZE−109016955 (17) PZE−109016955 (48.9)
PZE−109017196 (17.3) PZE−109017196 (49.3)
PZE−109017592 (17.7) PZE−109017592 (50)
PZE−109017768 (18.2) PZE−109017768 (50.7)
PZE−109018613 (18.9) PZE−109018613 (53.2)
PZE−109019740 (20.1) PZE−109019740 (54.6)
SYN9817 (20.3) SYN9817 (56.2)
PZE−109021713 (22) PZE−109021713 (60)
PZE−109022267 (22.5) PZE−109022267 (60.8)
PZE−109022525 (23) PZE−109022525 (61.5)
PZE−109023719 (23.8) PZE−109023719 (65)
PZB01110.1 (24.1) PZB01110.1 (65.5)
PZE−109023988 (24.1) PZE−109023988 (65.8)
PZB01963.1 (24.5) PZB01963.1 (66)
PZA02648.2 (25.7) PZA02648.2 (67.5)
SYN34139 (26.6) SYN34139 (68.2)
PZB00379.3 (26.7) PZB00379.3 (68.7)
PZE−109026962 (26.9) PZE−109026962 (69.8)
PZE−109027261 (27.2) PZE−109027261 (70.8)
PZE−109027834 (28.6) PZE−109027834 (71)
PUT−163a−101398342−33 (49.2) PUT−163a−101398342−33 (71.6)
PZE−109039951 (60.1) PZE−109040053 (72)
PZE−109040053 (60.4) PZE−109039951 (72.4)
PZE−109044048 (74.6) PZE−109044048 (73)
PZA01791.2 (81) PZA01791.2 (73.6)
PUT−163a−76289682−3927 (87.3) PUT−163a−76289682−3927 (74)
PZE−109050456 (87.8) PZE−109050456 (74.5)
PZE−109051241 (88.7) PZE−109051241 (75.2)
PZE−109052817 (91.3) SYN25681 (76.1)
SYN25681 (92.7) PZE−109052817 (76.6)
PZE−109054300 (94) PZE−109054300 (77.1)
PZE−109054978 (95.6) PZE−109054978 (77.9)
PZE−109055752 (96.4) PZE−109055752 (79)
PZA02897.12 (97.2) PZA02897.12 (79.8)
PZE−109056895 (97.8) PZE−109056895 (80.2)
PZE−109058460 (99.8) PZE−109058617 (82.3)
PZE−109058617 (100.1) PZE−109058460 (82.8)
SYN32233 (100.3) PZE−109059665 (83.3)
PZE−109059665 (101.3) SYN32233 (83.8)
PZE−109060695 (102.4) PZE−109060695 (84.8)
PZE−109063133 (105.4) PZE−109063133 (85.6)
PZE−109064318 (107.5) PZE−109064318 (87.6)
SYN35518 (107.8) SYN35518 (88.1)
PZE−109064888 (108) PZE−109064888 (88.6)
PUT−163a−5739922−2298 (108.7) PUT−163a−5739922−2298 (90.6)
PZE−109065969 (109.1) PZE−109065969 (91.1)
PZE−109066607 (109.6) PZE−109066607 (91.7)
PZE0003695226 (111.1) PZE−100001123 (92.4)
PZE−100001123 (111.3) PZE0003695226 (94.5)
PZE−109067632 (111.5) PZE−109067632 (96.3)
PUT−163a−78075531−4099 (112.1) PUT−163a−78075531−4099 (97.5)
PZA03468.1 (113.7) PZA03468.1 (98)
SYN18132 (114.2) SYN18132 (98.3)
PZB01867.2 (115.5) PZB01867.2 (98.6)
PZE−109071550 (116.3) PZE−109071550 (99.2)
PZE−109073072 (118.1) PZE−109073072 (100.2)
PZE−109073134 (118.3) PZE−109073134 (100.6)
PZE−109073543 (118.9) PZE−109073543 (100.8)
SYN27246 (119.7) SYN27246 (101.3)
PUT−163a−60342651−2460 (122.2) PUT−163a−60342651−2460 (102.2)
PZE−109075943 (122.9) PZE−109075943 (102.5)
PZE−109076558 (123.8) PZE−109076558 (103)
PZE−109077330 (124.8) PZE−109077330 (104.7)
PZE−109077933 (125.6) PZE−109077933 (105.7)
PZE−109078393 (126.3) PZE−109078393 (106.4)
PZE−109078789 (126.9) PZE−109078789 (107.4)
SYN23643 (127.5) SYN23643 (107.8)
PUT−163a−16926216−1141 (128.2) PUT−163a−16926216−1141 (108.4)
PZE−109080559 (128.6) PZE−109080559 (109)
PZE−109080751 (128.7) PZE−109080751 (109.3)
PUT−163a−60357793−2838 (129.2) PUT−163a−60357793−2838 (109.9)
PZE−109081422 (129.7) PZE−109081422 (110.2)
PZE−109082918 (131.6) PZE−109082918 (111)
PZA01866.1 (133.9) PZA01866.1 (113.2)
PZE−109086287 (134.8) PZE−109086287 (115.4)
PZE−109087395 (136) PZE−109087395 (116.9)
SYN22080 (136.9) SYN22080 (117.9)
SYN26337 (137.3) SYN26337 (119.1)
PZE−109089934 (137.8) PZE−109089934 (120.1)
PZE−109091272 (138.7) PZE−109091272 (122)
SYN4010 (138.9) SYN4010 (124)
PZE−109091985 (139) PZE−109091985 (124.4)
PZE−109093491 (140) PZE−109093491 (127.5)
PZE−109094881 (141.3) PZE−109094881 (128.2)
PZE−109095469 (141.5) PZE−109095469 (129)
SYN22829 (141.9) SYN22829 (130.3)
SYN16112 (142) SYN16112 (130.8)
PZE−109097083 (142.7) PZE−109097083 (132.3)
PZE−109097654 (142.9) PZE−109097654 (135.4)
PZE−109098921 (143.9) PZE−109098921 (137.7)
SYN25122 (145.4) SYN25122 (143.8)
PUT−163a−88750800−4538 (146.3) PUT−163a−88750800−4538 (146.2)
SYN30119 (146.5) SYN30119 (146.9)
PZE−109103504 (146.8) PZE−109103504 (148.5)
PZA00323.3 (146.9) PZA00323.3 (149.2)
SYN26903 (147.5) SYN26903 (150.2)
SYN10260 (147.7) SYN10260 (153.3)
PZE−109106296 (148.2) PZE−109106296 (154.2)
PZE−109106589 (148.4) PZE−109106589 (154.9)
PZE−109106846 (148.7) PZE−109106846 (155.2)
PZE−109107497 (149.1) PZE−109107497 (156.3)
SYN26497 (149.3) SYN26497 (156.9)
PZE−109108944 (149.7) PZE−109108944 (160.8)
PZE−109110002 (150.8) PZE−109110002 (162.2)
PZE−109110614 (151.1) PZE−109110614 (164.7)
SYN7109 (151.3) SYN7109 (165.5)
SYN35714 (151.5) SYN35714 (166.4)
PZE−109112955 (151.7) PZE−109112955 (166.8)
PZE−109113590 (151.8) PZE−109113590 (168.6)
SYN10591 (152) SYN10591 (169.9)
PUT−163a−50330000−2209 (152.6) PUT−163a−50330000−2209 (173.8)
SYN6090 (153.2) SYN6090 (175.3)
PZE−109116876 (153.4) PZE−109116876 (176.7)
SYN24330 (153.5) SYN24330 (179.6)
PZE−109117925 (153.6) PZE−109117925 (181.4)
SYN13929 (153.8) SYN13929 (184.3)
PZE−109119001 (153.9) PZE−109119001 (184.6)
SYN13950 (154) SYN13950 (185.4)
PZE−109119987 (154.3) PZE−109119987 (186.8)
SYN6660 (154.5) SYN6660 (187.6)
PUT−163a−78122776−4410 (154.5) PUT−163a−78122776−4410 (187.9)
SYN23701 (154.6) SYN23701 (188.6)
SYN5443 (154.9) SYN5443 (188.9)
PZE−109121439 (155.5) PZE−109121439 (189.4)
PZE−109122122 (156.4) PZE−109122122 (190.4)

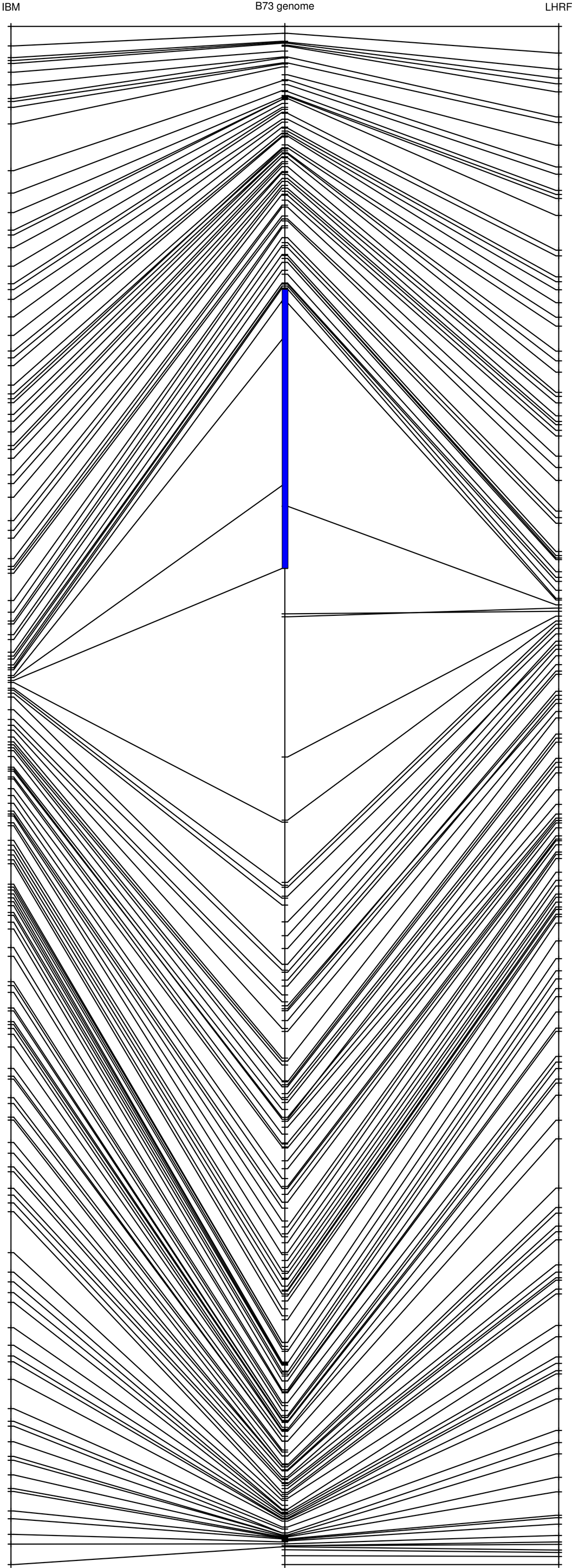

Chrom 10

IBM map (cM)	B73 genome (Mbp)
SYN17514 (0)	SYN17514 (1.1)
SYN16185 (1.8)	SYN16185 (2)
SYN16805 (3.8)	SYN16805 (2.3)
SYN17621 (4.3)	SYN17621 (2.4)
SYN12396 (5.7)	SYN12396 (2.7)
SYN12401 (8.3)	SYN12401 (2.8)
SYN16617 (8.6)	SYN16617 (3.2)
SYN17105 (10.7)	SYN17105 (4)
SYN14833 (12.3)	SYN14833 (4.5)
SYN4482 (13.1)	SYN4482 (4.6)
SYN4471 (14.6)	SYN4471 (4.7)
SYN4484 (15.4)	SYN4484 (4.7)
SYN17783 (18.2)	SYN17783 (4.9)
SYN16720 (21.7)	SYN16720 (5.9)
PZE−110009032 (27.9)	PZE−110009032 (7.1)
PZE−110009334 (29.8)	PZE−110009334 (7.2)
PZE−110009907 (30.1)	PZE−110009907 (8.4)
SYN17272 (30.4)	SYN17272 (8.9)
PZE−110010846 (30.6)	PZE−110010846 (9.5)
SYN23776 (32)	SYN23776 (9.9)
SYN17564 (32.6)	SYN17564 (10.6)
PZE−110012285 (33.5)	PZE−110012285 (10.7)
PZE−110013051 (34.7)	PZE−110013051 (11.7)
PZE−110014547 (35.4)	PZE−110014547 (13.8)
PZE−110014718 (36.1)	PZE−110014718 (14.1)
PZE−110015042 (36.6)	PZE−110015042 (14.6)
PZE−110015184 (36.8)	PZE−110015184 (14.8)
PZE−110016084 (37.1)	PZE−110016084 (16.4)
PZE−110016291 (38)	PZE−110016291 (16.6)
PZE−110019712 (39.2)	PZE−110019712 (24.7)
PZE−110020197 (40.3)	PZE−110020197 (25.6)
PZA03342.2 (41.1)	PZA03342.2 (59.9)
PZE−110033293 (41.7)	PZE−110033293 (62.7)
PZE−110036826 (42.3)	PZE−110036826 (70.3)
PZE−110039284 (42.8)	PZE−110039284 (75.7)
PZE−110039831 (43.1)	PZE−110039831 (76.9)
PZE−110040193 (43.5)	PZE−110040193 (77.3)
PZE−110042215 (44.3)	PZE−110042215 (80.6)
PZE−110042705 (44.5)	PZE−110042705 (81.2)
SYN11547 (44.8)	SYN11547 (83.5)
PZE−110044190 (46.2)	PZE−110044190 (84.1)
PZE−110044891 (46.8)	PZE−110044891 (85.3)
PZE−110048720 (47.4)	PZE−110048720 (91.4)
SYN23779 (47.8)	SYN23779 (95.2)
PZE−110052325 (48)	PZE−110052325 (98.9)
SYN15824 (49)	SYN15824 (99.8)
PUT−163a−89249339−4631 (49.8)	PUT−163a−89249339−4631 (108.4)
SYN1116 (50.6)	SYN1116 (113.2)
SYN16793 (51)	SYN16793 (113.7)
PZE−110060779 (51.2)	PZE−110060779 (115.1)
PZE−110061725 (51.4)	PZE−110061725 (116.9)
PZE−110062653 (51.8)	PZE−110062653 (117.7)
PZE−110064395 (52.3)	PZE−110064395 (120)
SYN30375 (53)	SYN30375 (121.5)
SYN23939 (53.3)	SYN23939 (123.6)
SYN22810 (53.8)	SYN22810 (124.2)
PZE−110069835 (54.2)	PZE−110069835 (126.9)
SYN18871 (55.4)	SYN18871 (127.3)
PZE−110070914 (55.9)	PZE−110070914 (127.4)
SYN17536 (56.2)	SYN17536 (129.5)
PZE−110073500 (56.4)	PZE−110073500 (130.2)
PZE−110073743 (57)	PZE−110073743 (130.3)
PZE−110074757 (58.2)	PZE−110074757 (131)
SYN4216 (59)	SYN4216 (132.1)
PZE−110076741 (60.1)	PZE−110076741 (132.5)
SYN36342 (60.5)	SYN36342 (132.9)
PZE−110077785 (60.8)	PZE−110077785 (133.1)
PZE−110078631 (61.2)	PZE−110078631 (133.4)
SYN4826 (63.7)	SYN4826 (134.7)
PZE−110082048 (66.3)	PZE−110082048 (135.3)
PZE−110082557 (66.6)	PZE−110082557 (135.7)
SYN18730 (67.6)	SYN18730 (135.9)
PZA−001456002 (68.3)	PZA−001456002 (136.3)
PZE−110084754 (68.9)	PZE−110084754 (136.7)
SYN28385 (70.7)	SYN28385 (137.2)
PZE−110085763 (71.1)	PZE−110085763 (137.3)
SYN30303 (71.6)	SYN30303 (137.5)
PZE−110086343 (71.8)	PZE−110086343 (137.5)
SYN18456 (72.2)	SYN18456 (138)
PUT−163a−88747784−4527 (72.6)	PUT−163a−88747784−4527 (138.2)
SYN21905 (73.4)	SYN21905 (138.2)
PZE−110088311 (73.8)	PZE−110088311 (138.7)
PZE−110089201 (77.2)	PZE−110089201 (139.1)
PZE−110090887 (78)	PZE−110090887 (140)
SYN13120 (78.9)	SYN13120 (141)
PZE−110092089 (79.3)	PZE−110092089 (141)
PZE−110093416 (80.8)	PZE−110093416 (141.4)
SYN38150 (81.1)	SYN38150 (141.4)
SYN36714 (82.5)	SYN36714 (141.6)
SYN16965 (83.7)	SYN16965 (141.8)
SYN24308 (84.3)	SYN24308 (142.2)
SYN1018 (86)	SYN1018 (142.7)
PUT−163a−29544531−1779 (87.3)	PUT−163a−29544531−1779 (142.8)
PZE−110097359 (90.5)	PZE−110097359 (143.2)
SYN6070 (92.1)	SYN6070 (143.5)
SYN4350 (93.4)	SYN4350 (144.1)
PZE−110099681 (94.7)	PZE−110099681 (144.7)
PZE−110100685 (96.6)	PZE−110100685 (145)
PZE−110101412 (98.4)	PZE−110101412 (145.2)
SYN11610 (100.4)	SYN11610 (145.6)
PZE−110102934 (101.1)	PZE−110102934 (146)
SYN15271 (101.4)	SYN15271 (146.1)
SYN12666 (105.3)	SYN12666 (146.2)
SYN19616 (106.8)	SYN19616 (146.6)
SYN35585 (108.5)	SYN35585 (147)
PZE−110105598 (110.3)	PZE−110105598 (147.1)
SYN22560 (111.5)	SYN22560 (147.2)
PZE−110106108 (111.9)	PZE−110106108 (147.3)
SYN19213 (112.3)	SYN19213 (147.6)
SYN19209 (113.6)	SYN19209 (147.6)
SYN19279 (114.5)	SYN19279 (147.8)
SYN19290 (115.9)	SYN19290 (147.9)
SYN19286 (116.8)	SYN19286 (147.9)
PZE−110109364 (118.9)	PZE−110109364 (148.5)
SYN10867 (119.7)	SYN10867 (148.6)
SYN13564 (121.6)	SYN13564 (148.9)
PUT−163a−149071204−827 (122.5)	PUT−163a−149071204−827 (149)

B73 genome (Mbp)	LHRF map (cM)
SYN17109 (4)	SYN17109 (0)
SYN4482 (4.6)	SYN4482 (3.8)
SYN17783 (4.9)	SYN17783 (9.5)
PZA02095.10 (5.6)	PZA02095.10 (12.3)
PZE−110008028 (6.1)	PZE−110008028 (14.2)
PZE−110008833 (6.5)	PZE−110008833 (16.4)
PZE−110009032 (7.1)	PZE−110009032 (18.3)
PZE−110009225 (7.1)	PZE−110009225 (19.7)
PZE−110009334 (7.2)	PZE−110009334 (20.2)
PZE−110010396 (8.9)	PZE−110010396 (22)
PZB01301.6 (10.2)	PZB01301.6 (24.3)
PUT−163a−71767261−3524 (10.4)	PUT−163a−71767261−3524 (24.8)
PHM15331.16 (10.9)	PHM15331.16 (25.6)
SYN18994 (12.7)	SYN18994 (27.4)
PUT−163a−71758789−3424 (13.5)	PUT−163a−71758789−3424 (28.1)
PZE−110014694 (14)	PZE−110014694 (28.8)
PZE−110014871 (14.4)	PZE−110014871 (29.4)
PZE−110015359 (14.9)	PZE−110015359 (30.9)
PHM3922.32 (17.7)	PHM3922.32 (31.8)
PUT−163a−78086732−4196 (20.4)	PUT−163a−78086732−4196 (32.3)
PZE−110018270 (21.7)	PZE−110018270 (32.8)
SYN26652 (22.3)	SYN26652 (33)
SYN26634 (22.4)	SYN26634 (33.4)
PUT−163a−110518051−97 (23.2)	PUT−163a−110518051−97 (33.8)
PZE−110020065 (25.3)	PZE−110020065 (35)
PUT−163a−110516422−93 (29.9)	PUT−163a−110516422−93 (35.4)
PZE−110025113 (55.8)	PZE−110025113 (35.8)
PZE−110025413 (57.3)	PZE−110025413 (36.2)
PUT−163a−13177814−171 (70.3)	PUT−163a−13177814−171 (36.6)
PZE−110038481 (73.6)	PZE−110038481 (37)
SYN22037 (76.6)	SYN22037 (38.1)
PZE−110040063 (77.2)	PZE−110040063 (39)
PZE−110040221 (77.3)	PZE−110040221 (41.4)
PZE−110040541 (77.4)	PZE−110040541 (42.8)
PZE−110041758 (79.9)	PZE−110041758 (43.7)
PZE−110042016 (80.1)	PZE−110042016 (44)
PZE−110043055 (82.1)	PZE−110043055 (44.2)
PZE−110044004 (83.7)	PZE−110044004 (44.7)
SYN8173 (85.2)	SYN8173 (44.9)
PZE−110044817 (85.3)	PZE−110044817 (45.2)
PZE−110046358 (87.2)	PZE−110046358 (47.2)
PZE−110047212 (88.4)	PZE−110047212 (48.6)
PZE−110049100 (92)	SYN4166 (49.2)
SYN4166 (92)	PZE−110049100 (49.5)
PZE−110049940 (94.1)	PZE−110049940 (50.1)
PZE−110050295 (95)	PZE−110050295 (50.7)
PZE−110050659 (95.4)	PZE−110050659 (51.1)
PZE−110052085 (98.4)	PZE−110052085 (51.5)
PZE−110052763 (99.6)	PZE−110052763 (51.7)
PZA03585.1 (105)	PZA03585.1 (52.3)
PUT−163a−89249339−4630 (108.4)	PUT−163a−89249339−4630 (52.7)
PZA01292.1 (109.8)	PZA01292.1 (53.3)
SYN19553 (109.9)	SYN19553 (53.7)
PZE−110058323 (112.2)	PZE−110058323 (54.3)
PZE−110058964 (113.2)	PZE−110058964 (57.1)
PZB02237.1 (114.3)	PZB02237.1 (57.8)
PZE−110060456 (114.8)	PZE−110060456 (58.1)
PZE−110061523 (116.5)	PZE−110061523 (58.4)
PZE−110061629 (116.8)	PZE−110061629 (58.8)
PZE−110062213 (117.5)	PZE−110062213 (59.7)
PHM13687.14 (118)	PHM13687.14 (60.6)
PZE−110065112 (120.8)	PZE−110065112 (61.1)
SYN27054 (122.3)	SYN27054 (62.2)
PUT−163a−148967626−626 (123.2)	PUT−163a−148967626−626 (63.3)
PUT−163a−16921481−1061 (124.1)	PUT−163a−16921481−1061 (64)
PZE−110068820 (125.7)	PZE−110068820 (64.5)
PZE−110070282 (127.3)	PZE−110070282 (66.2)
PZE−110070863 (127.4)	PZE−110070863 (66.8)
PZE−110072497 (129)	PZE−110072497 (68)
PZE−110073579 (130.3)	PZE−110073579 (69.1)
PZE−110073807 (130.3)	PZE−110073807 (69.7)
PZE−110073934 (130.4)	PZE−110073934 (70.2)
PZE−110075161 (131.9)	PZE−110075161 (72.1)
SYN22958 (132.5)	SYN22958 (73.3)
PZE−110077785 (133.1)	PZE−110077785 (74.4)
PZE−110078631 (133.4)	PZE−110078631 (75.9)
PZE−110079903 (134.2)	PZE−110079903 (76.9)
SYN18728 (135.9)	SYN18728 (78.9)
PZE−110084185 (136.4)	PZE−110084185 (79.7)
PZE−110084754 (136.7)	PZE−110084754 (80.8)
PZE−110085296 (137.1)	PZE−110085296 (81.3)
PZE−110085688 (137.3)	PZE−110085688 (81.7)
PZE−110086687 (137.7)	PZE−110086687 (82.8)
SYN18455 (138)	SYN18455 (83.4)
SYN37370 (140)	SYN37370 (91.3)
PZE−110091181 (140.3)	PZE−110091181 (93.1)
SYN13120 (141)	SYN13120 (94.4)
PZE−110091989 (141)	PZE−110091989 (94.9)
SYN9682 (142.1)	SYN9682 (98.3)
PZE−110095199 (142.2)	PZE−110095199 (99.3)
SYN650 (143.4)	SYN650 (104.8)
SYN15700 (144.4)	SYN15700 (106.3)
PZE−110099681 (144.7)	PZE−110099681 (107.1)
PZE−110100655 (145)	PZE−110100655 (109.3)
SYNGENTA1862 (145.3)	SYNGENTA1862 (111.5)
SYN8538 (145.5)	SYN8538 (113.3)
PZE−110102934 (146)	PZE−110102934 (114)
PZE−110103696 (146.3)	PZE−110103696 (117.5)
PUT−163a−101396692−29 (147)	PUT−163a−101396692−29 (120.6)
SYN22567 (147.2)	SYN22567 (122.1)
PZE−110106461 (147.4)	PZE−110106461 (123.9)
SYN19279 (147.8)	SYN19279 (125.5)
SYN19774 (148.1)	SYN19774 (128.6)
PZE−110109218 (148.4)	PZE−110109218 (129.6)
PZE−110109364 (148.5)	PZE−110109364 (131.2)
PZE−110110463 (148.9)	PZE−110110463 (136.5)

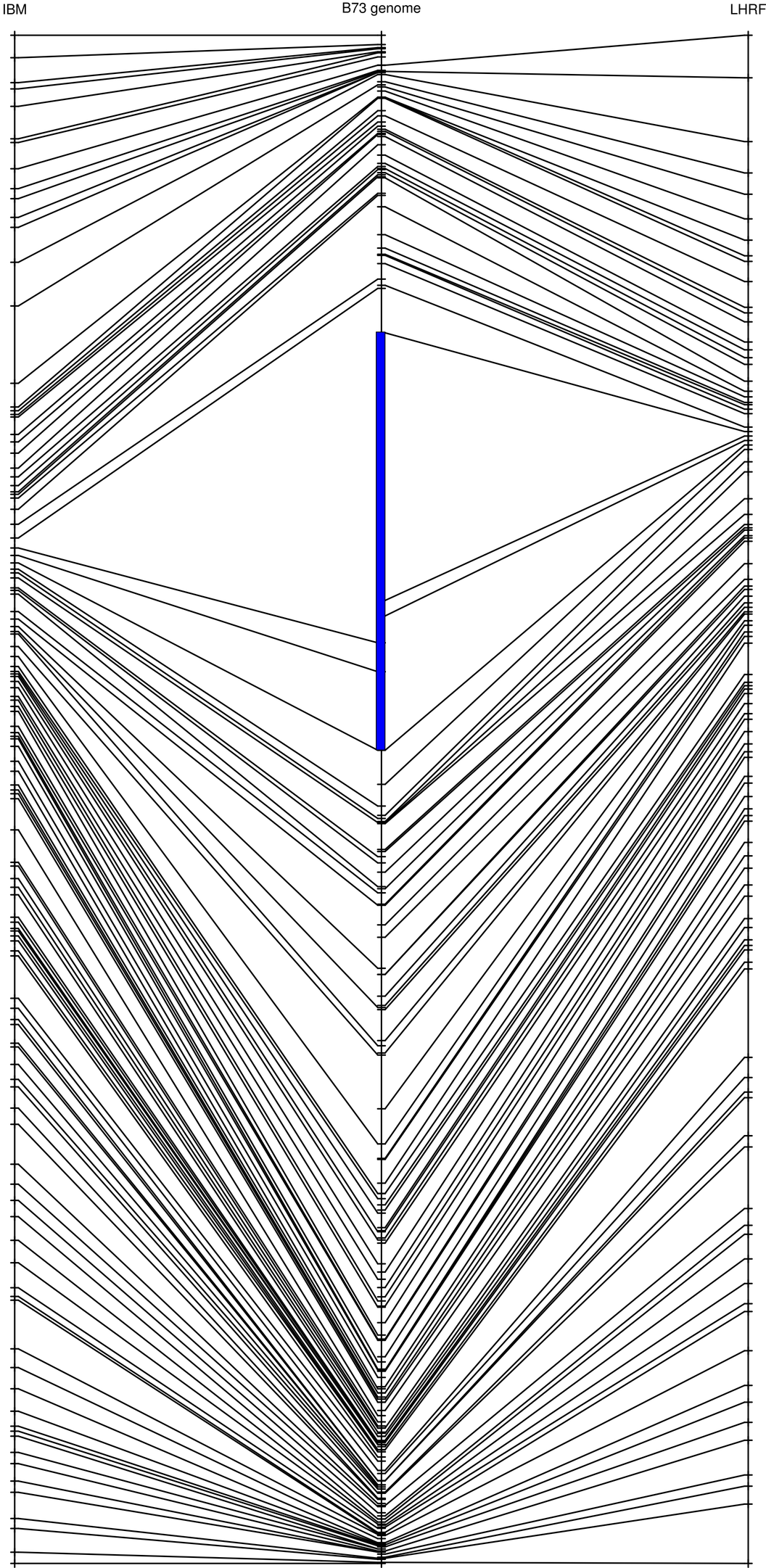

Supplement: Figure S4 — Whole-chromosome comparison between the framework genetic maps IBM and LHRF and the B73 genome coordinates for entire chromosomes. In the ladder diagrams of the two left panels, the position of a marker corresponds to its index in the ordered map and not to its genetic position. Numbers in parentheses indicate the map coordinate in cM for IBM or LHRF genetic maps and in Mbp for the B73 genome sequence. In the right panel, positions of the markers are proportional to the cM or Mb map coordinate. The ladders have their scales adjusted to fit the two maps to the same height. In the right panel, genetic maps are scaled to the physical map length. Blue rectangles indicate marker intervals containing the centromere, according to MaizeGDB. (PDF) [file pone.0028334.s004.pdf]
