# Supplementary material for: A Large Maize (Zea mays L.) SNP Genotyping Array: Development and Germplasm Genotyping, and Genetic Mapping to Compare with the B73 Reference Genome
Source: PLoS One. 2011 Dec 8;6(12):e28334. doi: 10.1371/journal.pone.0028334 (PMC3234264; doi:10.1371/journal.pone.0028334)
Supplement: Figure S6 — Comparison between the complete genetic maps IBM and LHRF, and the B73 genome for nine regions for which either genetic map contains markers non-colinear with the B73 genome. The complete genetic maps containing both framework and placed markers are displayed for the nine non-colinear regions defined in Figure 6 and Table S11. In the ladder diagrams of the upper panels, positions of the markers correspond to their index in the ordered maps and not to their genetic position. Numbers in parentheses indicate the map coordinate in cM for IBM or LHRF genetic maps and in Mbp for the B73 genome sequence. In the lower panel, positions are proportional to the physical map coordinates. Scales were adjusted to fit the two maps to the same height. (PDF) [file pone.0028334.s006.pdf]

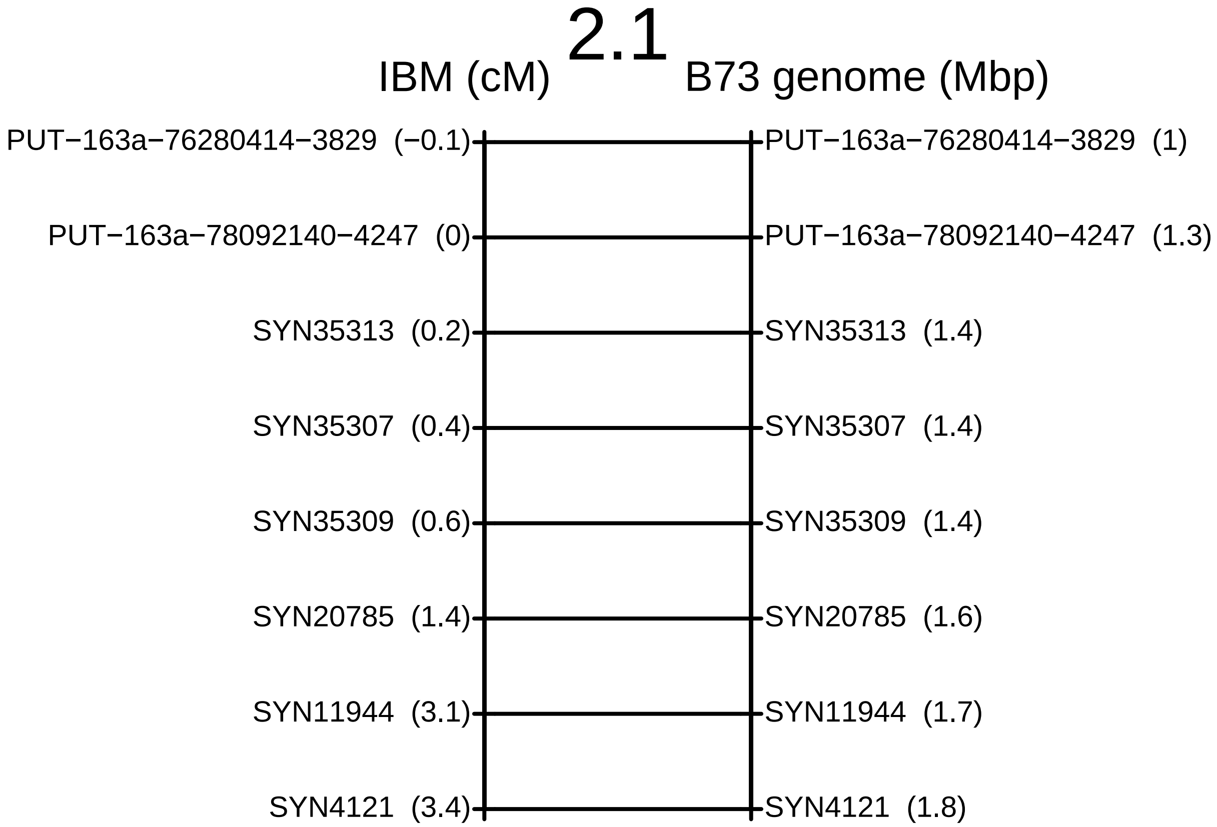
2.1
IBM (cM)
B73 genome (Mbp)
PUT−163a−76280414−3829 (−0.1)
PUT−163a−78092140−4247 (0)
SYN35313 (0.2)
SYN35307 (0.4)
SYN35309 (0.6)
SYN20785 (1.4)
SYN11944 (3.1)
SYN4121 (3.4)
PUT−163a−76280414−3829 (1)
PUT−163a−78092140−4247 (1.3)
SYN35313 (1.4)
SYN35307 (1.4)
SYN35309 (1.4)
SYN20785 (1.6)
SYN11944 (1.7)
SYN4121 (1.8)

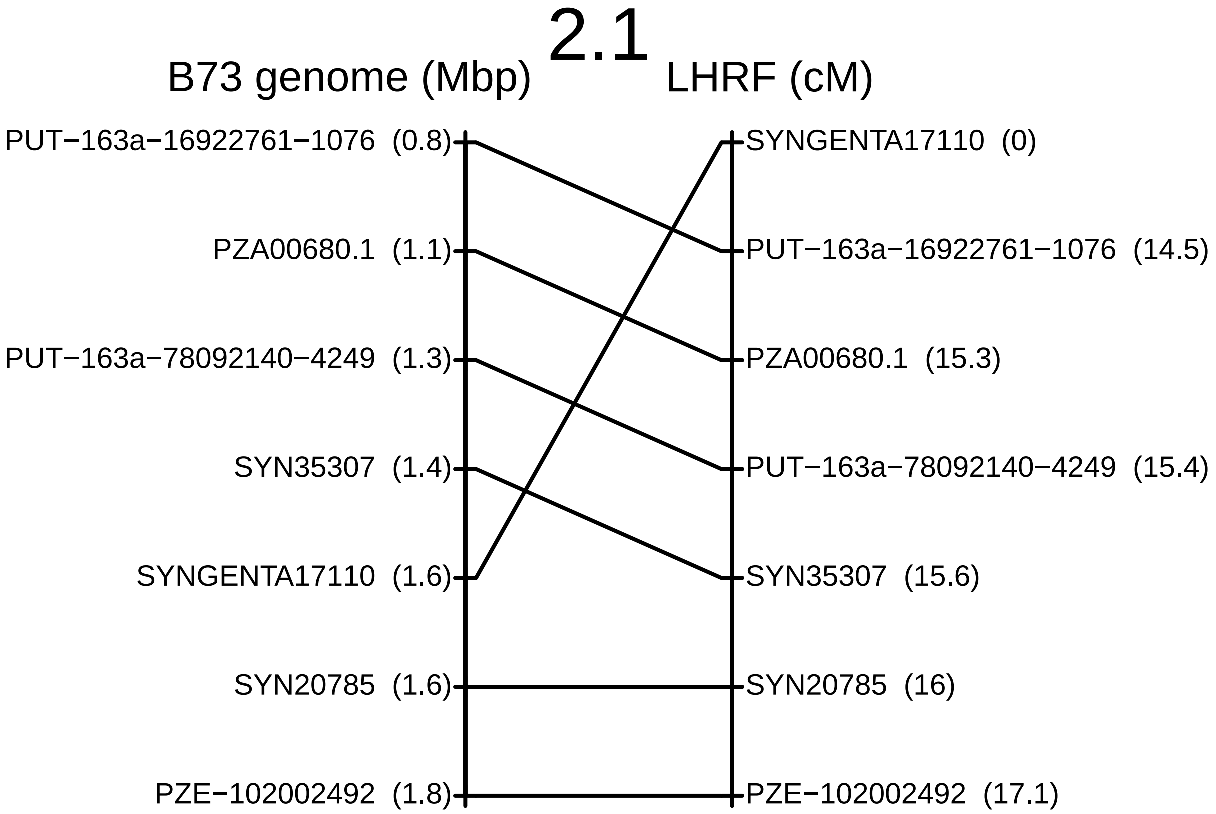
2.1
B73 genome (Mbp)
LHRF (cM)
PUT−163a−16922761−1076 (0.8)
PZA00680.1 (1.1)
PUT−163a−78092140−4249 (1.3)
SYN35307 (1.4)
SYNGENTA17110 (1.6)
SYN20785 (1.6)
PZE−102002492 (1.8)
SYNGENTA17110 (0)
PUT−163a−16922761−1076 (14.5)
PZA00680.1 (15.3)
PUT−163a−78092140−4249 (15.4)
SYN35307 (15.6)
SYN20785 (16)
PZE−102002492 (17.1)

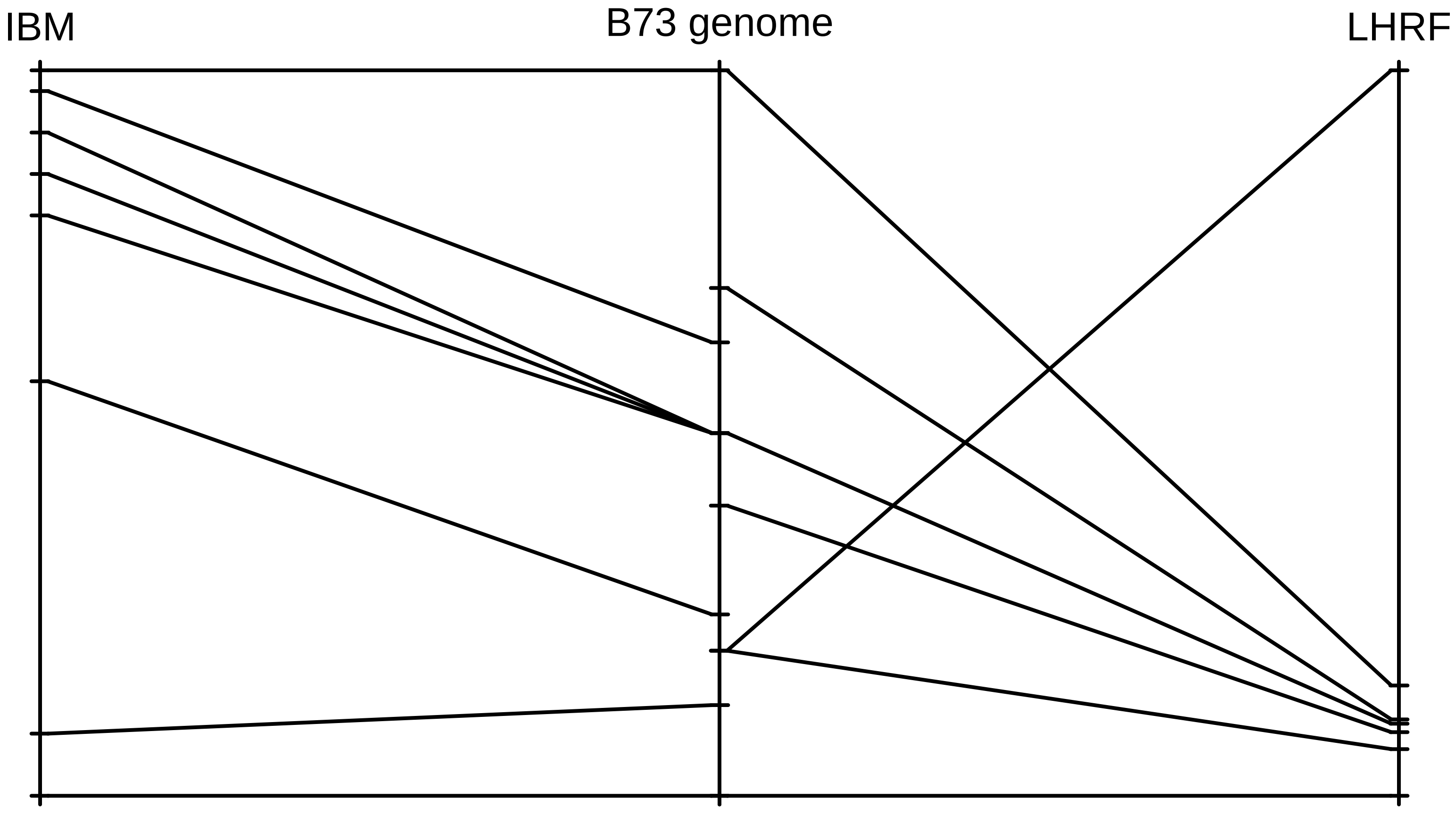
IBM
B73 genome
LHRF

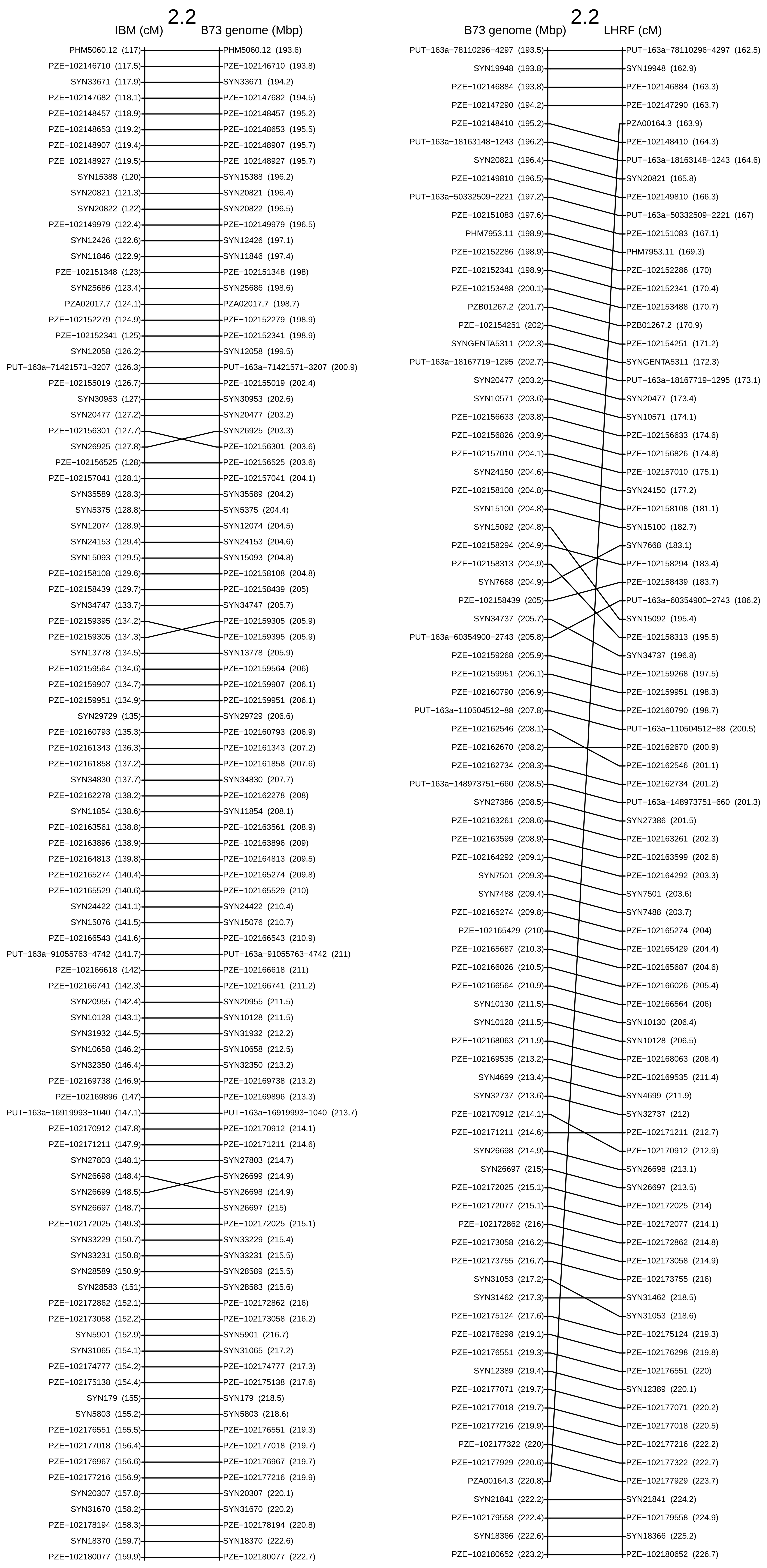
2.2
IBM (cM)
B73 genome (Mbp)
2.2
B73 genome (Mbp)
LHRF (cM)

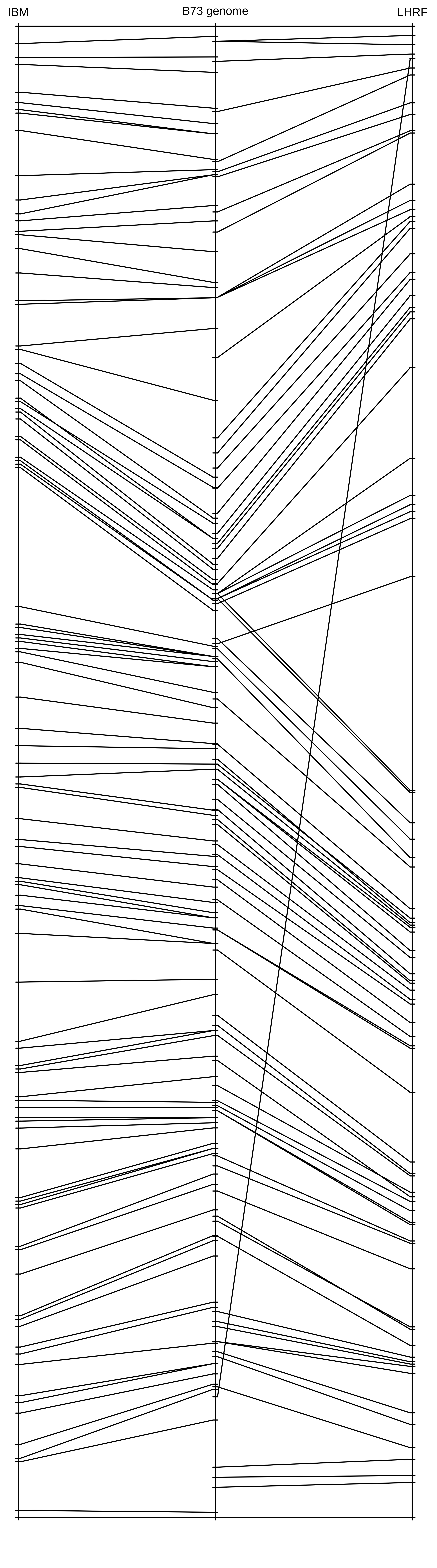
IBM
B73 genome
LHRF

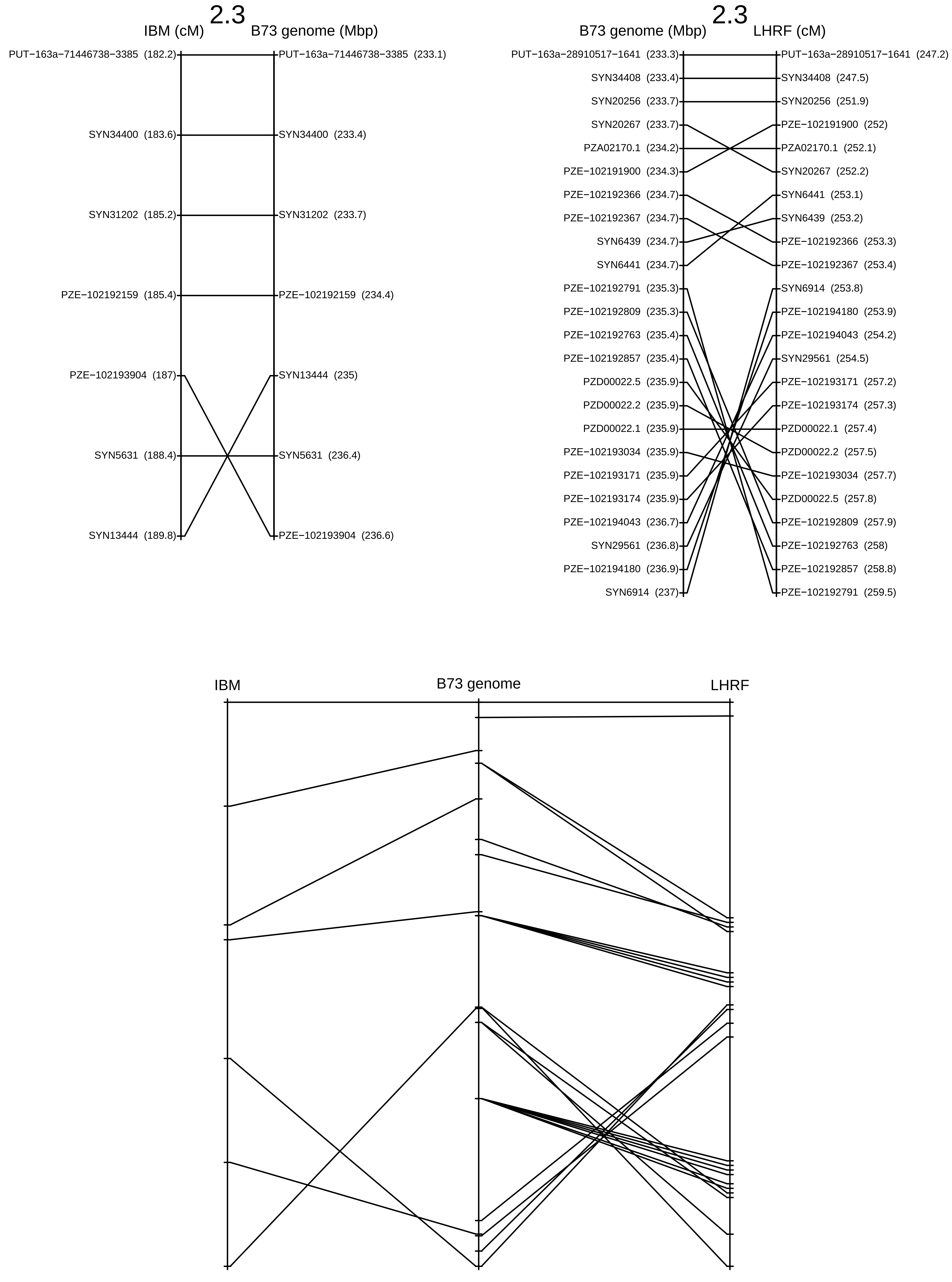
2.3
IBM (cM)
B73 genome (Mbp)
PUT−163a−71446738−3385 (182.2)
SYN34400 (183.6)
SYN31202 (185.2)
PZE−102192159 (185.4)
PZE−102193904 (187)
SYN5631 (188.4)
SYN13444 (189.8)
PUT−163a−71446738−3385 (233.1)
SYN34400 (233.4)
SYN31202 (233.7)
PZE−102192159 (234.4)
SYN13444 (235)
SYN5631 (236.4)
PZE−102193904 (236.6)
2.3
B73 genome (Mbp)
LHRF (cM)
PUT−163a−28910517−1641 (233.3)
SYN34408 (233.4)
SYN20256 (233.7)
SYN20267 (233.7)
PZA02170.1 (234.2)
PZE−102191900 (234.3)
PZE−102192366 (234.7)
PZE−102192367 (234.7)
SYN6439 (234.7)
SYN6441 (234.7)
PZE−102192791 (235.3)
PZE−102192809 (235.3)
PZE−102192763 (235.4)
PZE−102192857 (235.4)
PZD00022.5 (235.9)
PZD00022.2 (235.9)
PZD00022.1 (235.9)
PZE−102193034 (235.9)
PZE−102193171 (235.9)
PZE−102193174 (235.9)
PZE−102194043 (236.7)
SYN29561 (236.8)
PZE−102194180 (236.9)
SYN6914 (237)
PUT−163a−28910517−1641 (247.2)
SYN34408 (247.5)
SYN20256 (251.9)
PZE−102191900 (252)
PZA02170.1 (252.1)
SYN20267 (252.2)
SYN6441 (253.1)
SYN6439 (253.2)
PZE−102192366 (253.3)
PZE−102192367 (253.4)
SYN6914 (253.8)
PZE−102194180 (253.9)
PZE−102194043 (254.2)
SYN29561 (254.5)
PZE−102193171 (257.2)
PZE−102193174 (257.3)
PZD00022.1 (257.4)
PZD00022.2 (257.5)
PZE−102193034 (257.7)
PZD00022.5 (257.8)
PZE−102192809 (257.9)
PZE−102192763 (258)
PZE−102192857 (258.8)
PZE−102192791 (259.5)
IBM
B73 genome
LHRF

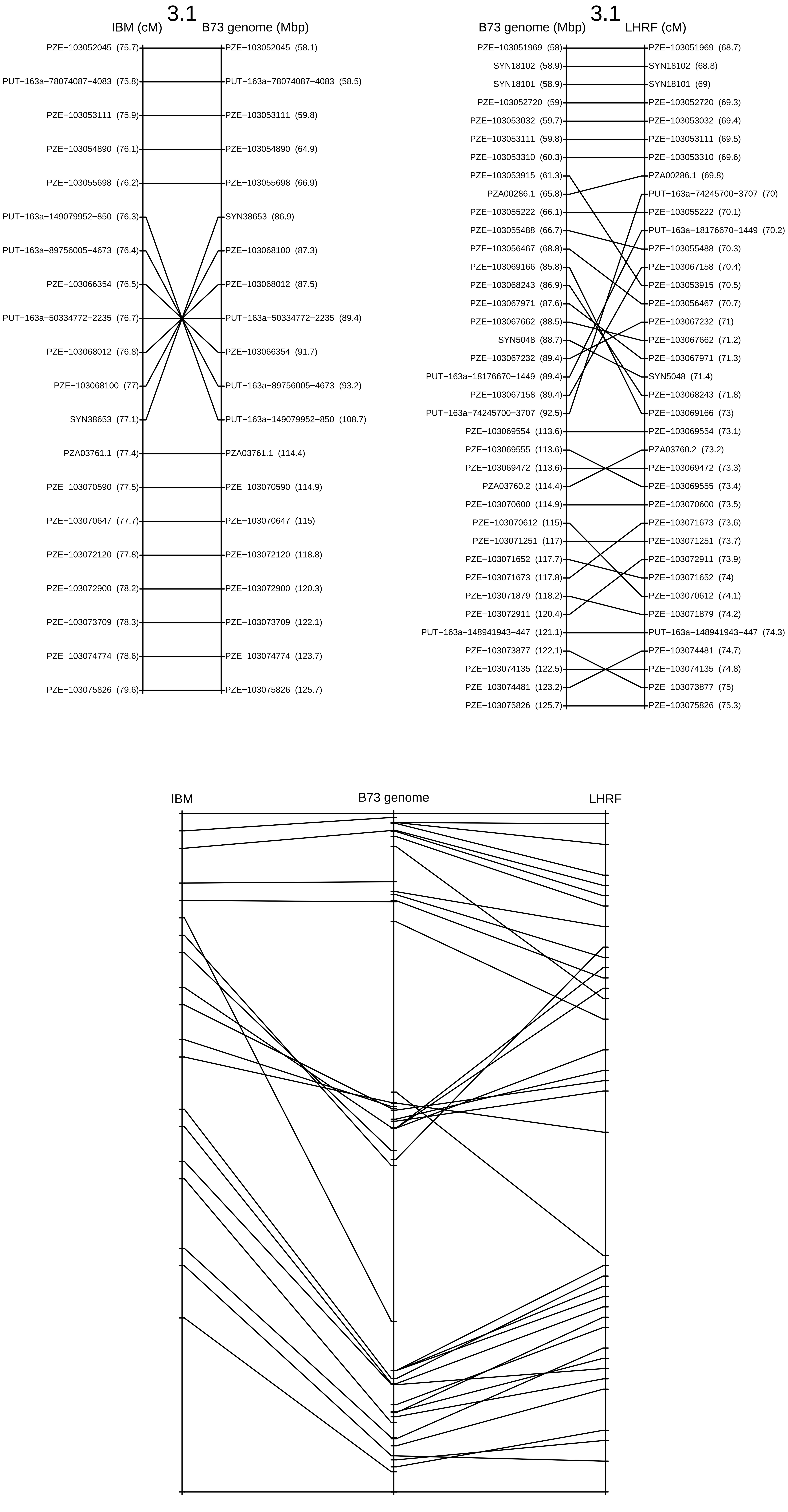

3.1
IBM (cM)
B73 genome (Mbp)
PZE−103052045 (75.7)
PUT−163a−78074087−4083 (75.8)
PZE−103053111 (75.9)
PZE−103054890 (76.1)
PZE−103055698 (76.2)
PUT−163a−149079952−850 (76.3)
PUT−163a−89756005−4673 (76.4)
PZE−103066354 (76.5)
PUT−163a−50334772−2235 (76.7)
PZE−103068012 (76.8)
PZE−103068100 (77)
SYN38653 (77.1)
PZA03761.1 (77.4)
PZE−103070590 (77.5)
PZE−103070647 (77.7)
PZE−103072120 (77.8)
PZE−103072900 (78.2)
PZE−103073709 (78.3)
PZE−103074774 (78.6)
PZE−103075826 (79.6)
PZE−103052045 (58.1)
PUT−163a−78074087−4083 (58.5)
PZE−103053111 (59.8)
PZE−103054890 (64.9)
PZE−103055698 (66.9)
SYN38653 (86.9)
PZE−103068100 (87.3)
PZE−103068012 (87.5)
PUT−163a−50334772−2235 (89.4)
PZE−103066354 (91.7)
PUT−163a−89756005−4673 (93.2)
PUT−163a−149079952−850 (108.7)
PZA03761.1 (114.4)
PZE−103070590 (114.9)
PZE−103070647 (115)
PZE−103072120 (118.8)
PZE−103072900 (120.3)
PZE−103073709 (122.1)
PZE−103074774 (123.7)
PZE−103075826 (125.7)
3.1
B73 genome (Mbp)
LHRF (cM)
PZE−103051969 (58)
SYN18102 (58.9)
SYN18101 (58.9)
PZE−103052720 (59)
PZE−103053032 (59.7)
PZE−103053111 (59.8)
PZE−103053310 (60.3)
PZE−103053915 (61.3)
PZA00286.1 (65.8)
PZE−103055222 (66.1)
PZE−103055488 (66.7)
PZE−103056467 (68.8)
PZE−103069166 (85.8)
PZE−103068243 (86.9)
PZE−103067971 (87.6)
PZE−103067662 (88.5)
SYN5048 (88.7)
PZE−103067232 (89.4)
PUT−163a−18176670−1449 (89.4)
PZE−103067158 (89.4)
PUT−163a−74245700−3707 (92.5)
PZE−103069554 (113.6)
PZE−103069555 (113.6)
PZE−103069472 (113.6)
PZA03760.2 (114.4)
PZE−103070600 (114.9)
PZE−103070612 (115)
PZE−103071251 (117)
PZE−103071652 (117.7)
PZE−103071673 (117.8)
PZE−103071879 (118.2)
PZE−103072911 (120.4)
PUT−163a−148941943−447 (121.1)
PZE−103073877 (122.1)
PZE−103074135 (122.5)
PZE−103074481 (123.2)
PZE−103075826 (125.7)
PZE−103051969 (68.7)
SYN18102 (68.8)
SYN18101 (69)
PZE−103052720 (69.3)
PZE−103053032 (69.4)
PZE−103053111 (69.5)
PZE−103053310 (69.6)
PZA00286.1 (69.8)
PUT−163a−74245700−3707 (70)
PZE−103055222 (70.1)
PUT−163a−18176670−1449 (70.2)
PZE−103055488 (70.3)
PZE−103067158 (70.4)
PZE−103053915 (70.5)
PZE−103056467 (70.7)
PZE−103067232 (71)
PZE−103067662 (71.2)
PZE−103067971 (71.3)
SYN5048 (71.4)
PZE−103068243 (71.8)
PZE−103069166 (73)
PZE−103069554 (73.1)
PZA03760.2 (73.2)
PZE−103069472 (73.3)
PZE−103069555 (73.4)
PZE−103070600 (73.5)
PZE−103071673 (73.6)
PZE−103071251 (73.7)
PZE−103072911 (73.9)
PZE−103071652 (74)
PZE−103070612 (74.1)
PZE−103071879 (74.2)
PUT−163a−148941943−447 (74.3)
PZE−103074481 (74.7)
PZE−103074135 (74.8)
PZE−103073877 (75)
PZE−103075826 (75.3)
IBM
B73 genome
LHRF

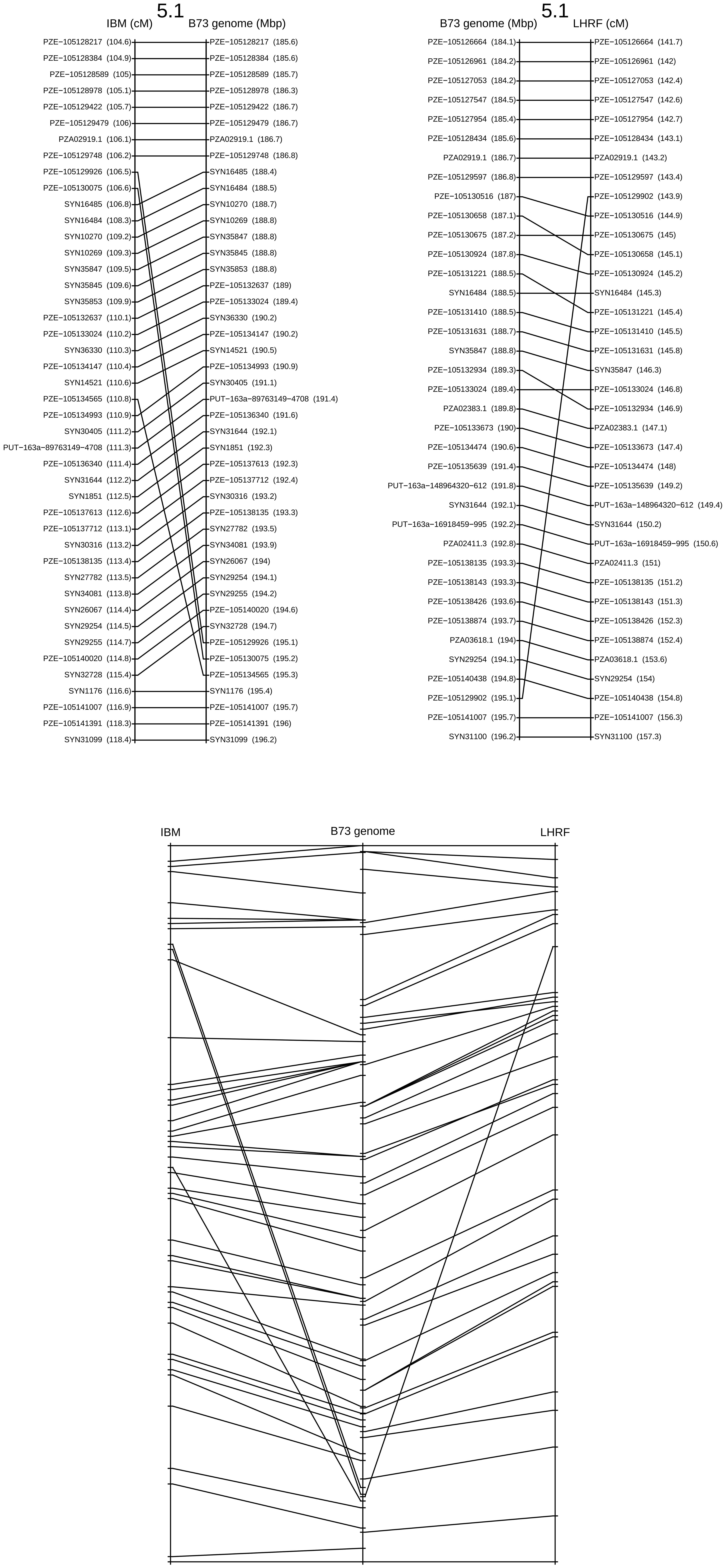
5.1
IBM (cM)
B73 genome (Mbp)
PZE−105128217 (104.6)
PZE−105128384 (104.9)
PZE−105128589 (105)
PZE−105128978 (105.1)
PZE−105129422 (105.7)
PZE−105129479 (106)
PZA02919.1 (106.1)
PZE−105129748 (106.2)
PZE−105129926 (106.5)
PZE−105130075 (106.6)
SYN16485 (106.8)
SYN16484 (108.3)
SYN10270 (109.2)
SYN10269 (109.3)
SYN35847 (109.5)
SYN35845 (109.6)
SYN35853 (109.9)
PZE−105132637 (110.1)
PZE−105133024 (110.2)
SYN36330 (110.3)
PZE−105134147 (110.4)
SYN14521 (110.6)
PZE−105134565 (110.8)
PZE−105134993 (110.9)
SYN30405 (111.2)
PUT−163a−89763149−4708 (111.3)
PZE−105136340 (111.4)
SYN31644 (112.2)
SYN1851 (112.5)
PZE−105137613 (112.6)
PZE−105137712 (113.1)
SYN30316 (113.2)
PZE−105138135 (113.4)
SYN27782 (113.5)
SYN34081 (113.8)
SYN26067 (114.4)
SYN29254 (114.5)
SYN29255 (114.7)
PZE−105140020 (114.8)
SYN32728 (115.4)
SYN1176 (116.6)
PZE−105141007 (116.9)
PZE−105141391 (118.3)
SYN31099 (118.4)
PZE−105128217 (185.6)
PZE−105128384 (185.6)
PZE−105128589 (185.7)
PZE−105128978 (186.3)
PZE−105129422 (186.7)
PZE−105129479 (186.7)
PZA02919.1 (186.7)
PZE−105129748 (186.8)
SYN16485 (188.4)
SYN16484 (188.5)
SYN10270 (188.7)
SYN10269 (188.8)
SYN35847 (188.8)
SYN35845 (188.8)
SYN35853 (188.8)
PZE−105132637 (189)
PZE−105133024 (189.4)
SYN36330 (190.2)
PZE−105134147 (190.2)
SYN14521 (190.5)
PZE−105134993 (190.9)
SYN30405 (191.1)
PUT−163a−89763149−4708 (191.4)
PZE−105136340 (191.6)
SYN31644 (192.1)
SYN1851 (192.3)
PZE−105137613 (192.3)
PZE−105137712 (192.4)
SYN30316 (193.2)
PZE−105138135 (193.3)
SYN27782 (193.5)
SYN34081 (193.9)
SYN26067 (194)
SYN29254 (194.1)
SYN29255 (194.2)
PZE−105140020 (194.6)
SYN32728 (194.7)
PZE−105129926 (195.1)
PZE−105130075 (195.2)
PZE−105134565 (195.3)
SYN1176 (195.4)
PZE−105141007 (195.7)
PZE−105141391 (196)
SYN31099 (196.2)
5.1
B73 genome (Mbp)
LHRF (cM)
PZE−105126664 (184.1)
PZE−105126961 (184.2)
PZE−105127053 (184.2)
PZE−105127547 (184.5)
PZE−105127954 (185.4)
PZE−105128434 (185.6)
PZA02919.1 (186.7)
PZE−105129597 (186.8)
PZE−105130516 (187)
PZE−105130658 (187.1)
PZE−105130675 (187.2)
PZE−105130924 (187.8)
PZE−105131221 (188.5)
SYN16484 (188.5)
PZE−105131410 (188.5)
PZE−105131631 (188.7)
SYN35847 (188.8)
PZE−105132934 (189.3)
PZE−105133024 (189.4)
PZA02383.1 (189.8)
PZE−105133673 (190)
PZE−105134474 (190.6)
PZE−105135639 (191.4)
PUT−163a−148964320−612 (191.8)
SYN31644 (192.1)
PUT−163a−16918459−995 (192.2)
PZA02411.3 (192.8)
PZE−105138135 (193.3)
PZE−105138143 (193.3)
PZE−105138426 (193.6)
PZE−105138874 (193.7)
PZA03618.1 (194)
SYN29254 (194.1)
PZE−105140438 (194.8)
PZE−105129902 (195.1)
PZE−105141007 (195.7)
SYN31100 (196.2)
PZE−105126664 (141.7)
PZE−105126961 (142)
PZE−105127053 (142.4)
PZE−105127547 (142.6)
PZE−105127954 (142.7)
PZE−105128434 (143.1)
PZA02919.1 (143.2)
PZE−105129597 (143.4)
PZE−105129902 (143.9)
PZE−105130516 (144.9)
PZE−105130675 (145)
PZE−105130658 (145.1)
PZE−105130924 (145.2)
SYN16484 (145.3)
PZE−105131221 (145.4)
PZE−105131410 (145.5)
PZE−105131631 (145.8)
SYN35847 (146.3)
PZE−105133024 (146.8)
PZE−105132934 (146.9)
PZA02383.1 (147.1)
PZE−105133673 (147.4)
PZE−105134474 (148)
PZE−105135639 (149.2)
PUT−163a−148964320−612 (149.4)
SYN31644 (150.2)
PUT−163a−16918459−995 (150.6)
PZA02411.3 (151)
PZE−105138135 (151.2)
PZE−105138143 (151.3)
PZE−105138426 (152.3)
PZE−105138874 (152.4)
PZA03618.1 (153.6)
SYN29254 (154)
PZE−105140438 (154.8)
PZE−105141007 (156.3)
SYN31100 (157.3)
IBM
B73 genome
LHRF

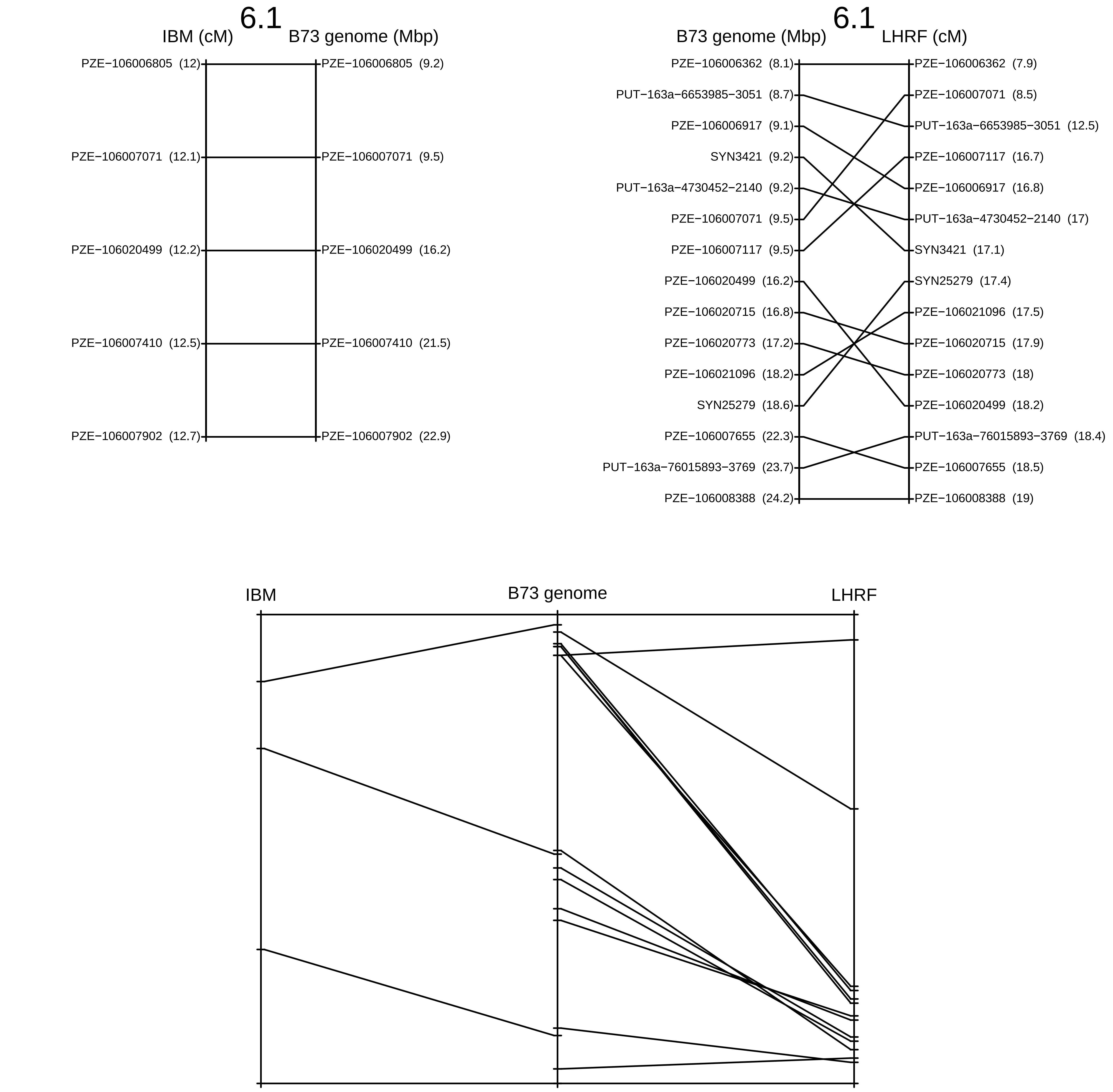
6.1
IBM (cM)
B73 genome (Mbp)
PZE−106006805 (12)
PZE−106007071 (12.1)
PZE−106020499 (12.2)
PZE−106007410 (12.5)
PZE−106007902 (12.7)
PZE−106006805 (9.2)
PZE−106007071 (9.5)
PZE−106020499 (16.2)
PZE−106007410 (21.5)
PZE−106007902 (22.9)
6.1
B73 genome (Mbp)
LHRF (cM)
PZE−106006362 (8.1)
PUT−163a−6653985−3051 (8.7)
PZE−106006917 (9.1)
SYN3421 (9.2)
PUT−163a−4730452−2140 (9.2)
PZE−106007071 (9.5)
PZE−106007117 (9.5)
PZE−106020499 (16.2)
PZE−106020715 (16.8)
PZE−106020773 (17.2)
PZE−106021096 (18.2)
SYN25279 (18.6)
PZE−106007655 (22.3)
PUT−163a−76015893−3769 (23.7)
PZE−106008388 (24.2)
PZE−106006362 (7.9)
PZE−106007071 (8.5)
PUT−163a−6653985−3051 (12.5)
PZE−106007117 (16.7)
PZE−106006917 (16.8)
PUT−163a−4730452−2140 (17)
SYN3421 (17.1)
SYN25279 (17.4)
PZE−106021096 (17.5)
PZE−106020715 (17.9)
PZE−106020773 (18)
PZE−106020499 (18.2)
PUT−163a−76015893−3769 (18.4)
PZE−106007655 (18.5)
PZE−106008388 (19)
IBM
B73 genome
LHRF

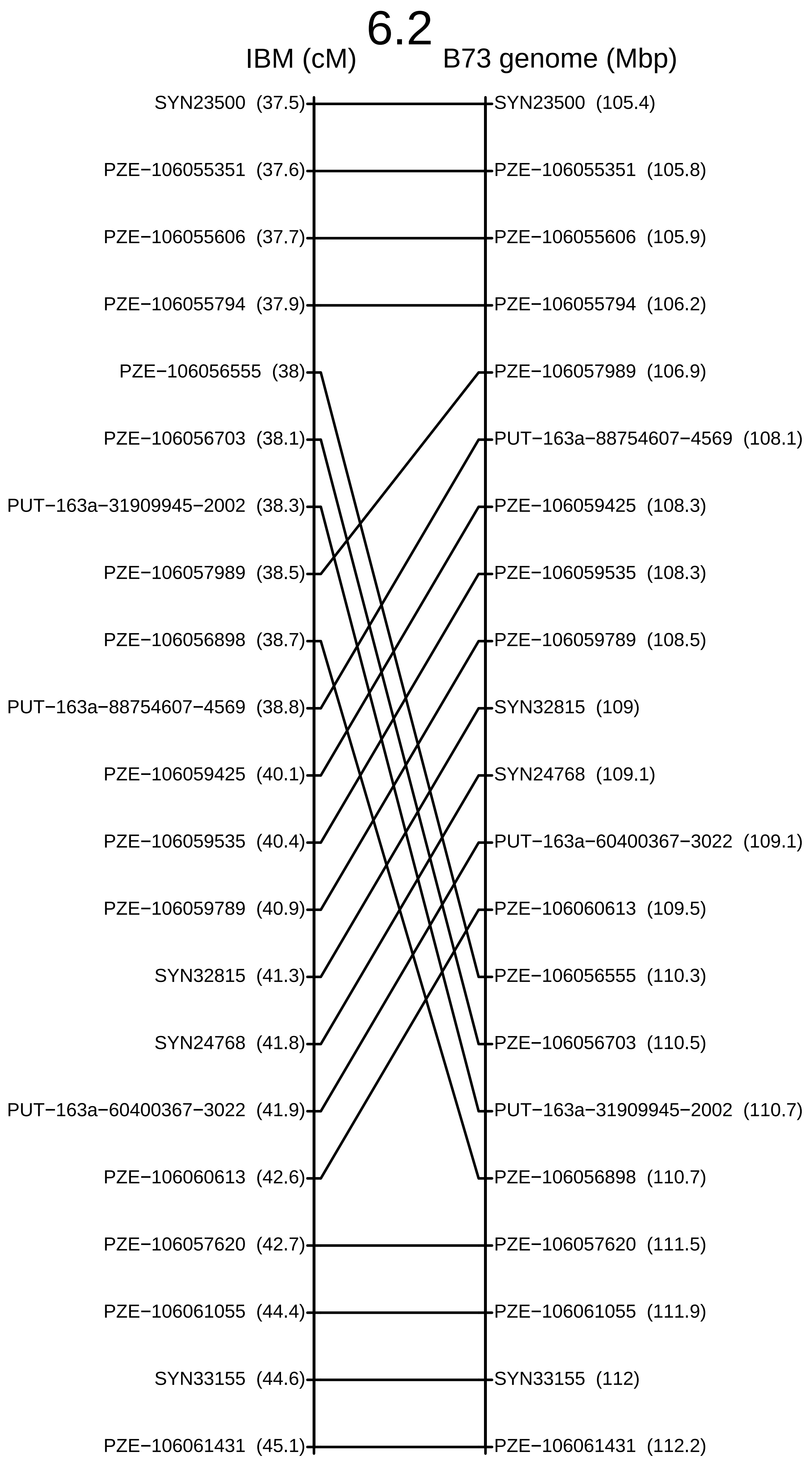
6.2
IBM (cM)
B73 genome (Mbp)
SYN23500 (37.5)
PZE−106055351 (37.6)
PZE−106055606 (37.7)
PZE−106055794 (37.9)
PZE−106056555 (38)
PZE−106056703 (38.1)
PUT−163a−31909945−2002 (38.3)
PZE−106057989 (38.5)
PZE−106056898 (38.7)
PUT−163a−88754607−4569 (38.8)
PZE−106059425 (40.1)
PZE−106059535 (40.4)
PZE−106059789 (40.9)
SYN32815 (41.3)
SYN24768 (41.8)
PUT−163a−60400367−3022 (41.9)
PZE−106060613 (42.6)
PZE−106057620 (42.7)
PZE−106061055 (44.4)
SYN33155 (44.6)
PZE−106061431 (45.1)
SYN23500 (105.4)
PZE−106055351 (105.8)
PZE−106055606 (105.9)
PZE−106055794 (106.2)
PZE−106057989 (106.9)
PUT−163a−88754607−4569 (108.1)
PZE−106059425 (108.3)
PZE−106059535 (108.3)
PZE−106059789 (108.5)
SYN32815 (109)
SYN24768 (109.1)
PUT−163a−60400367−3022 (109.1)
PZE−106060613 (109.5)
PZE−106056555 (110.3)
PZE−106056703 (110.5)
PUT−163a−31909945−2002 (110.7)
PZE−106056898 (110.7)
PZE−106057620 (111.5)
PZE−106061055 (111.9)
SYN33155 (112)
PZE−106061431 (112.2)

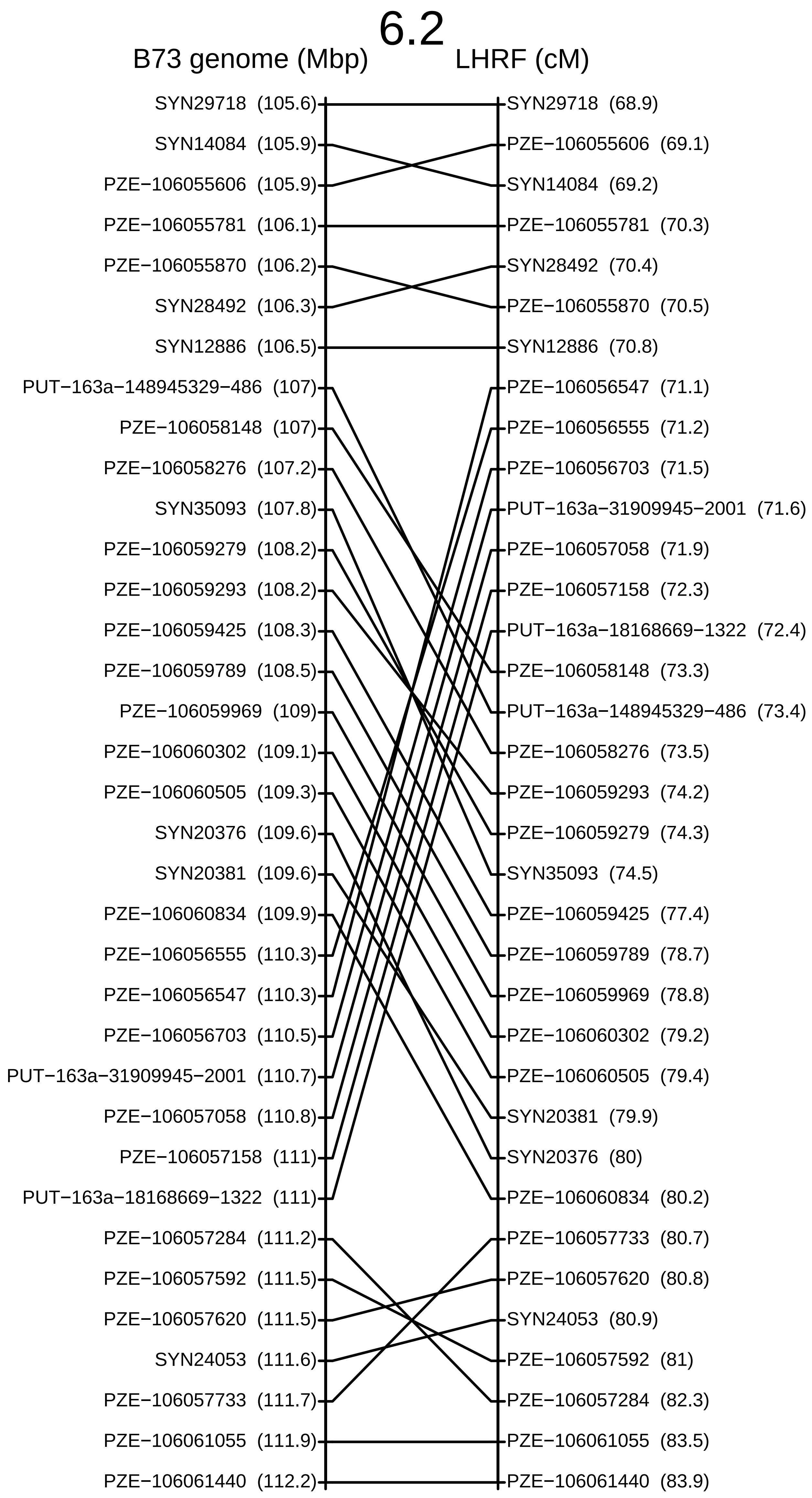
6.2
B73 genome (Mbp)
LHRF (cM)
SYN29718 (105.6)
SYN14084 (105.9)
PZE−106055606 (105.9)
PZE−106055781 (106.1)
PZE−106055870 (106.2)
SYN28492 (106.3)
SYN12886 (106.5)
PUT−163a−148945329−486 (107)
PZE−106058148 (107)
PZE−106058276 (107.2)
SYN35093 (107.8)
PZE−106059279 (108.2)
PZE−106059293 (108.2)
PZE−106059425 (108.3)
PZE−106059789 (108.5)
PZE−106059969 (109)
PZE−106060302 (109.1)
PZE−106060505 (109.3)
SYN20376 (109.6)
SYN20381 (109.6)
PZE−106060834 (109.9)
PZE−106056555 (110.3)
PZE−106056547 (110.3)
PZE−106056703 (110.5)
PUT−163a−31909945−2001 (110.7)
PZE−106057058 (110.8)
PZE−106057158 (111)
PUT−163a−18168669−1322 (111)
PZE−106057284 (111.2)
PZE−106057592 (111.5)
PZE−106057620 (111.5)
SYN24053 (111.6)
PZE−106057733 (111.7)
PZE−106061055 (111.9)
PZE−106061440 (112.2)
SYN29718 (68.9)
PZE−106055606 (69.1)
SYN14084 (69.2)
PZE−106055781 (70.3)
SYN28492 (70.4)
PZE−106055870 (70.5)
SYN12886 (70.8)
PZE−106056547 (71.1)
PZE−106056555 (71.2)
PZE−106056703 (71.5)
PUT−163a−31909945−2001 (71.6)
PZE−106057058 (71.9)
PZE−106057158 (72.3)
PUT−163a−18168669−1322 (72.4)
PZE−106058148 (73.3)
PUT−163a−148945329−486 (73.4)
PZE−106058276 (73.5)
PZE−106059293 (74.2)
PZE−106059279 (74.3)
SYN35093 (74.5)
PZE−106059425 (77.4)
PZE−106059789 (78.7)
PZE−106059969 (78.8)
PZE−106060302 (79.2)
PZE−106060505 (79.4)
SYN20381 (79.9)
SYN20376 (80)
PZE−106060834 (80.2)
PZE−106057733 (80.7)
PZE−106057620 (80.8)
SYN24053 (80.9)
PZE−106057592 (81)
PZE−106057284 (82.3)
PZE−106061055 (83.5)
PZE−106061440 (83.9)

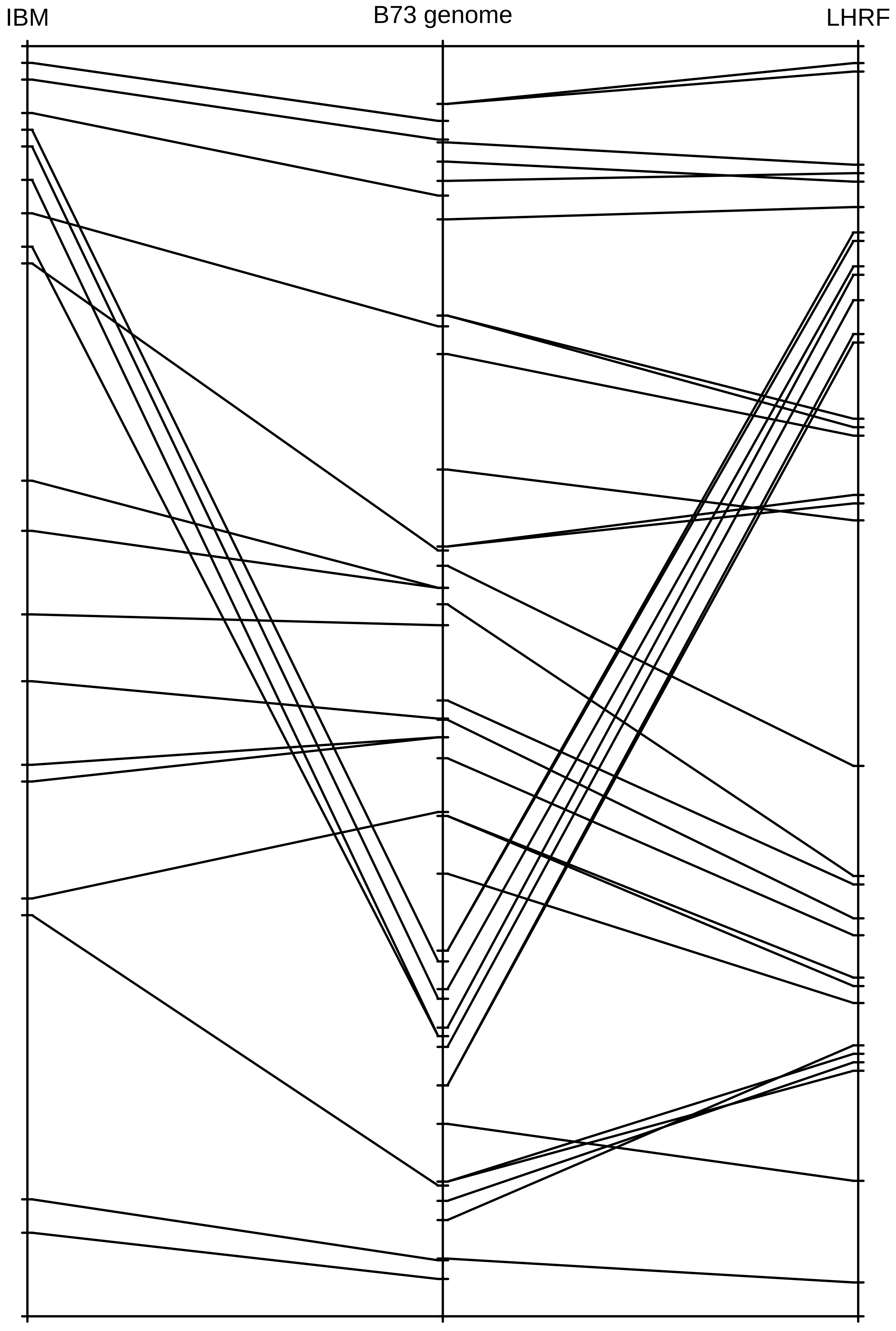
IBM
B73 genome
LHRF

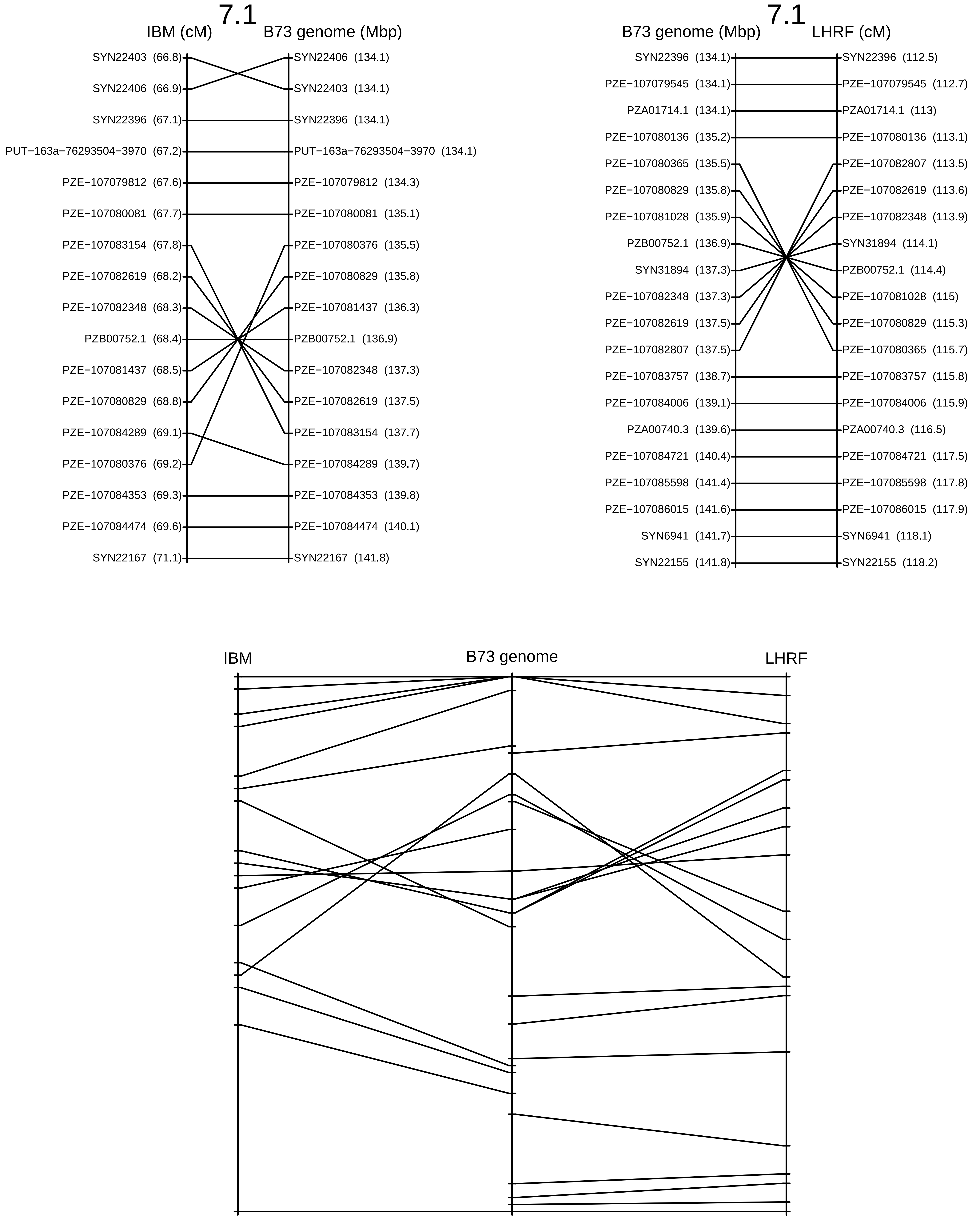
7.1
IBM (cM)
B73 genome (Mbp)
SYN22403 (66.8)
SYN22406 (66.9)
SYN22396 (67.1)
PUT−163a−76293504−3970 (67.2)
PZE−107079812 (67.6)
PZE−107080081 (67.7)
PZE−107083154 (67.8)
PZE−107082619 (68.2)
PZE−107082348 (68.3)
PZB00752.1 (68.4)
PZE−107081437 (68.5)
PZE−107080829 (68.8)
PZE−107084289 (69.1)
PZE−107080376 (69.2)
PZE−107084353 (69.3)
PZE−107084474 (69.6)
SYN22167 (71.1)
SYN22406 (134.1)
SYN22403 (134.1)
SYN22396 (134.1)
PUT−163a−76293504−3970 (134.1)
PZE−107079812 (134.3)
PZE−107080081 (135.1)
PZE−107080376 (135.5)
PZE−107080829 (135.8)
PZE−107081437 (136.3)
PZB00752.1 (136.9)
PZE−107082348 (137.3)
PZE−107082619 (137.5)
PZE−107083154 (137.7)
PZE−107084289 (139.7)
PZE−107084353 (139.8)
PZE−107084474 (140.1)
SYN22167 (141.8)
7.1
B73 genome (Mbp)
LHRF (cM)
SYN22396 (134.1)
PZE−107079545 (134.1)
PZA01714.1 (134.1)
PZE−107080136 (135.2)
PZE−107080365 (135.5)
PZE−107080829 (135.8)
PZE−107081028 (135.9)
PZB00752.1 (136.9)
SYN31894 (137.3)
PZE−107082348 (137.3)
PZE−107082619 (137.5)
PZE−107082807 (137.5)
PZE−107083757 (138.7)
PZE−107084006 (139.1)
PZA00740.3 (139.6)
PZE−107084721 (140.4)
PZE−107085598 (141.4)
PZE−107086015 (141.6)
SYN6941 (141.7)
SYN22155 (141.8)
SYN22396 (112.5)
PZE−107079545 (112.7)
PZA01714.1 (113)
PZE−107080136 (113.1)
PZE−107082807 (113.5)
PZE−107082619 (113.6)
PZE−107082348 (113.9)
SYN31894 (114.1)
PZB00752.1 (114.4)
PZE−107081028 (115)
PZE−107080829 (115.3)
PZE−107080365 (115.7)
PZE−107083757 (115.8)
PZE−107084006 (115.9)
PZA00740.3 (116.5)
PZE−107084721 (117.5)
PZE−107085598 (117.8)
PZE−107086015 (117.9)
SYN6941 (118.1)
SYN22155 (118.2)
IBM
B73 genome
LHRF

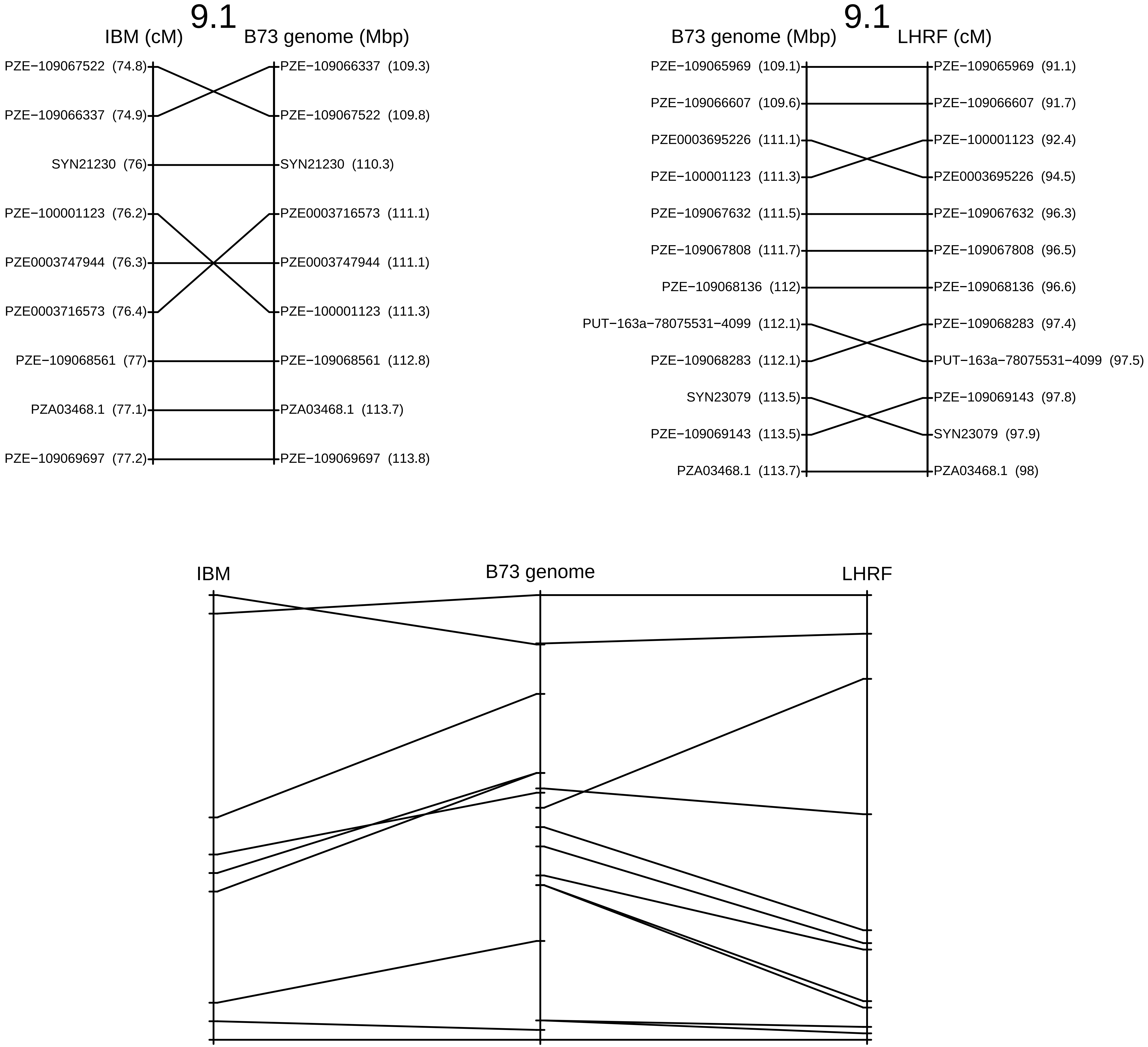
9.1
IBM (cM)
B73 genome (Mbp)
PZE−109067522 (74.8)
PZE−109066337 (74.9)
SYN21230 (76)
PZE−100001123 (76.2)
PZE0003747944 (76.3)
PZE0003716573 (76.4)
PZE−109068561 (77)
PZA03468.1 (77.1)
PZE−109069697 (77.2)
PZE−109066337 (109.3)
PZE−109067522 (109.8)
SYN21230 (110.3)
PZE0003716573 (111.1)
PZE0003747944 (111.1)
PZE−100001123 (111.3)
PZE−109068561 (112.8)
PZA03468.1 (113.7)
PZE−109069697 (113.8)
9.1
B73 genome (Mbp)
LHRF (cM)
PZE−109065969 (109.1)
PZE−109066607 (109.6)
PZE0003695226 (111.1)
PZE−100001123 (111.3)
PZE−109067632 (111.5)
PZE−109067808 (111.7)
PZE−109068136 (112)
PUT−163a−78075531−4099 (112.1)
PZE−109068283 (112.1)
SYN23079 (113.5)
PZE−109069143 (113.5)
PZA03468.1 (113.7)
PZE−109065969 (91.1)
PZE−109066607 (91.7)
PZE−100001123 (92.4)
PZE0003695226 (94.5)
PZE−109067632 (96.3)
PZE−109067808 (96.5)
PZE−109068136 (96.6)
PZE−109068283 (97.4)
PUT−163a−78075531−4099 (97.5)
PZE−109069143 (97.8)
SYN23079 (97.9)
PZA03468.1 (98)
IBM
B73 genome
LHRF
