# Supplementary material for: A Large Maize (Zea mays L.) SNP Genotyping Array: Development and Germplasm Genotyping, and Genetic Mapping to Compare with the B73 Reference Genome
Source: PLoS One. 2011 Dec 8;6(12):e28334. doi: 10.1371/journal.pone.0028334 (PMC3234264; doi:10.1371/journal.pone.0028334)
Supplement: Text S1 — Detailed methods. More detailed description of procedures and parameters used for the development of the SNP array and the construction of the IBM and LHRF genetic maps. (PDF) [file pone.0028334.s020.pdf]

SUPPLEMENTARY TEXT: DETAILED METHODS

Selection of SNP markers for the array

For the selection of SNPs in genes identified with high certainty, the published set of 32,540 filtered genes (containing introns and flanking sequences) was used. For the assignment of SNPs to maize genes a BLAST search of SNP border sequences against the Filtered gene set (release 4.53a, 16-Oct-2009, http://ftp.maizesequence.org/release-4a.53/filtered-set/ZmB73_4a.53_filtered_genes_500.fasta.gz) was performed.

In order to assign physical mapping positions to the SNPs, a stand-alone BLAST search of the SNP flanking sequences was performed against the then available maize genome assembly (release 4.53a, 16-Oct-2009, http://ftp.maizesequence.org/release-4a.53/assembly/). Search parameters were: blastall -p blastn -a 2 -b 1 -v 1 -m 8 -e 1e-30.

Prior to the final selection of the SNPs for the array, sequences located in highly repetitive DNA sequences were eliminated by running the cross_match program (http://www.phrap.org/phredphrapconsed.html#block_phrap) using TIGR_Zea_Repeats.v3.0 (TIGR_Plant_Repeats: ftp://ftp.plantbiology.msu.edu/pub/data/TIGR_Plant_Repeats/TIGR_Zea_Repeats.v3.0) as target sequences (cross_match parameters: minmatch 12, penalty 2 and minscore 20).

SNP marker selection based on the B73 sequence

The last selection step (Step 4) was based on the genomic maize sequence in order to saturate those regions that had been insufficiently covered in the gene-based selection steps described above. It was performed in two steps: In Step 4a, the genomic sequence of each chromosome was divided into regions of 1 Mb. Starting with the SNPs selected in Steps 1-3, further SNP were selected in the following way: SNPs mapped to each 1Mb region were selected in descending order of their assay design score. This selection step was done in 17 cycles. To each lowly saturated region, one SNP was assigned per region and cycle. Excessively covered regions were skipped in earlier cycles when their

SNP saturation was higher than the current cycle number. In selection Step 4b, the 1Mb regions defined in Step 4a were subdivided into 100 kb pieces. The selection procedure was similar to Step 4a but with 9 cycles only in order to achieve a relatively even distribution of the markers.

Building linkage maps

The IBM and LHRF linkage maps were constructed independently, using the same procedures with the same parameters. The different steps for building each of our linkage maps were as follows. First we selected which markers to use; second, a scaffold map with well separated markers was generated to maintain a high level of robustness; third, this scaffold map was densified, leading to the "framework map"; fourth, additional markers were placed, leading to a placement-augmented map hereafter referred to as "complete map".

Selection of markers

For all the map constructions, only polymorphic markers which were homozygous in the two founding parents of the respective IRILs were used. When a genotype data point was heterozygous, it was replaced by a missing data point.

Construction of IBM and LHRF scaffold maps

The starting point was a list of markers selected as described above which is hereafter referred to as the pool. If there were enough markers in the pool any of these markers could be used as a seed to further aggregate markers into a scaffold map of one chromosome. We used an iterative aggregation process in the following way: for each marker in the pool, we tentatively placed it in all the possible positions of the current scaffold map using the "buildfw" command of CarthaGene. If the current map has *k* markers, one can place the new marker in (*k-1*) internal intervals or in the two outside regions. The best position was determined. If it was in one of the internal intervals, the marker was removed from the pool and was no longer considered. The marker was also removed from consideration if its distance from the closest marker on the map was less than 10 cM. If this distance was larger than 10 cM, a score for the marker was obtained as the two-point LOD of its linkage with its closest neighbor (using the "mrklod2p" command of CarthaGene). Having considered all possible markers in the pool, the one with the best score was selected provided that this score was higher than ten. If this

was the case, the marker was added to the scaffold. The order of the map was then re-computed using the “lkh” command of CarthaGene, which is based on a state-of-the-art algorithm able to find the best order for close to a hundred markers within reasonable computation times. This addition resulted in a new current scaffold map with one extra marker. The process was iterated until no more markers fulfilled the criterion for addition.

The construction of the scaffold was purely deterministic but depended on the seed marker. In practice, because our data set, which although being large, had some gaps, different seed markers produced maps of variable quality (in particular chromosome extremities can be more or less well covered). To be sure to obtain high-quality maps for each chromosome, we performed several rounds based on different choices of seed markers with each replicate producing one scaffold map for each chromosome. Subsequently, for each replicate we produced the associated framework map for each chromosome (see next paragraph). The best framework map defined as the one with the largest number of markers was then selected for all subsequent steps. The use of random seed markers could be done completely blindly, repeating the attempts until each replicate has a scaffold for each chromosome. In practice, we used a more efficient procedure so that each seed marker should lead to a different chromosome. Specifically, for each replicate, we randomly took one seed marker among those that were assigned to the chromosome of interest according to the B73 genome assembly. This is the only case where we made use of the B73 genome information for building the genetic maps. Since for each replicate ten different scaffolds were constructed, any error in the B73 chromosomal assignment of seed markers would be detectable since two scaffolds would have their markers strongly linked. No such cases were found, justifying *a posteriori* this use of the B73 genome, which allowed to gain computation efficiency compared to the blind procedure.

Construction of the IBM and LHRF framework maps

The starting point was a scaffold map for each chromosome, as previously described. These maps were expected to be extremely robust because of the well separated markers. In particular, we took at face value the predicted marker order and preserved it while markers were added to build the framework map.

The first step consisted in assigning all markers (using the strictly filtered data set) to linkage

groups. Given a candidate marker and a chromosome, the marker's group score was defined as the highest two-point LOD between it and all markers in the scaffold map of that chromosome. For each candidate marker, its ten group scores were computed and the marker was assigned to the group having the highest group score, provided that (1) its best two-point LOD score was at least equal to six, and (2) the difference with the second best group score was at least six. This led to ten pools of markers, each of which could then be used to densify the scaffold map for its chromosome.

From a scaffold map, a framework map was developed by adding markers to fill the largest gaps. The process was iterative. Given the current map, candidate markers in the pool were selected and inserted using the "buildfw" command of CarthaGene (similar to the "try" command of MapMaker). This produced a LOD score for each interval in the scaffold. If the best LOD score differed by at least six units from that of the second best interval, the candidate was kept for the next test, otherwise it was discarded. To check that the order of the whole map after insertion was still robust, the "flips" command of CarthaGene (equivalent to the "ripple" command of MapMaker) was used. The candidate marker was accepted and inserted in the map if none of the orderings tested by "flips" (window size equals five) had a LOD score less than four compared to the default order. The densification was finished when all the markers of the pool had been examined. The result was a framework map, in which gaps had been reduced and marker order was still statistically robust.

Having done this for each chromosome of the current replicate, we applied the same procedures for the ten different replicates. Then, each chromosome had ten candidate framework maps; we kept the one having the largest number of markers.

Placing additional markers onto the IBM and LHRF framework map

The placement of markers onto the framework map was done independently for all markers, considering them one at a time. For a given marker, it was first assigned to a linkage group using exactly the same procedure and thresholds as described above for assigning markers to linkage groups of the scaffold maps, except that the framework map was used here instead of the scaffold map.

For each candidate marker which was successfully assigned to a chromosome, it was then tentatively inserted into each of the intervals of the corresponding framework map, as well as above the

first and below the last of the framework markers. The “buildfw” command of CarthaGene, which provides the likelihood for each possible insertion was used and the genetic distance to the flanking markers was calculated. The corresponding information was recorded for all markers, producing a list of “placed” markers whose positions were less strongly statistically supported than those of the framework map.

For both IBM and LHRF populations, we finally built the “complete map” consisting of both the framework markers and the placed markers along with their positions.

Computing centiMorgan (cM) distances

Intermated Recombinant Inbred Lines (IRILs) require the use of a specific method to compute genetic distances in centiMorgan (Winkler et al. 2003; Falque 2005). 100 cM must correspond to the distance over which the average number of crossovers per meiosis at the gamete level is one. From the observed fraction of recombinant IRILs, one can compute such cM by using the appropriate formula but often genetic distances from IRILs are computed as if the lines were normal RILs. This produces “pseudo-cM” distances which are overestimations by a non-constant factor close to two (Falque et al. 2005). In this work, we used “true” centiMorgan distances, but provided also “pseudo-cM”.

Segregation bias

For a given IRIL population of parents P_1 and P_2, we call N_1 (respectively N_2) the number of plants having at a particular locus the allele of P_1 (respectively of P_2). To estimate the statistical significance of the segregation distortion at that locus, it was tested whether the hypothesis of no distortion (a fraction compatible with 0.5 for each allele) resulted in a *p*-value smaller than 1%. This defined a region outside of an interval centered on the value 0.5; the half-width of this interval is 2.33 s where s is the standard error satisfying $s^2 = 1 /(4 (N_1 + N_2))$. The associated bands for all chromosomes and for the IRILs IBM and LHRF were slightly irregular because the number of valid data varied at each marker.

Identification of non-colinear regions between genetic and physical maps

A map comparison, represented by connecting positions of markers from a genetic and a physical map, becomes difficult to display when many markers are used. The reason for this is that the genetic and physical distances vary, so the connecting lines can be very slanted, making it difficult to detect non-colinearities. To overcome this drawback, “ladder diagrams” were used in which the position of each marker is determined by its index in the ordered map instead of its genetic and physical coordinate. In the case of two colinear maps, such a diagram produces a regular ladder with equi-spaced horizontal rungs which helps to visually detect non-colinearities.

Because of the high robustness of the marker order in framework maps, these maps were used first to detect non-colinearities between genetic and physical maps. The simplest cases corresponded to having two adjacent markers being inverted. More complex non-colinearities arose if one marker was displaced more than one marker away from its colinear position, or if multiple markers had to be moved to restore colinearity. To be able to examine these regions, we delimited segments on the physical map. First, a segment has to begin and end with two markers whose rungs are horizontal in the ladder diagram. Second, these markers should surround a complex non-colinear situation as defined above. This selection of regions was applied to each chromosome, for both IBM and LHRF genetic maps, from which we assigned names as follows. For a given chromosome, we ordered the regions according to their increasing physical coordinates. If IBM and LHRF had a strongly overlapping region, it was counted just once. We then labeled these regions as *n*.1, *n*.2, … if they resided on chromosome *n*. In addition, the suffix “I” or “L” was added to such a region label to indicate that it originated from the IBM or LHRF map respectively.

REFERENCES

Falque M (2005) IRILmap: linkage map distance correction for intermated recombinant inbred lines/advanced recombinant inbred strains. Bioinformatics 21: 3441-3442.

Winkler CR, Jensen NM, Cooper M, Podlich DW, Smith OS (2003) On the Determination of Recombination Rates in Intermated Recombinant Inbred Populations. Genetics 164: 741 -745.
